# Chromothripsis during telomere crisis is independent of NHEJ, and consistent with a replicative origin

**DOI:** 10.1101/gr.240705.118

**Published:** 2019-05

**Authors:** Kez Cleal, Rhiannon E. Jones, Julia W. Grimstead, Eric A. Hendrickson, Duncan M. Baird

**Affiliations:** 1 Division of Cancer and Genetics, School of Medicine, Cardiff University, Heath Park, Cardiff, CF14 4XN, United Kingdom;; 2 Department of Biochemistry, Molecular Biology, and Biophysics, University of Minnesota Medical School, Minneapolis, Minnesota 55455, USA

## Abstract

Telomere erosion, dysfunction, and fusion can lead to a state of cellular crisis characterized by large-scale genome instability. We investigated the impact of a telomere-driven crisis on the structural integrity of the genome by undertaking whole-genome sequence analyses of clonal populations of cells that had escaped crisis. Quantification of large-scale structural variants revealed patterns of rearrangement consistent with chromothripsis but formed in the absence of functional nonhomologous end-joining pathways. Rearrangements frequently consisted of short fragments with complex mutational patterns, with a repair topology that deviated from randomness showing preferential repair to local regions or exchange between specific loci. We find evidence of telomere involvement with an enrichment of fold-back inversions demarcating clusters of rearrangements. Our data suggest that chromothriptic rearrangements caused by a telomere crisis arise via a replicative repair process involving template switching.

Telomere erosion occurs during cell division due to the inability of semiconservative DNA replication to fully replicate linear chromosomes (Harley et al. 1990; Oeseburg et al. 2010). In most cells, telomere attrition will ultimately trigger apoptosis or senescence, preventing further shortening and telomere dysfunction (Hayashi et al. 2015). In cells that bypass such checkpoints, further losses can disrupt the telomere nucleoprotein complex, exposing the telomere repeat array to the DNA repair machinery and resulting in the formation of telomere fusions (Counter et al. 1992). Telomere dysfunction and genome instability lead to a state known as “crisis” that is characterized by massive cell death, and provide a strong selection pressure for reestablishment of functional telomeres through telomerase reactivation or the alternative lengthening of telomeres (ALT) pathway (Shay and Bacchetti 1997; Jones et al. 2014; Hayashi et al. 2015; Pickett and Reddel 2015).

Recent studies suggest telomere fusions are mediated by classical (C-NHEJ) and alternative (A-NHEJ) nonhomologous end joining (Smogorzewska et al. 2002; Bunting and Nussenzweig 2013; Liddiard et al. 2016). C-NHEJ typically involves blunt-end ligation by the XRCC4:LIG4 complex (Critchlow et al. 1997; Walker et al. 2001; Chang et al. 2017), whereas A-NHEJ uses LIG3, with an auxiliary role for LIG1 (Simsek et al. 2011; Lu et al. 2016; Liddiard et al. 2018). A-NHEJ additionally has a requirement for microhomology that can be exposed by preprocessing of the DNA end (Sfeir and Symington 2015; Chang et al. 2017).

Telomere fusions lead to the formation of dicentric chromosomes that may be resolved during or following mitosis by breakage of the conjoining sequence (Maciejowski et al. 2015; Maciejowski and de Lange 2017). The resulting free ends may initiate further rounds of fusion, giving rise to a breakage–fusion–bridge cycle (BFB) (Murnane 2012). Such cycles can generate a myriad of structural diversity and are thought to play a prominent role in driving cancer genome restructuring (Artandi and DePinho 2010; Hermetz et al. 2014; Cleal et al. 2018).

Recently, telomere dysfunction has been implicated in the generation of chromothripsis, a large-scale genome rearrangement configuration seen in many cancers and some congenital disorders (Li et al. 2014; Maciejowski et al. 2015; Mardin et al. 2015). Although few details have been established, chromothripsis is thought to result from a single event involving localized shattering of one or more chromosomes followed by random joining with loss of some fragments. The resulting patterns of randomly oriented and positioned fragments interspersed by regions showing loss of heterozygosity have been taken as evidence of a singular process, although others have cautioned a multistep process may be plausible (Kinsella et al. 2014). BFB resolution has been suggested to be the de facto event that underlies chromothripsis (Maciejowski et al. 2015).

Additional mechanisms have also been proposed to explain the chaotic rearrangements seen in cancer. In prostate cancer, structural variants (SVs) resembled a chain linking multiple chromosomes, with up to 88% of samples containing a chain with five or more SVs, leading to the conclusion that a unique mechanism, termed chromoplexy, was at play (Baca et al. 2013). In contrast, chromoanasynthesis was reported to result from a replicative mechanism, due to the identification of insertions of 50–1500 nt sandwiched between larger fragments and a predominance of copy number (CN) gains (Liu et al. 2011). However, the similarities and distinctions between these processes have yet to be evaluated, and it is unclear which repair pathways are used and to what extent telomere crisis drives these genome rearrangements.

## Results

### Investigating the escape from a telomere crisis

Recent reports have implicated C- and A-NHEJ in the repair of telomere fusions (Jones et al. 2014; Liddiard et al. 2016). Here, we investigated the consequences of a transit through crisis in the context of NHEJ deficiency, using HCT116 knockout cells, comprising *LIG4*^−/−^, a double *LIG3*^−/−^:*LIG4*^−/−^, a *LIG3* knockout line with a supra-normal complementation of nuclear LIG3 (*LIG3*^−/−:NC3^) (Oh et al. 2014), and a double *TP53*^−/−^:*LIG3*^−/−^ knockout (Supplemental Table 1; Supplemental Fig. S1; Supplemental Methods; Oh et al. 2014). We have previously shown that HCT116 *LIG3*^−/−^ cells are unable to escape crisis (Jones et al. 2014); however, the additional knockout of *TP53* in *LIG3*^−/−^ cells permitted escape and sequencing of post-crisis samples (Supplemental Fig. S2A).

Escape from crisis was modeled using an in vitro system that entailed continual passage of HCT116 following dominant-negative *TERT* expression (DN-*TERT*) (Hahn et al. 1999; Jones et al. 2014). We have previously documented that DN-*TERT* expression resulted in progressive telomere erosion to a low point at crisis, following which telomeres lengthened coincident with reestablishment of telomerase activity (Supplemental Fig. S3; Jones et al. 2014). Entry into crisis was characterized by a deflection in the growth curve (Supplemental Fig. S2), and following a period of slowed or absent growth, cultures escaped crisis and resumed continuous growth (Hahn et al. 1999; Jones et al. 2014). *LIG3*^−/−:NC3^ cells spent longer in crisis relative to wild type (WT; *P*-value <0.05, Mann–Whitney *U* test) (Supplemental Fig. S2G), although no differences were identified in other backgrounds. Time in crisis was weakly associated with SVs (Spearman's rho 0.46, *P*-value 0.0057) (Supplemental Fig. S2H) but not with vector integration rates (Supplemental Fig. S2I; Supplemental Methods); thus, the reason for increased duration of crisis in *LIG3*^−/−:NC3^ cells was unclear, but may relate to the error-prone and deleterious nature of A-NHEJ repair in *LIG3*^−/−:NC3^ (Wang and Xu 2017).

WT clones displayed the fastest transit through crisis with the possibility that multiple cells escaped crisis, resulting in a mixed population with subclonal SVs arising from telomere dysfunction. To ensure clonal analysis, 10 additional clones were taken from post-crisis cultures, providing a total of 15× WT. *LIG3*^−/−^:*LIG4*^−/−^ cells were compromised in their ability to escape crisis, with only three of 29 clones escaping (Jones et al. 2014). To obtain sufficient samples, cloning was modified so post transfection with DN-*TERT*, cells were split into 10 cultures and propagated through crisis. Single-cell escapees were then isolated and used to establish clonal cultures, providing a total of 10× *LIG3*^−/−^:*LIG4*^−/−^ (Supplemental Fig. S1; Supplemental Table 1).

Following escape from crisis, whole-genome paired-end sequencing was performed (Supplemental Table 1). Evidence of polyclonality could be gathered from assessing B-allele frequencies (nonreference allele) in the context of CN changes, along with assessing unique variant allele frequency (VAF) profiles in diploid regions (Supplemental Figs. S4–S8; Supplemental Methods). We inferred that 80% of post-crisis samples were monoclonal, with 13% showing evidence of polyclonality and 7% of unknown clonality. Mono- and polyclonal cultures showed no difference in crisis duration (41.5 and 44.5 d, respectively; *P*-value 0.86, two tailed *t*-test).

### Telomere crisis is associated with a prevalence of CN gains

**Figure 1.**
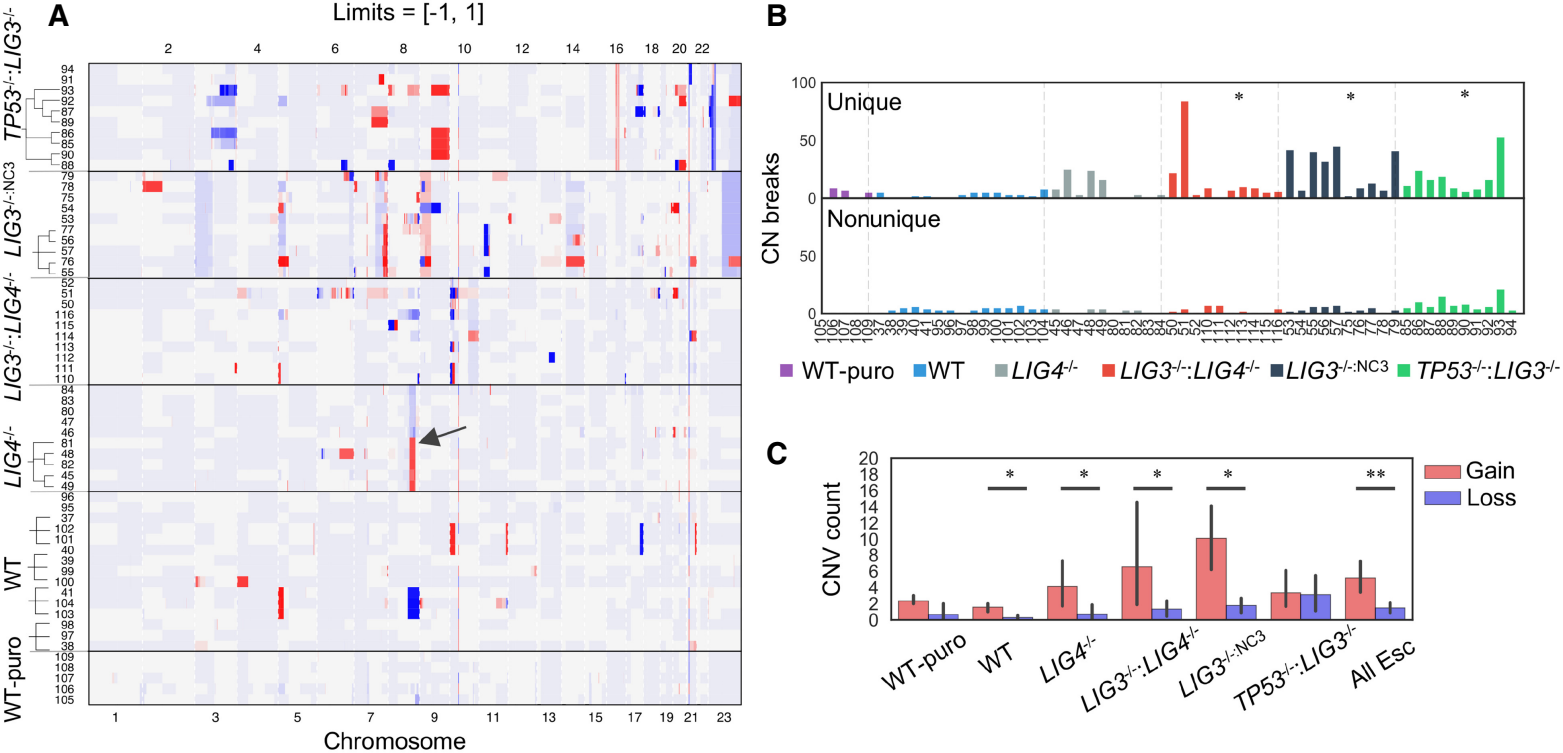
Copy number (CN) variation associated with a telomere crisis. (*A*) Cell lines with deficiencies for C- and A-NHEJ were driven through a telomere crisis by expression of DN-*TERT* with clonal selection occurring either pre- or post-crisis. Genomic CN changes of clones are presented as normalized values relative to the parental lines within the limits of +1 gain and −1 loss. Some samples appeared to share common CN breakpoints with other samples of the same genetic background, with an example from *LIG4*^−/−^ Chr 8q highlighted with a black arrow. (*B*) Unique and nonunique breaksites were analyzed, revealing an increase in unique sites for *LIG3*^−/−^:*LIG4*^−/−^, *LIG3*^−/−:NC3^, and *TP53*^−/−^:*LIG3*^−/−^ samples relative to WT. (*) *P*-value <0.002, Mann–Whitney *U* test. (*C*) CN segments for which both breaksites were unique were quantified, indicating a prevalence of CN gains relative to losses. (*) *P*-value <0.03. “All Esc” refers to a pooled analysis of all post-crisis clones. (**) *P*-value <2 × 10^−5^, Mann–Whitney *U* test.

Large-scale CN variants (CNVs) from post-crisis clones were plotted as a heat map with gains in red and losses in blue, and segment means limited to the range (+1, −1) (Fig. 1A; Supplemental Fig. S9; Supplemental Methods). CNVs were apparent in all post-crisis samples with an abundance in *LIG3*^−/−^:*LIG4*^−/−^, *LIG3*^−/−:NC3^, and *TP53*^−/−^:*LIG3*^−/−^ (Fig. 1B). The *TP53*^−/−^:*LIG3*^−/−^ parental line appeared to have already experienced a genome catastrophe, evident from the large number of CNVs across multiple chromosomes (Supplemental Fig. S9). Presumably loss of the TP53 DNA damage checkpoint was sufficient to foster the emergence and eventual dominance of a clone with this karyotype without the need to transit crisis.

Some post-crisis samples shared identical CNV breakpoints, suggesting they were derived from a common subpopulation within the parental line (Supplemental Fig. S10). Supporting this interpretation, the common Chr 8q amplification in *LIG4*^−/−^ cells (Fig. 1A, black arrow) was detected at low frequency in the parental line (Supplemental Fig. S10), implying that common CNVs resulted from outgrowth of subclones within the parental line.

*LIG3*^−/−^:*LIG4*^−/−^, *LIG3*^−/−:NC3^, and *TP53*^−/−^:*LIG3*^−/−^ samples showed increased numbers of unique CNV breaksites relative to WT (*P*-value <0.002, Mann–Whitney *U* test) (Fig. 1B), whereas no differences were identified for nonunique breaksites (*P*-value 0.085, Levene's test). CN events were quantified by counting segments with unique start and end breakpoints, revealing a pattern of increased CN gains for 4/5 post-crisis samples, with only *TP53*^−/−^:*LIG3*^−/−^ showing a balanced profile (*P*-value <0.03, Mann–Whitney *U* test) (Fig. 1C). Although CN counts at some chromosomal arms were enhanced, changes did not reach statistical significance across samples (*P*-value >0.05, Poisson test) (Supplemental Fig. S11).

### Identification of whole-genome catastrophe

**Figure 2.**
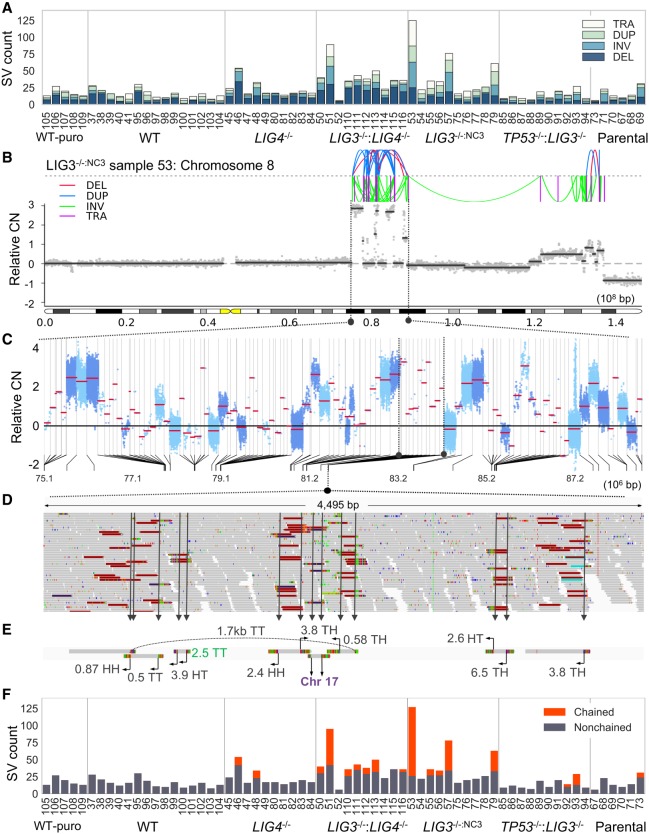
Identification of genome catastrophe in post-crisis clones. (*A*) The number of deletions (DEL), inversions (INV), duplications (DUP), and translocations (TRA) for all samples were quantified. (*B*–*E*) An example of a complex rearrangement is illustrated. To visualize the dense spatial clustering of breaks present in *B*, the lengths of segments separating neighboring breakpoints were transformed to a log_10_ scale. A region from Chr 8, indicated by black dotted lines, has been transformed in this way with the linear scale illustrated in the *lower* half of panel *C*. A further subsection of this rearrangement, highlighted with black dotted lines, is expanded in *D*, showing a 4.5-kb span with 14 breakpoints. (*E*) Assembly of discordant reads from this region was performed to aid in interpretation. Join types are given by a two-letter code corresponding to the head (H) or tail (T) ends of fragments. The distance to partner fragments is given in Mb with the arrow specifying the direction. The breakpoint colored in green was missed during SV calling. (*F*) Applying a clustering procedure, the number of SVs that resembled a chainlike pattern was quantified for each sample.

A total of 1621 unique SVs were identified (Fig. 2A), with *LIG3*^−/−^:*LIG4*^−/−^ cells displaying increased deletions and inversions and with *LIG3*^−/−:NC3^ displaying increased inversions and duplications relative to WT (*P*-value <0.05, Mann–Whitney *U* test) (Supplemental Fig. S12). However, no differences were identified between WT and WT-puro controls, indicating that, in this system, transition through crisis was not associated with escalated genome rearrangement when NHEJ was functional.

In contrast, *LIG4*^−/−^, *LIG3*^−/−^:*LIG4*^−/−^, and *LIG3*^−/−:NC3^ lines showed considerable heterogeneity in SV number, with *LIG3*^−/−:NC3^: sample 53 showing more than 120 SVs (Fig. 2A). Furthermore, samples with raised SV numbers often harbored patterns of rearrangement reminiscent of chromothripsis or other genome-wide catastrophes (Fig. 2B; Supplemental Fig. S13; Maciejowski et al. 2015). An example from *LIG3*^−/−:NC3^: Sample 53 is outlined (Fig. 2B–D), depicting an alternating multilevel CN profile and densely clustered breakpoints giving the appearance of a chain (Fig. 2B). Nearest-neighboring breakpoints within the chain were often found in extreme proximity (<150 bp), resulting in reads that sometimes straddled two breakpoints (Fig. 2D).

To illustrate this complexity, Chr 8:75–Chr 8:88 Mb was reconstructed. Segment lengths (*x*-axis) were transformed to a log_10_ scale, with the linear scale depicted below (Fig. 2C). A subregion marked with black dotted lines highlights a 4.5-kb region (Fig. 2D), showing raw reads from a dense cluster of breakpoints. Discordant reads are colored nongray with crimson reads mapping intrachromosomal events. To aid in interpretation, contigs assembled from this cluster depict 14 breakpoints, with most joined with distant intrachromosomal loci with megabase separation distances, in addition to two translocations with Chr 17 (Fig. 2E).

We developed a clustering methodology to identify and study chained SVs, which showed a low false-positive rate when validated using randomized data (Supplemental Fig. S14; Supplemental Methods). We identified chained SVs predominantly in *LIG3*^−/−^:*LIG4*^−/−^ (seven samples, *P*-value 0.0002 vs. WT, Fisher's exact test) and *LIG3*^−/−:NC3^ (five samples, *P*-value 0.0047) backgrounds with two samples from each of *LIG4*^−/−^ and *TP53*^−/−^:*LIG3*^−/−^ cell lines also showing chaining (*P*-value 0.15) (Fig. 2F). The *TP53*^−/−^:*LIG3*^−/−^ parental line also harbored chained SVs consistent with this sample having experienced a genome catastrophe prior to cloning. Chained SVs also showed random 5′ and 3′ end-joining profiles consistent with reported features of chromothripsis (Supplemental Fig. S15; Korbel and Campbell 2013).

The absence of chained SVs in WT, and obvious chained SVs in parental *TP53*^−/−^:*LIG3*^−/−^, indicates that transit through crisis is not solely sufficient to induce genome catastrophe and may require additional destabilizing influences such as a NHEJ deficiency or *TP53* loss in the absence of crisis (Korbel and Campbell 2013).

### Increased frequency of fold-back inversions in chromothripsis

**Figure 3.**
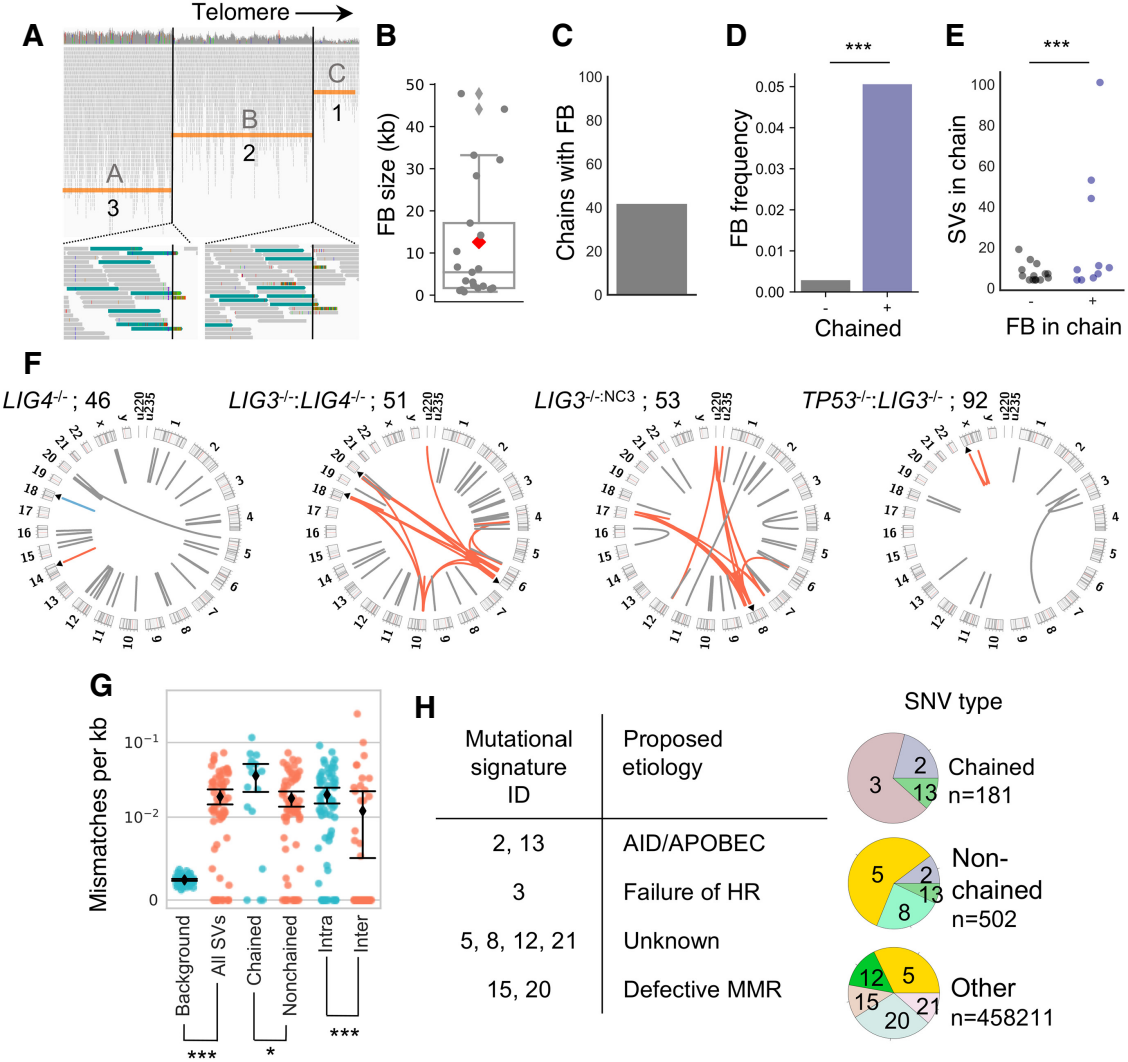
Mutational processes associated with chained rearrangements. (*A*) FB inversions were identified by a stepped CN profile across an inverted segment. The size distribution of FBs (*B*), the percentage of chains with a FB (*C*), and the FB frequency per chain (*D*) are displayed, along with the number of SVs per chain in the presence or absence of a FB (*E*). (*F*) Circos plots show chained SVs using red or blue arcs, with FB positions indicated as black arrows and nonchained SVs drawn as gray arcs. Mismatch rates in regions surrounding SV breaks were quantified (*G*), and the mutational signatures of different classes of SNVs were analyzed and plotted as pie charts, with colors depicting the different mutational signatures identified (*H*). (*) *P*-value 0.015; (***) *P*-value <4 × 10^−6^; Mann–Whitney *U* test (*D*,*G*), Fligner–Kileen test (*E*).

Fold-back (FB) inversions are a distinctive class of SV that show a characteristic steplike CN pattern, comprising a duplication to one side of an inverted disomic region with a deletion to the other (Fig. 3A; Hermetz et al. 2014). We consider the most likely mechanism to be end-to-end fusion of sister chromatids at dysfunctional chromosome ends during a BFB cycle (McClintock 1941; Campbell et al. 2010). Thus, we used FB as a surrogate marker for the involvement of dysfunctional telomeres during chained-SV formation. FB (*n* = 26) were often found with a stepped pattern decreasing toward the telomere, although some patterns decreased toward the centromere, suggesting additional rounds of BFB cycling and perhaps contributing to the CN gains across post-crisis clones (Fig. 1C). The depicted FB (Fig. 3A) from *LIG3*^−/−^:*LIG4*^−/−^: Sample 51 was situated 6.2 Mb from the Chr 6p telomere, suggesting a dicentric break may have preceded this FB, giving rise to the large terminal deletion.

Sizes of FB disomic-spacer regions (mean 5.42 kb) were consistent with previous reports (Fig. 3B; Hermetz et al. 2014). Around 42% of chains harbored one or more FB (10/24 chains harboring 17 FBs) (Fig. 3C), and the frequency of FBs was higher within a chain (*P*-value 2.9 × 10^−9^, Fisher's exact test) (Fig. 3D). However, FBs were not associated with increased numbers of SVs per chain (*P*-value 0.081, Mann–Whitney *U* test), although the variance was increased (*P*-value 5.5 × 10^−5^, Fligner–Kileen test), suggesting FBs may sometimes be associated with propagation of more complex chains (Fig. 3E).

FB locations were depicted using black triangle markers on Circos plots (Fig. 3F), and individual SV chains were drawn with colored arcs. FBs were commonly found at the edges of chained-SV clusters (9/17 FBs, *P*-value 0.02, Fisher's exact test), further suggesting a role for telomere dysfunction in chain formation.

### Mutational patterns of chromothriptic rearrangements

Recently, chromothripsis was associated with localized hypermutation known as kataegis, thought to arise as an unsolicited side effect from repair of fragmented dicentric chromosomes following mitosis (Maciejowski et al. 2015). However, using a previous method for identifying kataegic sites (KS), we did not find differences in the frequency of KS sites at chained or nonchained breakpoints in contrast to previous reports (*P*-value 0.53, Fisher's exact test) (Supplemental Fig. S16; Roberts et al. 2013; Maciejowski et al. 2015). Mutations at KS are thought to result from editing by APOBEC enzymes (Nik-Zainal et al. 2012; Alexandrov et al. 2013; Maciejowski et al. 2015), and a recent report suggested functional TP53 in HCT116 may result in reduced APOBEC activity, which may explain the low incidence of KS in our samples (Periyasamy et al. 2017).

We questioned whether SVs showed increased local mutation rates that could be correlated with APOBEC mutational signatures. After analyzing high-confidence mutations, SVs showed increased mismatch frequencies above background (7.8-fold, *P*-value 2 × 10^−7^, Mann–Whitney *U* test) (Fig. 3G). Additionally, differences were identified across chained and nonchained rearrangements (twofold, *P*-value 0.015) and intra- and interchromosomal events (1.6-fold, *P*-value 2 × 10^−6^), suggesting the activity of distinct repair pathways with mutagenic properties.

Mutational signatures of chained or nonchained SVs, in addition to all other SNVs, were then deconstructed as a linear combination of predefined signatures (Alexandrov et al. 2015; Rosenthal et al. 2016). A minor APOBEC mutational signature was identified at chained and nonchained rearrangements (signatures 2 and 13), suggesting samples showed low levels of APOBEC activity below the threshold for detection of KSs (Fig. 3H; Supplemental Fig. S17). Mutational signatures of chained and nonchained rearrangements differed, with nonchained rearrangements displaying signatures of unknown etiology (signatures 5 and 8), whereas chained SVs were associated with signature 3, which is thought to arise from defective HR, and associated with insertions and deletions that show microhomology at breaksites (Alexandrov et al. 2015; Polak et al. 2017). Other mutations displayed signatures of unknown etiology (5, 12, and 21) and patterns associated with defective mismatch repair (MMR) (15 and 20), consistent with the well-known MMR defects of HCT116. Although relatively few mutations were analyzed for chained SVs (*n* = 181), meaningful signatures have reportedly been extracted from as few as 50 mutations using the analysis software (Rosenthal et al. 2016). These data suggest that chained and nonchained rearrangements are repaired by distinct processes with differing mutagenic profiles against a background of APOBEC editing.

### Network level features of chromothripsis

**Figure 4.**
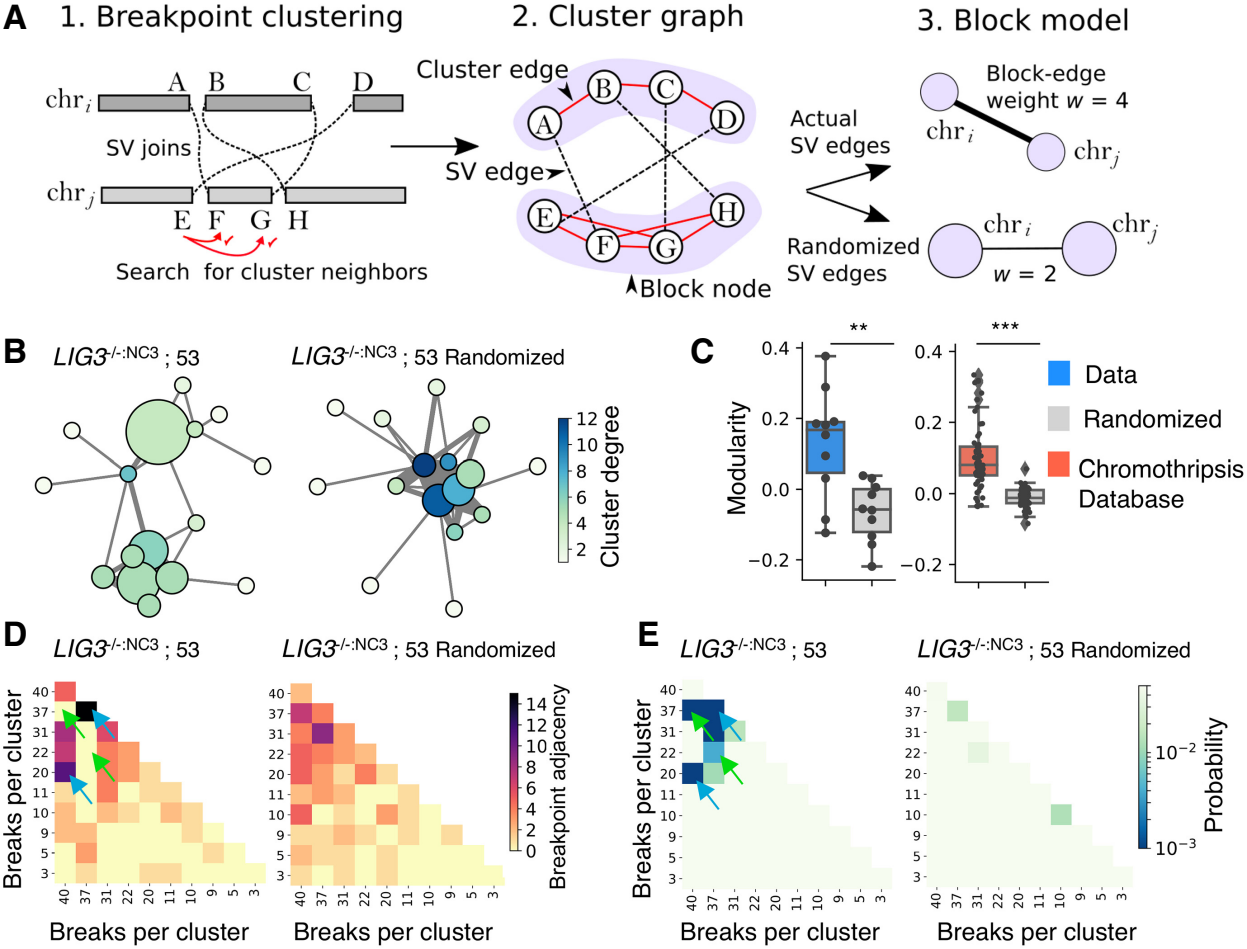
Network analysis reveals genome catastrophe joining is nonrandom. Chained breakpoints were grouped into local clusters using a statistical clustering procedure (*A1*) and represented as a cluster graph (*A2*). Components of the cluster graph that were connected by cluster edges (red edges, *A2*) were partitioned using a block model, so each node in the block model corresponds to a single cluster of breakpoints (*A3*). The example in *A* shows two clusters of breakpoints on chr_*i*_ and chr_*j*_ that are then partitioned into a block model with two nodes. Block-node diameter relates to the numbers of intracluster SVs, whereas edge thickness (weight [w]) symbolizes the number of SVs between two clusters. Block-node color represents the degree or number of SVs between all neighboring clusters. (*B*) A chain from *LIG3*^−/−:NC3^; sample 53 has been displayed in this manner along with an equivalent graph with randomized SV edges to reflect a random end-joining process. (*C*) The modularity coefficient for all chain-graphs was calculated, alongside data downloaded from the chromothripsis database (Yang et al. 2016). (*D*) Block models were represented as adjacency matrices, with each row and column corresponding to a cluster of breakpoints with the color depicting the number of intercluster SVs (nondiagonal cells) or intracluster SVs (diagonal cells). (*E*) Using simulation, the distributions of adjacency values were determined for the randomized data, allowing the probability of the observed adjacency values to be estimated, assuming a random model of end joining. Blue arrows in *D* and *E* highlight examples of clusters with more linkages than expected, whereas green arrows indicate fewer linkages.

The genome-shattering model of chromothripsis argues that fragments are randomly joined with scant consideration for the resulting genomic configuration (Ly and Cleveland 2017). By using graph theoretic approaches, we tested this hypothesis by assessing the randomness of joining. For each chain, breakpoints were represented as nodes on a graph, with edges representing SVs (Fig. 4A). Breakpoints (nodes) that formed clusters along the reference genome were then collapsed using a block model so each block-node represented a single cluster of breakpoints.

By using the same block model, breakpoint joining was then randomized while fixing the numbers of breakpoints and locations on the reference genome to reflect the purported joining process in chromothripsis (Fig. 4A). An example chain from *LIG3*^−/−:NC3^: Sample 53 is displayed, showing a qualitative difference between the real and randomized graphs with fewer intracluster SVs (represented with smaller block-node size) and increased intercluster SVs (thicker and/or more numerous block-edges) when compared to observed data (Fig. 4B; Supplemental Fig. S18).

We measured network modularity, which describes the tendency of nodes (breakpoints) to cluster into modules (Newman and Girvan 2004). A higher modularity would indicate a bias in favor of breakpoints from the same genomic cluster to connect with one another. After analyzing the distribution of modularity coefficients from all chain graphs, randomized data showed weak modularity (−0.038), whereas observed data showed a significantly higher coefficient of 0.165 (*P*-value 5.8 × 10^−3^, paired *t*-test; Fig. 4C), suggesting that chained breakpoints are frequently repaired with intracluster sites and are less prone to joining with distant loci, arguing against a completely random end-joining process.

We also analyzed SV networks listed in the chromothripsis database, analyzing 16,073 rearrangements from 53 samples (Yang et al. 2016). Database rearrangements also showed positive modularity (0.101 vs. −0.012 for randomized data, *P*-value 9 × 10^−13^, paired *t*-test), suggesting a bias toward intracluster repair is a feature of chromothripsis (Fig. 4C).

Analysis of modularity can describe a homophilic mixing pattern, but provides no information on heterophily, corresponding to preferential linking of distinct clusters within a chain. To investigate this possibility, we estimated the probability of intercluster SV enrichment in observed chain graphs by bootstrapping using Monte Carlo simulation. Visualizing our chain graphs as adjacency matrices, some clusters displayed significantly more links than random (Fig. 4D,E, blue arrows), whereas others displayed significantly fewer (Fig. 4D,E, green arrows; Supplemental Fig. S18), suggesting that repair of chained SVs can sometimes be coordinated between clusters, perhaps owing to their spatial proximity within the cell. Similar patterns were also identified across a range of cancer types listed in the chromothripsis database (Supplemental Fig. S19A–F), suggesting chromothripsis is also associated with heterophilic repair (Yang et al. 2016).

Finally, we investigated if differences in the rate of DNA breakage along a chain could be identified between our genetic backgrounds by comparing nearest-neighbor breakpoint distances across chains. However, no differences were identified, with all backgrounds showing a similar rate of breakage over an arbitrary interval, characterized by a mean rate parameter of λ = 0.099 ± 0.0065 SD (Supplemental Fig. S18I). Rearrangements listed in the chromothripsis database also showed similar spacing (λ 0.098), indicating that our data are consistent with documented chromothripsis cases.

### Chromothripsis rearrangements are consistent with a replicative origin

Chained SVs appeared to consist of short fragments, suggesting an opportunity to assemble SVs into contigs. Using SV-derived reads, 1101 were de novo assembled with a mean length of 579 bp and a longest contig of 2826 bp from *LIG3*^−/−:NC3^: sample 53 (Supplemental Methods). We performed Sanger sequencing of four contigs to validate the accuracy of calling and assembly (Supplemental Figs. S20–S23). Additionally, we examined whether contig sequences were present within cell populations before transfection with DN-*TERT* and telomere crisis. Using a single-molecule assay to detect a contig present in *LIG3*^−/−:NC3^: sample 53 (DB53_1501), we found no evidence of the contig in separate experiments, despite testing 210,000 single-cell equivalents (Supplemental Fig. S24; Supplemental Methods).

**Figure 5.**
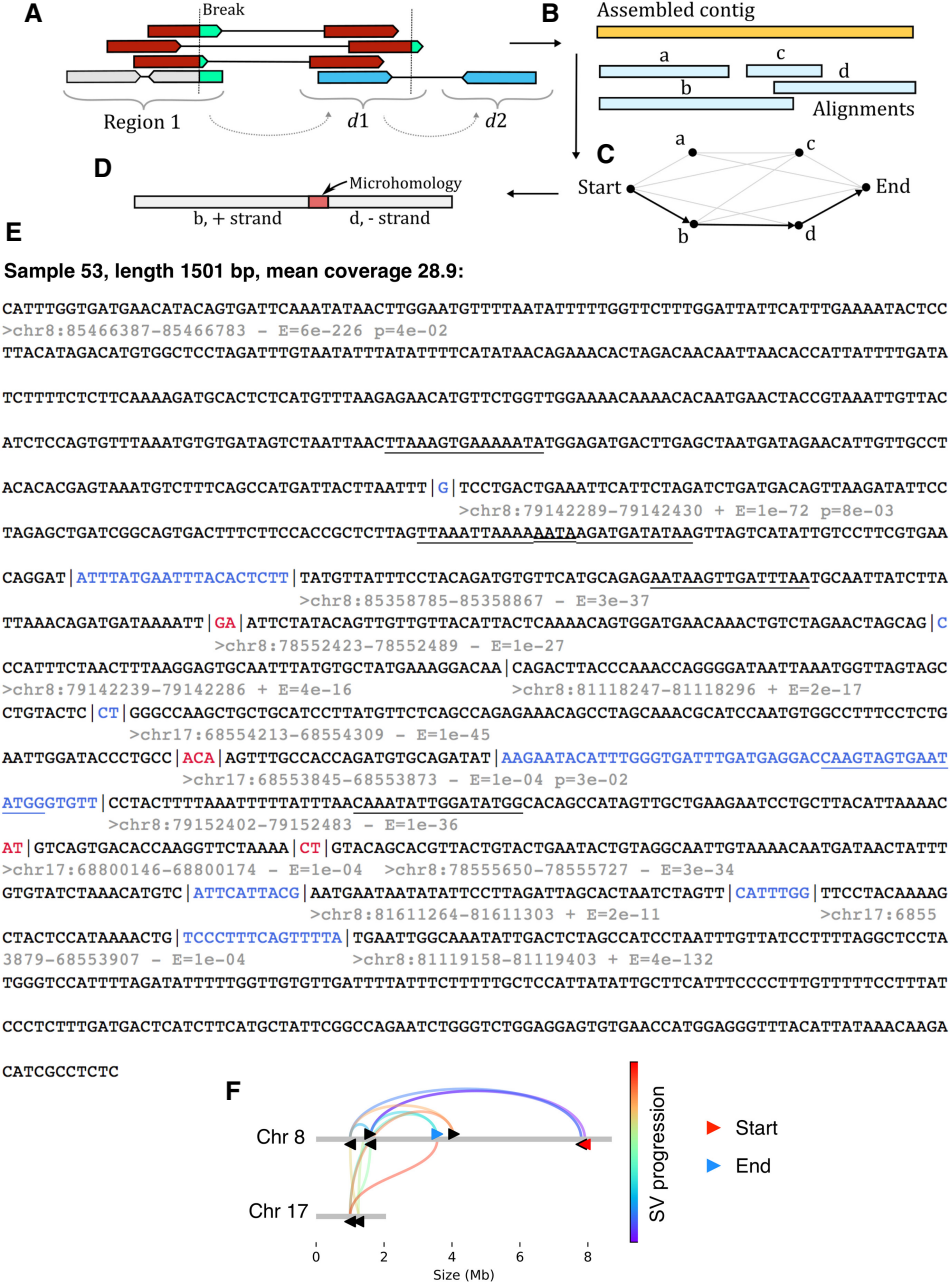
Assembly of complex SVs reveals signatures of replicative repair. (*A*) Reads were collected from breakpoint regions before recursively fetching mate pairs from distal genomic loci. (*B*) Reads were then assembled into contigs and aligned to the reference genome. An optimal set of alignments was then determined using the fnfi align algorithm https://github.com/kcleal/fnfi (*C*) before further annotation of the contigs (*D*). (*E*) An example contig from *LIG3*^−/−:NC3^:sample 53 is provided with the following annotations: Black text demarcates alignment to the reference genome, whereas red and blue indicate sections of microhomology and novel sequence insertions, respectively. *Below* the start of each alignment, the genomic interval is given along with the DNA strand and an *E*-value for the alignment. An additional annotation is added to lines for which adjacent alignments show significant levels of similarity with respect to one another, as determined by a statistical test, giving the probability “p” indicated in the annotation. Significant stretches of similarity have been underlined in adjacent segments and overlapping annotations appear as a double underlined stretch. (*F*) The progression of rearrangement over the reference genome is depicted.

Initial analysis using BLAT (Supplemental Table 2) indicated contigs often spanned multiple breakpoints with numerous candidate alignments (Kent 2002). Manually selecting a set of alignments proved time-consuming, so we developed an algorithm to optimally select a set of alignments from a candidate list based on a scoring scheme (Supplemental Code; also available at https://github.com/kcleal/fnfi). Breaksite microhomology was identified by the overlap of adjacent alignments, and breaksite insertions corresponded to gaps between alignments (Fig. 5A–D).

Many contigs were found to harbor complex events (Supplemental Fig. S25), defined as a contig with more than one SV, with one from *LIG3*^−/−:NC3^: sample 53 displaying 18 rearrangements in 2532 bp (Supplemental Fig. S26). Annotated contigs are provided in Supplemental_file_1.zip with example contigs from other backgrounds displayed in Supplemental Figures S27 and S28. A contig from *LIG3*^−/−:NC3^: Sample 53 is detailed (Fig. 5E,F), which spans 13 rearrangements >1501 bp, with nine fragments on the reverse strand and five on the forward, linking clusters on Chr 8 and Chr 17. Complex contigs often harbored very short alignments, with the example contig displaying an alignment of only 28 bp to Chr 17: 68553845 (*E*-value 1 × 10^−4^, Fig. 5E). Such short alignments are usually regarded as spurious due to the potential for multiple occurrences throughout the genome; however, a confident alignment adjacent to this fragment is found at Chr 17: 68554213 (*E*-value 1 × 10^−45^), which is only 340 bp along the reference genome, suggesting a biological process may be involved in configuring these events (Fig. 5F; Supplemental Fig. S29).

Assembly revealed huge complexity in the makeup of chained SVs and provided evidence, through the patterns of short fragment joining, which was consistent with a replicative repair process.

### Analysis of end processing at chromothriptic breaksites

Given the range of end-joining deficiencies of our cell lines, we expected to find differences in the microhomology and insertion profiles at breaksites. However, no significant differences were identified between samples that had transited crisis (*P*-value 0.19 and 0.18, respectively, Levene's test) (Supplemental Fig. S30A,B). This led us to consider whether additional repair pathways may have been used.

**Figure 6.**
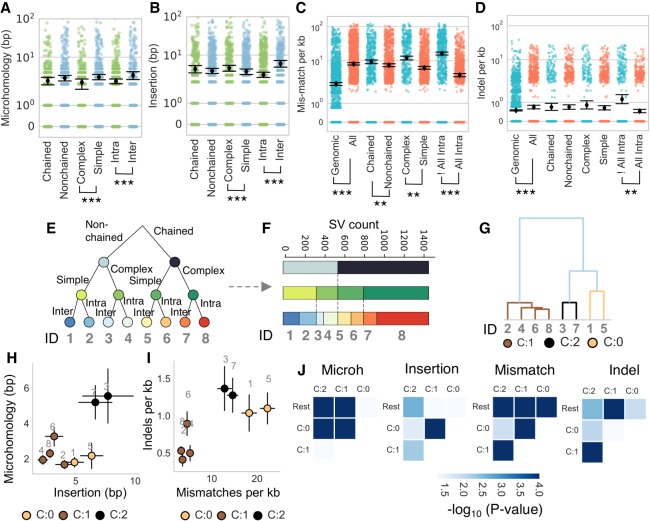
Differential end processing reveals distinct pathways used in repair of chained SVs. (*A*,*B*) Differences in microhomology and insertion profiles of SVs were quantified according to a classification of SVs as chained versus nonchained, complex versus simple, and intrachromosomal versus interchromosomal. (*C*,*D*) Upstream and downstream sequences were also analyzed for the numbers of mismatches and indels, with the All Intra versus !All Intra comparison referring to contigs with entirely intrachromosomal events versus contigs with one or more translocation. SVs were binned into eight groups (*E*) with the numbers of each division of the data presented in *F* with the category ID numbered in gray. (*G*) Agglomerative clustering was used to identify groups with similar properties labeled from C:0 to C:2. The mean values for microhomology and insertion (*H*), and mismatches and indels (*I*) of these groups are visualized as scatterplots in *H* and *I* with the color relating to the cluster in panel *G* and the gray text annotation referring to the category ID from panel *E*. (*J*) A pairwise statistical test was used to compare features and identify significant differences between clusters. (**) *P*-value <0.005; (***) *P*-value <0.0005; Mann–Whitney *U* test. Error bars, SEM.

SVs were subdivided into several nonmutually exclusive categories according to whether they were chained (chained vs. nonchained) or whether the parent contig contained multiple SVs (“complex” contigs containing multiple SVs vs. “simple” contigs with one SV) or intra- or interchromosomal events (inter vs. intra). Analyzing all backgrounds together, pairwise comparisons revealed significant differences between complex–simple and intra–inter events for microhomology and insertions (*P*-value <0.005, Mann–Whitney *U* test) (Fig. 6A,B; Supplemental Fig. S31).

No differences were identified among chained and nonchained rearrangements, which was unexpected given that chained SVs are thought to arise via a very different mechanism to nonchained events. Again, few differences were evident between genetic backgrounds or when comparing different categories of events (*P*-value >0.05, Levene's test) (Supplemental Fig. S32). Most backgrounds displayed a consistent trend in the pairwise comparison of different categories, which was reflected in lower *P*-values when all backgrounds were grouped (Supplemental Figs. S32, S33). These findings suggested that SVs were not repaired via the canonical C- or A-NHEJ pathways.

Contigs also displayed 3.85- and 1.3-fold higher mismatches and indels, respectively, to contigs assembled from random loci (*P*-value <1 × 10^−6^, Mann–Whitney *U* test) (Fig. 6C,D), supporting our previous analysis (Fig. 3G). For contigs harboring multiple SVs, it was not possible to uniquely associate every alignment with a breaksite as alignments may be sandwiched between consecutive SVs. A further category was introduced to distinguish contigs with exclusively intrachromosomal events from those with one or more interchromosomal event (All Intra vs. !All Intra) (Fig. 6C,D). A higher incidence of mismatches was identified for chained (1.3-fold vs. nonchained, *P*-value 1 × 10^−3^), complex (1.9-fold vs. simple, *P*-value 0.004) and !All Intra (4.4-fold vs. All Intra, *P*-value <1 × 10^−6^) consistent with our previous analysis (Fig. 3G), and higher indels were found for All Intra events (2.2-fold vs. !All Intra, *P*-value 0.002, Mann–Whitney *U* test). These data indicate that repair of complex SVs is intrinsically mutagenic and involves distinct repair processes between intra- or interchromosomal and between complex or simple events.

The genome-shattering model of chromothripsis posits that rearrangements occur in a single step by random ligation of fragments (Korbel and Campbell 2013; Ly and Cleveland 2017), implying that repair signatures of chained SVs should be relatively homogenous, with few differences between subcategories. Testing this possibility, SVs were binned into eight subcategories according to the divisions nonchained versus chained, simple versus complex, and inter versus intra (Fig. 6E,F; Supplemental Fig. S34). For each subcategory, microhomology and insertion lengths, as well as mismatch and indel rates, were used as features for agglomerative clustering (Fig. 6G).

Clustering identified three groups with intrachromosomal SVs forming the major category and interchromosomal SVs being subdivided according to the logical condition of being simple/complex (Fig. 6G–I; Supplemental Fig. S31E,F). These complex interchromosomal events showed high levels of mutations, insertions, and microhomology, suggestive of mutagenic repair relying on microhomology and polymerase extension.

Pairwise comparisons of cluster features revealed that all clusters showed significant differences across two or more features when compared to other clusters, suggesting clusters may reflect specific pathways used by the cell that are tailored to subsets of events (Fig. 6J; Supplemental Fig. S31G). These clustering experiments highlight differences in repair of chained and nonchained rearrangements. The heterogeneous repair of chained SVs suggests multiple processes are used and invokes a multistep repair process.

## Discussion

Recent reports have implicated telomere involvement in the formation of chromothripsis, a rearrangement pattern purported to result from the random ligation of DNA fragments following a shattering event involving one or multiple chromosomes (Li et al. 2014; Maciejowski et al. 2015; Ernst et al. 2016). Here, we documented a catastrophic genomic-restructuring pattern identified following escape from a telomere-driven crisis. The chained SVs reported here shared many features with documented chromothripsis cases, including a fluctuating CN profile, random 5′ to 3′ end-joining profiles, and signatures of APOBEC editing near SVs (Forment et al. 2012). However, several features were at odds with a shattering model of chromothripsis, including high incidence of multiple chromosomes involved, independence from C- or A-NHEJ, increased mutation rates, prevalence of CN gains, mutational signatures associated with defective HR, complex joins involving short fragments, and biases toward homo- or heterophilic cluster repair. It is possible that multiple rounds of SV chaining may have occurred in some samples, and this may have contributed to heterogeneities in the topology of repair. However, at the background level, the spacing of breakpoints into clusters appeared relatively consistent among samples, suggesting that overlapping of multiple chains may have been infrequent (Supplemental Fig. S18I).

The frequency of FBs was increased within chains, suggesting a role for telomere dysfunction in driving SV chaining, in support of recent studies (Fig. 3; Li et al. 2014; Maciejowski et al. 2015). In some samples, multiple BFB cycles may have occurred due to the identification of additional FBs or a multilevel CN profile. However, BFB cycling is unlikely to account for most chained SVs as few CN states were typically present, and SVs typically occurred in clusters along the reference genome, which is contrary to expectation for BFB cycling, in which breakages would be expected to occur more randomly between centromere and chromosome end (Bailey and Murnane 2006). FBs were often found at the edge of SV clusters, which might be expected if SV chaining was initiated at a free chromosome end. However, with increasing SV complexity, the correspondence of read-pairs as they map to the reference genome, with the actual genomic configuration, can become extremely convoluted, making interpretation of chain structure intractable. As an aid, we performed assembly of SVs into contigs that revealed an unexpected level of complexity in the configuration of chained SVs (Fig. 5).

Several features of these events were consistent with chromoplexy, including their multichromosomal nature, and occurrence of short duplicated fragments interspersed with gaps that were consistent with the “deletion bridges” reported by Baca et al. (2013). However, evidence of deletion bridges is also evident in data of Liu et al. (2011), who suggested chromothripsis be better named as chromoanasynthesis to reflect a replicative mechanism and account for the short “template insertions” (50 to 1500 bp) they identified. An emerging picture is that multiple mechanisms may result in chromothripsis or chromothripsis-like events, with the telomere crisis–induced pattern we report representing a distinct class (Ly and Cleveland 2017).

In our data, we found abundant examples of short fragments, interposed between longer sequences, in support of a replicative origin for chained SVs. For repair via NHEJ, end joining involves the interplay of multiple factors coordinating at each breaksite, and it is difficult to see how this could be achieved with such short sequences (Critchlow et al. 1997; Costantini et al. 2007; Chang et al. 2017). For example, although the Ku heterodimer (XRCC5/XRCC6), an essential factor in C-NHEJ, can bind double-stranded oligonucleotides as short as 14–18 bp, optimum binding requires ∼30 bp of dsDNA (Blier et al. 1993; Falzon et al. 1993; Walker et al. 2001) and is followed by an inward translocation requiring an additional 10–20 bp (Yoo and Dynan 1999). Although ultrashort DNA fragments could theoretically be ligated in a Ku-independent but LIG4-dependent fashion (Oh et al. 2014; Waters et al. 2014), the absence of LIG4 made little difference in our data in repairing profiles, arguing against this.

The observation of SV chaining in the absence of C-NHEJ (*LIG4*^−/−^) or A-NHEJ (*TP53*^−/−^:*LIG3*^−/−^), and in the absence of both A- and C-NHEJ (*LIG3*^−/−^:*LIG4*^−/−^), suggested repair involved an NHEJ-independent pathway. Contrary to expectations, functional NHEJ appeared to inhibit the occurrence of genome catastrophe during the telomere crisis, as SV chaining was prevalent only when NHEJ was dysfunctional. The severity of chaining was greatest when only LIG1 was present (i.e., *LIG3*^−/−^:*LIG4*^−/−^ background) or when a (super)abundance of LIG3 (*LIG3*^−/−:NC3^) was available. It is unclear if the balance of these components influenced chain propagation or if the inherent genome instability of these ligase-deficient cell lines somehow exacerbated SV chaining during crisis. The observation that *TP53*^−/−^:*LIG3*^−/−^ cells had experienced a genome catastrophe in the absence of crisis also indicates that SV chaining may arise during repair of nontelomeric double-strand breaks, albeit in the context of a deregulated repair phenotype.

Taken together, our observations are inconsistent with genome shattering followed by NHEJ-mediated repair creating complex SV chains. Instead we consider the data more consistent with a replicative process initiated directly or indirectly by dysfunctional telomeres, using microhomology and error-prone DNA polymerases, with template switching within localized genomic regions, which leads to a prevalence of CN gains. This model is consistent with the hypothesis of replication-based mechanisms driving complex rearrangements outlined by Carvalho and Lupski (Lee et al. 2007; Carvalho and Lupski 2016). Although it is possible that chained SVs may arise through various mechanisms, with genome shattering and replicative chaos two extremes, we suggest a model based on microhomology-mediated break-induced replication (MMBIR) to account for our observations (Hastings et al. 2009). Initially, telomere dysfunction triggers BFB cycling, accounting for the large terminal deletions in some samples and partially explaining raised CN profiles of others. Following breakage, free chromosome ends are processed for repair, creating a 3′ overhang. In the context of NHEJ dysfunction, failure of repair may permit the 3′ overhang to invade nearby homologous sequences and form a displacement loop (D-loop), which subsequently triggers error-prone BIR (Malkova and Ira 2013). This scenario could also arise directly from short dysfunctional telomeres, with a 3′ overhang capable of strand invasion. The BIR machinery, however, progresses toward the centromere in a retrograde fashion, leading to template switching and mutagenesis, accounting for the CN profile of FBs and fragment orientations, and chaining of duplicated segments upstream of a FB. Moreover, this mechanism is consistent with the model of Hermetz et al. (2014) to explain FB formation in patients with developmental abnormalities. Nevertheless, considerable uncertainty remains in the underlying mechanism of telomere-associated chromothripsis, and this model requires further testing in a judicial model system.

In summary, we document catastrophic genome rearrangements following escape from a telomere-driven crisis. Our data are consistent with a replicative origin for these rearrangements, plausibly carried out through repair of chromosome ends, denuded of their telomeres. Our findings have important implications for understanding cancer progression and evolution.

## Methods

### Cloning and DNA sequencing

HCT116 knockouts and derivatives were generated using recombinant adeno-associated virus-mediated gene targeting as previously described (Oh et al. 2013, 2014; Jones et al. 2014; Liddiard et al. 2016). Telomere erosion was induced following transduction by retroviral vector carrying DN-*TERT* (Hahn et al. 1999). Single-cell clones were isolated under selection and cultured crisis as described previously (Preto et al. 2004; Jones et al. 2014; Liddiard et al. 2016). Duration of crisis was calculated as the total time spent below a cell division frequency (D_f_) threshold, defined as the mean D_f_ over the experiment minus the standard deviation of D_f_. Following crisis escape, paired-end whole-genome sequencing was undertaken with 15× target depth. Reads were mapped to GRCh37 using BWA-MEM (v0.7.1) (Li and Durbin 2009). We do not expect the conclusions reached within our study to be altered by mapping to GRCh38 reference, as typically only small differences are seen in the number of variants identified between references (Guo et al. 2017).

### DN-*TERT* copy number

Reads that spanned exon boundaries of *TERT* were isolated, as these likely originated from the intronless DN-*TERT* vector. CN was inferred from read depth.

### Copy numbers

Read-depth in 10-kb nonoverlapping windows was normalized using GC and mapability information. Relative CN changes were determined by a background subtraction against the parental line and segmented using copynumber (Nilsen et al. 2012).

### Single-nucleotide variants

SNVs were called using Genome Analysis Toolkit's Haplotype Caller (GATK, v3.3) according to best practices (McKenna et al. 2010). SNVs were filtered to remove low quality variants, and SNVs unique to each sample were isolated.

### Clonality

Samples from protocols B/C were regarded as monoclonal. Remaining samples were manually assessed by two methods. VAF method: VAF distributions of unique variants at diploid regions were assessed. CN method: assessment of changes in B-allele (nonreference) distributions in the context of a CN using unique and nonunique variants.

### Structural variant calling

Deletions, inversions, duplications, and translocations were called using DELLY (v0.7.5) (Rausch et al. 2012) keeping calls with support of three or more. Unique variants were identified by crosschecking raw calls against other samples from the same background. For variants whose start and end coordinates spanned >2.5 kb, and translocations, discordant reads were collected from intervals surrounding each breaksite with padding of 1.25 kb. If a pair was identified in another sample that shared >80% reciprocal overlap with the SV in question, the call was discarded. For variants spanning <2.5 kb, padding was reduced to 1 kb and reciprocal overlap threshold was increased to 90%.

### Chained structural variants

SV chains were identified by a statistical clustering procedure. The mean number of breaks per base S_bp_ was calculated for each chromosome across samples, providing a baseline for the null model of uniform breakpoint spacing. Using this information, the spatial proximity of breakpoint pairs a, b, with separation d could be tested for significance using the binomial distribution

$$P\text{-}\text{value}=1-\mathrm{Binomial}(0,d,S_{\mathrm{bp}}),$$

representing the probability of one or more breaks within [a, b]. Chained SVs were identified by clustering on a graph: Nodes represented breakpoints, and black edges represented SVs joining DNA ends. Gray edges were added if separation distances between breakpoint pairs were deemed significant (*P*-value <*t*). Gray edges thus linked nodes, forming a connected subgraph. To qualify as a chain, a minimum number of breakpoints (b_min_) in the subgraph was required. Suitable values for *t* and b_min_ were identified by simulation using randomization.

### Fold-back inversions

Inversions mapping to “normal” reference chromosomes (Chr 1–Chr Y), <50 kb in size with three or more supporting reads, and MAPQ ≥2 were isolated. Read depth was determined in 150-bp bins across the inverted segment; *inv*_*D*_. Depth of sequencing coverage was determined over a 20-kb window preceding the inversion start coordinate *b*_*D*_, and also over a 20-kb window after the inversion end coordinate *a*_*D*_. Inversions with a mean depth ≥75 across windows were discarded. Inversions with a stepped coverage pattern were kept such that *b*_*D*_ > *inv*_*D*_ > *a*_*D*_ or *b*_*D*_ < *inv*_*D*_ < *a*_*D*_. *T*-tests were performed for each step in the CN profile: *t*-test (*b*_*D*_, *inv*_*D*_), *t*-test (*inv*_*D*_, *a*_*D*_), discarding inversions with any *P*-value ≥0.02.

### Kataegis

Kataegis clusters were identified according to Roberts et al. (2013). When assessing associations of kataegic clusters with SV chains, clusters of chained SVs were defined as intervals over the reference genome. Overlap between kataegic and SV clusters were assessed using Fisher's exact test (Quinlan and Hall 2010).

### Mismatches and mutational signatures near SVs

SV-associated SNVs were identified in 20-kb intervals centered over each breakpoint. Mean mutation rates over these intervals were determined, whereas background was taken as the mean mutation rate across all other samples, using identical intervals. Signatures were deconstructed using the deconstructSigs package (Rosenthal et al. 2016).

### Network analysis of chained SVs

Network analysis and visualization were performed with NetworkX (v1.9) (Hagberg et al. 2008). Block models were used to analyze SV chains, partitioning and labeling nodes according to the identified clusters within a chain. Modularity was determined using the partition label as the attribute parameter. Randomized graphs were generated by randomizing SV edges (black edges), whereas partition labels were fixed. To estimate the probability of observing a given number of edges between clusters, Monte Carlo simulations were run, producing 1000 randomized graphs per SV chain and generating a distribution for the expected number of edges between clusters for randomized data. Using the cumulative density function, the probability of observing at least *n* number of edges between adjacent clusters was calculated:

P(X≥n)=1−CDF(s),

where s is the proportion of simulations with <*n* edges between clusters.

### Chromothripsis database analysis

Data were downloaded from the chromothripsis database (Yang et al. 2016). Some database samples contained few breakpoints (CTDB0352 had six), whereas a small number had very high numbers (CTDB0433 had more than 7000). Such samples were considered too sparse or dense to undergo reliable separation into clusters using our methodology; therefore, samples with more than 40 and less than 1500 breakpoints were analyzed.

### Nearest-neighbor analysis

Nearest-neighbor distances were determined for intracluster breakpoints. For points x and y, which are both nearest neighbors to each other, the distance d_xy_ was included once to avoid violating assumed independence of nearest-neighbor distances.

### Structural variant assembly

Reads from an interval ±650 bp around each breakpoint were collected, keeping discordants or reads with >20 bp of soft-clip. A recursive operation was then performed to discover additional reads. For any read identified in a primary region whose mate was mapped to an unvisited genomic interval, this interval was also searched. This operation was performed up to two times, visiting up to two secondary regions. Reads were assembled using SPAdes (Bankevich et al. 2012), and low-quality contigs were discarded. Contigs were validated by PCR using primers specific to each contig, followed by Sanger sequencing of amplicons.

### Contig mapping

Contigs were mapped to GRCh37 using LAST (Kielbasa et al. 2011), generating a list of candidate alignments. An optimal set of alignments then was chosen by solving an optimal-path problem parameterized by a scoring scheme. Query alignments were represented as nodes on a directed acyclic graph *G* = (*V*, *E*). Edges represented order of alignments and imply a sequence of SVs. Edges were associated with a weight relating to the properties of an SV transition and the alignment score of the downstream node. The optimal set of alignments is found as the highest scoring path in *G* using a longest path algorithm *O*(*V*^2^) (Sedgewick and Wayne 2016). For an overview of the edge costs and algorithm implementation, see Supplemental Code (also available online at https://github.com/kcleal/fnfi).

### Sequence similarity between adjacent alignments

Sequence similarity between successive alignments was identified by performing pairwise alignment. The statistical significance of alignment scores was assessed by random sampling using alignment of random sequences.

### Verification of post-crisis contig identity

We used single-molecule PCR using oligonucleotide primers designed from the DB53_1501 contig sequence. Amplification products were detected by Southern hybridization.

### Mutation rates in contigs

Mismatch and indel rates in contigs were calculated by taking the mean mutation rate of alignments upstream of and downstream from the join. For comparison, de novo assembly of random genomic loci was performed by selecting 100 random 1-kb intervals per sample from “normal” reference chromosomes (Chr 1–Chr Y), making sure intervals did not overlap gaps in the reference. Contigs were assembled and analyzed as described. Mutations rates were determined from the primary alignment of the contig.

### Clustering of SV signatures

SVs were divided into eight categories, subdividing as chained versus nonchained, followed by complex (referring to more than one SV in the contig) versus simple, and intrachromosomal versus interchromosomal. Agglomerative clustering (linkage=“average”) was performed (Pedregosa et al. 2011), using mean insertion, microhomology, mismatch, and indel rates as features.

### Programming, statistics, and visualization

Programming and analysis was performed using Python (2.7) and SciPy ecosystem (http://www.scipy.org/). Statistical tests from Scipy.stats were used (*t*-test for independent samples with identical variance, Fisher's exact test, χ^2^ test, Mann–Whitney *U* test, Fligner–Kileen test, and Levene's test). Plotting was performed using Matplotlib and seaborn (https://seaborn.pydata.org; Hunter 2007). Circos (v0.69-2) was used (Krzywinski et al. 2009).

## Data access

WGS data from this study have been submitted to the NCBI BioProject database (https://www.ncbi.nlm.nih.gov/bioproject) under accession number PRJNA417592. Sanger sequences from this study have been submitted to GenBank (https://www.ncbi.nlm.nih.gov/genbank) under accession numbers MK404064–MK404085.

## Supplementary Material

Supplemental Material
