# Supplementary material for: Chromothripsis during telomere crisis is independent of NHEJ, and consistent with a replicative origin
Source: Genome Res. 2019 May;29(5):737–49. doi: 10.1101/gr.240705.118 (PMC6499312; doi:10.1101/gr.240705.118)
Supplement: Supplemental Material [file supp_29_5_737__index.html]

Chromothripsis during telomere crisis is independent of NHEJ, and consistent with a replicative origin — Chromothripsis during telomere crisis is independent of NHEJ, and consistent with a replicative origin — Supplemental Material 

# Chromothripsis during telomere crisis is independent of NHEJ, and consistent with a replicative origin

## Supplemental Material

- Supplemental\_Figures.docx
- Supplemental\_Tables.docx
- Supplemental\_file\_1.zip
- Supplemental\_Material.docx
