# Supplementary material for: Chromothripsis during telomere crisis is independent of NHEJ, and consistent with a replicative origin
Source: Genome Res. 2019 May;29(5):737–49. doi: 10.1101/gr.240705.118 (PMC6499312; doi:10.1101/gr.240705.118)
Supplement: Supplemental Material [file supp_gr.240705.118_Supplemental_Figures.docx]

**Chromothripsis during telomere crisis is independent of NHEJ and consistent with a replicative origin**

Kez Cleal^1^, Rhiannon E. Jones^1^, Julia W. Grimstead^1^, Eric A. Hendrickson^2¶^, Duncan M. Baird^1¶*^

^1^ Division of Cancer and Genetics, School of Medicine, Cardiff University, Heath Park, Cardiff, CF14 4XN, UK.

^2^Department of Biochemistry, Molecular Biology, and Biophysics, University of Minnesota Medical School, Minneapolis, MN 55455, USA

^¶^joint senior authors

*Correspondence email: bairddm@cardiff.ac.uk

# Supplementary Figures

Table of Contents

[Supplemental Figure S1. Depiction of the cloning protocols utilized. 3](#_Toc534885977)

[Supplemental Figure S2. Genetic background influences time spent in crisis. 4](#_Toc534885978)

[Supplemental Figure S3. Telomere length profiles during transit through crisis. 5](#_Toc534885979)

[Supplemental Figure S4. Analysis of clonality of escaped WT cultures. 6](#_Toc534885980)

[Supplemental Figure S5. Analysis of clonality of escaped *LIG4*^-/-^ cultures. 8](#_Toc534885981)

[Supplemental Figure S6. Analysis of clonality of escaped *LIG3*^-/-^:*LIG4*^-/-^ cultures. 9](#_Toc534885982)

[Supplemental Figure S7. Analysis of clonality of escaped *LIG3*^-/-:NC3^ cultures. 10](#_Toc534885983)

[Supplemental Figure S8. Analysis of clonality of escaped *TP53*^-/-^:*LIG3*^-/-^ cultures. 11](#_Toc534885984)

[Supplemental Figure S9. Copy number profiles of parental lines. 12](#_Toc534885985)

[Supplemental Figure S10. Chromosome 8q amplification in *LIG4*^-/-^ samples present at sub-clonal levels in the parental line. 13](#_Toc534885986)

[Supplemental Figure S11. Copy number events are not enriched at specific chromosome arms. 14](#_Toc534885987)

[Supplemental Figure S12. The influence of genetic background on the numbers of SV identified. 15](#_Toc534885988)

[Supplemental Figure S13. Representative complex genome rearrangements. 16](#_Toc534885989)

[Supplemental Figure S14. Validation of statistical model for identifying chained breakpoints. 17](#_Toc534885990)

[Supplemental Figure S15. Chained SVs show no preference of end-join type. 18](#_Toc534885991)

[Supplemental Figure S16. Association of kataegic sites with chained rearrangements. 19](#_Toc534885992)

[Supplemental Figure S17. Mutational signatures at SV loci. 20](#_Toc534885993)

[Supplemental 21](#_Toc534885994)

[Figure S18. Network analysis of chained SVs identifies biases for intra-cluster repair or repair between specific clusters. 21](#_Toc534885995)

[Supplemental Figure S19. Nonrandom network topology in cancer chromothripsis. 23](#_Toc534885996)

[Supplemental Figure S20. Contig validation by Sanger sequencing. 25](#_Toc534885997)

[Supplemental Figure S21. Contig validation by Sanger sequencing. 26](#_Toc534885998)

[Supplemental Figure S22. Contig validation by Sanger sequencing. 27](#_Toc534885999)

[Supplemental Figure S23. Contig validation by Sanger sequencing. 28](#_Toc534886000)

[Supplemental Figure S24. Verification of the post-crisis identity of contig DB53_1501. 29](#_Toc534886001)

[Supplemental Figure S25. Structural variants identified from contig assembly. 30](#_Toc534886002)

[Supplemental Figure S26. An assembled contig harbouring 18 rearrangements. 31](#_Toc534886003)

[Supplemental Figure S27. Example contigs derived from WT and *LIG4*^-/-^. 32](#_Toc534886004)

[Supplemental Figure S28. Example contigs derived from *LIG3*^-/-^:*LIG4*^-/-^ and *TP53*^-/-^:*LIG3*^-/-^. 33](#_Toc534886005)

[Supplemental Figure S29. Raw read supporting a complex join involving short fragments. 34](#_Toc534886006)

[Supplemental Figure S30. Few differences in microhomology and insertion usage for SV categories despite radically different NHEJ function. 35](#_Toc534886007)

[Supplemental Figure S31. Analysis of fractions and of break sites with microhomology or insertions, and absolute sequence lengths. 36](#_Toc534886008)

[Supplemental Figure S32. Analysis of microhomology and insertion profiles of SV categories. 37](#_Toc534886009)

[Supplemental Figure S33. Analysis of fractions of break sites with microhomology or insertions for SV categories. 38](#_Toc534886010)

[Supplemental Figure S34. Division of SVs into categories based on genetic background. 39](#_Toc534886011)

Supplemental Figure S1. Depiction of the cloning protocols utilized. DN-*TERT* indicates the expression of a dominant-negative human telomerase construct. Cloning protocol A refers to single-cell cloning prior to crisis and protocol B indicates single-cell cloning post crisis, whilst protocol C involves single-cell cloning both prior to and post-crisis. Large circles represent culture dishes and red dots represent single-cell clones with the clone to be isolated indicated with a dotted line. Samples were analyzed by next generation sequencing (NGS).

Supplemental Figure S2. Genetic background influences time spent in crisis. The time spent in crisis was quantified by analyzing the frequency of cell division between consecutive passages of the culture. Growth curves of *TP53*^-/-^:*LIG3*^-/-^ clones that transited crisis are displayed in A, with population doublings (PD) shown on the x-axis. Examples of growth curves from the indicated genetic backgrounds are provided in the top panels (B to F). The crisis period (indicated by the red line) was defined as the time the culture spent below a growth rate threshold, defined as the mean growth rate minus the standard deviation of the growth rate and indicated as a blue-dashed line in the bottom panels (B to F). A summary of the distributions of times spent in crisis by the clones is provided in panel G, with the LIG3-complemented line displaying an increase in time spent in crisis relative to the WT (*p-value < 0.05 Mann-Whitney *U* test). In a pooled analysis, a weak correlation was also observed between the time spent in crisis and the number of structural variants subsequently identified (panel H). Copy numbers of the DN-*TERT* vector were determined by assessing the mapping depth of exon-spanning read-pairs at the *TERT* locus, which likely originated from integrated vector cDNA (I)

Supplemental Figure S3. Telomere length profiles during transit through crisis. Mean telomere length of samples was quantified before crisis (green), during crisis (red), or after escape from crisis (blue) as documented previously (Jones et al. 2014). Sample numbers are displayed on the x-axis. Sample 89 was sampled twice prior to crisis, whereas Samples 91 – 94 were not quantified prior to crisis. For samples prepared by cloning protocol B (Table S1, Figure S1), it was not possible to obtain length profiles prior to, or during crisis, so these samples were not analyzed. For length profiles of other samples, please refer to (Jones et al. 2014).

Supplemental Figure S4. Analysis of clonality of escaped WT cultures. Mono- or polyclonality was inferred by assessing B-allele frequency in the context of a copy number (CN) change (A), or by assessing the unique VAF frequency at diploid sites within the genome (B). Using principles outlined below, we manually inferred the clonality of cultures (C). For a monoclonal culture with a gain of CN +1 to a triploid state, the B-allele frequency profile would show two peaks at frequency 0.33 and 0.66 (A). Likewise, a monoclonal culture with a -1 CN loss to a haploid state would show a single B-allele peak at frequency 1.0 (A). The presence of an additional clone, with perhaps additional karyotypic alterations, will tend to distort the positions of these idealized peaks in both the x and y planes. If a culture is polyclonal and each clone is sufficiently diverse, the unique VAF profile from diploid regions of the genome may be utilized to provide evidence of polyclonality with a left-shift in the VAF peak suggestive of the presence of additional subpopulations that tend to dilute the idealized VAF profile. However, if clones are not sufficiently diverse or many subclones are present, few SNPs unique to each subclone may be identified making inference challenging. As larger numbers of variants were analyzed, the first method took precedence. All samples that had undergone cloning protocols B or C (cloning post-crisis), were deemed to be monoclonal in nature (please refer to Table 1, Figure S1 for a list of samples and summary of cloning protocols). Highlighting specific examples, sample 37 was deemed to be polyclonal as many unique SNVs were identified (B) giving a robust VAF profile that was left-shifted. Samples 38 and 39 were deemed to be of unknown clonality as too few unique SNVs were identified for inference based on the VAF peak alone, and CN changes were deemed to be inconclusive (A). Sample 40 was deemed to be monoclonal as duplicated segments (CN + 1) showed the expected peaks in allele frequency (A); the VAF peak in the sample was deemed inconclusive due to the very small number of unique variants identified (B).

Supplemental Figure S5. Analysis of clonality of escaped LIG4-/- cultures. The clonality of the cultures was assessed by analyzing B-allele frequencies in the context of CN changes (A), or by analyzing the VAF profile at diploid sites within the genome (B). Mono- or polyclonality was manually inferred by comparing these profiles against theoretical distributions of mono or polyclonal cultures (C). The analyses were performed as described in the legend to Figure S4.

Supplemental Figure S6. Analysis of clonality of escaped LIG3-/-:LIG4-/- cultures. The clonality of cultures was assessed by analyzing B-allele frequencies in the context of CN changes (A), or by analyzing the VAF profile at diploid sites within the genome (B). Mono- or polyclonality was manually inferred by comparing these profiles against theoretical distributions of mono or polyclonal cultures (C)**.** The analyses were performed as described in the legend to Figure S4.

Supplemental Figure S7. Analysis of clonality of escaped *LIG3*^-/-:NC3^ cultures. The clonality of cultures was assessed by analyzing B-allele frequencies in the context of CN changes (A), or by analyzing the VAF profile at diploid sites within the genome (B). Mono- or polyclonality was manually inferred by comparing these profiles against theoretical distributions of mono or polyclonal cultures (C). The analyses were performed as described in the legend to Figure S4.

Supplemental Figure S8. Analysis of clonality of escaped *TP53*^-/-^:*LIG3*^-/-^ cultures. The clonality of cultures was assessed by analyzing B-allele frequencies in the context of CN changes (A), or by analyzing the VAF profile at diploid sites within the genome (B). Mono- or polyclonality was manually inferred by comparing these profiles against theoretical distributions of mono or polyclonal cultures (C). The analyses were performed as described in the legend to Figure S4.

Supplemental Figure S9. Copy number profiles of parental lines. The read coverage was determined using 10 kb non-overlapping windows and was normalized against the GC content and mapability of the window. For the *TP53*^-/-^:*LIG3*^-/-^ cell line (bottom pane), blue triangles indicate chromosomes with abnormalities consistent with a whole genome rearrangement catastrophe.

Supplemental Figure S10. Chromosome 8q amplification in *LIG4*^-/-^ samples present at sub-clonal levels in the parental line. The read depth profiles of LIG4^-/-^ parental (A) and five post-crisis clones (B to F) identify a common chr8q amplification that is not readily detected in the parental line (A). Inspection of the copy number breakpoints indicated by *x* and *y* for the parental line (G and H) and sample 45 (I and J) demonstrated that the CNV is indeed present in the parental line but at a sub-clonal level. Read-pairs that span the tandem duplication have been colored in crimson using IGV (G to J). Counting the average number of reads supporting the break site (K) indicated a CNV frequency of 0.11 in the parental cell line suggesting a prevalence of around 25% assuming a triploid state as the normal copy number for this region.

Supplemental Figure S11. Copy number events are not enriched at specific chromosome arms. The mean number of CN events was calculated for each chromosome arm across all our samples and plotted as a bar chart. The expected number of CN events per chromosome arm was calculated based on a uniform probability of occurrence over the genome and shown using red dots. The number of CN events per chromosome arm was modeled as a Poisson random variable. Probability values were determined using a cumulative Poisson distribution and a Bonferroni (alpha = 0.05) correction, representing the probability of obtaining a value greater than or equal to the observed data.

Supplemental Figure S12. The influence of genetic background on the numbers of SV identified. SVs were grouped by genetic background to show the numbers of deletions, inversions, duplications and translocations (DEL, INV, DUP, TRA, respectively).

Supplemental Figure S13. Representative complex genome rearrangements. A single example of a complex rearrangement for *LIG4*^-/-^, *LIG3*^-/-^:*LIG4*^-/-^ and *TP53*^-/-^:*LIG3*^-/-^ cells is shown (A to C, respectively).

Supplemental Figure S14. Validation of statistical model for identifying chained breakpoints. A statistical clustering procedure was developed to identify chain-like rearrangements in our data (A). An example rearrangement involving 8 free ends is depicted in A1, with four ends residing on chr*_i_* labelled A to D, and four ends on chr*_j_* labelled E to H. These ends were represented as nodes on a graph in A2, with black edges representing a SV linking two free ends. Grey edges are then added to the graph if the separation of neighboring breakpoints along the reference genome was deemed to be statistically significant based on a null model of uniform breakpoint density. In this example, breaks on chr*_i_* and chr*_j_* are deemed significantly close to one another warranting the addition of grey edges between nearby breaks (A2). Once all grey edges have been added, removal of the black edges from the graph leaves only the remaining grey edges, which breaks the graph into a series of connected subgraphs. These subgraphs represent clusters of breaks along the reference genome. These clusters may then be collapsed using a block model so nodes now represent entire clusters whilst edge weights represent the number of SV edges linking adjacent clusters (A3). This clustering methodology requires two parameters; the p-value threshold for classifying separation distances as significant; and the number of breaks (free ends) required to classify linked SVs as a chain. The choice of these parameters was informed by performing simulations that involved the shuffling of breakpoint positions along the parent chromosome of each sample (B to E). Each point in B to E represents the false positive rate for a single sample using the stated p-value threshold and break number threshold. The black line represents the average false positive rate over all samples (B to E). Based on these simulations a p-value of 0.04 was chosen with a break number threshold of 8, giving a mean false positive rate of < 1%.

Supplemental Figure S15. Chained SVs show no preference of end-join type. The numbers of non-chained (A) or chained (B) SVs for each background were quantified in terms of their end-to-end join types with 3to5 representing the "deletion" type, 5to3 representing the "duplication" type, and both 3to3 and 5to5 representing "inversion" type joins. P-values < 0.004, Levene’s test.

Supplemental Figure S16. Association of kataegic sites with chained rearrangements. A total of 26 kataegic sites were identified with 16 sites falling within 20 kb of a break site (A). Of these 16 sites, 7 were found to be associated with chained rearrangements (B). Treating entire clusters of chained rearrangements as intervals over the reference genome, the number of overlaps between kataegic sites and chained clusters was determined (C). For informative samples with overlaps, a p-value appears above the bar, calculated by Fisher’s exact two-sided test (C). For each sample with chained (+) or non-chained (-) SVs, the frequency of kataegic sites was compared (D), although no statistical difference was identified (p-value = 0.53, Fisher’s exact).

Supplemental Figure S17. Mutational signatures at SV loci. SNVs were classed as being associated with chained SVs (A), non-chained SVs (B), or else being categorized as ‘Other’ (C). Mutational signatures were delineated using the deconstructSigs package and the observed (i) and reconstructed (ii) mutational signatures were plotted along with the error profile (iii) (Rosenthal et al. 2016).

**Supplemental** **Figure S18. Network analysis of chained SVs identifies biases for intra-cluster repair or repair between specific clusters.**  Two large networks of chained SVs from LIG3^-/-^:LIG4^-/-^; sample 51 and LIG3^-/-:NC3^; sample 57 are depicted in A to D and E to H, respectively. Breakpoints were initially visualized as individual nodes with black edges representing a SV (A, E). Each node is labelled by chromosome number, or for unplaced contigs in the human genome a ‘Un_gl’ prefix is added (A, E). For breakpoints that were in spatial proximity on the reference genome, a grey edge was added between nodes (A, E). This allowed clusters of breakpoints on the reference genome to be identified by removal of black edges. Clusters were then collapsed using a block model so each node in B and F represents a cluster of breakpoints with the node size indicating the number of intra-cluster SV (black) edges, and the edge weight (thickness) representing the number of SV edges between adjacent cluster nodes. Utilizing the same block model labels, which represent the clusters of breakpoints on the reference genome, breakpoint joining was then randomized to simulate a random repair process with the randomized graph representation shown on the right-hand side of the panel in B to D and F to H. Graphs were visualized as adjacency matrices (C, G) with each row and column representing a cluster from the block model. The number of breakpoints in the cluster is provided as an annotation on the x and y axes. The color of each matrix element represents the adjacency between two clusters, that is, the number of SV edges between two clusters (C, D, G and H). Therefore, the diagonal of these matrices represents intra-cluster linkages, whilst other elements represent inter-cluster linkages. Using simulation, the breakpoint adjacency of the random graph was estimated allowing the probability of the observed numbers of linkages between clusters to be estimated (D, H). For clustered SVs identified within this study, or listed in the chromothripsis database, the nearest neighboring break was plotted as a cumulative distribution, and annotated with an exponential line of best fit (I). Visualizing the cumulative distribution on a log-x plot in this way, illustrates that chained breakpoints tend to be very closely spaced, with for example, around 40 % of breakpoints found around 1kb from an independent chained breakpoint.

Supplemental Figure S19. Nonrandom network topology in cancer chromothripsis. Chromothripsis rearrangement networks were downloaded from the chromothripsis database and analyzed for heterophilic mixing patterns (Yang et al. 2016). Cancer samples included small cell lung (A) (Stephens et al. 2011), colorectal (B) (Stephens et al. 2011), osteosarcoma (C) (Stephens et al. 2011), pancreatic (D) (Waddell et al. 2015), ependymoma (E) (Parker et al. 2014), and esophageal (F) (Nones et al. 2014). Chain-graphs derived from the data are depicted in the left panel (i) whilst an equivalent randomized graph is shown in the middle panel (ii). Matrices to the right (iii) display the probability of observing the given linkage pattern between specific clusters within the chain when compared to randomly joined graphs. Each row and column correspond to an individual cluster, with the number shown on the x and y-axis giving the number of breaks within the cluster (iii).

Supplemental Figure S20. Contig validation by Sanger sequencing. Sanger traces from contig “sample 53, length 1501” were assembled using cap3 (Huang and Madan 1999) and aligned to contigs generated from whole-genome sequencing data by the SPAdes assembler (Bankevich et al. 2012). Rearrangements have been highlighted using grey blocks to depict alignment to the reference genome, whilst red and blue blocks indicate microhomology and insertions, respectively. Vertical bars indicate blunt end joins.

Supplemental Figure S21. Contig validation by Sanger sequencing. Sanger traces from contig “sample 53, length 2061” were assembled using cap3 (Huang and Madan 1999) and aligned to contigs generated from whole-genome sequencing data by the SPAdes assembler (Bankevich et al. 2012). Rearrangements have been highlighted using grey blocks to depict alignment to the reference genome, whilst red and blue blocks indicate microhomology and insertions, respectively.

Supplemental Figure S22. Contig validation by Sanger sequencing. Sanger traces from contig “sample 51, length 1041” were assembled using cap3 (Huang and Madan 1999) and aligned to contigs generated from whole-genome sequencing data by the SPAdes assembler (Bankevich et al. 2012). Rearrangements have been highlighted using grey blocks to depict alignment to the reference genome, whilst red and blue blocks indicate microhomology and insertions, respectively. Vertical bars indicate blunt end joins.

Supplemental Figure S23. Contig validation by Sanger sequencing. Sanger traces from contig “sample 51, length 681” were assembled using cap3 (Huang and Madan 1999) and aligned to contigs generated from whole-genome sequencing data by the SPAdes assembler (Bankevich et al. 2012). Rearrangements have been highlighted using grey blocks to depict alignment to the reference genome, whilst red and blue blocks indicate microhomology and insertions, respectively.

Supplemental Figure S24. Verification of the post-crisis identity of contig DB53_1501. Partial sequence of contig DB53_1501 (A). PCR oligonucleotide primer sequences are highlighted in yellow. The primer pair DB53_1501F and DB53_1501R1 generated a 704 bp PCR amplicon spanning 8 breakpoints. (B) Single-molecule detection of the PCR amplicon generated with DB53_1501F and DB53_1501R1 detected by Southern hybridization using a hybridization probe derived from the same PCR primers. The parental cells (*LIG3*^-/-:NC3^) were analyzed in isolation (50ng/reaction and 10ng/reactions) or spiked with dilutions of *LIG3*^-/-:NC3^: sample 53 (+samp53) post crisis cells as indicated above the panel (B).

Supplemental Figure S25. Structural variants identified from contig assembly. Contigs were generated from SVs identified during our initial analysis. Remapping these contigs identified a new set of SVs with numbers of intra- and inter-chromosomal events (A), chained and non-chained SVs (B), as well as complex and simple SVs (C).

Supplemental Figure S26. An assembled contig harboring 18 rearrangements. Different alignments to the reference genome are shown in black text and demarcated using a vertical bar. Microhomology at break sites appears in neighboring alignments and is shown in red whereas blue characters indicate break site insertions that do not align to the reference genome. The genomic coordinates of alignments are annotated underneath in grey text along with the DNA strand, and an E-value for the alignment. Underlined text indicates significant stretches of similarity between adjacent alignments. Stretches of similarity may also overlap between adjacent alignments, which is indicated using a double underline.

Supplemental Figure S27. Example contigs derived from WT and *LIG4*^-/-^. A contig with 3 SVs from the WT (A) background and a contig with 6 SVs from the *LIG4*^-/-^ background (B) are shown. Microhomology at break sites is shown in red whereas blue characters indicate break site insertions. Alignments have been annotated in grey text with the genomic coordinates, strand, and E-value for the alignment. Underlined text indicated significant stretched of similarity between adjacent alignments.

Supplemental Figure S28. Example contigs derived from *LIG3*^-/-^:*LIG4*^-/-^ and *TP53*^-/-^:*LIG3*^-/-^. A contig with 3 SVs from *LIG3*^-/-^:*LIG4*^-/-^ (A) cells and a contig with 6 SVs from the *TP53*^-/-^:*LIG3*^-/-^background (B) are shown. Microhomology at break sites is shown in red whereas blue characters indicate break site insertions. Alignments have been annotated in grey text with the genomic coordinates, strand, and E-value for the alignment. Underlined text indicated significant stretched of similarity between adjacent alignments.

**** Supplemental Figure S29. Raw read supporting a complex join involving short fragments. Discordant read pairs have been colored purple indicating their mate pair maps to chromosome 8. Following contig assembly and analysis of alignments, these purple reads were identified as being involved in a complex joining event with black vertical lines indicating the positions of breakpoints along these reads. From right to left, a short fragment from only 340 bp upstream on the reference genome is joined with the right-hand fragment. This short fragment is followed by a longer insertion before an inter-chromosomal join with chr8 on the left-hand side of the read.

Supplemental Figure S30. Few differences in microhomology and insertion usage for SV categories despite radically different NHEJ function. Microhomology (A to G) and insertions (H to N) found at join sites were quantified. Subsets of the data referring to the individual categories outlined in Figure 6E are shown in the panels below A and H.

Supplemental Figure S31. Analysis of fractions and of break sites with microhomology or insertions, and absolute sequence lengths. Differences in the fractions of break sites with either microhomology (A) or insertions (B) were analyzed for the categories of chained versus non-chained, complex versus simple, intra-chromosomal versus inter-chromosomal. The absolute lengths of microhomology and insertions found at break sites were analyzed in (C, D), referring to only those sites with > 0 bp of microhomology or insertion. A clustering procedure referred to in Figure 6G using 8 categorical groups identified three clusters labelled C:0 to C:2. Here, these same cluster labels are utilized with the color of each point (E, F) corresponding to the cluster label in Figure 6G and the grey text annotation referring to the categorical ID in Figure 6E. However, calculation of the mean microhomology and insertion of each category (each scatter point in E) was limited to only sites with > 0 bp of microhomology or insertion, so only the mean absolute lengths of sequence marks are assessed (E). Similarly, for the indicated categories calculation of the mean mismatch and indel rates of nearby sequences was limited to only alignments with > 0 indel or mis-matches (F). Pairwise statistical tests were performed over each cluster feature, comparing against individual clusters and the remaining data. In A, B, G, p-values *** < .0005, chi-squared. In C, D, p-values *** < .0005, Mann-Whitney *U*. Error bars represent SEM.

Supplemental Figure S32. Analysis of microhomology and insertion profiles of SV categories. The overall microhomology and insertion profiles of non-chained and chained (A, B), simple and complex (C, D) and inter and intra-chromosomal (E, F) events are displayed. P-values * < .05, ** < .005, *** < .0005, Mann-Whitney *U*. Error bars represent SEM.

Supplemental Figure S33. Analysis of fractions of break sites with microhomology or insertions for SV categories. The fractions of break sites with microhomology or insertions for the categories non-chained and chained (A, B), simple and complex (C, D) and inter and intra-chromosomal (E, F) events are displayed. P-values * < .05, ** < .005, *** < .0005, Mann-Whitney *U*. Error bars represent SEM.

Supplemental Figure S34. Division of SVs into categories based on genetic background. SVs were divided into categories based on the decision tree depicted in (A). The breakdown of SVs into these categories for each genetic background is displayed (B to H).
