# Supplementary material for: Chromothripsis during telomere crisis is independent of NHEJ, and consistent with a replicative origin
Source: Genome Res. 2019 May;29(5):737–49. doi: 10.1101/gr.240705.118 (PMC6499312; doi:10.1101/gr.240705.118)
Supplement: Supplemental Material [file supp_gr.240705.118_Supplemental_Tables.docx]

**Chromothripsis during telomere crisis is independent of NHEJ and consistent with a replicative origin**

Kez Cleal^1^, Rhiannon E. Jones^1^, Julia W. Grimstead^1^, Eric A. Hendrickson^2¶^, Duncan M. Baird^1¶*^

^1^ Division of Cancer and Genetics, School of Medicine, Cardiff University, Heath Park, Cardiff, CF14 4XN, UK.

^2^Department of Biochemistry, Molecular Biology, and Biophysics, University of Minnesota Medical School, Minneapolis, MN 55455, USA

^¶^joint senior authors

*Correspondence email: bairddm@cardiff.ac.uk

# Supplementary Tables

Table of Contents

[Supplemental Table 1. The list of samples that underwent paired-end sequencing. 1](#_Toc534798374)

[Supplemental Table 2. Example output produced by BLAT. 3](#_Toc534798375)

| **Cell line** | **Abbreviation** | **Identifier** | **N** | **Cloning protocol** | **Crisis** |
| --- | --- | --- | --- | --- | --- |
| WT parental | WT(p) | 67 | 1 | - | - |
| *LIG4*^-/-^ parental | *LIG4*(p) | 68 | 1 | - | - |
| *LIG3*^-/-:mL3^ parental | *LIG3*(p) | 69 | 1 | - | - |
| *LIG3*^-/-^: *LIG4*^-/-^ parental | *LIG3*^-/-^:*LIG4*^-/-^(p) | 70 | 1 | - | - |
| *LIG3*^-/-:mL3:NC3^ parental | *LIG3*^-/-:NC3^(p) | 71 | 1 | - | - |
| *TP53*^-/-^: *LIG3*^-/-:mL3^ parental | *TP53*^-/-^:*LIG3*^-/-^(p) | 73 | 1 | - | - |
| WT empty vector | WT-puro | 105-109 | 5 | A |  |
| WT | WT | 37-41, 95-104 | 15 | 37-41 = A, 95-104 = C | + |
| *LIG4*^-/-^ | *LIG4*^-/-^ | 45-49, 80-84 | 10 | A | + |
| *LIG3*^-/-^: *LIG4*^-/-^ | *LIG3*^-/-^:*LIG4*^-/-^ | 50-52,110-116 | 10 | 50-52 = A, 110-116 = B | + |
| *LIG3*^-/-:mL3:NC3^ | *LIG3*^-/-:NC3^ | 53-57, 75-79 | 10 | A | + |
| *TP53*^-/-^: *LIG3*^-/-:mL3^ | *TP53*^-/-^:*LIG3*^-/-^ | 85-94 | 10 | A | + |

Supplemental Table 1. The list of samples that underwent paired-end sequencing. The relevant genotypes of the cell lines used in this study are indicated. The identifier is an arbitrary number designation that was used for the NGS sequencing. N indicates the number of samples that were utilized for NGS sequencing. Cloning protocol A refers to cloning prior to crisis, protocol B indicates cloning post crisis, whilst protocol C involves cloning both prior to and post-crisis (see Figure S1).

**
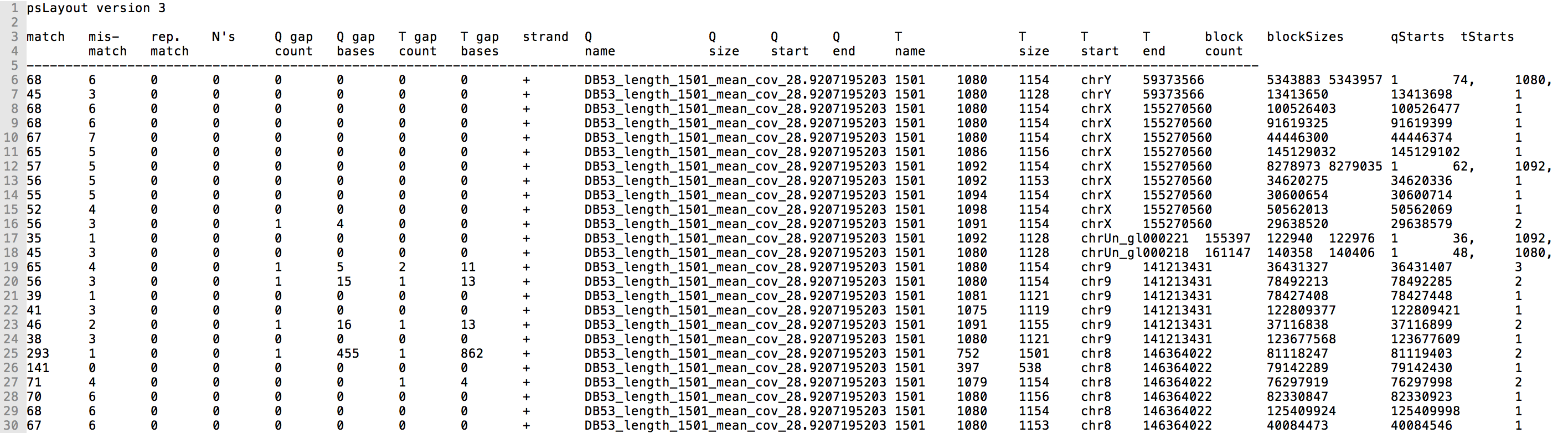
**

Supplemental Table 2. Example output produced by BLAT. The contig depicted in Fig. 5E of length 1501 bp was mapped using BLAT to hg19. The table shows the first 25 alignments in psl format, out of a total of 332 alignments for this contig.
