# Supplementary material for: Chromothripsis during telomere crisis is independent of NHEJ, and consistent with a replicative origin
Source: Genome Res. 2019 May;29(5):737–49. doi: 10.1101/gr.240705.118 (PMC6499312; doi:10.1101/gr.240705.118)
Supplement: Supplemental Material [file supp_gr.240705.118_Supplemental_file_1.zip › contigs/annotated_contigs/DB101/contig.2.DB101_length_311_mean_cov_15.0450160772.docx]

**DB101_length_311_mean_cov_15.0450160772**

CACCCCCCCCCCCCCCCTCCACACACACCCAC|CATACAACCATACCACACACACCACACCCCTCCACATTCACACACCACACTACACA
 >chr6:106459892-106460079 + E=3e-95 p=0e+00
CACACACAAACATACCACACACACCACACCCCTCCACATTCATACACACACACACAAACATACCACACACACCACACCCCTCCACATTC

ACACACACCACACT|ACACACACACACAAACATACCACACACA|TTTGAATGCTTAGCTTCTTTTAACAAAGAGTTATTGAATTGATAC
 >chr7:81917750-81917867 + E=1e-50
ATTCTGTTGAGTCATCCAGGCAGACTAAAAATTATTCCTGCTAAAAT
