# Supplementary material for: Chromothripsis during telomere crisis is independent of NHEJ, and consistent with a replicative origin
Source: Genome Res. 2019 May;29(5):737–49. doi: 10.1101/gr.240705.118 (PMC6499312; doi:10.1101/gr.240705.118)
Supplement: Supplemental Material [file supp_gr.240705.118_Supplemental_file_1.zip › contigs/annotated_contigs/DB101/contig.2.DB101_length_347_mean_cov_4.50720461095.docx]

**DB101_length_347_mean_cov_4.50720461095**

GCAGTGGCACAATCTTGGCCCACTACAACCTCCACGCCCCAGGCTCAAGTGATCCTCCTACCTCAGCCTCCTGAGTAGCTGGAACCACA
 >chr16:29688139-29688298 - E=2e-81 p=0e+00
GGCGTCTGCCACCATGCCCAGCTAATTTG|TGTATTTTTAGTAGAGAAGGGGTTTCACCGTGTTAGCCAGG|ATGGTCTCAATCTCCTG
 >chr1:98313825-98314054 + E=1e-123
ACCTCGTGATCCACCCACCTCGGCTTCCAAAAGTGCTGGGATTGCAGGCTGCACAAATACCACTTCTATTTAAAAACATTTAAGAAACT

TTTAGTGACTATTTTCCAAAAATGTCATATTGAGTGAAGTGAAGTAAATTCAGTTCATCTTTTAAGAGGATGTGAAAGTGTT
