# Supplementary material for: Chromothripsis during telomere crisis is independent of NHEJ, and consistent with a replicative origin
Source: Genome Res. 2019 May;29(5):737–49. doi: 10.1101/gr.240705.118 (PMC6499312; doi:10.1101/gr.240705.118)
Supplement: Supplemental Material [file supp_gr.240705.118_Supplemental_file_1.zip › contigs/annotated_contigs/DB101/contig.2.DB101_length_679_mean_cov_3.9764359352.docx]

**DB101_length_679_mean_cov_3.9764359352**

ATAAATGAATTCCAGGAGATTCTAGAAAAATCAGTTTCAGATAAATCAAGAGAGGAGATTATTTCAAGAAGAAAAGTGTGAAAAATATT
 >chr18:72003458-72003759 - E=2e-164 p=3e-02
ATAAGTGTTTCAAAGATTTCACATATGATTCCCTCAGACATTTTCTTTTTCTCTTAATTCCTCAAGTAACATAGTTCCTATAACACAAA

TTTTGATACTCCATTAACAAACATTGTTTTTAATGTATTTTTATTTAACATATATTAGGTTTCTATTTCTGTAAGGCTCTATGGGACAT

ACAAATTTGAACCAAAAAACCTAAGGCTAGG|TG|GCAACTTGTGCATATAAATTCCTTTAAGAAACATTTAATTACTTCCAGGAAAGC
 >chr18:72002396-72002777 - E=2e-214
ATTATTTTAAAGTTTAGCACAGTCCAAAATTTGAGAAATGAGTAAATTACTGTTTCAATTACCTCTTAAATTTTTTTATCTCTATTTAA

ATTGTAAAGATAAGGGCTGAAATCCCATTTTTCATAATGAATTATAATAAACTGCTTCAAAATTTGTCTAAATAGGGTCAAAGGTCAGG

ATTTAGAAAACTACTCCCATCTCTAGCCCAGAGGAATTAAAAAAGAATTTAGAAACAAAAGTCACCTAGTTCAAAAAATAAATTAGTCT

TTCACTCCAGTCAATGCTCCATTCATTCCATAAATCTTTACTAAAGTGAGTGACAGGC
