# Supplementary material for: Chromothripsis during telomere crisis is independent of NHEJ, and consistent with a replicative origin
Source: Genome Res. 2019 May;29(5):737–49. doi: 10.1101/gr.240705.118 (PMC6499312; doi:10.1101/gr.240705.118)
Supplement: Supplemental Material [file supp_gr.240705.118_Supplemental_file_1.zip › contigs/annotated_contigs/DB101/contig.3.DB101_length_964_mean_cov_6.06846473029.docx]

**DB101_length_964_mean_cov_6.06846473029**

ATTTTTGTATGTTTAGTAGAGACGAAGTTTCACCATGTTGGCCAGGATGGTCTCAATCTCTTGACCTCGTGATCCACCCGCCTCGGCCT
 >chr19:35296267-35296685 + E=2e-239
CCCAAAGTTCTAGGACTACAGGCATGAGCCACCGTGCCCAGCTGCTACCTTTTTTTCTCTCACCCTCAACATTAGAAAATCCAGCATTC

AGCCCTATTTTTAAAAAAGGTCTGGAATCCAGCCACTTTCTCCATTTCCAGATACCACCAACACTTACTTAGATTATTGCAGTAGCCCC

ATCCATGATCTTCCTGCCTCCTCCCTCACTCTCAAAATTTGTTCAGTCTGCAGCCAGAGGGAGCCTATTAAGACCTGAGTCATGTCACC

TCATTGCTCCTATGCCATTGCTCCCATCAACTCCAGAAACCACCCAACTTCTCAGACTG|CCA|AATGGAGTTTGTTCCTGGAATTCAA
 >chr19:35303537-35303832 +
GGCTGGTTTCATATTTAAAATAAATCAATGTAATTTACCACACTAAGAGAATAAAGAAGAAAAGCCATGTAAGTATCTCAAAGAGGTGG
E=2e-165
GAAAAGTGATTGACAAGATTTAATTGCTTTTCAAAAAACAATATCTCAGCAAAAGTAGGAATTGAAGGAAATTTCTTTTGTCTCAAGAC

AAAAGCAGAGCTATGAAGTAATCATAAGGAATTATGCCTTGAGACACACAGCTTTTGTACTAGAGTGGATCCTGCACCCAACTGATG|A

G|AAACCACCCAACTTCTCAGACTGCCATTCCAGATCTGACCCCTACTCTCCTGCTAGCAAGAAAGAGAGGAGATCTGTGATTCTCTGA
>chr19:35296657-35296913 + E=5e-139
ACACTCCCTTCTAAGCCTTACCTTCAAGCATTTGGAGATGGTGGTACCTGTGCTTAGGATAACCTTCCACACCTCATCCTATTGGTTGC

TAGATATACATAGGAACTATTATATATACTACCTCTGGCCGAGGACCATGGGCTTGGGGTTTCTCAAGCAACCCCAGT
