# Supplementary material for: Chromothripsis during telomere crisis is independent of NHEJ, and consistent with a replicative origin
Source: Genome Res. 2019 May;29(5):737–49. doi: 10.1101/gr.240705.118 (PMC6499312; doi:10.1101/gr.240705.118)
Supplement: Supplemental Material [file supp_gr.240705.118_Supplemental_file_1.zip › contigs/annotated_contigs/DB102/contig.2.DB102_length_243_mean_cov_3.71604938272.docx]

**DB102_length_243_mean_cov_3.71604938272**

C|TGGGCTCAAGCGAGCCACCTGCATTGGCCCCCCAAAGTGCTGGGATTACAGGCGTGAGCCACTGCGCTTGGT|GGAGAAGATCGGAA
 >chr15:66820668-66820739 + E=3e-12
GAGCGTCGTGTAGGGAAAGAGTGTCTTCGCCTGTGTAGATCTCGGTGGTCGCCGTATCATTAAAAAA|AATGGCCCCTTTTTCAGTATA
 >chr5:164898848-1648
ATGAATTTTTTATCTTAAACATACTAAATATACTATAAGAAATGATATTTTGACATGATTTCAGGCAA98937 + E=2e-42
