# Supplementary material for: Chromothripsis during telomere crisis is independent of NHEJ, and consistent with a replicative origin
Source: Genome Res. 2019 May;29(5):737–49. doi: 10.1101/gr.240705.118 (PMC6499312; doi:10.1101/gr.240705.118)
Supplement: Supplemental Material [file supp_gr.240705.118_Supplemental_file_1.zip › contigs/annotated_contigs/DB102/contig.2.DB102_length_273_mean_cov_5.89010989011.docx]

**DB102_length_273_mean_cov_5.89010989011**

TCCACGGCTGGGCTTGGGTGGCTGCAGCTGCACCTGTGAGCTCCTACCCAGCAAAATTGGAAAGGGCAGGGCTCCCGCTTGTCCCCGGC
 >chr2:34373193-34373358 + E=4e-85
TCCCCTGGCTCAGTAGAGCATGCAACCCCGGCCATGCCTCTGATATTGGAGCAGGCACTGACAGCAGGGAGAAGGC|CCCTAAAAATTC
 >chrX:8374
AATGTGTCAAAATTTGCCTAAGCTCCCCGGGAGCCTAATTACTGTTGGCACTCAGCTCAGACCATTTTTAGGACAGGTGTGTCTTTTAA
727-8374835 - E=9e-54
GATGCTG
