# Supplementary material for: Chromothripsis during telomere crisis is independent of NHEJ, and consistent with a replicative origin
Source: Genome Res. 2019 May;29(5):737–49. doi: 10.1101/gr.240705.118 (PMC6499312; doi:10.1101/gr.240705.118)
Supplement: Supplemental Material [file supp_gr.240705.118_Supplemental_file_1.zip › contigs/annotated_contigs/DB102/contig.2.DB102_length_601_mean_cov_8.38269550749.docx]

**DB102_length_601_mean_cov_8.38269550749**

GTTTCTCTGCTAGTCCCTTAACTATGTCTCAGAGTCTTATTCAGATTTGCCTATATTCCTTTCTCACTAAGCCTTCTCTACCTCTTCCT
 >chr4:81587385-81587696 + E=2e-169 p=2e-02
TTTTTCCATGTTTTCATCTCATGGTGAAGGCTCACAAATCTCTATCATTTTTGCTCTCTCCCCTCAACTCCAGAACCATATAATCAGAT

TCTACTGGAGATCGCCACTGGGTTCAAATACCATAACATTAGCTTGTCCATCCCAACCTGATCCCAGCCCATGTATCCCCACCACTATT

TGAAAAAGAAAAGAGTCAAGGATATCATCATTTGGATGCCACCT|ATGTAAA|TAACTCGTCATTTACATTAGGTATTTCTCCTAATGC
 >chr4:81591243-81591526 + E=1e-158
TATCCATCCTCTTGCCCCCCTCCCCATGACAGACCCCCATGTGTGATGTTCCCCGCCCTATGTCCAAGTGTTCTCATTGTTCAATTCCC

ACCTATGTATGAAAACATGCAGTGTTTGGTTTTCTATCCTTCTGGTAGTATGCTCAGAATGATGATTTCCAGGTTCACCCATGTCCCTG

CAAAGGACATGAACTCATCCTTTTTTATGGCTGCATAGTATTCCATGGTGTACATGTGCCACAATTTCT
