# Supplementary material for: Chromothripsis during telomere crisis is independent of NHEJ, and consistent with a replicative origin
Source: Genome Res. 2019 May;29(5):737–49. doi: 10.1101/gr.240705.118 (PMC6499312; doi:10.1101/gr.240705.118)
Supplement: Supplemental Material [file supp_gr.240705.118_Supplemental_file_1.zip › contigs/annotated_contigs/DB102/contig.3.DB102_length_342_mean_cov_15.5058479532.docx]

**DB102_length_342_mean_cov_15.5058479532**

TGGGAGCTCACGCCTGTATTCCCAGCACTTTGAGAGGCCTAGGCGGGAGGATCACTTGAGGCCAGGAGTTCAAAACCACTCTGGGCAAC
 >chr2:63299589-63299722 - E=1e-68 p=0e+00
ACAGCGAGACCCTGTCTCTACAAACAATTTAAAACTTAGCCAGG|T|GTGGTGGTGGGCACCTGTAGTCCCAGCTACTTGGGAGGCTGA
 >chr10:113449582-113449746 + E=3e-69
GGCAGGAGAATCGCTTGAACCCGGGAGGCGGAGCTTGCAGTGAGCCGAGATCGCGCCACTGCACTCCAGCCTGGGCGACAGAGCGAGAC

TCCGTCTCAAAAAAAAAAAAAAAAAAAAAATTA|TAAA|ATATATAAATATATATATAAATATATGTAACAATATATA|A >chr18:39157304-39157343 - E=5e-06
