# Supplementary material for: Chromothripsis during telomere crisis is independent of NHEJ, and consistent with a replicative origin
Source: Genome Res. 2019 May;29(5):737–49. doi: 10.1101/gr.240705.118 (PMC6499312; doi:10.1101/gr.240705.118)
Supplement: Supplemental Material [file supp_gr.240705.118_Supplemental_file_1.zip › contigs/annotated_contigs/DB102/contig.3.DB102_length_343_mean_cov_4.81049562682.docx]

**DB102_length_343_mean_cov_4.81049562682**

CACGACGCTCTTCCGATCT|CCACAATCCAGAGAAAACGGGAGGAAAA|CAATCCAGAGAAAA|CGGAAATGAAGGAGATGAGAGGCTG
 >chr7:152147770-152147798 + E=2e-05 p=0e+00 >chr7:152147813-15214
ACTTGGATAGTGGAGGCTGGGACTGAGCCTTGGGAACTGCTCATCCATTTGGGCTATATGTAGGCCAGTCATGGTGGCTCATCACCTCT
8004 + E=1e-100
AATCCCAGTAGTTTGAAAGGCCAAGGCAGGCGGATCCCTTGAGGTTAGGAGTTTGAGACCAGCCTGGACAGCGCAC|T|TTTTTTTGAG
 >chr7:1521
ACGGAGTCTTGCTCTGTCACCCAGGCTGGAGTGCAGTGGCGCGATCTCGGCTCACTGCAACCTCCGCCTTCCACGTTCAAC51239-152151331 + E=6e-35
