# Supplementary material for: Chromothripsis during telomere crisis is independent of NHEJ, and consistent with a replicative origin
Source: Genome Res. 2019 May;29(5):737–49. doi: 10.1101/gr.240705.118 (PMC6499312; doi:10.1101/gr.240705.118)
Supplement: Supplemental Material [file supp_gr.240705.118_Supplemental_file_1.zip › contigs/annotated_contigs/DB103/contig.2.DB103_length_516_mean_cov_9.07751937984.docx]

**DB103_length_516_mean_cov_9.07751937984**

AGCCACTGCGCCCGGCCTATTCTTTCCGTTTCTGGTGGCCACTTATACAATGTGAGGCACAGACTGCCCAAAAACAACCTGTCCCCAGG
 >chr10:129251326-129251668 - E=3e-185
CTGGGCTCTCCGTGGTCGCCTACATGGGTGTGCCCATTCGACGATTTCTAGGGTTGTGGATAATATTCAGTTTCTCACACACTCGACAG

AAGATGAATATTTATCTTGGTGCAACAGTTGAAACAGTAAAGATTCTTGAAGAAAAAAATCCCACATTCACACAGGCCTACAGTTTCTC

GGGGGTGGACTGGATATTTACAAGCATGAATACGCTGGGGACAGCACCTGAGTAGGAGGAAGAGCCTCCCAGC|TT|ACCTGTGTAATC
 >chr10:129514
CTGGATGAGTTAACCTGGACCCTCAATTTTCTTATCTGTAACACAGTAATAATAATCATCTCACAGGGCTTCATTTCCCATCCGGGTTA
464-129514640 - E=3e-91
AATGAAACCATGCATATAAAGCACTTTGCACTTTTCCCGGCACCATACATGTAGCTAATGTTATTAGGGTGGT
