# Supplementary material for: Chromothripsis during telomere crisis is independent of NHEJ, and consistent with a replicative origin
Source: Genome Res. 2019 May;29(5):737–49. doi: 10.1101/gr.240705.118 (PMC6499312; doi:10.1101/gr.240705.118)
Supplement: Supplemental Material [file supp_gr.240705.118_Supplemental_file_1.zip › contigs/annotated_contigs/DB103/contig.2.DB103_length_675_mean_cov_8.37777777778.docx]

**DB103_length_675_mean_cov_8.37777777778**

CACACATTATAAGTAGAAGCATAAGTCTTTCCATTTAAATGGAACCTTGAAAGGCAGAATAAACCCATCTAACAAATCATATCATATAT
 >chr9:106440667-106441016 + E=3e-198 p=3e-02
ATATTATATATATATATGTATATCTGAGAAAGAACCCTTGGAGGAGGATAGACTTCCACTGTAGCTATACCAGTGAAGGAATGATAAGA

CTTTCCTGCCTTCAGTATTTTATATGTGTACTTAGTTAAGGCCCACTGGAAAGAGTTACAGGTATATAGAGATTCTCCTTGCTCTTTGG

CATGGTTCACAGACATGCCAGCACACACCCAATCTTTCAAAAAGTTCTTCTGTCATCTTCTTGCCTTTACAATAGCAGCAC|G|AATCT
 >chr9
CTGCATAGGTCTTACTGGGCTAAAATTAAGGTGTCAACAGGACTACATTCCTTCTTGGGGCACCAAGGGAAAACCACATTTCTTTTTTC
:106439665-106439992 + E=5e-185
AGCTTCTAGAGGCCACCTGCATTCCTTAGCTCATAATCCCCTTCCATCTTCAGACCCCAAAATTGCATCAACACAAACCTCTGCTTGTC

TTATCTCCTCAGACTCTGACATTTCCTGCCTCCATCTTTCACTTATTAGGACTCTTGTGATGACGTTGGACATGAGTGAACATAATACA

GGACACTTTCTCAGTCTCAATATTCTTAACCTAATTACATCCGCAAAGTCTCTT
