# Supplementary material for: Chromothripsis during telomere crisis is independent of NHEJ, and consistent with a replicative origin
Source: Genome Res. 2019 May;29(5):737–49. doi: 10.1101/gr.240705.118 (PMC6499312; doi:10.1101/gr.240705.118)
Supplement: Supplemental Material [file supp_gr.240705.118_Supplemental_file_1.zip › contigs/annotated_contigs/DB103/contig.3.DB103_length_482_mean_cov_5.67842323651.docx]

**DB103_length_482_mean_cov_5.67842323651**

GGCGTGAGCCACCGTGCCCCACCTTTAATTAACATTTCTGTTAGTGTTTT|TTTTTATGATTCGGCGACCACCGAGATCTACACAGGCG
 >chr3:142233162-142233212 - E=2e-18
GAGACACTCTTTCCCTACACGACGCTCTTCCGATCT|AATTAGCTGGTATGGTGGCGGGTGCCTGTAGTTCCAGATACTCGGGAGGCTG
 >chr3:142232964-142233115 + E=9e-50
AGGCAGGAGAACCACTTAAACCCGGGAGGCGGAGGTTGCAGTGAGCCAAGATGGAGCCACTGCACTCCAGCCTGGGCAACAAAGCAAGA

CTCCGTCTC|AAAAAAAAA|AAAAAAGAAAGGTAAATTAAAAACAAAAATTGTAAATTTTAAATATACTGTAAACATTTTCTTGGAACA
 >chr3:142233655-142233863 + E=1e-113
CAGAGTACCTTTTGTCTATCCAGCATGTAAAAATTTCTCATTAAGGAAATAGAAATATACCTGTTTTTCTATGAAAATTCATTGTAAAA

ATCTTTATTTTTATTTTAGAGAAAGTTCAGATAATATAACT
