# Supplementary material for: Chromothripsis during telomere crisis is independent of NHEJ, and consistent with a replicative origin
Source: Genome Res. 2019 May;29(5):737–49. doi: 10.1101/gr.240705.118 (PMC6499312; doi:10.1101/gr.240705.118)
Supplement: Supplemental Material [file supp_gr.240705.118_Supplemental_file_1.zip › contigs/annotated_contigs/DB103/contig.3.DB103_length_547_mean_cov_5.16270566728.docx]

**DB103_length_547_mean_cov_5.16270566728**

AGGCATAATTTTTTTCAGTTCATGCCTGGTTAAGGATAAAATATATGACTGATTTATGGACTAGGTTATATTTTTTGAGGAATGGGGAA
 >chr1:114504036-114504283 + E=5e-137 p=9e-03
AAGCATTCTAAATAAGAAGGTAACTTAGATTATAAATTATTGAGCTTTTCGTAATCTTTCTTATCTAATTCACTAAAAGTTATACTTAA

GCCTTTGCTTTTAAAGATTAGAATTTTAAAAACAAGTTTTTTTTCCACCTAGTGGATTAAAAAGT|GAAT|CAAGCCTTTGATCAGTTT
 >chr1:114430644-11443
AATAGGGAAAC|TA|TTGAGCCCAGGAGGTGGAGGCTGCAGTGAGCTGAGATCACACCACTGCACTTGAGCCTGGGTGACACAGTAAGA
0677 + E=3e-08 >chr1:114428754-114429032 + E=3e-141
CCCTGTCTGAAAAAAAAAAAGATACTATACTTTGCCAAGAATAAAGTGTTATAAATGTGCTCTGTGCACACTAAGCACAACAGCTGTGA

TGAAGACCAAGTAGAGACAAACTGAAGGTTCATCAGCCTATGCCCAAAATTTTCCATCTCTGTACCACTTAGGTTTTAGCAATTGGAGA

ATTCTTGCTATACTTCC
