# Supplementary material for: Chromothripsis during telomere crisis is independent of NHEJ, and consistent with a replicative origin
Source: Genome Res. 2019 May;29(5):737–49. doi: 10.1101/gr.240705.118 (PMC6499312; doi:10.1101/gr.240705.118)
Supplement: Supplemental Material [file supp_gr.240705.118_Supplemental_file_1.zip › contigs/annotated_contigs/DB104/contig.2.DB104_length_264_mean_cov_11.0416666667.docx]

**DB104_length_264_mean_cov_11.0416666667**

CCCCCCCTCCTTCCTTCTTTCCTTC|CTTC|CCTCCCTCCCTCCCTCCTTCCTTCCTTCCCTTCTTACTTTCCCTCTCTCCCTCCCTCC
 >chr1:24439870-24439899 + E=4e-03 p=0e+00 >chr12:7253724-7253916 + E=1e-101
CTTCTTTCTTTCCCTCTTTCTCCTTTCTCGCTTTCTTCCCTCCCTTCCCTTCCCTCTCCCTTTTCCTCCTTCCTTCCTTCCTCTCTTCC

TCGCTTTCTCTCTTCTAGTTTCTCCGTGTCTCCCAGGCTGG|CGGGCGGGGGCCTGAGTGTGTCTGATCGGCCCCTCACCACCTCCGGC
