# Supplementary material for: Chromothripsis during telomere crisis is independent of NHEJ, and consistent with a replicative origin
Source: Genome Res. 2019 May;29(5):737–49. doi: 10.1101/gr.240705.118 (PMC6499312; doi:10.1101/gr.240705.118)
Supplement: Supplemental Material [file supp_gr.240705.118_Supplemental_file_1.zip › contigs/annotated_contigs/DB104/contig.2.DB104_length_268_mean_cov_3.35820895522.docx]

**DB104_length_268_mean_cov_3.35820895522**

CCAAGTCTCACCTTCATCTGAATCAAAGAGCATGTCTGAAGTGATAGTTTTAACTTCCTATTCTGATGGTAGGGAGGATGGTAGGAAGG
 >chr6:110885788-110885990 - E=1e-99
ATGGGGGGGGTGCGTTCTTTGTCCTTGTGGATTTCTGATTTTTTTTAAAAAAAATTATTTTTGTTTTTTGTAGAGACGGGGTCTCCCTA

TGTTGCCCCGGCTAGTCTT|GAAC|GCCTGGCCTCAGGTGATCCACCCACCTTGGCCTCCCAAGGTGCTCGGTTTACAGGCAGGAGCC|
 >chr6:110886898-110886965 + E=6e-17
TCTC
