# Supplementary material for: Chromothripsis during telomere crisis is independent of NHEJ, and consistent with a replicative origin
Source: Genome Res. 2019 May;29(5):737–49. doi: 10.1101/gr.240705.118 (PMC6499312; doi:10.1101/gr.240705.118)
Supplement: Supplemental Material [file supp_gr.240705.118_Supplemental_file_1.zip › contigs/annotated_contigs/DB104/contig.2.DB104_length_344_mean_cov_6.10465116279.docx]

**DB104_length_344_mean_cov_6.10465116279**

GAAATCACCACCACCGAATGGAGGATTTGATATGAATGAACGACATCTTTCTTCTTATATAATCATATAACAAATCACATTATTAAATG
 >chr2:5491146-5491257 - E=2e-55
TTAAGTAAAAACATTACAAG|TT|CGACCTCTGTTCAGACGTCAGGGGAGATCTCCAGGCAGGTCAGTACCTTCTCTGTCTGGCATTCA
 >chr2:5490089-5490324 - E=3e-130
GTTGGGGTCTTCAGTATGCTGAGAATCCTTCTTTACTAAGGCAGAGTCCGTCCAGATGTACCCAGACATCACAGCCCTCACCAGCCTGT

GATTACAAAATAAGGTGACCTTCGTTTTAAAGGCCAAATTATCAATTGAAACACACTGGCCTGGTCACTGGAAATTAAG
