# Supplementary material for: Chromothripsis during telomere crisis is independent of NHEJ, and consistent with a replicative origin
Source: Genome Res. 2019 May;29(5):737–49. doi: 10.1101/gr.240705.118 (PMC6499312; doi:10.1101/gr.240705.118)
Supplement: Supplemental Material [file supp_gr.240705.118_Supplemental_file_1.zip › contigs/annotated_contigs/DB104/contig.2.DB104_length_439_mean_cov_5.6013667426.docx]

**DB104_length_439_mean_cov_5.6013667426**

TG|GTGGCTCACGTCTGTAATCCCAAACACTTTGGGAGGCCGAGGTGGGCGGATCACCTGAGGTCGGCAGTTCAAGACCAGCCTGACCA
 >chr5:37352053-37352382 + E=2e-164 p=0e+00
ACATGAAGACGCCCCGTCTCTTCTAAAAATACAAAATTAGGCCGGGCACAGTGGCTCACGCCTGTAATCCCAGCACTTTGGGAGGCCAA

GGCAGGTGGATCACGAGGTCAGGAGTTCAAGAACAGCCTGGCCAACACAGTAAAACCCTGTCTCTACTAAAAATACAAAAAAATTAGCT

GGGTGTGGTAGTGGGTGCCTGTAATCCCAGCTACTTGGGAGGCTGAGGCAGGAGAATCGCTTGAAC|TCAGGAGGCGAGACTGCAGTGA

GCACCAAGGTGGCTAA|ACTGCACTCTAGCCTGGGCAACAGAGTGAGACTCTGTAACAGAAAAA|GCAGAGGGGTGGATTGAAAGGC >chr12:48564897-48564944 + E=1e-10
