# Supplementary material for: Chromothripsis during telomere crisis is independent of NHEJ, and consistent with a replicative origin
Source: Genome Res. 2019 May;29(5):737–49. doi: 10.1101/gr.240705.118 (PMC6499312; doi:10.1101/gr.240705.118)
Supplement: Supplemental Material [file supp_gr.240705.118_Supplemental_file_1.zip › contigs/annotated_contigs/DB104/contig.2.DB104_length_444_mean_cov_4.05405405405.docx]

**DB104_length_444_mean_cov_4.05405405405**

TCATGATAGTTACATTTATGTACACACTTATCAAAATGTATTAAATTATAAATTTAAGATGGCACTTTCTTCCATGTATGCTATAAAAT
 >chr18:30970687-30970888 + E=2e-109 p=3e-02
AAAAAAATTGTTTTACTCTAAAACCACTAAAATATTTTTATATATTATAAATACATACACACATACATACATAAATATAAGGTTAAAAT

GCTTGCAGATTAACTTATGTTAA|CACTTGCTGCTTCACTGTGCACTTTCATGCTATGGAGATGACTTTTTTCCTTAGACTTCATGAAC
 >chr18:30980457-30980700 + E=9e-135
CAACTTCTGCAAGCTTCCAACTTTCCCTCTGCAGCTTCTTCACCTCTCTTAGCCTTTATAGAACTGAATAGAGTTAGGGCCTTGCTCTT

GATTAAGCTTTTGTTTAAGGGAATGTTGTGTCTGGTTTGATCTTCTATCCAGACCACTAAAACTTTCTTCGTATCAGCAATAAGGGTGT
