# Supplementary material for: Chromothripsis during telomere crisis is independent of NHEJ, and consistent with a replicative origin
Source: Genome Res. 2019 May;29(5):737–49. doi: 10.1101/gr.240705.118 (PMC6499312; doi:10.1101/gr.240705.118)
Supplement: Supplemental Material [file supp_gr.240705.118_Supplemental_file_1.zip › contigs/annotated_contigs/DB104/contig.2.DB104_length_460_mean_cov_4.23913043478.docx]

**DB104_length_460_mean_cov_4.23913043478**

TGAAACTATCATCAGAGTGAACAGGCAACATACAGAATGGGAGAAAATTTTTGCACTCTGTCCATCTGACAAAGGCCTAATATCCAGAA
 >chr9:5298203-5298457 + E=2e-133
TCTACCAAGAACTTAAACAACTTCACAAAAAAAAAACCCCATCAAAAAGTGGGCAAAGGATATGAACAGATACTTCTCAAAAGAAGACA

TTTATGCAGCCAACAGACATGAAAAAATACTCATCATCACAGGTCATTAGAGAAATGCAAATCAAAACCACAA|TG|GGCAATATGGCC
 >chr9:5297414
ATTGTCATGATATTGATTCTTTGTATCCACGAGCATGGAATGCTTTTCCATTTGTTTGTATCCTCTCTTATATTCTTTAGCAGTGGTTT
-5297596 - E=8e-59
GTATTTGTCTTTGAACAGGTATTTCACCTCGGTTGTAATTTGTTTTGCTAGTTATTGTATTTTTTTAGTAGCAATTGTG|TAGGTTTGG

GTGCTCATGTTTTTGCTG
