# Supplementary material for: Chromothripsis during telomere crisis is independent of NHEJ, and consistent with a replicative origin
Source: Genome Res. 2019 May;29(5):737–49. doi: 10.1101/gr.240705.118 (PMC6499312; doi:10.1101/gr.240705.118)
Supplement: Supplemental Material [file supp_gr.240705.118_Supplemental_file_1.zip › contigs/annotated_contigs/DB104/contig.3.DB104_length_455_mean_cov_13.1868131868.docx]

**DB104_length_455_mean_cov_13.1868131868**

AAAAACAGGTGAGCGAGAGTGGGAATGG|CATAAGTAAGTGCAAGGGGAGGTACTATGCCTGGATGTGCATGTAGGCCAGATTTATGTT
 >chr4:191033980-191034180 + E=9e-97
ACTGTCCACCCAAACATTTCAGTGGAGTAAAGAATAACTAGACAGCATTGCTGCCAACATGTCTCACCTCCCACCATAGGGTGGTTTTC

CTCCTACCTCAGAATTGAACAGGTGTACAATCGGGTTTTATACCGAGAC|AT|TTTGGGGGTATTCCAAAAGATACATGTTATGAATGG
 >chr7:112601878-112602056 + E=9e-88
AATCGTGTTCTTCCCTTCCCCAAATTCATTTTGTGCTGCCCAATCCCAATGTATTTGGAGAAGAGCCCTTAAAAAAATCATTAAGGTTA

AATAAGGTCACAAGGGTAGAACCCTAATCTCATAGGACTGATGTCCTTAT|CCCGGAGGTGCCGACCGAGACGGGGACGCGGCGGGTCC
 >chrUn_gl000220:119733-119785 - E=1e
GGCTCGTCCCGACG-19
