# Supplementary material for: Chromothripsis during telomere crisis is independent of NHEJ, and consistent with a replicative origin
Source: Genome Res. 2019 May;29(5):737–49. doi: 10.1101/gr.240705.118 (PMC6499312; doi:10.1101/gr.240705.118)
Supplement: Supplemental Material [file supp_gr.240705.118_Supplemental_file_1.zip › contigs/annotated_contigs/DB105/contig.2.DB105_length_281_mean_cov_3.73665480427.docx]

**DB105_length_281_mean_cov_3.73665480427**

AGGCCCTTTCTCCTCCTTCTTCTCTCAAAA|TCCC|AGCTTGTCCCAATGCTCATTAGTGGTTGGATGCAGAAATTCCCAACGATTCCT
 >chr16:72410078-72410108 - E=1e-06 >chr16:72407338-72407585 - E=5e-138
GCTAATCCGCTAATTTGCCTATTTCCATCAATAGTTACTGGCTATTTGTTTCTGCTGCCCAGTCTTCACCTAACTTGCCTTACAAGAGC

TTGCTTTTAAACTGAGAATTTCTTTGCTTGCTTTGCCATATCCTTGCTTGGCTTTTCTTTCTTTAACAAGCACAACAGCATGCTGCTGC

ATCTTCAAAGAGTAAA
