# Supplementary material for: Chromothripsis during telomere crisis is independent of NHEJ, and consistent with a replicative origin
Source: Genome Res. 2019 May;29(5):737–49. doi: 10.1101/gr.240705.118 (PMC6499312; doi:10.1101/gr.240705.118)
Supplement: Supplemental Material [file supp_gr.240705.118_Supplemental_file_1.zip › contigs/annotated_contigs/DB105/contig.2.DB105_length_300_mean_cov_6.0.docx]

**DB105_length_300_mean_cov_6.0**

ACATTAAAAATGGTAATAAAGACATGCTTCTCAGTTTTACAATATCATCTATGTAAAATGAATGTATTAAAAATTAATCAAACTCATGT
 >chr11:75375188-75375392 + E=1e-108 p=4e-02
TTGTGAATAATCCCAAAGCTATCCTGGATACTACTTGAATGCGAAAATCTCAGGCTGATATGAATGGATTGGGAATTAGATGAGCTATT

GAATAGGGAAGGTTTCTCAAAACCA|T|GTAAAATGGGCACACAATTAAACATGGCCCATGGAGATGTTACAACAATGACTAGGGCAAT
 >chr11:75186053-75186150 + E=5e-47
GCATGTAAACTGTTTGCTCCCTGCATACAGTAGGT
