# Supplementary material for: Chromothripsis during telomere crisis is independent of NHEJ, and consistent with a replicative origin
Source: Genome Res. 2019 May;29(5):737–49. doi: 10.1101/gr.240705.118 (PMC6499312; doi:10.1101/gr.240705.118)
Supplement: Supplemental Material [file supp_gr.240705.118_Supplemental_file_1.zip › contigs/annotated_contigs/DB105/contig.2.DB105_length_305_mean_cov_4.4262295082.docx]

**DB105_length_305_mean_cov_4.4262295082**

TAAATTTGGGGACATCAGTCCATAGGGGATATGTTCCAGAAGGTTGTATTCTCATCTGGGATCTACACGTGGTGAGCTGAAAAACTTCT
 >chr8:64905317-64905496 - E=2e-90
TCTAGATTTTTCTTTATTTTGATGATTACACAATGAATGAAATTTACCTCCTTCTTGTTTCAGAATTATATATATATATATATATATAT

A|ATATATAGGGAAAAT|GAATTCATTCACCATTTTCTCCTCTAATTAAGGATTAGATTGGATTGTTCAGAAAAACATAACATTTTATT
 >chr8:64875184-64875295 - E=2e-55
GGGTGTGTTACATTTTACGTATTGGGATGAGCACCTTTGA
