# Supplementary material for: Chromothripsis during telomere crisis is independent of NHEJ, and consistent with a replicative origin
Source: Genome Res. 2019 May;29(5):737–49. doi: 10.1101/gr.240705.118 (PMC6499312; doi:10.1101/gr.240705.118)
Supplement: Supplemental Material [file supp_gr.240705.118_Supplemental_file_1.zip › contigs/annotated_contigs/DB105/contig.2.DB105_length_340_mean_cov_3.97058823529.docx]

**DB105_length_340_mean_cov_3.97058823529**

TGGGAAAATATGTGCATGGTAGAATCATGCAAATTACTGTGTAAAACTTCCTATAATTTGAAAGGTATTATGATATTTTATCTACCTCA
 >chr20:15183317-15183503 + E=1e-97
TGACCCAAGATGGCTGCTGGAGTTCCAGCCATCATCAGGTTAGAGTATGTCAGTGTAGACAGAAAGAAAAAGGAAAGTCAGAAGAGCAA

AATGAAAT|TTTT|CTTATCTACTTAAGAAAAGAAACTTTTAAGGCATTTCTTTACCTAAGGCCCTGGCCACCCTCTTGTGTCTCCTCT
 >chr20:15230634-15230784 + E=6e-79
GCTGCCACTCTTAAATACAAATTTTTGGCTACCTGGAAGGTCCAGGAAGGTCATCATGCCTTTGTTTGTGATAGC
