# Supplementary material for: Chromothripsis during telomere crisis is independent of NHEJ, and consistent with a replicative origin
Source: Genome Res. 2019 May;29(5):737–49. doi: 10.1101/gr.240705.118 (PMC6499312; doi:10.1101/gr.240705.118)
Supplement: Supplemental Material [file supp_gr.240705.118_Supplemental_file_1.zip › contigs/annotated_contigs/DB105/contig.2.DB105_length_381_mean_cov_9.44881889764.docx]

**DB105_length_381_mean_cov_9.44881889764**

ATGCTCTGGATATTTCCTTATTTGCTGTACTTTCCGAGCACTTGAAATCATCCCTTAACTTTAGTCTCAAAGGCCTGAAATATAAAGAT
 >chr5:99955735-99955926 - E=1e-103 p=0e+00
GTCTGTATTAGATTCCAATTGCTGCCTTAGCAAACTGCTACAAACTCAGTGACATAAACCTATACAAATTTAAAATCTTGCAATTCTGA

AGGTCAGAAGTTA|CCTTTCTTAGAAATATAG|TACCTTTCTTAGAAATATAGTACAATATATTCTTAATGAAATATTATTTCTCAATG
 >chr5:100353590-100353762 - E=4e-92
GTAACACATGATCAAAGTTTGATCTTTGTAATATATTGAAAACTACAAATTACACTTATACCCTAAAATAGGGCATTTCCAATAAACAT

TTGAACTACACTGCCACTCATGTCGCA
