# Supplementary material for: Chromothripsis during telomere crisis is independent of NHEJ, and consistent with a replicative origin
Source: Genome Res. 2019 May;29(5):737–49. doi: 10.1101/gr.240705.118 (PMC6499312; doi:10.1101/gr.240705.118)
Supplement: Supplemental Material [file supp_gr.240705.118_Supplemental_file_1.zip › contigs/annotated_contigs/DB105/contig.2.DB105_length_443_mean_cov_12.1896162528.docx]

**DB105_length_443_mean_cov_12.1896162528**

TC|AACAAGGATACCCAGGAATTGAACTCAGCTCTGCACCAAGCAGACCTAATAGACATCTACAGAACTCTCCACCCCAAATCCATGGA
 >chr11:26906653-26906813 + E=1e-72
ATATACATTCTTCTCAGGTCTACATCGCACTTATTCTAAAATTGACCACATAGTTGGAAGTAAAGCACTCCTCA|TATATATTTATAAA

AGG|GACTTCATGGCAGCCCCTTCCATTTCTTGCCCAGAGGCCTAAGAGGAAGAGGAAAAAATGATTTGTTGTCCAGGCCAAGTGCCCA
 >chr11:26900625-26900889 - E=2e-147
GCTGCCTGGCATAGCCTCAGGACACTGCTCCCTGTATTCCAGCCACTCTGGCTTCAGCCTCAGCTCAAATAGTCCCAGGTCCAACTTGG

GCCACAACTCAGGAGGGTGCAACCCATAAGCCTTGGTGGTTTCCATGTAGTGTTAAGTCTGTAGATGCTCAGAATGTGAGTGTGAAGAA

G
