# Supplementary material for: Chromothripsis during telomere crisis is independent of NHEJ, and consistent with a replicative origin
Source: Genome Res. 2019 May;29(5):737–49. doi: 10.1101/gr.240705.118 (PMC6499312; doi:10.1101/gr.240705.118)
Supplement: Supplemental Material [file supp_gr.240705.118_Supplemental_file_1.zip › contigs/annotated_contigs/DB105/contig.2.DB105_length_569_mean_cov_7.64499121265.docx]

**DB105_length_569_mean_cov_7.64499121265**

TAACTGGTTGAAATTTAAAAATAAACAAAAAAACATTTAAGAATAATCACTATAAAATTTATAAAGAAAATATTATTGATATCCTAAGA
 >chr5:28923454-28923671 - E=5e-105 p=4e-02
GAAGATAAAATGAAATCACATAAAGTATTCAATTCATACCACAGAAGGCAAGTAAAGAGAGGAAGATAAGAAAAGAACCAAACAAGTGC

AACTTTAGTAAGGAGTAAAATATGGTAGATATT|AA|AAATTGACCAAAAGAAAATTCAGCCACGAATAAGCTATTGCAGCAACGTTGT
 >chr5:28922494-28922852 - E=8e-201
AACATACTAGTTTGATGTACAAAAGGTTGTTGCTGTTCTATATACCTTTGATAGACAACTGGAATTTGAAACAAAAAATACAATACCGT

TTATAACAGCACTAAATAACATGAAGTACTTTGGTAAAAATCTAACAAAATATGTATACCATTTATATACCCACTCTTTCTTTGTCTAG

CTAAGACAAAAATGATGACATGAAATTTCCCCTGGATCAGAGCATATATCGTATTTTTTTGCGACATTACTGAAACTCAGGTTAACGTT

ATAAAAGGTACGTTAATAGATATCACAATGTTCTATA
