# Supplementary material for: Chromothripsis during telomere crisis is independent of NHEJ, and consistent with a replicative origin
Source: Genome Res. 2019 May;29(5):737–49. doi: 10.1101/gr.240705.118 (PMC6499312; doi:10.1101/gr.240705.118)
Supplement: Supplemental Material [file supp_gr.240705.118_Supplemental_file_1.zip › contigs/annotated_contigs/DB105/contig.2.DB105_length_601_mean_cov_4.23960066556.docx]

**DB105_length_601_mean_cov_4.23960066556**

CCACCTTCTTAAACTAAAATCCAAGAAGTGCTATGAGATTTGATAGCCACTGAGTCCTAGAATGAAGACATGTAGAAGTTCTTGTTTGT
 >chr11:90286190-90286410 - E=1e-120
TTCCTTTTAGACATTTATTCATTTTCTTATATGACTTACAATTTTTCAGACTGCAGTTAGTCAAATTACTAATGCCAGTATATAATGTC

TTTACAGAAAGTTCCTTATTATCTGCAAAATGTGTAGTATG|C|TCCTTGGATCTAAAATGAAAGTTGAAATTAAAAAACAAAAAAAAG
 >chr11:90272427-90272812 - E=8e-213
GAATTACCAAAAGTAAGTATAGAAAAAGAGACAAACATTTATGGTAGTGAGGTTTGCTACACATAAAGATAGCAAGAACACACAGGGCA

GTAGAAAGTGCTACGGCAGACAAGAAGGTGCTAATCATAAAACTAGAATACCAATAAATATTAAAACATGGGCTCTGCTTTTACCATCA

TATACAGAGAGAGATCTGCCAGAGAGAGATTTATGGAAAGTTAAAGAACAGCGGTCAGTATAGCTCTGATCTTGGTGTCCATGGCTTCT

CTGGCTTCCATTATAGTAATCTTAGGTGAACTTTAAAAAAAAAAGTGTATGATTTAGTTCATAATTCTG
