# Supplementary material for: Chromothripsis during telomere crisis is independent of NHEJ, and consistent with a replicative origin
Source: Genome Res. 2019 May;29(5):737–49. doi: 10.1101/gr.240705.118 (PMC6499312; doi:10.1101/gr.240705.118)
Supplement: Supplemental Material [file supp_gr.240705.118_Supplemental_file_1.zip › contigs/annotated_contigs/DB105/contig.2.DB105_length_636_mean_cov_7.60377358491.docx]

**DB105_length_636_mean_cov_7.60377358491**

CAAGTTATGAGTTTCCATTCTTCCCTTCATCTCCAGCTCCAAAGAAACTGAGAAGTCTTGAAGCATCCTATAAACCAAATGCGACATTC
 >chr3:26774077-26774242 - E=2e-81
CATTGAAAAGGAGATAGGATTTCATGAAAAGAATCTGTCTCTGAAAAATCAGGAATGATGTATTTCTCTAGAATA|T|CTGAATAGACC
 >chr3:26769
AATAACAGGATCGGAAATTGTGGCAATAATCAATAGCTTACCAACCAAAAAGAGTCCAGGACCAGATGGATTCACAGCCGAATTCTACC
369-26769841 - E=2e-257
AGAGGTACAAGGAGGTACTGGTACCATTCCTTCTGAAACTATTCCAATCAATAGAAAAAGACAAGGTATTTCTGAAATATGAGAAAACT

TGATTCTTTCTGCTCACCAGGTCAGCACCAATTGTTGTCCCCTGCAGAACATGCTGGGTGGGGGAATTTTATCCCTAGACATATGGTTG

AAATTCATTAGGGGTATGATTAACTAAAGGAAGAATTGTCCCAGCTGGACACAATCTATGTTCTCACCATTCAGCTAGAGGAAAAGCAG

GAAAGGCCATTTCACCTAAGTCACCTACTCATCGATATATGGCCTACCTGGAAATCCCAAGGACTGGGATTAAGGCAGTGCATCTGATT

TCTCAGAACCAATAG
