# Supplementary material for: Chromothripsis during telomere crisis is independent of NHEJ, and consistent with a replicative origin
Source: Genome Res. 2019 May;29(5):737–49. doi: 10.1101/gr.240705.118 (PMC6499312; doi:10.1101/gr.240705.118)
Supplement: Supplemental Material [file supp_gr.240705.118_Supplemental_file_1.zip › contigs/annotated_contigs/DB105/contig.2.DB105_length_693_mean_cov_13.7027417027.docx]

**DB105_length_693_mean_cov_13.7027417027**

AGAAACAGCACCAAGCACACTCAGGAATAGCTGCACCTTCGAACCTGGCCACTCACCTGGTCAGCTAAGGATGTAGAGAGCATGCCCAT
 >chr22:33780121-33780447 + E=1e-168 p=3e-03
GAGCTCCCTCTCTGCGGTCAGCCTCCACATCTGCTCCCATTTCATCTTCCGCAGCTTATCCAGAAGTAACAGGATCACCCCTGGGCAAC

AGCACAGGATAAAAGAGAAAATTAAAAAATAAAAAAGATGGAGAGCTACTCACATCACAGCCACAGTCTCTCGGGGATGATTGTCTTAT

GCAGACAAGAGGCCTATGCACTGCTGCAGATGAGTCTTCCCTCTAAAAA|AGAT|GGAGACGGAGGATGCGGTGAGCTGAGATTGCACC
 >chr22:33783338-33783716 + E=5e-2
ACTGTCCTCCAGTGGGGGTAACAGAGTGAGACTCTGTCTCAAAAAAAAAAAAAAAGAAAGAAAGAATTTCATTAATCTTATTACAAAAA
06
TGAATTATAATTAGGTTGGATAGATGCTCCAATGTATTAAAAAATGGGGGGGGGGATGGTGGGAAACTAGAAACAATGATGAAATATAT

TAATGCACATTTTTGAAGCCTCTAGAGAGGTCCAGGATAAACCTATGAGTCTCTGGTGACCAAGCTGAGAACTGATGCTTTTAAATGGT

GTGAGACTTGGCAGATGTGATTTGCCAGGTTGCCTGAAAAGTCGGAATGTCTAAAACAGATGACTAAATGCA
