# Supplementary material for: Chromothripsis during telomere crisis is independent of NHEJ, and consistent with a replicative origin
Source: Genome Res. 2019 May;29(5):737–49. doi: 10.1101/gr.240705.118 (PMC6499312; doi:10.1101/gr.240705.118)
Supplement: Supplemental Material [file supp_gr.240705.118_Supplemental_file_1.zip › contigs/annotated_contigs/DB106/contig.2.DB106_length_310_mean_cov_18.4096774194.docx]

**DB106_length_310_mean_cov_18.4096774194**

GACTAATGGTACAGTTTTTCTTTTCCTGGTTAGATTTTTAAAATGAAACATTATTTTTGGAATTTTAGAACTTGCACTGCCCTGCATCC
 >chr6:37813661-37813807 + E=1e-76 p=0e+00
TGGAATTTGTGAAGTGAAGTGTTCTCTCATCTCTACCTCAGCTCTTAGAATTGCTCT|AAGAGCTATTCTCAGCTCTTAGAATTGCTCT

AAGGCTGGGCACGGTGGCTCATGCCTGTAATCCCAGCACTTTGGGAGGCCAAGATGGGCAGATCACGAAGTCAGGAGTTCGAGACCAGC
 >chr6:37350471-37350604 + E=1e-65
CTGGCCAACATGGTGAAACCCCGTCTCTACTAAAAATACAAAAA
