# Supplementary material for: Chromothripsis during telomere crisis is independent of NHEJ, and consistent with a replicative origin
Source: Genome Res. 2019 May;29(5):737–49. doi: 10.1101/gr.240705.118 (PMC6499312; doi:10.1101/gr.240705.118)
Supplement: Supplemental Material [file supp_gr.240705.118_Supplemental_file_1.zip › contigs/annotated_contigs/DB106/contig.2.DB106_length_318_mean_cov_8.01886792453.docx]

**DB106_length_318_mean_cov_8.01886792453**

AAAGCTGAAACTGGATCCCTTCCTTACACCTTATACAAAAATTAATTCAAGATGGATTAAAGACTTAAATGTTAGATCTTAAACCATAA
 >chr3:60427979-60428215 - E=6e-131
AAACCCTAGAAGAAAACCTAGGCAATACCATTCAGGACATAGGCATGGGGGGAGTTTTTCTTTTCTATAGACCATCTTCCCACCACCAG

AAGTTCTTTTGATTCTATGTAAAAAGTCTTTGTCACTTAGGAGGATGAATCAGGAA|CC|AGGTTTCCCAGTACCATTTATTAAATAGA
 >chr3:60180019-60180103 - E=3e
GTGTCCTTTCCCCAGTTTATGTTTTTTAATGCTTTGTTTCAGATCACTTGGTC-39
