# Supplementary material for: Chromothripsis during telomere crisis is independent of NHEJ, and consistent with a replicative origin
Source: Genome Res. 2019 May;29(5):737–49. doi: 10.1101/gr.240705.118 (PMC6499312; doi:10.1101/gr.240705.118)
Supplement: Supplemental Material [file supp_gr.240705.118_Supplemental_file_1.zip › contigs/annotated_contigs/DB106/contig.2.DB106_length_435_mean_cov_7.24137931034.docx]

**DB106_length_435_mean_cov_7.24137931034**

GGATGGTGAGAGAGAAAACAACAAAATGACAATAACAAAACCTCCCGCTCCAGATAAGCATGCAAAGGCACTATACTATCCTTTGCTCC
 >chr5:104466813-104467071 - E=8e-144
AGACAACCGTTTTTACGATGTTTGTATAATGAAGAGTCATGGAAGATACAGAAAATGTCTCCCTCTGGCACAAAGGCCAAGCATACTCA

TTTGCTCATTGCTGATAATTACAGAGTCTGGTCCTCTCCTGTAATTCTAACCACTGCAAGTGCAGGCATCTATCTAGGCC|T|GCAAAA
 >chr5
CAGCAATCGATTTTTGATGAGTCCTTAAGGAAATTTAGCAGAATATGTGAATGAGTAGACATGCAACATGCAGGCCTTAAAGCTTAAGG
:104234322-104234498 - E=2e-94
TTTTGTAGTAGAAGAAAAGTACATTGCGGGCGATTACCACAGCTATGAATTGCTGAAGAGAAGTGAAATGACAGCCACTGT
