# Supplementary material for: Chromothripsis during telomere crisis is independent of NHEJ, and consistent with a replicative origin
Source: Genome Res. 2019 May;29(5):737–49. doi: 10.1101/gr.240705.118 (PMC6499312; doi:10.1101/gr.240705.118)
Supplement: Supplemental Material [file supp_gr.240705.118_Supplemental_file_1.zip › contigs/annotated_contigs/DB106/contig.2.DB106_length_473_mean_cov_6.34249471459.docx]

**DB106_length_473_mean_cov_6.34249471459**

GAATCAATATTAGTATTTCCTTTACCTATTTTTTTAGTAAAATTGCTAATTGACTGCATACATTTACCTAAAAGCAAAACTTATATTTT
 >chr2:187123758-187123995 - E=3e-126
CTGCATTTCTTACATGGTACATAATGATATTTTTATTATCAACCAGATAATGATTCATAAATTTATAGTTTGTTTATTTTACTACCATG

CATGTCGAGGACTTTTTAAATTTCTTTTTGAAAAAATGTCTATGGTAGGCACAGCT|AT|AAGACATTCAGGGATAATGCTAATACTTA
 >chr2:187087103-187087342 - E=
CTAATTTTTCTTCTTTTGCATAGTAAGGCAAAATGTTGAGAAATATTAAGTAGTAAGAACCTCTGGTTATAGCCTTGCTTCATTGGGAT
3e-132
ATAAACCAGTTCATTCTATGTATTCAAAGTCTATAGAGTAGAAGAAATACTAAATTCTAATAATATTGCTGCATTAGATTTGCATCTAT

CTAATTCCCCAATATTTCTTTGAGAAATCT
