# Supplementary material for: Chromothripsis during telomere crisis is independent of NHEJ, and consistent with a replicative origin
Source: Genome Res. 2019 May;29(5):737–49. doi: 10.1101/gr.240705.118 (PMC6499312; doi:10.1101/gr.240705.118)
Supplement: Supplemental Material [file supp_gr.240705.118_Supplemental_file_1.zip › contigs/annotated_contigs/DB106/contig.2.DB106_length_478_mean_cov_5.96234309623.docx]

**DB106_length_478_mean_cov_5.96234309623**

TTGTTTGCACTGTTTGTATCTGTTACCCGTGGAGGGTCTGCTTCTACTGTTGTAATTGCTTGTGTTGTGACTTTGCATCATAGCTGGAG
 >chr3:187880909-187881181 + E=3e-152
ATCCCTCTGTAGTTCAAATGGAAAGCTGGTGTTGAAGGTGAAACCAGGTCTTCTTCAGAAACTTGTGTGGGAACCTTTCATGGGCTGGA

TAAGCATGAGGATCAGGACAGGGCAGGGTCACTGACCTTAAAGGAGGATGCTTTGACCTGCCTCCAGCATGCACCCTTCCTGCTGAAGC

CATT|G|CTCCACCGAGTTCGAGCTTCCTGGCCGCTTTGTTTACCTAATCAAACAACTAACTGGGCAATGGCAGGCGCCCCTCCCCCAG
 >chr3:188096135-188096342 + E=5e-107
CCTCACTGCCGTCTTGTTGTTTGATCTCGGACCGCTGTGCTAGCAATGAGTGAGACTCCATGGGCTTAGGACCCTCCGAGCCAGGTGGG

GGATATAATCTCCTGATGTGCCATTTTTAAAGCCC
