# Supplementary material for: Chromothripsis during telomere crisis is independent of NHEJ, and consistent with a replicative origin
Source: Genome Res. 2019 May;29(5):737–49. doi: 10.1101/gr.240705.118 (PMC6499312; doi:10.1101/gr.240705.118)
Supplement: Supplemental Material [file supp_gr.240705.118_Supplemental_file_1.zip › contigs/annotated_contigs/DB106/contig.2.DB106_length_497_mean_cov_6.33802816901.docx]

**DB106_length_497_mean_cov_6.33802816901**

TAAATAAGACTGCTGATGGGAGGGTTTTTTTTAACCTCTGGTAAAATGATTTTTAAAGTGGATTTATGGAAAAAGTAGCAAACACATCA
 >chr20:14707387-14707613 - E=2e-121
TGTGTGTGATTTTTTCCAAGTAATTCATATAGTCAGGGATAAGATCACAGCCTATTACTGCCACATAAGCTGTGTAACAGCTGTGTAAG

AGTCATCTGAGGAGAACAGAGCTGGAGGCAGGTGCCTGGGTTTGAA|TC|TGATCCTTCATTTATTCACATTAATTCAGCACATATTTA
 >chr20:14669237-14669510 - E=1e-149
GGAGACACTATGATTGATACTGACATTGGAACTGACTGAGATAGACAAATCTTGCCTATGTGAAGCTGACTACTTGGTGGAATGATGAT

AGCATGAATAGAAATGTCCCATATGTCAACAAGCTTCTAAAAATCTATAAAAATTTAAATTGCATTATGAATTCAAAAATATCTAATAT

TGTAGAGAAGATTCCCAAAAGATCCAATTACATAAACAATCCTCCATCTACTTT
