# Supplementary material for: Chromothripsis during telomere crisis is independent of NHEJ, and consistent with a replicative origin
Source: Genome Res. 2019 May;29(5):737–49. doi: 10.1101/gr.240705.118 (PMC6499312; doi:10.1101/gr.240705.118)
Supplement: Supplemental Material [file supp_gr.240705.118_Supplemental_file_1.zip › contigs/annotated_contigs/DB106/contig.2.DB106_length_519_mean_cov_5.20231213873.docx]

**DB106_length_519_mean_cov_5.20231213873**

CTGGGCATGATGGCATATGCCTGTAGTCTCAACTACTTAGGAAGCTGAGTCAGGAGGATTGTTTGAGCCTAAAAATTTGAGGCTATGGA
 >chr5:104007541-104007745 + E=4e-111 p=7e-03
GAGCTATGATGGGGCCACTGCACTGCAGCCTGGGCAACAGACAGAGATTCTGTCTCCAAAATAAAAACAACAACAACAAAAACAAATGG

GTATGCCATGTATCCCTGTTTTATTT|ATTT|ACAGAAATGTTTTATACCTGGATTACACTGTGGGTTAGATAGGGGAATATGCTTGTC
 >chr5:104193785-104194096 + E=1e-175
AAAATTTATTAAACTGTAAAATTAACTGTATTGAAGATACTCATCAACCAATAAGAAATAAAAAATTAGAGAAATTATGATTCTATATA

GCAATTAAATGGAATATTAAAATTTTGAAATAAAAAGACTAAAATATTTGCCTTGTTTCCTAAAAAAACTGAAGATTATTCAAACATTT

GAATAGAAGTTCATGTGTTAAAAATTTGAGAATAATTCTTGAGGTTTGAAAGAGTTGATAACTGATTCTTTTTCAT
