# Supplementary material for: Chromothripsis during telomere crisis is independent of NHEJ, and consistent with a replicative origin
Source: Genome Res. 2019 May;29(5):737–49. doi: 10.1101/gr.240705.118 (PMC6499312; doi:10.1101/gr.240705.118)
Supplement: Supplemental Material [file supp_gr.240705.118_Supplemental_file_1.zip › contigs/annotated_contigs/DB106/contig.2.DB106_length_532_mean_cov_5.18609022556.docx]

**DB106_length_532_mean_cov_5.18609022556**

ATCTACCTAGGGAGGTTAAGAATAATTTGTTTCAGAATGGTATTGCTTAAAGAGATTCCCAGCCTCTTTTAGTTTATGGCTTCCATTGA
 >chr3:114278184-114278516 - E=3e-188
TAATATGATGAAAGCTATGGACCCTCTTCCCAAAAGAGGGTGCAATACCTTTTCAAAGGATTCACAAACCCCCTCAAGCCTATTGGTGG

ACCTCAGATTAATTATACCTGGTTCACAATTGTGTTCTAAGTAAATCACTGTAGGACTGACTGGGCTAACAAGATTTCAGACCAGAATT

GTGCAATACGTATTTACCATATGATCTTGGACCAGTCATTAATGTAAATTGAGGTCCTGGCTTTT|TTT|AGACTCCGTCTAAGAAAAA
 >chr3:114442548-11
AAAAAGAAAAGAAGGAAAGAAACCCAAGTATATCTTTTTCTATGCAAAGGCTACTGGAATAAGCTTTTTATTATTTTATTGAGAGATCT
4442745 - E=6e-107
CTCACAATAATGTGAGGCTAATTTCTCTGAACAATGGCTTGTTTTAATAAATATTTTGCAAATACAGCTAATACACTGTGTGGCTGACT
