# Supplementary material for: Chromothripsis during telomere crisis is independent of NHEJ, and consistent with a replicative origin
Source: Genome Res. 2019 May;29(5):737–49. doi: 10.1101/gr.240705.118 (PMC6499312; doi:10.1101/gr.240705.118)
Supplement: Supplemental Material [file supp_gr.240705.118_Supplemental_file_1.zip › contigs/annotated_contigs/DB106/contig.2.DB106_length_561_mean_cov_8.41711229947.docx]

**DB106_length_561_mean_cov_8.41711229947**

TGGGTTTTCTTTAAATAGGGGCTCTCGATGTGGACAGC|GCTGGTCTGGAACTCCTGACTTCATATGATCCTCTTGCCTCAGGCTCCTG
 >chr17:56099357-56099730 + E=7e-204 p=0e+00
ATTCACTGGGATTACAAGTGTAAGCCACTAAACCCAGTAGTTTTTCTTAAGTCTTCAAACTCAACTTACAAATCTTGATAATTATTAAT

CTAATCCTATGTTATATAAGAAATAAGATTCATTATTTTTACTTGTTCTGTGATTAATATTCAACTAATACTTATTTTAAAAAATACTT

ATATGTAAAAAATTAAATCCCGGCCAGGCCACGGTGGCTCAC|GCCTGTAATCCCAGCACTCTGGGAGGCGGAAGCGGGTGGATCACCT
 >chr17:56107101-56107354 + E=2e-113
GAAATCAGGAGTTCAAGACCAGCCTGACCAACATGGTGAAACCCTGTCTCTACTAAA|AAATATAAAAATTAACCAGGTGTGGTGGTGG

GTGCCTGTAGTCCCAGCTACTTGGGAGGCTGAGGCAGGGAGAATTGCTTGAACCAGGAGGCGGAGGTTGCAGTGAGCCGAGATCGCGCC

ACTGCACTCCAGCCTGGGTGACAGAGCGAC
