# Supplementary material for: Chromothripsis during telomere crisis is independent of NHEJ, and consistent with a replicative origin
Source: Genome Res. 2019 May;29(5):737–49. doi: 10.1101/gr.240705.118 (PMC6499312; doi:10.1101/gr.240705.118)
Supplement: Supplemental Material [file supp_gr.240705.118_Supplemental_file_1.zip › contigs/annotated_contigs/DB106/contig.2.DB106_length_588_mean_cov_9.91156462585.docx]

**DB106_length_588_mean_cov_9.91156462585**

TTAAAGATGAGATAAGTGAAGTTTGAAGACATTGGGTAACTTGTTTAGGATTCTTGCCCATGTAGCTGACTGTAGAGACTGTGGCATCA
 >chr3:24174020-24174362 + E=4e-194
GCCACCACAGGGCAGGCCTGATAAAGTTCAAGTGTCCCAAAGGGAAAGTCGAAGAACTCCGGTGGGACAGCTCTACAGGGTGCCCTGAA

GATGGGACAAGCAAGAGTGAGCTTATAGGAAAAGACGAAAGATTTGTGGGGATATTAAGAGCCTGGTTCTATGAGGGGCATTTAACTTA

TTCTTGAGGATTAAGAAACGACCACACAGTCATCTCACTGGGATATGCACTGAGAAGAATACAGACCTAGGCT|CT|GAGAGGCTGCTC
 >chr3:2418351
CTCCAAAGAGCTGGGATCTTTCAGAGGCTTTTTGGGTTTGTGCAGGTGCCAAGATTGCATAGGGTAGTGGATAGAGACATAAATCCCGG
2-24183760 + E=1e-137
AGCTGGCTGCATCCATCACCACTGTTTACCAGTTGTGGGAGCTCAGGCAAGTTTCCTCAACTTCTGTGTCTTACTTCTCTCTTCTGTAA

AATGGAGATATTAACCACATCTACCTACAGGATTGGTATAGGAAGTTAATGAATGA
