# Supplementary material for: Chromothripsis during telomere crisis is independent of NHEJ, and consistent with a replicative origin
Source: Genome Res. 2019 May;29(5):737–49. doi: 10.1101/gr.240705.118 (PMC6499312; doi:10.1101/gr.240705.118)
Supplement: Supplemental Material [file supp_gr.240705.118_Supplemental_file_1.zip › contigs/annotated_contigs/DB106/contig.2.DB106_length_591_mean_cov_7.86125211506.docx]

**DB106_length_591_mean_cov_7.86125211506**

ATCT|TTCATTTAGCATTAGGTATATCTCCTAATGCTATCCCTCCCCTCTCCCCCAACCCCACAACAGGCCCCACTGTGTGATGTTCCC
 >chr8:133644002-133644167 - E=2e-65
CATCCTGTGTCCGTGTGTTCTCATTGTTCAATTCCCACCTATGAGTGAGAACATGCGGTGTTTGGTTTTTTTGTCCTTGCAA|AAAACC

T|TGATGGTTTCTAGTTTCATCCATGTCCCTGCAAAGGACATGAACTCATCATTTTTTATGGCTGCATAGTATTCCATGGTGTATATGT
 >chr1:83134234-83134648 + E=2e-222
GCCACATTTTCTTAATCCAGTCTATCATTGTTGGACATTTGGGTTGTTTCCAAGTCTTTGCTATTGTGAATAGTGCCACTATAAACATA

CGGGTGTATGTGTCTTTATAGCAGCATGATTTATAATCCTTTGGGTATATACCCAGTAGTGGGATGGCTGGGTCAAATGGTATTTCTAG

TTCTAGATCCCTGAGGAATCGCCACACTGACTTCCACAATGGTTGAACCAGTTGACAGTCCCACTAACAGTGTAAAAGTGTTCCTATTT

CTCCACATCCTCTCCAGCACCTGTTGTTTCCTGACTTTTTAATGATCGCCATTCTAACTG
