# Supplementary material for: Chromothripsis during telomere crisis is independent of NHEJ, and consistent with a replicative origin
Source: Genome Res. 2019 May;29(5):737–49. doi: 10.1101/gr.240705.118 (PMC6499312; doi:10.1101/gr.240705.118)
Supplement: Supplemental Material [file supp_gr.240705.118_Supplemental_file_1.zip › contigs/annotated_contigs/DB106/contig.2.DB106_length_675_mean_cov_8.66666666667.docx]

**DB106_length_675_mean_cov_8.66666666667**

CCCAGCCCTTAGAGATAGCTCCCATAAACTTTTCACCGAGTTCTATCTCTGCTATTCTCTCATTGCTAAAGGGACAAGGTGCTTCTTTT
 >chr13:98359146-98359484 - E=1e-191
AAATATGCTGTCTATGTGATTTCTCATGCAGTCCAACACTCTCTGCTTCCTAGTTCTGGGGAAGCTCTTGATGCTAGTTTGTGCACTCA

TCCTACTGCTGCAAATGAATGATTTTTATTTTGCCCTTGTCTCTCACTGCCAAGAAATATACTTTGCTGGCTCATGCATGATCCAGGGT

CACAGTCTTTTTCCCCCCTTCACTTTCTGCCACGTATATCTCCACTTGATCTCTGCTTTTGGCGGCC|TTTC|TCCATAGAACTTACAC
 >chr13:98153157-981
CCTTTAATGTACTACATTTTACTTATTTTAAGGAAAAATTTTGCCTCTATTCCTCTCTCTATTACACACACGTGAGAATATAAGTTCAT
53498 - E=2e-193
GAGGGCAGGAATTTTATTTTTGTCTTTTTGTTTACTATTGTATCTCCTATACCTGAAAGATTCCTTGACACAGAATAAGCACCTAATAA

ATATTTTTTGAATGAATGAATGCCTCAGAAGATGGTGTTTTCTCAGGTGAATATTTGACTTTCTCTCCCAATCCACCCACCTAGAAGCT

AGATCTCTTGAATGGCTTTGAGGTCTCACTCCTTTTATAGCAAAGGCTGAGAAA
