# Supplementary material for: Chromothripsis during telomere crisis is independent of NHEJ, and consistent with a replicative origin
Source: Genome Res. 2019 May;29(5):737–49. doi: 10.1101/gr.240705.118 (PMC6499312; doi:10.1101/gr.240705.118)
Supplement: Supplemental Material [file supp_gr.240705.118_Supplemental_file_1.zip › contigs/annotated_contigs/DB106/contig.2.DB106_length_700_mean_cov_13.9828571429.docx]

**DB106_length_700_mean_cov_13.9828571429**

CTCCCTCCCCTAGCACACCTAACACACCCCCCAAAATGAGGAAAACAAGAATTGTGCACCATCTTTAGAATGGTGCTGTAGCTGTCCCT
 >chr20:41816815-41817126 + E=3e-166
CTTCTCTCACTTGCTAATCCAACCTTTCAGCTTCACACACCTCAAAAGGAGTGTCTTCGTCACCCAACTTCCCAGCCAGGGAAACAGGC

TGTCCCTTCACAGAAGTCGCCTATTCCGCTTCCATTGTAGTACCAAGCAAGGAAGGCTCTAAAGCATTTCCAACGGAATGCCCCTCCAG

CGGCCCTTAACCTCAGACGCAGCTTGTGCAAAGACGCTGTACCT|G|AAAGAATGGGTTTCAAATGAACAGTAATACACACAATTGCTT
 >chr20:41753928-41754316 - E=1e-221
GAACATTTTTTCATAGTGAAAATAATGCTAGCATTTATCACATCAAAATTAAAATACAAGTTTTGCTCAAGCCAAGTCTAGATTTAAGC

AAGTTGGATGCTTATAGATGCTAACGTCCAGTCTAGCTATAGAGCATTTCTTTAGTCTTAGCTTTATTTTCTGAGTTTGAGAGTCTCTT

ACATGGGGGGAGCTTATTAACAGGGATTTTTCTATTGTGCAGTCAGTGAGAAATGAGGTCAAAAGTGCTTGTGTACAGGTATTCATTCA

TTCAGTGTGGGTGGTAGAAAGAGGGTGGAATTTGGCACCAGGAAGGTCAGAGTTCAAATCCCAAATCTGGCCCAGCACG
