# Supplementary material for: Chromothripsis during telomere crisis is independent of NHEJ, and consistent with a replicative origin
Source: Genome Res. 2019 May;29(5):737–49. doi: 10.1101/gr.240705.118 (PMC6499312; doi:10.1101/gr.240705.118)
Supplement: Supplemental Material [file supp_gr.240705.118_Supplemental_file_1.zip › contigs/annotated_contigs/DB106/contig.2.DB106_length_701_mean_cov_13.0527817404.docx]

**DB106_length_701_mean_cov_13.0527817404**

AGGACAGCTATAGGGGCCAAGAGTAAACCAGATATCCATTATCTTTATGTTTCTCAGTTCTGCTTTAAGAAAGAAATGTACCTAACCAA
 >chr4:169525413-169525713 + E=1e-168
AGGAAATGTCTCAATTTATCTATGAAACACACAACACCCAGAAAAATCTCATAAAATCTCATACATGGAACACGTAACATGGCCAGTTT

AACAATCGGCCAAACAATCTGCAAAAATGATATAACAGTGCCTGAATTTGAACATCTCTAGACATAACAAATAGAGCATAGTTACATTA

CCAGACTAACAACGCTTGATTAAGGCATAC|AGA|TGTTCAATCTCATTAAGTAATAAGAAAAATATGAATTAAAACAACAGAGATACC
 >chr4:169721656-169722060 + E=2e-231
ATTACACACCCATTTAATTGGCAAAAATTTAAGCCTGATAATACCAAGTTCTGGCAAGGATGTGAAGTAACAGGATATTTCATATACTG

CCAGTGGGAGTATAAATTGTTAAAAACCACGTTGGAAAAGAGTTTGGCATAATTTGATAAACTCAAAGTTATGCAAACTCTATGACCTA

GCAAACTTCACTCTTGAGTATATTTCTCAAAGAAATGCTTACATACGTGCACCCAGAGACACGTAACCCAAATGTACATAGCAGCCCTG

CTCATAATAACCCCAAACTGGAAATATTCCAGAAGTCCATCAACAGTAGAACAAGTAAGGAAATTGTGGTAGAGTAATTC
