# Supplementary material for: Chromothripsis during telomere crisis is independent of NHEJ, and consistent with a replicative origin
Source: Genome Res. 2019 May;29(5):737–49. doi: 10.1101/gr.240705.118 (PMC6499312; doi:10.1101/gr.240705.118)
Supplement: Supplemental Material [file supp_gr.240705.118_Supplemental_file_1.zip › contigs/annotated_contigs/DB106/contig.3.DB106_length_528_mean_cov_7.10227272727.docx]

**DB106_length_528_mean_cov_7.10227272727**

GAGCCAGGCAGTGTTTTGTGAGTGTCTCCTAAGCTTCTGTTGAGAAGAAAAATAGCTCAGAGAAGTTTGAGCTACGCGAGGTGTGCAAA
 >chr10:116085908-116086203 + E=6e-166 p=1e-03
ATGTATCAAGCCCAGGAGTATGGGGCTTCAGGCACCGTTTTCTCCCCTCCCCATTCCTGGGGGCAACTGTTTAAAGGCATTTTACTCCT

CACTAGCTGCCTCATCCATTGTCTTCAGGTTCCTGTAATTTATGATACAAAGAACAATGTTTAGCTGATCGATAGCTTACAGTATTTTA

ATATAAAATATTGGTAAACAACTTAAA|A|TATATAAAATATATTAAAATATATATATAAA|AC|TTAGATGGGAAAGGCAGGAAGGAT
 >chr4:108477610-108477642 - E=1e-04 >chr22:27211257-272
GGCAATAAGGAGACAGCCAGGTAAGAGGGCTACGGGCACTGACTTCTAGGCAGAGGGAACAACATGTGCAAAGGCTCTCAGGTTAAAGC
11443 + E=3e-100
AAGTTTGGCTTGTTCACAGACAAGAGTGAAAGCTAGTGGGGCAGGAGGTGTGTGACGGGTGAGGGAGGTGGGC|TATATATATATATA
