# Supplementary material for: Chromothripsis during telomere crisis is independent of NHEJ, and consistent with a replicative origin
Source: Genome Res. 2019 May;29(5):737–49. doi: 10.1101/gr.240705.118 (PMC6499312; doi:10.1101/gr.240705.118)
Supplement: Supplemental Material [file supp_gr.240705.118_Supplemental_file_1.zip › contigs/annotated_contigs/DB106/contig.3.DB106_length_765_mean_cov_44.2784313725.docx]

**DB106_length_765_mean_cov_44.2784313725**

GAGAAACACAATCTAAAAT|AAAAACAGAAAACTGTGCGGGCGCGGTGGCACATGCCT|ATCATCCCAGCTGCAAGGAAG|GCTGAGGC
 >chr8:33440007-33440045 - E=5e-02 >chrUn_
AGGAGAATCGCTTGAACCTGGGAGGCGGAGGTTGCAGTGAGCCGAGATCGCGCCACTGCAACCCAGCCTGGGCGACAGAGCGAGACTCC
gl000220:145413-146067 + E=0e+00 p=8e-03
GTCTCCAAAAAATGAAAATGAAAATGAAACGCAACAAAATAATTAAAAAGTGAGTTTCTGGGGAAAAAGAAGAAAAGAAAAAAGAAAAA

AACAACAAAACAGAACAACCCCACCGTGACATACACGTACGCCTCTCGCCTTTCGAGGCCTCAAACACGTTAGGAATTATGCGTGATTT

CTTTTTTTAACTTCATTTTATGTTATTATCGTGATTGATGTTTCGAGACGGAGTCTCGGAGGCCCGCCCTCCCTGGTTGCCCAGACAAC

CCCGGGAGACAGACCCTGGCTGGGCCCGATTGTTCTTCTCCTTGGTCAGGGGTTTCCTTGTCTTTCTTCGTGTCTTTAACCCGCGTGGA

CTCTTCCGCTCGGGTTTGACAGATGGCAGCTCCACTTTAGGCCTTGTTGTTGTTGGGGACTTTCCTGATTCTCCCCAGATGTAGTGAAA

GCAGGTAGATTTGCCTTGCCTGGACTTGCCTGGCCTTGCCTTTTCTTTCTTTCTTTCTTTATTACTTTCTCTTTTTCTTCTTCTTCTTC

TTCTTCTTCTTCTTCTT|CTTCTT|TTTTTTTGAGACAGAGTTTCACTCTTGTTGCCC >chr19:50809511-50809550 - E=1e-11
