# Supplementary material for: Chromothripsis during telomere crisis is independent of NHEJ, and consistent with a replicative origin
Source: Genome Res. 2019 May;29(5):737–49. doi: 10.1101/gr.240705.118 (PMC6499312; doi:10.1101/gr.240705.118)
Supplement: Supplemental Material [file supp_gr.240705.118_Supplemental_file_1.zip › contigs/annotated_contigs/DB106/contig.4.DB106_length_368_mean_cov_9.71467391304.docx]

**DB106_length_368_mean_cov_9.71467391304**

CAATGCAATGGACCTCACCCTGCCAAGGCGTGGCAATGAGCTGGAAGAGCCTATACTG|AT|ATATATATATATATATATATATATATA
 >chr12:76355812-76355872 + E=1e-24 >chr13:46396654-46396691 - E
TATATA|TT|GCCCACTCATGGATGCAAAAGCAGTGATCAAATACTAC|**ATATATATATATATATATATATATA**|AACTTAATTCATAA
=9e-11 p=0e+00 >chr2:137560227-137560292 - E=1e-27 p=0e+00 >chr2:137559999-13756023
ATTATTTTTAAAGGCATTATAATCAATAGTACACATGCAGGGATACCTGAAGGACCATAACACCAGGTTATCACAAAGCTGTACAGCAT
4 - E=4e-130
TTCAATTGTTGAATATTTTGTTATCCTCTCAGGAGGGCCAACTTGGGGAGAAGCACACACTTTTTATTCCTGCTGTGTACATTTCTGAA

ATCAAGAGAAGCTATCTT
