# Supplementary material for: Chromothripsis during telomere crisis is independent of NHEJ, and consistent with a replicative origin
Source: Genome Res. 2019 May;29(5):737–49. doi: 10.1101/gr.240705.118 (PMC6499312; doi:10.1101/gr.240705.118)
Supplement: Supplemental Material [file supp_gr.240705.118_Supplemental_file_1.zip › contigs/annotated_contigs/DB107/contig.2.DB107_length_344_mean_cov_10.8895348837.docx]

**DB107_length_344_mean_cov_10.8895348837**

TGAGCTCTTTACTGGTGTGTATAGTAGAAAAGAAGAATGGCTTACTGGGCTCTCAGTAAATTGTTCCATAAAAGAAGACAGCAAATAAG
 >chr12:97329524-97329843 - E=2e-181
TCCTAACAGTAAAAATTCTAGCATCATAATAAAGGCTGACAGTGGGACCTTCCCTTGTAGTAACATTCATACTCCACATGATCTACAGA

CAGTGGTAAGCATCTTGAATACAACTGTATCACTCTTGCATTCTTTTGTTTTTGTTTTTGTTTTTTAGCTAACTAAGCATACCTAAATT

TAACAAGAAGAGGGTACAGTGGGCCCAAATGCTTAGAGGATTATGTACAC|CA|TATATATATATATATATATTTTTTT >chr2:140526443-140526470
