# Supplementary material for: Chromothripsis during telomere crisis is independent of NHEJ, and consistent with a replicative origin
Source: Genome Res. 2019 May;29(5):737–49. doi: 10.1101/gr.240705.118 (PMC6499312; doi:10.1101/gr.240705.118)
Supplement: Supplemental Material [file supp_gr.240705.118_Supplemental_file_1.zip › contigs/annotated_contigs/DB107/contig.2.DB107_length_365_mean_cov_4.93150684932.docx]

**DB107_length_365_mean_cov_4.93150684932**

CATTCATTCTAATGTTGTCTGTCATGTTTCATTCAAGTATTCTCTAAGCTGCTGCTGTGAACCTGACGCTGCTGAGCACAGGGACACAG
 >chr8:140869096-140869315 + E=2e-117
AGCTGACTCGGGCACACACCTCTGCTCCCGGGAGCACTGAGGTCTGAGAGAGGAAGCAGGGTGATAAGGAGCAACGGGACAGTCGGGAC

GCCCTGGGAATCTGGGCGGGGGGAGCTGGTTCAGACAGAGG|GGGGTGCCTGCCCTGCATTT|TGAAAGAGCAGAAAGGTGCCCAGGGC
 >chr8:140869315-140869441
ACTCCAGGCAGAAGGAAAGCATGTGTGAAGGCAGGGGAGTGTGAGAGTCCATGGTTTCCAGCAAGTGCACAGGGATCAGCACAGCAGAG
 + E=2e-64
GAGGGAGCGGT
