# Supplementary material for: Chromothripsis during telomere crisis is independent of NHEJ, and consistent with a replicative origin
Source: Genome Res. 2019 May;29(5):737–49. doi: 10.1101/gr.240705.118 (PMC6499312; doi:10.1101/gr.240705.118)
Supplement: Supplemental Material [file supp_gr.240705.118_Supplemental_file_1.zip › contigs/annotated_contigs/DB107/contig.2.DB107_length_369_mean_cov_10.2764227642.docx]

**DB107_length_369_mean_cov_10.2764227642**

AACCCTGCAGACTGGCGGCACTGCCAAACCACGGGCTCCAACCGAACTGTTATTTGCATAGAAAACCTAAAAGAATCAGCCAGGCGCAG
 >chr1:44384819-44385127 - E=2e-174 p=2e-02
TGGCTCATGCCTGTAATCCCAGCACTTTGGGAGGCCAAGGCGGGTGGATCACCTGAGGTTAGGAGTTCAAGACCAGCCTGGCCAACATG

GCAAAACTCCATCTCTACTAAAAATACAAAAAATTAGCCAGGCATGGTGTCAGTTGCCTGTAATCTCAGCTACTTGGGAGGCTGAGGCA

GGAGAATTGCTTGAACCTGGGAGGTGGAGGTTGCAGTGAGC|TGAGATCAT|GCCACTGGACTCCAGCCTGGAGTGGTT|TGGGAGATA
 >chrY:21739325-21739352 - E=9e-02
CAGGCAGCCCATCGTA
