# Supplementary material for: Chromothripsis during telomere crisis is independent of NHEJ, and consistent with a replicative origin
Source: Genome Res. 2019 May;29(5):737–49. doi: 10.1101/gr.240705.118 (PMC6499312; doi:10.1101/gr.240705.118)
Supplement: Supplemental Material [file supp_gr.240705.118_Supplemental_file_1.zip › contigs/annotated_contigs/DB107/contig.2.DB107_length_434_mean_cov_5.29493087558.docx]

**DB107_length_434_mean_cov_5.29493087558**

ATCCTCTTCCGCAGAAGGAGTTAGTAAAGATGTGCCTCAACTTACAATGAATGGGGTTATGCCCCACTAATCCCATCATAAGTTGAAAA
 >chr3:122184037-122184245 + E=1e-113
TATTAAGTAAAAAGTACACTTAATACACCTAACCTACCAAACTTCATTGCCTAGCCTAGCCTACCTTAAACATGCTCAGAACACATACA

TTAGCCTACAGCTGAACAAATGATCTAACA|GATCTAA|CAGAACTCAAAGAAAGAAAATCACTGCAAATCTCATGAACATTAAAAGGA
 >chr3:122191575-122191794 + E=3e-108
CAATAAAATAATACTGGCCAGGCACAGTATACTGGCTCACACCTGTAATCCCAGCACTTTGGGAGGCCGAGGCAGGCAGATCACTTGAG

ATCAGGAGTTCAAGACCAGCCTGGCCAACGCGGCAAAACCCCGTTTCCACTAAAAATATGAAAATTAGCCAGGTGTGGTG
