# Supplementary material for: Chromothripsis during telomere crisis is independent of NHEJ, and consistent with a replicative origin
Source: Genome Res. 2019 May;29(5):737–49. doi: 10.1101/gr.240705.118 (PMC6499312; doi:10.1101/gr.240705.118)
Supplement: Supplemental Material [file supp_gr.240705.118_Supplemental_file_1.zip › contigs/annotated_contigs/DB107/contig.2.DB107_length_460_mean_cov_7.36086956522.docx]

**DB107_length_460_mean_cov_7.36086956522**

CTTA|CCTTAGAGTAATAATTTTAAATGTTATGATATATCCTTCTGTTGTTTATTATAAGCATATATATATGTATTCTTAGGGAATATT
 >chr6:27820653-27820963 + E=4e-169 p=2e-02
AACTTCATTTTGCTGTTGATGTCTAGGGATTGGACTTTTTAATATTTGTTTCTACCGTTACATAAAATATTTTTACATAGTCCCAAGGT

CAAATCAAATTATATCCAAAGAAATCTTTCTCCTTCATCCTAATAGCCTCTCTTACCTTAGAGTAATAATTTTAAATGTTATGATATAT

CCTTCTGTTGTTTATTATAAGCATATATATATGTATTCTAGGGAAGA|T|AGGCAATTCATCTTGACTATATTTTTGTCACCAAAGAGC
 >chr6:27822247-27822394 + E=7e-77
TTAATATTGAACCATCAAAATATATTCACTTTACCATCTGGGACATTTTAATCAAACCTCTCATCTGTTCAATCACATCTTTCACTTCA

GAAAGAACTTTCACTTCT
