# Supplementary material for: Chromothripsis during telomere crisis is independent of NHEJ, and consistent with a replicative origin
Source: Genome Res. 2019 May;29(5):737–49. doi: 10.1101/gr.240705.118 (PMC6499312; doi:10.1101/gr.240705.118)
Supplement: Supplemental Material [file supp_gr.240705.118_Supplemental_file_1.zip › contigs/annotated_contigs/DB107/contig.2.DB107_length_468_mean_cov_6.41025641026.docx]

**DB107_length_468_mean_cov_6.41025641026**

ATGGAAACCAACAAGTGGTTATATGCAACACAAAATGTCACCTAGGATGCCATGCTCTGCCCTCTAGGGAAAAACAAAGGGAACGAGGC
 >chr2:191860375-191860592 + E=5e-116 p=0e+00
TTGTGCCAGCAGTAGAAGCTTTGTTATTTGAGGAGTACAACTAACATGCACTGAATGATTACAGTCTACATCAAAAACTTCTACACACC

ACTGAGGCCTATGTGAGGAGAGATGGGTGGAGGCAGGGC|AAGAAACTAGTCTGAA|CTGGCAAGAAACTAGTCTGAACTGGTTCTTTT
 >chr2:191792235-191792470 + E=7
AAAAATGCAGTTTGAACTTTGCTTCTAAAGCTGTATATTAATTTTTGAGAGAGAGGCTTGTTTTCAAGGTTAATTTGTTACAAGATTAG
e-130
TAAGGAATAGAGATAAGGTCTACCAGATGTTCTTAAGAACTTGGAAGTTTGATTTATAGAACCTCATCCCTTCTGCTTTTAAGGCAATC

CAGTGTACAAGGTTAGTACAGTCTT
