# Supplementary material for: Chromothripsis during telomere crisis is independent of NHEJ, and consistent with a replicative origin
Source: Genome Res. 2019 May;29(5):737–49. doi: 10.1101/gr.240705.118 (PMC6499312; doi:10.1101/gr.240705.118)
Supplement: Supplemental Material [file supp_gr.240705.118_Supplemental_file_1.zip › contigs/annotated_contigs/DB107/contig.2.DB107_length_486_mean_cov_4.93827160494.docx]

**DB107_length_486_mean_cov_4.93827160494**

CATTGTAGGGTATGGGGAGGGGGAAAGGAGCTGCATGGGGTACAGTGGTGGCCCAGGCTGGCAGTCAATAGAGGCACAAACTCTCGCCT
 >chr8:140857479-140857751 + E=4e-152
GTGCTCCACTGCATTCCTGGCCCAGGCCTTTATAAACATATACGGGTTCCATAAAAGCGTACGACATTAAAACGCACTGGAGTGCCAGA

TGGCTAATAGCTTCCTAATATCCACTTTCTCCCTCCAAAAGAATGGAACCCCAATATTTAGCTGGGCATATTAATGGAGCCCGTACTTC

CA|GGC|ACCATACGGAGCTCCTGTGGCTGCTCCGGCTGTTGTGAGGCTGCTCTGTGGCCACCATGCCTTCCTCGGCCAGTGGCAGCCG
 >chr8:140869656-140869873 - E=5e-119
TGAGAGTGAACGGGCCTTGGACTTGGTGTCAGCAGGCCTGGACTGGGGTCCACGTTGCCCCATGTGTTTATTGTGTGACCCTCGGCTTC

CCCAGCTGTCAAGTGGAGATAAAATGGGGACTGTCGCCTTGGG
