# Supplementary material for: Chromothripsis during telomere crisis is independent of NHEJ, and consistent with a replicative origin
Source: Genome Res. 2019 May;29(5):737–49. doi: 10.1101/gr.240705.118 (PMC6499312; doi:10.1101/gr.240705.118)
Supplement: Supplemental Material [file supp_gr.240705.118_Supplemental_file_1.zip › contigs/annotated_contigs/DB107/contig.2.DB107_length_503_mean_cov_7.45526838966.docx]

**DB107_length_503_mean_cov_7.45526838966**

GATGGCTCGATGGTTGGGAGACAAGTGCTAGAGATGGGGACAACTGCCTCTTCGGTGACCTGTCAATAACAGCTGAAGGCAGCAGGGTC
 >chr8:140869925-140870174 - E=3e-135
CCTGGCCAGGCCACCTGGGTCCATTGCACCTTTTTTGGGGCTAGTTGCTCTCTCCACCTGCTGCTGGCTGTGTGCAGCTGTTGGCAGGC

AGCCCAGGGCACCCAGCCTCGTGCTTCCTGGTGAGCAAGTTGCTTTACAAGCAGTGGGCCGCCCAGGCA|GG|CTGCTGTTTTCCTATC
 >chr8:140858605-1
CATGGCCCACGACCAGGCCCCACCAGTTGGCACCAGCCTGTGAAACGCAGACTCATCTCTCCGAACTGACTGCCTCTGGCAGGACGAGG
40858861 + E=2e-142
TGAATTATCCTAAATCAAGGGGAGGCAGTGAATACATTATCTCAGGAATAAGAAGAAAAATGAAGATGGATATTTACAAACAACTCCTA

AAGAAATTCACCCCCACACACATATAATTTTAATGGTCTCTGAAAGAAAAGGCAACTCTT
