# Supplementary material for: Chromothripsis during telomere crisis is independent of NHEJ, and consistent with a replicative origin
Source: Genome Res. 2019 May;29(5):737–49. doi: 10.1101/gr.240705.118 (PMC6499312; doi:10.1101/gr.240705.118)
Supplement: Supplemental Material [file supp_gr.240705.118_Supplemental_file_1.zip › contigs/annotated_contigs/DB107/contig.2.DB107_length_511_mean_cov_7.63209393346.docx]

**DB107_length_511_mean_cov_7.63209393346**

CATCCCCAGCACACATTGGGCCAGACAAGGTGCTGCCCCTTGTCTGATTATTTTGTGTCATCACATCAAGTTTTCAAAGTAGGAATTTT
 >chr1:44502459-44502738 + E=2e-156
CTGTGTTTTCTAGAATGAGAGCTCAGGCAGGTTAACTAATTTGCTGCAGGTTATAGAACTAATAAGTGGTAGAACGTGAACTTGGATTC

AGGTTGGCCTGTGTCCAAAACGTGAACTTGAATTCAGGTTGGCCTGTGTCCAAAAGCTATTATTTCACCCCATGCACAATATACTAGGG

GGGATGTGCTGG|AAAAGTTGT|GAGTTGGAAAAGGATAGAGTTTATGGAGAGTAGAGAATGTTTGGAATAGCTGCTGAGCAAAGTTGA
 >chr1:44352778-44353001 + E=1e-122
GGGCCCAGCGCCTCATAAATTTGTGGTGGCATCAGTGCACTTTAGTGTATATGTGTGTGTGTTCTCGCAGCCCCTCAAGTATAAAAGGT

ACAACAGTGTAAGATTAGATAAATCTCTGGTTGGATTTTGCCTAATGTGTGGGACAAAAGAACAGTGG
