# Supplementary material for: Chromothripsis during telomere crisis is independent of NHEJ, and consistent with a replicative origin
Source: Genome Res. 2019 May;29(5):737–49. doi: 10.1101/gr.240705.118 (PMC6499312; doi:10.1101/gr.240705.118)
Supplement: Supplemental Material [file supp_gr.240705.118_Supplemental_file_1.zip › contigs/annotated_contigs/DB107/contig.2.DB107_length_562_mean_cov_7.20640569395.docx]

**DB107_length_562_mean_cov_7.20640569395**

AGAAAATTTGGATGCAGACAGGTACACAGGAAAAATGATGTGAAGACACAGGGAGAAGACCACTATCTACAAGTTAAGGAGAGTGGCTG
 >chr7:90426411-90426668 - E=5e-140 p=5e-02
GCACAGATCTTTCCTCATTGCCCTCATTTGGAGGGCCATGACACCTTACTCTCAGACTTCCAGCTTCCAAGACTGCAAGACATTAAGTT

TCTGTTGTTTAAGCTACCCAGGCTGTGGTACTTTTTTTCAGCAGCCCTAGCAAACTAACTGAGGTGGCTTATCTTATA|C|CAATACCC
 >chr7:90
TCTTTATATAACAAATATTTTACTACTTTTTTTGCCTTTACTACTATGAAATGGAATTCATATATAACAAGATACACATACATAATTTC
424606-90424912 - E=2e-172
AAATATTTATATATAAATATAATTTCAAATATATTAATTTATATCTATATGTAACTAATACAAAAAATAAATAAAAATTAATTTATAAT

AAAACAAAGTCTTTAAATATGTAAGTAGACACAACTATACCGGAACACACTGAAGAGTGGTGCTCACATCTATGTACAGAATCAGCATA

AGCACAACCACTACAAATGCAGACTGTGGG
