# Supplementary material for: Chromothripsis during telomere crisis is independent of NHEJ, and consistent with a replicative origin
Source: Genome Res. 2019 May;29(5):737–49. doi: 10.1101/gr.240705.118 (PMC6499312; doi:10.1101/gr.240705.118)
Supplement: Supplemental Material [file supp_gr.240705.118_Supplemental_file_1.zip › contigs/annotated_contigs/DB107/contig.2.DB107_length_563_mean_cov_6.91829484902.docx]

**DB107_length_563_mean_cov_6.91829484902**

TATATATTGTCTGTAACTACTTCTAAATAAGTTTTTGAAACCTTCCCCCCACCTTTAGTGAGTGGTATGTTTTTTATATTTAGAAACAA
 >chr12:97325795-97326266 + E=8e-265 p=0e+00
TCTCCCTCCTCCACAAACATGCAACATTCTTACACAATAATGTGGTATAAGAAAAAATAACATTGCTGCTTATACTGCCGGGATTTTTT

CCCCCCCATAAGCTTCTTGATTTAATTCTTTTCTTATAAATAATTTAGTCTGGAACAAGATAGGGAGTTAATGACTTAAGTGCTGCTGA

TACTGAAATAAGTTAATATTTATTTTAAATATAATTTGGAGGCCAGGTGCGGTGGCTCACACCTGTAATCCCAGCACTTTGGGAAGCTG

AGGCAGGTGGAGTGCTTGAGTCCAGGAGTTTGAGACCAGCCTGGGCAAAATGGCAAAATCCTGTCTCTTAAAAAAAAAAAAAAAAAAAA

ATATATATATATATATATATATATAT|A|TATATATATATATATATATATATAAAATGCACACACACAAGTAAATATATTTATATATAT
 >chr12:97326241-97326333 + E=1e-43
AAATTATATATATATAATTTAGTAAATCAGT
