# Supplementary material for: Chromothripsis during telomere crisis is independent of NHEJ, and consistent with a replicative origin
Source: Genome Res. 2019 May;29(5):737–49. doi: 10.1101/gr.240705.118 (PMC6499312; doi:10.1101/gr.240705.118)
Supplement: Supplemental Material [file supp_gr.240705.118_Supplemental_file_1.zip › contigs/annotated_contigs/DB107/contig.2.DB107_length_602_mean_cov_7.47508305648.docx]

**DB107_length_602_mean_cov_7.47508305648**

TTATGAGCTTCCATTCTTGTTTCACACACACTTAGTTTATATGTAATTTAAATATATAAAAGACTATACAGTTAGAATTTATTTTACAT
 >chr8:64453893-64454235 + E=4e-194
AATTAAAATTATATATGAATATAAATATAATATATTTCTATATTTAAATATGTTTTGAAATATATTTTCCACCAGTTTTAAAGTTTTGT

GGGAAATTGTGGAGTGTACTTAATCCAGCAAGATTTAATGGAAATTTACTTTTTTGTATATTGGGACTTGTATATTATTTTGTGAGTTA

TGTACCTTACTTTCCTTTAGAAACAAGGTTCTTTATGCACATTCACATCTAAGTAATAGAGCAGACCATATGTGT|ACCAGAGTGGTAC
 >chr8:64464
ATTTGTTACCATTGGTAAACCTGGACAGACATATAATTATCATTCAGAGTCTATAATTTATATTAGGGTTCACTCTTGGTGTGCATGTT
018-64464278 + E=9e-145
CTATGGGTTTGGACAAATGTATAATGACATGTATTCGCCATTATAGCATCATACAGAACAGTTTAACAGCCCTAAAAATCTTCTGCTGT

CTGCCTGTTCACCCTTACCTTCCCCCATAGGGAAACAGGATTTTTTAAGTGGAAAGTTGAAAGTTATAA
