# Supplementary material for: Chromothripsis during telomere crisis is independent of NHEJ, and consistent with a replicative origin
Source: Genome Res. 2019 May;29(5):737–49. doi: 10.1101/gr.240705.118 (PMC6499312; doi:10.1101/gr.240705.118)
Supplement: Supplemental Material [file supp_gr.240705.118_Supplemental_file_1.zip › contigs/annotated_contigs/DB107/contig.2.DB107_length_607_mean_cov_8.64909390445.docx]

**DB107_length_607_mean_cov_8.64909390445**

TGAAGTTGGATAAAATCCGAAGTAAAATGCAAGAGAGGAGAAACTGTGAATTAAATGAGATTGTCCTGAATTGACTGAGAATAAGATTA
 >chr7:133957487-133957704 - E=7e-119
ATTCTTTCACTTTGGATTGCACTTAAAATTAAAAGTTTGTGGCCTGAAGTATTCATACTCACCACAGATGCATTACCTAGCTTAATCAT

CACAAGACTCTTAATAAGAAATTCTGTTAGTTTTATAAA|C|GGGGCAGTGGCTATAATGAAGACTGTGTTAGTCTATTCTTGCATTGC
 >chr7:134020061-134020450 - E=2e-213
TATAAAGAAATACATGAGGCTGGGTAATTTATAAAGAAAAGAGGTTTCTGGCCAGGCACAGTGGCTCACGCCTATAATCCCAGCACTTT

GGTAGGCTGATGCAGGTGGATCACTTGAGCTCAGGAGTTAGAGACCAGCCTGGCCAACATATTGAAACTTCATCTGAACTAAAAATATA

AAACATTAGCTGGGCATGGTGGCATATGCCTGTAAGTCCAGCTACCGAGGAGGCTGAAGCACGAGAATTGCTTGAACTCAGAAGGCAGA

GGTTGCAGCAAGCTGAGATTGTGCCACTGCACTCTAGCCTGGGTGACAAAGTGAGACTCTGTCTCAAAACAAAAC
