# Supplementary material for: Chromothripsis during telomere crisis is independent of NHEJ, and consistent with a replicative origin
Source: Genome Res. 2019 May;29(5):737–49. doi: 10.1101/gr.240705.118 (PMC6499312; doi:10.1101/gr.240705.118)
Supplement: Supplemental Material [file supp_gr.240705.118_Supplemental_file_1.zip › contigs/annotated_contigs/DB107/contig.2.DB107_length_674_mean_cov_11.5727002967.docx]

**DB107_length_674_mean_cov_11.5727002967**

AAAATATAGTCAACCTCATCTCTGGACTAAACTGGAAAATGTAAGAGACAAGTTAAGTAGTACTTATGGAACTGTAGGCTCATTAGAGA
 >chr14:67515067-67515473 + E=1e-229
AACCTGTAGCATCAATTTGTGCCCACTTTAGAATAGCTTTAATCCTACAAATCTACTTAAGAAAAGTAAACCCATTGCAGGGGAATGCT

GTCAGTTTAGATGTTATAATAATTCAGTCTGTCACTCTTAGCTCCTTAGCTCAGTTCAGACAGCTATAGTGGGGAAGATGTCTAGACTC

CCTCCCCTACCTCTGCCCCAACTCTCAAATAAAAATCCTATATCCTAAAGAACCTTGTAGTTTATTTTTACCAAACAAGCTAGTCTACC

TGCAAATTAACAGTAACAGTGATATCACCAGGTAATTTTAATATTGTAA|A|GGTCCATACTGATGTTTGTTCTTGGCTCTGTTCCATC
 >chr14:67567528-67567797 + E=4e-150
TCACAGCTGCTGATGGCCCAGGAGATCGTTTCATCATTGGGGAATCCCAAGCTGGTGAACAGGTGAGATTGATAGGCCTGAAAGGCACT

GAAGATTTCAGCTTCATGGACTTTGGGCATCTAATTCTAAATTTCTCCTATCAGTTTAGTGGAAATTAGAGAGAGATTAGTCAGAAGAT

TATCAAAGGGATGAAAGTATAGTTCCCAGGAGAACCAAAAGAATTATTTCTTA
