# Supplementary material for: Chromothripsis during telomere crisis is independent of NHEJ, and consistent with a replicative origin
Source: Genome Res. 2019 May;29(5):737–49. doi: 10.1101/gr.240705.118 (PMC6499312; doi:10.1101/gr.240705.118)
Supplement: Supplemental Material [file supp_gr.240705.118_Supplemental_file_1.zip › contigs/annotated_contigs/DB107/contig.2.DB107_length_791_mean_cov_6.97850821745.docx]

**DB107_length_791_mean_cov_6.97850821745**

TTAATAGAAAAATTATTCTCAGGATTCTAGAAAAACCCAGATGGTATTCAGATACTATACACGCTGTTTTAAAAACTAAAAAGTGACTC
 >chr8:126131325-126131695 - E=1e-210
ATTTACTGTTTCACAAAGTGACATCCTAATGTGAATGGGAGAGCTTAGCAAACAGGGGAACACAGCAAGTAATTATTTCCACACACGCC

ATCATGTGGAGAAACCATGCCAACTTTCAGGTTGTTACAGTCGGTGGAGGAAAGAAAATCTGGAGGTGTTGGAATGATTGGAAATTTAT

GGAGTGAGATTCCACGGTAACAGGAGGTGAGATGAGGTGAACAGCAGCCAAAACTTTCCTCTCAATAGCTCTTAAAAAGGAAACTACTC

AACTTTCTGAGTAA|TAA|TCCCGAATAGTTGGGATTACAGGCACACACGTCCACGCCCAGCTAATTTTTGTATTATTAGTAGAGACAG
 >chr8:126392764-126393182 - E=1e-221
GGTTTCACCATGTTGGCCAGGCTGGTCTCAAACCCCTGACCTCGTGATCCATACGCCTTAGCCTCCCAGAGTGCTGGATTACAGGCGTG

AGCCACTGCACCTGGCCCTAGACATATATTTTCAATAGCTGTTTTAATATTTTCATCTACTAATTCTGTCATCTATGTCTATTAATATT

ACACAAATCTTCCTTTTATTATGGATTATATTTTCCTGCTTATTTGCATAGCTGGAACTTAAAAAAAATTTTTAAATTAGGTTATTACT

TTTTTAGAGACAGGGGCTTGCTATGGTACCGATGCTAGAGTGCAGGGGAATGATCATAGGTCACTGCAGCCTAAAACTCCT
