# Supplementary material for: Chromothripsis during telomere crisis is independent of NHEJ, and consistent with a replicative origin
Source: Genome Res. 2019 May;29(5):737–49. doi: 10.1101/gr.240705.118 (PMC6499312; doi:10.1101/gr.240705.118)
Supplement: Supplemental Material [file supp_gr.240705.118_Supplemental_file_1.zip › contigs/annotated_contigs/DB107/contig.2.DB107_length_795_mean_cov_10.4691823899.docx]

**DB107_length_795_mean_cov_10.4691823899**

TAACATGTAATAGGAAAATTATTAATGAGATATTTTATGTTCTTTACATTGGACCAGGTTTTTTGAAATATCAAGTGTGTTTTACACTT
 >chr10:32214623-32214843 - E=2e-120
AGAGCATTTCTCAATTTGCATTAGGCACATTTGACTAGTGACTGCTGTATCGAACAACAGTTTTGGAGTCTAAAAGTGTAGACCAATGT

TACTCTTAGTGTATTTTCAGATTGGCCTCATCAGCAATCCTT|TGTGATGTGATAAAAATCCTTTTTATCCTGGGATATTCA|CTTTCC
 >chr1
TGGTAGACCTTCTACTTCTAACTTTCCTTTCTGCCTCCATCACTTAGTCTGGTTCTTTAGTTATGTTAATTGCAATAATAATTATTATG
0:32154649-32155098 - E=3e-243
CTAACAATAGAAATCACTATGCAAGATCATATGCTGACTTTAAACTTATGTTCTCATTTTAATTCATAAAGCAACAGCTTAGTATTTTC

CAGGGTTCTCATTTCATCTTGCACTTACTCTGTATGCTTTCATATGTTATTGACCCCACGTGTATATTAATGACTTCTATATTTTTAGC

CCTGATTTTTTTTTTTTTGAGACGGAGTCTTGCTCAGTCGCCCCGGCTGGAGTGCAGCGTCGCGGTCTTGGCTCACTGCAAGCTCTGCC

TCCTGGGTTCACGCCATTCTCCTGCCTCAGCCTCCGGAGTAGCTGGGACTACAGGCGCCCGCCACCACGCCCGGCTAATTTTTTT|GTT

TTTTTTGGAGAGGCGGGGTTTCACCGTGTTCGCCAGGGTGGTTTGTTATTGTTCACCGCGGTATGCGCCCCTCAGAGCGGGAGCGT
