# Supplementary material for: Chromothripsis during telomere crisis is independent of NHEJ, and consistent with a replicative origin
Source: Genome Res. 2019 May;29(5):737–49. doi: 10.1101/gr.240705.118 (PMC6499312; doi:10.1101/gr.240705.118)
Supplement: Supplemental Material [file supp_gr.240705.118_Supplemental_file_1.zip › contigs/annotated_contigs/DB107/contig.3.DB107_length_554_mean_cov_6.76173285199.docx]

**DB107_length_554_mean_cov_6.76173285199**

GCAAGGAATAAAAATACCAGTTATGTAATAGTCATTTGCCTTAGTCATGCAGCCCTGCCGGAGTGCTGTTCGAACAGAAAGATGATTAA
 >chr15:67334310-67334619 + E=2e-174
GTTAAACACTGCATATTTATTCCCCTGCGGTCACCCTAATTGGTTCTGCCGCTGTAATTGATTTGACAGGTTATTTTTACTCAGAATGA

GCTGGGTTCCTCTGCTGGCTTGCAGATTTGCTCTTGCTCTTCGAGTCCAGGCCGGCAATTGTCCAGATGGCCAGATGTGGCTGATTTCT

ACCTGAGGAGGGGGAGGGGGGTCCTCAACCTAGCCTGGGGAG|T|CATCTCTACTAAAAACACAAAAATTAGCTGGGCATGGTGGCAGG
 >chr1:44381618-44381747 + E=6e-60 p=1e-03
CACCTGTAATCCCAGCTACTCAGGAGGCTGAGGCAGGAGAATTGCTTGAACCCAAGAGATGGAGGTTGCAGTGAGCTGAGATCAT|TGC

ATCGCTTGAACCTGGGGACAGAGGTCGCAGTGGGCCGAGATGGCACCACTGTACTCCAGCGTGAATGACAGAGTGAGACTCTGTTTAAA
 >chr1:44383331-44383445 - E=6e-51
ATATATATATATAATATATATTA
