# Supplementary material for: Chromothripsis during telomere crisis is independent of NHEJ, and consistent with a replicative origin
Source: Genome Res. 2019 May;29(5):737–49. doi: 10.1101/gr.240705.118 (PMC6499312; doi:10.1101/gr.240705.118)
Supplement: Supplemental Material [file supp_gr.240705.118_Supplemental_file_1.zip › contigs/annotated_contigs/DB108/contig.2.DB108_length_389_mean_cov_12.9588688946.docx]

**DB108_length_389_mean_cov_12.9588688946**

TGCGTGGTGGGAAGTAGGGGGGGGAGGTGAGGGGTGGGGTCTTCCGGTCG|TGTGGAAGGTGAGTGTGAGAGA|TGGGTGTGGAAGGTG
 >chr16:81702774-81702829 - E=1e-18 p
TGAGGTGGGTGTGGAAGG|CCGTGAGTGTGAGGTGGGTGTGGAAGGTGTGAGGTGGGTACGGAAGGCAATGAGTGTGAGGTGGGTGTGG
=0e+00 >chr16:81702276-81702589 - E=8e-120
AAGGCAGTGTGAGGTGGGTGTGGAAGGCAGTGAGTGTGAGGTGGGTGTGGAAGGCAGTGAGTGTGAGGTGGGTGTGAATGGATGTGTGT

TTGTGACAGGCTGCTGTGGACAGAGGCATACATGTGCAATGAGGAGTTGGGCACCAGGCATTAGTGGCAGGGGAGGCACTGGCACGTGT

CACTATGATGAGGGGACAGGGACACGACCCTGG|GTG
