# Supplementary material for: Chromothripsis during telomere crisis is independent of NHEJ, and consistent with a replicative origin
Source: Genome Res. 2019 May;29(5):737–49. doi: 10.1101/gr.240705.118 (PMC6499312; doi:10.1101/gr.240705.118)
Supplement: Supplemental Material [file supp_gr.240705.118_Supplemental_file_1.zip › contigs/annotated_contigs/DB108/contig.2.DB108_length_405_mean_cov_5.99012345679.docx]

**DB108_length_405_mean_cov_5.99012345679**

CTATTGGGATGCTAAATTCACNAAAAATGTTAATAGGAGTTGGGTGGAGGTGGGGGACTGTGAGTCAGGTGCTAGAATCTTCGGGGAAT
 >chr9:73036805-73037062 - E=3e-140
GAGGGGGAGTGCAGGAGAGCCTGCAGATGACAGCCACAAAGCGGAGGTGGTATAGTCTGATGGCAGGTACTTCAAAGCAGCTGGGGTGG

AGGGGGAAGAAAGAAGAGTCTATCTTGGTGAATAATTCTCCTGCCAAGGGTTTGTTCTGAATTCCACTCTCTAGTATCT|ATTAGCCGG
 >chr9:7
GTGTGGTGGTGCACACCTGTAATCCTAGCTACTCAGGAGGCTGAGGCAGGAGGATCACTTAAACCCAGGAGGTGGAGGTTGTAGTGAGC
3047037-73047182 - E=9e-76
CGAGATTATGCTGCTGCACTCCAGCCTGGGCAACAGAGCGAGACTCT|ATC
