# Supplementary material for: Chromothripsis during telomere crisis is independent of NHEJ, and consistent with a replicative origin
Source: Genome Res. 2019 May;29(5):737–49. doi: 10.1101/gr.240705.118 (PMC6499312; doi:10.1101/gr.240705.118)
Supplement: Supplemental Material [file supp_gr.240705.118_Supplemental_file_1.zip › contigs/annotated_contigs/DB108/contig.2.DB108_length_427_mean_cov_8.38173302108.docx]

**DB108_length_427_mean_cov_8.38173302108**

CGCTAGGGAAAGCAGCGCCTGCACCAGGCCCCCGGAACTAAAGTGTCCACTGCGGGGCTCTGTCAAGGGGCCAGGTCAAGGACACAGGG
 >chr16:81702104-81702255 - E=2e-79
ACACTGCCTGTGCCGGCTGAGGGCCATATGTGGTGGCTGCCGTGCTCGGCCTCTTCCAGGTG|GAAGA|GGTCCTCTTGCTTTGTGCTG
 >chr16:81655531-816
TGGGGCCAAGTCTCAGAAGCTGAGCTGAGTCTCCACGTTGGGTTGCAAAGGGGAAGAGAACTAGTGTTTACTGAGCTTCTACTGTGTGC
55802 + E=1e-151
CAGGACTGGCGTGTTTTGAGCTCCTACTGTGTGCCAGGAGCTCTGCTAGTCTCATCATGTCCATTTCACCCTCACCACAACCGCATAAG

GTTGATGATTATCGTTGTTGCCATTTTCCATACGGGAAAGATGAGGCTCAGACTGGAGTCCAAGCTCCCATCG
