# Supplementary material for: Chromothripsis during telomere crisis is independent of NHEJ, and consistent with a replicative origin
Source: Genome Res. 2019 May;29(5):737–49. doi: 10.1101/gr.240705.118 (PMC6499312; doi:10.1101/gr.240705.118)
Supplement: Supplemental Material [file supp_gr.240705.118_Supplemental_file_1.zip › contigs/annotated_contigs/DB108/contig.2.DB108_length_581_mean_cov_8.51979345955.docx]

**DB108_length_581_mean_cov_8.51979345955**

ATGTACATGATGTTGATATTGTATTACTGGTCATTTTAACTTTGATCCTTTAGTTAAGGCGGCATCTTACAAGTTCCCCTTAAAAGTCC
 >chr7:40909525-40909840 + E=7e-178
TGGGTTTGCTTTTGTTTGTTTCTGTAATCAGTAAGTATATTTTGGGGAAATATTCTGAGACTGTACTAGTTCACTTCAGCAACTGTAAC

AAATTACCACACTCATGATCATGTAAAACAATAGAACTTAATTCTCTCAAAATTCTAGAGGCCAGAAGTCTTTGGATTTCATTCTGATC

AATGTCATTGAACTGAAAGAAAAGTGTTACATGGCTGTGCTCTCTGTG|TTATCTGTCATAGATAA|CATAGATAACACTGTAACAGCT
 >chr7:40800978-408012
TAATTATGGGAAATTAGAGAGGGGCCCATGATAGAGCACAGCATTTCCTAAATGTTCCTTGCCTAGAGGACCCTAGTTTTCTGAAATAG
27 + E=4e-138
TAATAGTTGTCCCACCACAAAATGACCCTTTTGTCAAACAACTTTGGGAAACCTGCATTAGGCAAAGGTCAGTGAATTTTCTTTACTTT

GACATTTCTCAGAAGCATTGATAGTCTGAAATGTGTTTGCCTAAGAGGG
