# Supplementary material for: Chromothripsis during telomere crisis is independent of NHEJ, and consistent with a replicative origin
Source: Genome Res. 2019 May;29(5):737–49. doi: 10.1101/gr.240705.118 (PMC6499312; doi:10.1101/gr.240705.118)
Supplement: Supplemental Material [file supp_gr.240705.118_Supplemental_file_1.zip › contigs/annotated_contigs/DB108/contig.2.DB108_length_607_mean_cov_8.07578253707.docx]

**DB108_length_607_mean_cov_8.07578253707**

GAACATAAACACAGAATCAGGGTCAAAGGAGATTAGAAAATGTTCCATTCCCATCCATTTTAATTAACACCTAAAGAATAATTTTATTA
 >chr11:128857527-128857847 + E=7e-181
TAATGCAAATTTCCTCTCTCATGGCTGTCATCGGTTCAAACTCAATTTGTTAAAATCTTCAAACACTATAAAGAGGCAAAATACAAGTA

CTGAAGAGATTAAGGTGCATGGTTCTCTGATACTACAGATGAAAAAAATGCCATTTAGAAAAGTATACAGAGACAATAACATGTATATT

ATTTCCTATTTTATCTAAATTATCTTTAATGAAAATAGGTTATCAAAGTAAG|T|ATAATTTTGTTTTTTTTTGAGACGGAGTCTCACA
 >chr4:34078768-34079056 + E=1e-155
CTCCCGCCCAGGCTGGAGTGCAGTGGCACAATCTCTGCTCGCTGCAAGCTCCGCCTCCTGGGTTCACGTCATTCTCCTGCCTCAGCCTC

CCGAGGAGCTGGGACTACAGGCACTGGCCACAATGCCTGGCTAATTTTTTGTATTTTTAGTAGAGACGGGGTTTCACCATGTTAGCCAG

GATGGTCTCGAACTCCTGACCTCGTGATCCGCCCACCTCAGCCTCCCAAAGTGCTGGGATTACAGGCGTGAGCCA
