# Supplementary material for: Chromothripsis during telomere crisis is independent of NHEJ, and consistent with a replicative origin
Source: Genome Res. 2019 May;29(5):737–49. doi: 10.1101/gr.240705.118 (PMC6499312; doi:10.1101/gr.240705.118)
Supplement: Supplemental Material [file supp_gr.240705.118_Supplemental_file_1.zip › contigs/annotated_contigs/DB108/contig.2.DB108_length_611_mean_cov_7.77086743044.docx]

**DB108_length_611_mean_cov_7.77086743044**

GGCCCATCCACCCCCCCTCTTCCAAAAGGAGACAGGCCCAGTGGGGGGCGAGATCATGCTGGAGTCCACAACTCTGTCCTCGGGGCTGT
 >chr2:242738401-242738702 - E=2e-139 p=4e-02
TAGGAGGTACCCGGGGCAGGACTTATGGTGGGGGCAGGACTTACGGTGTGTCCAGCTCTAAGTCCGAGTGCAGGAATCCGGCCAGCAGG

AGCAGAGTCAGGGCCAGGAGGAGGAGGAGGATGACCCGGAAGTATCTGTGGAAAGGGCAGGCCATGAGTGCCCAGTCCGGGTCGTGCCA

GCCCCACCTCCACCTCCCATCCTGCAGCCAAGT|C|TCCTGCTCTGCCATTGGGCCACTTCTGCGGACTTCCCAAGACCCTCCCCGGGA
 >chr2:242734801-242735112 - E=2e-175
GAGTGGCTGCACGAGGTGGGAAGGGCAGTTTTACGGCTCCGCACAGTTAGAGATTAACTCACTGATAAGGCCCACGCAGGACTTCACCA

GCAGGGTTTCTCAGCCACCACCCTTGGCCATCACTTCGTTCCGCCATAGTCAACTCCTTCCTCAAACACCCTGCACCTTTATGGCCCCT

CACTCTGCACTTTTATAGCCCCTCTGCACTTTTATGGGACGCACTGGCCATCTGTTTGAGATGCTGCTTTCACACTTAC
