# Supplementary material for: Chromothripsis during telomere crisis is independent of NHEJ, and consistent with a replicative origin
Source: Genome Res. 2019 May;29(5):737–49. doi: 10.1101/gr.240705.118 (PMC6499312; doi:10.1101/gr.240705.118)
Supplement: Supplemental Material [file supp_gr.240705.118_Supplemental_file_1.zip › contigs/annotated_contigs/DB108/contig.2.DB108_length_619_mean_cov_11.5605815832.docx]

**DB108_length_619_mean_cov_11.5605815832**

GAAGGACAGGCTCTGAAAAATCTTTCTTTCAGCAAAACGTCTACTAATCTCAGAAAGTGAGCTTTGTCACCATTGATACTCTGTAAGGT
 >chr7:130598896-130599164 - E=1e-149 p=4e-02
GGGATGAAAGCAAGTTTTCTTACTCATTTTGCCCAGTTGTCAAGAAATGCTGCCCTTCTCTCTAACATAAATTAACAGGTTTGGCTACA

GCCTAGAGAAATACAAAATGTCACATTTTAGCCTTAGTGGCCGGTTTTCAAAGGGTGCCTGAGTGTGAACACCATGTCGGCTGCTCTGT

T|CCTTGTT|CCGTCCACATAGGAACAAGGAACCTGAGGTGAAGTGAGCCGAACTGGCTTACCCAAGCTCACACAGCGCAGGGCCGTCA
 >chr7:130611245-130611534 + E=2e-151
GCATTGGGAGGCAGCCCTGGGTCCTATAGGCCTCATGCACGGCCTGCCTTGCTGTCGAGAAAGTCCCAGCCACCGACACAGGCTGCAGG

AAACGGCACCAAAGTCACTTGCAGCAGCGCAGAGAAAGTGTGAACAAAGAACCTTTTTTTTTTTTGGGAGATGGAGTCTCACTCTGTTT

CCCAGGCTGGAGTACAGTGGGGCACG|CTCCCAGCGCCCGTCCACCTCCATCTCCCTGGTATATGTGCCTCTCCTGCCTCCTCCCCCC
