# Supplementary material for: Chromothripsis during telomere crisis is independent of NHEJ, and consistent with a replicative origin
Source: Genome Res. 2019 May;29(5):737–49. doi: 10.1101/gr.240705.118 (PMC6499312; doi:10.1101/gr.240705.118)
Supplement: Supplemental Material [file supp_gr.240705.118_Supplemental_file_1.zip › contigs/annotated_contigs/DB108/contig.2.DB108_length_631_mean_cov_8.77020602219.docx]

**DB108_length_631_mean_cov_8.77020602219**

GAGGCTGAGACAGGAGAATTGCTTGAACCTGGGAGGCAGACTTGCAAGAGCCGAGATTATGCCATTGCACTCCAGCCTGGGCAACAGAG
 >chr17:57586346-57586716 + E=4e-200 p=0e+00
TGAGATTTTGTCTCAAAAAAAAAAAAAAATCAGGTGTCCTCAACCTCTAGGCCATGGACCAGTCCACAGCCTGTTAGGAACAGGGCTGC

ACAGCAGGAGGTGAGTGGCAAGCAAACAGGAGCGAGCATTGCCACCTGAGCTCCGCCTCCTGTTAGATCAGCAGCGGCATTAGATTCTC

ACTGGAGCACAAACCCTATTGTGAACTGAGTATGCAAAAGATCTAAGTTGCAGGCTCCTGATGAGACTCTAACTAATGCTTGATGTACT

GTACTGCC|GGCAGGTTGCAGTGAGCCCAGATTGCATCACCACTGCACTCCAGCCTGGGCAACAGAGGGAGACTCTATCTCAAAAAAAA
 >chr17:57762095-57762365 + E=2e-140
AAAAAATCTTGCCCTCCAAATTAGAAATATTGTCCATTTCATAGCCATATGTTAGTCCTTGATAGTGAAAATCTTGGAGAAAATGGTAT

CTTTAGGTAAAATGAAAAATCATACCTTTTGATGCTTGAATCTAGGTGTCATATTACATGTTACCATCTAAGGGGCTCATTGATTGAGA

ACGGAATAT
