# Supplementary material for: Chromothripsis during telomere crisis is independent of NHEJ, and consistent with a replicative origin
Source: Genome Res. 2019 May;29(5):737–49. doi: 10.1101/gr.240705.118 (PMC6499312; doi:10.1101/gr.240705.118)
Supplement: Supplemental Material [file supp_gr.240705.118_Supplemental_file_1.zip › contigs/annotated_contigs/DB108/contig.2.DB108_length_647_mean_cov_9.43894899536.docx]

**DB108_length_647_mean_cov_9.43894899536**

GAGTGCAATGGCGTGATCTCAGCTCACCGCAACCTCCACCTCCCGGCTTCAAGCAATTCTCCTCCCTCAGCCTCCCAAGTAACTAGGAT
 >chr16:81857787-81858222 + E=5e-235
TATAGGCATGCGTCACCACACCCGGCTAATTTGTGTATTTTTAGTAGAGACAGGGTTTCACCACGTTGGCCAGGCTGGTCTTGAACTCC

TGATCTTAAGTAATCCACCCACCTCAACCTCCCAAAGTGCTGGGATTACAAGCGTGAGCCACTGCACATGGCCCCAGCCACCTCTTTTC

TTGTGATTTGTCAGCAAACAGCCTCTGGGGTTGTGACTGCTGGAAAGCACCTCCAGCTTTTGTGGAGCCAGGTGTGGCTGGGACTTTTC

GTTGGGATCATGTTTGGATCTCCCTCAGGCCTTCTCACCATGTTCCTCGTGTGCCACACTGGTGAGATAACGGGGTGTG|AAATTATAC
 >chr16:
GGGCTCTGCCATCTCCTGCCTTCAACTCCCTAGGCAGAGACGCAGGAAGGAGGAGGCGATTATGATACATTTGCTACAAACCTCAAAGA
81691517-81691729 - E=8e-116
CAGACAATTACACTCCCGCTCCTATCAACTGGGGCTCTGAAATATGCAAAGGCAGAGGGAGGTGCGTCCGGCTTGCTAGAAAAGACAAA

ACGGAGCAAGGCCAGATGCTGGTGG
