# Supplementary material for: Chromothripsis during telomere crisis is independent of NHEJ, and consistent with a replicative origin
Source: Genome Res. 2019 May;29(5):737–49. doi: 10.1101/gr.240705.118 (PMC6499312; doi:10.1101/gr.240705.118)
Supplement: Supplemental Material [file supp_gr.240705.118_Supplemental_file_1.zip › contigs/annotated_contigs/DB108/contig.2.DB108_length_663_mean_cov_9.39366515837.docx]

**DB108_length_663_mean_cov_9.39366515837**

GGAGTGGTGGCTTCTGGCACTTTGGAAGACTGAGGTGGGAGGATGCCTTGAGCCCAGGAGTTCAAGGCTGCAGTGAGCTGTGATCATGC
 >chr17:16264058-16264355 - E=1e-159 p=0e+00
CACTGCACTCTAGCCTGGGTGACAGAGTTAGACCCTGTTTCAGAAAAAAAAAAATAGATGTTATGATTTAGCAAGTCTTATCAAAACCT

ACCCAAACTCAATGTATGTTACTAAGAGGGTATAATACAGATGATGAAAATATTTTCCAAAGGACCCAAGAAAGCATATAAGATCCTGT

GATAAGTGTTTTGAGTTTATTCTGTTT|ATGTTGTGTT|GCCTGGGCAACACAGCGAGACTCTGTCCCCCACCCCCACCACCCACCACC
 >chr17:16307412-16307772 - E=5e-197
GCAAACACACACACAAAAACTGAAATAAAAACTGAAATTGAAAATTGGAAAACACTGCATTAAACAATGTTAAACCAGTTCCTATAAGC

TAGAACTCCTTGTTAGCTAGGCATGGGAGTGCATGCCTGTAATCTCAGCTACTCAGGAGGCTGAGGCAGGAGAATCACTTGAGTTTGGG

AGTTGAGGCTGTAGTAAACTATGATTGTTCCACTGCACTCCAGCCTGGGTAACAAGGTGAGACAGTCTCTGAAAAAAACAAACAAGCAA

ACAAAACAACAACAACAACAAAACACACAAAGAAAATAGGAT
