# Supplementary material for: Chromothripsis during telomere crisis is independent of NHEJ, and consistent with a replicative origin
Source: Genome Res. 2019 May;29(5):737–49. doi: 10.1101/gr.240705.118 (PMC6499312; doi:10.1101/gr.240705.118)
Supplement: Supplemental Material [file supp_gr.240705.118_Supplemental_file_1.zip › contigs/annotated_contigs/DB108/contig.2.DB108_length_671_mean_cov_8.49478390462.docx]

**DB108_length_671_mean_cov_8.49478390462**

AGCAATGTTTTCACAGACCATTACTATTCAGCTACTCACTGCCTGTAATAGTAGCTAAATCGTATCTGAGATTTATTCCAGCTCTCAAA
 >chr2:35537923-35538231 + E=1e-173
ATATAAAAAGTCTAAGATAATTGCAATATAGTTTTAAAAAATCTTTAAAACAAATAGAACAAGGAAGAACAATTAATGTTTTAGTCAGA

GTCTTGGATCATTAATACTCTTCATGAGTAAATTCATAATTTGTTCTCCACCATGAGAATTATTCAGAGAGAAATTTCTTAAATCTGCC

TGTGGCACACACTGGCTGAAAGGGATAATAAATTCA|TGAAA|AATAATGATAGCATGAAGATACACATTTAACTAATTTATAATACAG
 >chr2:35540283-35540651 + E=1e-209
ATTTAGTAGAGTCTTAAAATCTATGGTAGTCAAAATATTGCTTCCATTTCCTTATGTCTGTTTCTAATCTGTAGGAACACATCAAAAAG

ATGTCAAAAAGAAAGCAACTCATGAAAGTGGCACTGGGAGCCACCCTTTGCACAATTCCAACAGGTTCTTGGAGAAGACTTTATTAGGA

TGAAGTGCCAGGGTTTAGATTTGTATTTATGTATTGACTCAATAATTCACTCAATACATGCTTTGACATGTGTAATAGTTCCTCTTCCT

TAACAATGTACTGCTCCAAAATTAGTTTATGAGGACTGAAGTCAACATGT
