# Supplementary material for: Chromothripsis during telomere crisis is independent of NHEJ, and consistent with a replicative origin
Source: Genome Res. 2019 May;29(5):737–49. doi: 10.1101/gr.240705.118 (PMC6499312; doi:10.1101/gr.240705.118)
Supplement: Supplemental Material [file supp_gr.240705.118_Supplemental_file_1.zip › contigs/annotated_contigs/DB108/contig.2.DB108_length_717_mean_cov_6.83821478382.docx]

**DB108_length_717_mean_cov_6.83821478382**

CCCCGCAACCCACCCTGGAAAC|CAGAGCAAGCCACCAAGAAAATCAAAATAAAAATAAAAATAAATAAATAAATAAATTTCTCCTTCT
 >chr16:81751730-81752021 - E=3e-148
CAAATGGGTATCAGTCATATTAAACTTGTGCTCACCCTAATGATCTCACTTTAATTTGATCACCTGTGTAAAAACCCTATTCCCAAATA

AAGTCGCATTCTGAGGAACTGCAGTTTAGGACTTCAATGTCTCTTTTCTGGGGGATACAATTCAACCCTGACAACCGTACACCCCCAAG

CCCATCACACTTCCCACTGGCCGTTCCCAGCCTATATCCGGCTCTGG|AAGACCGGATATA|GGCACAAAGAAAGTTATCATGGATGCA
 >chr16:81730437-81730795 -
GCTATTGGTTTGGTGACCTCAGACCCCTCATGATAGTGGGAACAATCCCCCTGACCGCGACACCTCCTTGTAAGGATTAAACAAGCTGG
 E=1e-200
TGGACCTGCAGTGCCTTCCAGAATCCCTGGTCCCGTTGGGCACTCAATTTCTTGTAGGAACATAACCATGGCAATGAACTGTGTGACCT

TAGGCAGTGACGGCCCAGGCTTTAGCTGCCTTGGGTATAAAATGGGGCAATGACATGTGTCGTCTTCTCACTGTGCTGTGAGAGCTGAG

TGCAACTGGGAAAGCCAAGTGCTTGGCTTGGAGCCTCAGATAAGAAATGAGTAAGTGACAGTCA|AGATCGGAAGAGCGTCGTGTAGGG

AAAGAGTGT
