# Supplementary material for: Chromothripsis during telomere crisis is independent of NHEJ, and consistent with a replicative origin
Source: Genome Res. 2019 May;29(5):737–49. doi: 10.1101/gr.240705.118 (PMC6499312; doi:10.1101/gr.240705.118)
Supplement: Supplemental Material [file supp_gr.240705.118_Supplemental_file_1.zip › contigs/annotated_contigs/DB108/contig.3.DB108_length_583_mean_cov_7.46140651801.docx]

**DB108_length_583_mean_cov_7.46140651801**

CTGTACCAATCAACAGAACCCCATTAACTGTCTAAGTGAGCATACACGTGACTTCACTGTTTTCAAGGTTACCCTTCATCTCAGCTCAC
 >chr5:166956428-166956680 - E=6e-140 p=8e-03
TCTGTATTTACTCATGTGTTCAGACATTCTGGGCTTAAGATATATGCTGGTTACTCTACAAAAGACAGGAAAGTTCTCCTAAGTGCAGA

AAGTGAACACATTAAGTTGCAATACAGGAAAGACTAAACCAAGAAGCATGGCAATGGATTCTAGTTATAGCAAC|TATAAAAGTTATAA

AAAGT|TAAAAAACTTTTTATAAAAAACTTTTTATAAAAG|TTAT|AAAAAGTTAAAAAATTTTCTCCATTTATATAGCTAGACCGACA
 >chr3:161596627-161596661 + E=9e-03 p=1e-02 >chr5:166955374-166955648 - E=3e-15
AAAAGGGACTTGAATCCCAGCTCACTTAGTATGTGGCAGATACTATGGTAAGAATTTTATATACATGACTTTATTTAAGCCTCCCAACA
3
AACTTCTAAAGTAGGTGCTATTATATGTCAAGTTTAAAGATGAAATTACCCAGACGCAGATTGAGACATTAAGCTACACCTCTTAGTAA

GGCAAAGAGCTGGGATTCTGAATGCAGTACCCATGCTTTGTTCACATTACCAC
