# Supplementary material for: Chromothripsis during telomere crisis is independent of NHEJ, and consistent with a replicative origin
Source: Genome Res. 2019 May;29(5):737–49. doi: 10.1101/gr.240705.118 (PMC6499312; doi:10.1101/gr.240705.118)
Supplement: Supplemental Material [file supp_gr.240705.118_Supplemental_file_1.zip › contigs/annotated_contigs/DB108/contig.3.DB108_length_634_mean_cov_5.91482649842.docx]

**DB108_length_634_mean_cov_5.91482649842**

AGAAAAAAAACACATGTATAAATTTAAAGTTTGTTTTGAAAAATTACTTCATCCATGTACTGTCCAGCTCATAAAAACAAAACTTCTTC
 >chr7:130626883-130627198 + E=8e-178 p=0e+00
CAGCGAAACGGGACACTTTGACCTTGTTTACTGCAGTGTTTGGAAACCGCTGTCTCGTAACTCCTGACATCACCGGTGGTGCATTAGCA

CCTGGACTGCTGTTGGCTGCACATGCTGATACCCCAACTCTCTTGAATCACCCGTGGCTGAACAAATACTCACAGAACAGTAAGGATAG

TGGCATTAGCATTAAACCCACAAAACTCACAAGACCTTTTAGAAGTGT|ATATATATATATACTTTA|AGTATATATAGAAGTGTATAT
 >chr15:83898785-8389
ATATAC|ATGCTAGAATCGC|TGTCTCCTAACCCTCCCCTTGCTGTTGTTCTTGGAATGGGTACCTTATTCATGGCCTCCTTACATAGA
8812 - E=2e-01 p=1e-02 >chr7:130573426-130573687 - E=3e-142
GGGAGCAGATTCAGGACAGAGGCTCTGCACGCTCCTGGGTCCCTCCTAAGCGAGCACTCCCCTCCACTGCTCCTCCTGTTCGGCATGCT

AGATTTTCTGTGCACTTGCAGTCATACGGAAACTGGCTTCCTTCTTAGCTGTGCGTGTGAAAGTCACCCCAGTGTTTCACCTGCAGTGT

GAGTGTAATTTGGGG
