# Supplementary material for: Chromothripsis during telomere crisis is independent of NHEJ, and consistent with a replicative origin
Source: Genome Res. 2019 May;29(5):737–49. doi: 10.1101/gr.240705.118 (PMC6499312; doi:10.1101/gr.240705.118)
Supplement: Supplemental Material [file supp_gr.240705.118_Supplemental_file_1.zip › contigs/annotated_contigs/DB109/contig.2.DB109_length_309_mean_cov_2.90938511327.docx]

**DB109_length_309_mean_cov_2.90938511327**

AAGGAGAAAAATGATAACCACATGCATTTCATGATCCATAGCAATTCTGAAACAAAATTGGGCAAATAGGTCATATTTTCCAGTTTCAG
 >chr21:23625931-23626205 + E=3e-154 p=1e-03
GGGAAAGCATGCTCCCTGACGAAGCCATATTCCTTTTCCAGTGAGCGACTTCCCATATTTCCTTCATGTTCCCTGGCTCTACCCTCTGA

GATATCCTTTATTTTTTTTCATCCTCCTTAGATATTTCTGGAGAGTTCAAAGGATAATATTTTCTTACAGAAATTTTCTTAACCTAT|T

TTCTTTTC|TTTTTTTTTTTTTTTTTATAAAGGA|AAGATGTTTA>chr7:112193172-112193206 - E=5e-03
