# Supplementary material for: Chromothripsis during telomere crisis is independent of NHEJ, and consistent with a replicative origin
Source: Genome Res. 2019 May;29(5):737–49. doi: 10.1101/gr.240705.118 (PMC6499312; doi:10.1101/gr.240705.118)
Supplement: Supplemental Material [file supp_gr.240705.118_Supplemental_file_1.zip › contigs/annotated_contigs/DB109/contig.2.DB109_length_370_mean_cov_7.02702702703.docx]

**DB109_length_370_mean_cov_7.02702702703**

AATAGACCAATCTATAGAGATGGAAAGTTATTTAGTGATTGCCTAAAGGTGGGGTTCAGAGTGGAGAATGGAGAGTAACTGCAAATGGG
 >chr2:124502015-124502310 - E=2e-166 p=0e+00
TGTGATACTACTTTTGTAGTGATGAAAATGTTCTAAAATTAGATTGTAGTGATGGTGGCATAAATCTATAAATATGCTATAATCCATTG

AATTTTATACTTAAAATTTTAATAGGAAAATTATATATATACATATATACACACACACATATACATATACGTATATGTGTATATATATG

TATATGTG|TATATATATGTATATGTATA|TATATGTATATGTATATGTATATGTATATGTATATATATATATGTATATGTATATGTAT
 >chr8:106342777-106342870 - E=1e-24
ATATATATATATGTAT
