# Supplementary material for: Chromothripsis during telomere crisis is independent of NHEJ, and consistent with a replicative origin
Source: Genome Res. 2019 May;29(5):737–49. doi: 10.1101/gr.240705.118 (PMC6499312; doi:10.1101/gr.240705.118)
Supplement: Supplemental Material [file supp_gr.240705.118_Supplemental_file_1.zip › contigs/annotated_contigs/DB109/contig.2.DB109_length_440_mean_cov_5.44545454545.docx]

**DB109_length_440_mean_cov_5.44545454545**

TGGAATATATTAGATCAATGGCTTAAATGAGAGATCCTTTGTTAAGACTTCTGAAAGAATAAAATCAGTACTTCTTAGTAACTCTCAAT
 >chr21:23626190-23626547 - E=1e-194
TAGACAAAATTCACCATTTTGAAGATTTGCCCCTCTGAAAACTAAATGCCCAATGTATCAGTTAATACATTGAGAGACAATTACTCAAA

AAGAATTTCTGATGTGTCTTTCGAGTTACAACTCTTAGAAATCAGACTCATAGATAATTTGCCAAGTGTTTAATGGAGTTATGCCCAGA

AACAAGTTGGCAGTCTTTATCGAAAGGCACTGAGACTGTTCAAACTCCAAAAATTCTCTGATTCCACATCTTTATGAAAAGAAAATAGG

T|GGGAGGAGCCAAGATGGCCGAATAGGAACAGCTCCGGTCTACAGCTCCCAGCGTGAGCGACGCAGAAGACGGTTATTTC|CGCA >chrX:118575377-118575456 - E=5e-33
