# Supplementary material for: Chromothripsis during telomere crisis is independent of NHEJ, and consistent with a replicative origin
Source: Genome Res. 2019 May;29(5):737–49. doi: 10.1101/gr.240705.118 (PMC6499312; doi:10.1101/gr.240705.118)
Supplement: Supplemental Material [file supp_gr.240705.118_Supplemental_file_1.zip › contigs/annotated_contigs/DB109/contig.2.DB109_length_487_mean_cov_7.01026694045.docx]

**DB109_length_487_mean_cov_7.01026694045**

CTTCCATCACCGTTACCAACTGACTTTTGATCATTGCCCGAAGTGTCAGTCTGAACGCCTTTCACTCAATTTTTCATTCTTCCTCCCTT
 >chr11:129607614-129607838 - E=3e-120 p=1e-02
TCCACAGTCTCCGCTAGCACAGGAAGGAGTTATTCAAAAGTATTTACTGAGATCCTATTACTTGCCTTCAGTCATTCTGCACACTGGGA

ATTGCAGGCAATGCAAAGGCCGGAAATAAGGCAGGCTGGGTGGG|TG|GAAAGAAGAAAGTGGTCATGGATCTCCTGTCCTGGAACCTG
 >chr11:129705886-129706094 - E=1e-98
GTAGGGCAAATTCCGTTCAATTTTTAATTGAATTTTAATTTTTTTTAAATTTGAAATGGAGTTTCACCCTTGTCGCCCAGGCTGGAGTG

CAATGGCGCGGTTTTGGCTCACTCCACCCTCTGCCTCCTGGCTTCAAGTGATTCTCCTGCCTCAGTCTCCCAAGTAGCTAGGATTACAG

G|TGCCTGCAACCAAACCTGGCTAATTTTTTTTGTATTTTTAGTA
