# Supplementary material for: Chromothripsis during telomere crisis is independent of NHEJ, and consistent with a replicative origin
Source: Genome Res. 2019 May;29(5):737–49. doi: 10.1101/gr.240705.118 (PMC6499312; doi:10.1101/gr.240705.118)
Supplement: Supplemental Material [file supp_gr.240705.118_Supplemental_file_1.zip › contigs/annotated_contigs/DB109/contig.2.DB109_length_495_mean_cov_12.1696969697.docx]

**DB109_length_495_mean_cov_12.1696969697**

CATATTCAAGTCTTAATGTCTAAAGAGCACTCATTGCAATACACAGACCTCCAGTTTTAGGTTTCTTTTTTCAGAGACAGGGTTTCACT
 >chr6:5221010-5221273 + E=1e-146
GTCACCCAGGCTGAAGTGCAATGGCCCTATTATAGCTCTCTGTAGCCTCAAACTCCTGGGCTCAAACGATCCTTCCGCCTCACCCTCCC

AAGTAGCTGGGAATACAGCAGCATGCCACCATGACCTGCTAATTTTCATTTTGTAGAGAGAGGGTGTCACTATGCTGCCCAGG|CT|AT
 >ch
ACCTTTATAAATCAACTACGATTAAATCAAAGGATCAATCTACATATTTAAAAAGTAATAATAGGCTGGGTGCAATAAAAATGTAATAA
r6:5209505-5209739 - E=3e-129
TAGGCTATTGTAATAAAAAAGTAATAATAGATTCACACCTGTAATCTCAGCACTTTGGGGGGCCAAGGTGGGTGGATCACTTGAGGTCA

GGAGTTCCAGATCAGCCTGGCGAACATGGTGAAACCCCATCTCTACTAAAAA
