# Supplementary material for: Chromothripsis during telomere crisis is independent of NHEJ, and consistent with a replicative origin
Source: Genome Res. 2019 May;29(5):737–49. doi: 10.1101/gr.240705.118 (PMC6499312; doi:10.1101/gr.240705.118)
Supplement: Supplemental Material [file supp_gr.240705.118_Supplemental_file_1.zip › contigs/annotated_contigs/DB109/contig.2.DB109_length_519_mean_cov_9.77263969171.docx]

**DB109_length_519_mean_cov_9.77263969171**

AAGTATTTCCTTCATTTAGCAACAGGTATAGCATGCAAATGTAGTACAAAATAAAATATTCAACCTTAAAAAGGATTTCCTTCCTTTTT
 >chr9:21882045-21882319 + E=3e-153
ACTGCAAATACAAATGCAAAAGAATGAAGTTGGACTCATCACCATATTAAAAAAATTAAAGTGGATTGAAAACTTAAACACAAGACGAA

CAACTGTAAAGGAAGGAAAACCTTTTTAAGGTTCGATAATATTTTATTTTATACTACGTTTGCATGCTACCCCTGTTGCTAAACTGTGT

ACTT|AAA|TATGTTGTTTTTTAAATCTAATAAACAAGCTATTTGACAATTCAGGAACATGAAGTTGACCATTATCAATGTAATTACAT
 >chr9:21531439-21531687 + E=1e-137
TAACCTTGTGCCAACTGAGTCTCCTCCTCCTTCCCTAATTTCTCTCTTCATAGCAATCAGAGCTTTCCCTAGTATTTTTTTAGTGTGTG

ACCTACATCTGAGTTTCCCAATTTGCTTTGGTGGAGGGAATCAAAAAGTCAGTTTCTACATCCAATAAAATCTACC
