# Supplementary material for: Chromothripsis during telomere crisis is independent of NHEJ, and consistent with a replicative origin
Source: Genome Res. 2019 May;29(5):737–49. doi: 10.1101/gr.240705.118 (PMC6499312; doi:10.1101/gr.240705.118)
Supplement: Supplemental Material [file supp_gr.240705.118_Supplemental_file_1.zip › contigs/annotated_contigs/DB109/contig.2.DB109_length_537_mean_cov_5.74301675978.docx]

**DB109_length_537_mean_cov_5.74301675978**

TCTGATCTTGTGGCCCCTATCCAGGAACTGACTAAGCACAAGAAGACAGCTTCAACTCCCTATGATTTAATCTCTGACCAGTCAGCACT
 >chr7:129864966-129865225 + E=3e-141
CCCGGCTCACTGGCTTCCCTGCACCCACTAAGTTGTCCTTAAAAACTCTGATCCCCTGGCCAGGTGCAGTGGCTCACGCTTGTAATCCC

AGCACTTTGGGAGGCCAAGGCGGGCAGATCATCTGAGGTCAGGAGTTTGAAACCAGCCTGACTAACATGGTGAAATCCTGT|TTC|AAC
 >c
AAATTGAAGGATGGTAAATGTGGGGAATTTTACTGCTGATGAAAGTGACTCTCAGTGAGAAGGGGAGCTGAATAAGGGATGGGGTGGGC
hr7:129871894-129872169 + E=7e-151
AGGTAATTTTCCCCTGGAGTCTGGCCATCTCTGGCTGGATTCTTCTCTGAAATTACACTGTCAGACTGTCCCTCTGAAGTCAAGCTGCT

TCTCTCCAACATCCAACCATAGTCCCTGAGGTACAGCTGCTTCTCAGGGATTTTTATGGGCACAGGATTTGGGGTAGGGCAGGCCATGG

GTAGT
