# Supplementary material for: Chromothripsis during telomere crisis is independent of NHEJ, and consistent with a replicative origin
Source: Genome Res. 2019 May;29(5):737–49. doi: 10.1101/gr.240705.118 (PMC6499312; doi:10.1101/gr.240705.118)
Supplement: Supplemental Material [file supp_gr.240705.118_Supplemental_file_1.zip › contigs/annotated_contigs/DB109/contig.2.DB109_length_587_mean_cov_9.33049403748.docx]

**DB109_length_587_mean_cov_9.33049403748**

CAGCTCAGGGATCACCTTAAAAGGACAGCCTTCCTGTCTTCCAGGGCCAAATCGGTAACTCCCTCTTCAAGCGCTGCCTAAGCACTCAC
 >chr6:4774840-4775116 - E=2e-148
GCGTGCTCACTGCAGCACTCGTCTCTGTATTTTAAGGTTTGTCTACTCTTCACCCCACCCCAACTCCAAACGATGACCTTCAGTCAGAC

TTCATCTTCAAATTTGACGCTGGCCCCTAGCACCATTCTCTATACAGGAAATAAGGTGTTGTTCAATGAATGGAATTTCAGTGTCCACG

AGAAA|GCCC|TGGGGAGGGCGCCCTTGCCCTGCACATGCCACTGCTCCAGTGCGTCTGTCCTGGGAGGGGAACTCACTGGGATGTCAG
 >chr6:5215172-5215488 + E=1e-173
ATGAGGGCCAGTTTAGTATTTTTTTTTTTTGCAAGGGTAGTTTGAGCATTTCAAACCAAGAAGTGACACAGACAATTAGATATAGCGGG

AGAAATGATGGCACAGATTATTTACAATCCCCCTTTTCTAGGCAGTTTATACTCTGGATAATTTTACGCTAAAGATAAAACCTGCCTGT

TTTGCCAACCTCTACACAGAGTAAGTGCCAAAGAACAAAATCTTTCTGAGTTTAT
