# Supplementary material for: Chromothripsis during telomere crisis is independent of NHEJ, and consistent with a replicative origin
Source: Genome Res. 2019 May;29(5):737–49. doi: 10.1101/gr.240705.118 (PMC6499312; doi:10.1101/gr.240705.118)
Supplement: Supplemental Material [file supp_gr.240705.118_Supplemental_file_1.zip › contigs/annotated_contigs/DB109/contig.2.DB109_length_634_mean_cov_8.62460567823.docx]

**DB109_length_634_mean_cov_8.62460567823**

GATTTTCCTATTGGCATTTCAAATATATGATATTTAAGTTCAAATCAAATACATGTGTTCCCCTCAAATCTACATCTAGTGCTAATACT
 >chr12:79359220-79359481 + E=3e-145
TACAATCTTTACAATTATAACCTAGTAAATCAAATATCACTTTAACTAGTCTTTGAGGGAAACCACTTTTTGAGAGTTTTCACAGGTGA

GAAACAGGAGGCTTAATGGGGCTCAGCCACTTGCCAACAATCACATAGTTAATAAGTCCCAGAGCTGACACTTGAGCCAAG|AT|TAGA
 >chr1
ATTATTTTTGAGGACTGGAAGAATCTTTCCTCACTAACTAGCATATCCCAAATTTACACCATGACACTTACCAATTTATAAAAATATTT
2:79369834-79370209 + E=6e-214
TATTTTATTTCCATATATGTCGCCCTAACTTCTTTAGAGGCATGAACCTATCTTTTCTCTTTTTTATCCCCAAGGACTGGCCAAAAAGA

AGAAAAAAAAGTTTAAAATAGGCAATTAATATTCATTAAATAAATCACAGATAGTAAAGAAGTATTAAAAGCAGCCTGAGCTGGAGTCA

ACTATTTCTTGTCTGTATCAGCCAGGCAAAATGTTGCTCAGAAGCCATTCAGGGAATAAAACTCTCTTGTGAAGAATCTGTTCCTTTAA

ACTAAGGACTTAC
