# Supplementary material for: Chromothripsis during telomere crisis is independent of NHEJ, and consistent with a replicative origin
Source: Genome Res. 2019 May;29(5):737–49. doi: 10.1101/gr.240705.118 (PMC6499312; doi:10.1101/gr.240705.118)
Supplement: Supplemental Material [file supp_gr.240705.118_Supplemental_file_1.zip › contigs/annotated_contigs/DB109/contig.2.DB109_length_767_mean_cov_7.43155149935.docx]

**DB109_length_767_mean_cov_7.43155149935**

CATCTATACCCCTTCAAACCCTGTGAGGTAGGGAGGGGAGGTATGATTAACCCCATTTGATGGATGAGTAAACTGAGATTTAGAGATTA
 >chr7:129985709-129986063 + E=4e-201
GGGCAGCTGCCCCAGATATTTTAGACGGGAAGTGAGCACCCAGTCTCTGTCTCTAAGTCCAGCGGTGTTTCAATGGCATCCACACCTTA

TGTGTAAGGAGGATATGGCCCAGGGAAAGCATGAGTGGGCTAGGCGGAGAAGAAGAGCGAAAGAGAAGATGCTGTAGAGGAGGGAGGAA

AGGAGGGGTATCCCAGGGCTGCTGGCTACTTTGGTCTTTCTCCAGAGATGGAAGCTTTGATGCTCACAAGGGGCTGCCCTGGCAA|AC|
 >c
ATCATTTCACGCCTACTGCAGGATGACTATAAAACATATGACAAGTGTTGCAAGGATGTGGAGAAAGCAGAACCCCATACACGCTGGTG
hr7:129820608-129821023 + E=8e-235
GGAATGTAAAATGGTGCAGCTAGGTGCTTTAGAAGAGTCTGGCAGTGCCTCAAAAGGTTAGTCAGAGTTACCATACAGCTCAACAATTC

CACCCCTAGGTATATACCCAGGAGAAATGAAAACATAGGTCCAATATAAACACTTGTACGTGACTGTTCACAGCAGCATTATTCATAAG

AGCCAAAAAGTGAAAACCACCTAAATGTCAATCAACTGCTGAATGAATAAGCAAAGTGTAGTAGATCTATTTTGAATATTATGCAGCAA

TAAAGAAAATGATGTAATGATAAATTCTACAACATGGACAAACTTTGAAAGCATTAT
