# Supplementary material for: Chromothripsis during telomere crisis is independent of NHEJ, and consistent with a replicative origin
Source: Genome Res. 2019 May;29(5):737–49. doi: 10.1101/gr.240705.118 (PMC6499312; doi:10.1101/gr.240705.118)
Supplement: Supplemental Material [file supp_gr.240705.118_Supplemental_file_1.zip › contigs/annotated_contigs/DB109/contig.4.DB109_length_584_mean_cov_12.4606164384.docx]

**DB109_length_584_mean_cov_12.4606164384**

GCAGCTGGGACTACAGGTGTGCACCACCACATCCAGCTGATTAT|AT|TGTGGTTGGGTGTGGTGGCTCACACCTGGGCGGGCAGATTG
 >chr2:124501712-124501756 - E=9e-15 >chr2:124501491-124501681 + E=1e-96 p=0e
CTTGAGCCCAGGAGTTCAAGACTAGCCTGGGCAACATAGCAAGGCCCCGTTTCTACAAAAACAAACAAACAACAACAACAACAACAAAA
+00
ATATATATATATCATTTATATATATATATTTATATCATTATATATATATCATTTATATATATAT|TTTATATATA|TGTATATGTATAT
 >chr2:124501
ATATATATATATAATATCTCAATAAAGCTGTTAAATCCAAAGAGATTTGAGGGGGGATGTTGAGGGAGAATTTTTCTTCATTTGTTGTT
712-124501979 - E=6e-146
GCTGTTGTTGTTGAGACAGGGTCTCACTCTGTCACCCAGGCTACAGTGCAGTGTCACAATCATGGCTCACTGCAAACTCTGCCTCCCGG

GCTCAAGTGATTGTTTCACCTCACCCTCCTGAGCAGCTGGGACTACAGGTGTGCACCACCACATCCAGCTGATTAT|AT|TGTGGTTGG
 >chr2:12
GTGTGGTGGCTCACACCTGGGCGGGCAGATTGCTTGAGCCCAGGAGTTCAAGACTA4501491-124501556 + E=2e-27
