# Supplementary material for: Chromothripsis during telomere crisis is independent of NHEJ, and consistent with a replicative origin
Source: Genome Res. 2019 May;29(5):737–49. doi: 10.1101/gr.240705.118 (PMC6499312; doi:10.1101/gr.240705.118)
Supplement: Supplemental Material [file supp_gr.240705.118_Supplemental_file_1.zip › contigs/annotated_contigs/DB110/contig.2.DB110_length_381_mean_cov_33.8320209974.docx]

**DB110_length_381_mean_cov_33.8320209974**

GGGATCATTGTAGAGATATGTCTCAATCCCCCTGTGGGCACAGCCTAGACAACAGTTACATCACCTCTGTTAACAGTGCAGAGATATGT
 >chr18:108130-108223 - E=2e-32 p=0e+00
CAAA|CACAATTGTTTTGTGATATGT|CTACACAATTGTTACATCACTTAGGTGAACAGGGCAGAGATATATCACAATAACCCCTTTAA
 >chr18:107857-108111 - E=2e-105
GCAGAGCGTAGACAAGAGTTACATCACCTGGGTGATCAGTGCAGAAATATGTGACAAGGCCCCTTTAAGCAGAGCCTAGAAAATAGTTA

CATCACCTGAGTGATCAGTGCAGAGATCTGTCACAATGCCCCTTTAGGCAGAGCTTAGACCAGAGTTACATCACCTGGGTGATCAGTGC

AGGGATATGTCACA|AAGCACCCTGTAG
