# Supplementary material for: Chromothripsis during telomere crisis is independent of NHEJ, and consistent with a replicative origin
Source: Genome Res. 2019 May;29(5):737–49. doi: 10.1101/gr.240705.118 (PMC6499312; doi:10.1101/gr.240705.118)
Supplement: Supplemental Material [file supp_gr.240705.118_Supplemental_file_1.zip › contigs/annotated_contigs/DB110/contig.2.DB110_length_388_mean_cov_8.50515463918.docx]

**DB110_length_388_mean_cov_8.50515463918**

ATAAACTTTCCCTCCTACCAGAGCCAATTAATGGGCTATAATCTTTCCAGTACATTGAAAATAAGGTACTTTCCTTGAAGTATGGATTA
 >chr3:181734824-181734967 - E=1e-74 p=5e-02
TACGTCCATGCCTTTTCATATATACAACAAAATGTTTTTAGATTATGCCACTCA|TTATTTTAA|TGAAAAGGCCATCCAACCTTGGGT
 >chr3:181731925-1817321
TCATATAATTTTTCTTCCACTTACCAGCTTGGAAATCCTGGACCTCAAGTTTCTTGTCTATAAGGATTGTAATAATAAAAAACTTCTAA
61 - E=1e-130
GATTGTGTTGAGTATTAAATGATCCTGTGGTACAGATCGTATTTCTAGTACATAGCACAATGCCTGGCATGCAGTAGGTGCCCAACAAA

TGTGTTACTTCACCAAGTCCCAATATGAAAAGTG
