# Supplementary material for: Chromothripsis during telomere crisis is independent of NHEJ, and consistent with a replicative origin
Source: Genome Res. 2019 May;29(5):737–49. doi: 10.1101/gr.240705.118 (PMC6499312; doi:10.1101/gr.240705.118)
Supplement: Supplemental Material [file supp_gr.240705.118_Supplemental_file_1.zip › contigs/annotated_contigs/DB110/contig.2.DB110_length_430_mean_cov_5.17441860465.docx]

**DB110_length_430_mean_cov_5.17441860465**

GGAAAGCTGGTGGAGTGAAACTCCAAATGAACAATTAATTCCACTTTCACTTCCCCCATCTACCTCCCTGAATACTACCTCCCTGCCCC
 >chr3:114342561-114342785 + E=2e-123
CACTCACCTATCTTCCTCCCCCCAACAAGCATGTTTGCTAGGCATGTCAAGGCTGACATCCATCGGTGGCAGTATGAAACCTATGCCTA

CAGGCTGCTCCTGAGCATAGCCTCAAGGCTCTGCCATTGTCCGTAG|G|ACAAAACAGAGGAAAAAAGATTTCATCCCCTTGAATTCTG
 >chr3:114343356-114343561 + E=7e-106
TTGGGAAGTTCATGTGAGTTCCCAGTAGACAAACTAAACTAAGCAATTTCAACAGTGTCACTGTTCCAGAATTTTGTGCAGAGGCTGCT

TATTAGCCAAATTTCATTTCTGCCCAGATCACTGCTGTACCATCCAGACATTTTCAGAAGGGAAGTTCAGGGAAGG
