# Supplementary material for: Chromothripsis during telomere crisis is independent of NHEJ, and consistent with a replicative origin
Source: Genome Res. 2019 May;29(5):737–49. doi: 10.1101/gr.240705.118 (PMC6499312; doi:10.1101/gr.240705.118)
Supplement: Supplemental Material [file supp_gr.240705.118_Supplemental_file_1.zip › contigs/annotated_contigs/DB110/contig.2.DB110_length_436_mean_cov_6.70412844037.docx]

**DB110_length_436_mean_cov_6.70412844037**

CCACACGGTTCAGGCATTTATTGATTTATGCATTGGAATTGTTTCCTTCATCAGGCAATACATTCTGCGACCGGGTTTTGCATATGTGC
 >chr2:2267025-2267225 - E=7e-109
AGCACCCCTTGTTTCTGGGATGTAAGCAGAGGGCGTCGTTAACCCTGGAGCTGGGAGGTGATTGGCCCTCCTCGCTCTCTGCCAGTAGG

GTTCCCCTTCTCTTCCATTTTC|C|GACTTTGGGCGGGCTAGAAAAGCTCCAACCAGCTGCAAAGACAGGTCAGATGTTATAGCTGCAG
 >chr2:2266315-2266554 - E=6e-121
GAATCATAATTTTTTTTTTTTTGAGATGGAGTCTTGCTCTGTCACCCAGGCTGGAGTGCAGTGGCACGATCTTGGCTCACTGCAAGCTC

TGCCTCCTGGGTTCATGCCATTCTGCTGCCTCAGCCTCCTGAGTAGCTGGGACCACAGGTGCCAGCCACCATGCCCGGCTAA
