# Supplementary material for: Chromothripsis during telomere crisis is independent of NHEJ, and consistent with a replicative origin
Source: Genome Res. 2019 May;29(5):737–49. doi: 10.1101/gr.240705.118 (PMC6499312; doi:10.1101/gr.240705.118)
Supplement: Supplemental Material [file supp_gr.240705.118_Supplemental_file_1.zip › contigs/annotated_contigs/DB110/contig.2.DB110_length_495_mean_cov_8.22626262626.docx]

**DB110_length_495_mean_cov_8.22626262626**

CTTCACCCTCAGAACACTGTGCAAGCACTGCATGCTCCAAGCCATTCATGACACACTACACTAGAGTGATGGAGGTGCCCCTTCTCCGT
 >chr8:136655501-136655809 + E=7e-174
ATACCCACTTAACATATGTAACGTGAGGGTCTCCTCCAGGCCAAAAGAATGAGAACCAGGGCATGCTGTCGTGGGAAACCAACCTCACT

CAGGGATACCAGCAAGGTTCTCTTTGGAAGTGGCATTTAGGTTAAGGGCTTGAGTTGGAAATAGCCTGGAGGACAGGAGAGAAGGGCCT

TCTGGCAGAAAGAGTAGCAAATATAAAAACTCATGCC|CACA|GAAAGCCAAAAAATTCCTCCAGAGCTATCTGATGTGTGCTGTTACC
 >chr8:136399250-136399441 + E=2e-100
ATCTTTAGGTTGAAAAAGGAATAATGAAACCCATATCTTCAGAAACTTAAGTCGTATTTTTCACCCTTTCCATCTCCCTTTACCCTATT

TATTAGAGAAATTGAATCTGAGCTGAGATTTTAATTAAAAATCTCATAATAA
