# Supplementary material for: Chromothripsis during telomere crisis is independent of NHEJ, and consistent with a replicative origin
Source: Genome Res. 2019 May;29(5):737–49. doi: 10.1101/gr.240705.118 (PMC6499312; doi:10.1101/gr.240705.118)
Supplement: Supplemental Material [file supp_gr.240705.118_Supplemental_file_1.zip › contigs/annotated_contigs/DB110/contig.2.DB110_length_502_mean_cov_14.03187251.docx]

**DB110_length_502_mean_cov_14.03187251**

CAAAATAACAGAAATCATAACAAACAGTCTCTGAGACCACAGTGCAATCAAATTAGAACTCAGGATTAAGAAACTCACTCAAAACTGCA
 >chr3:173102706-173102998 + E=3e-164 p=4e-02
CAACTACATGGAAACTGAACAACCTGCTCCTGAATGACTACTGGGTAAATAATGAAATTAAGGCAGAAATAGATAAGCTTTTTGAAACC

AATGAGAACAAAGACACAATGTACCAGAATTTCTGGGACACAGCTAAAGCAGTGTTTAGAGGGAAATTTGTAGTGCTAAATGCCCACAG

GAGGAAGCAAGAAAGATCTAAA|ATT|TTTAAATTCTTTTTGGCAGTTTGTATATCCTCATTTACTTGGAATTAACTACTGGGAAATTA
 >chr3:173068001-173068210 + E=4e-111
CTATGTTCTTTTGGTGATGTTTTGTCTCTTTGGTTTTTCATGTGTCTGGTTGCCTTTTACATTGATGTCTGCATGTTTGATGTATTCCA

GTCACCTATTCCAAAGTTTATGGACTTGTTTCTCTGTAAAAAGTCCTTTCCCAAA|CGAG
