# Supplementary material for: Chromothripsis during telomere crisis is independent of NHEJ, and consistent with a replicative origin
Source: Genome Res. 2019 May;29(5):737–49. doi: 10.1101/gr.240705.118 (PMC6499312; doi:10.1101/gr.240705.118)
Supplement: Supplemental Material [file supp_gr.240705.118_Supplemental_file_1.zip › contigs/annotated_contigs/DB110/contig.2.DB110_length_530_mean_cov_8.17735849057.docx]

**DB110_length_530_mean_cov_8.17735849057**

CATTGGGTTTAGGGCCCACCCAAATCATCCGAGATGACCTCGTCTTGAGATCCTTAACCTGCAAAGACCCTTTTTCCAAAAAAAGGTTA
 >chr11:68196091-68196405 + E=2e-177 p=0e+00
TGCTCACAGATTCTAGGCCTTAAGACATGGGTGTATCTTTCTGGGGGGCACTATCCAACCCCTTATACAATGAAAGACGGGAAGAGGGC

CAGGTGTGGTAGTTCACGCCTGTAATCTCAGCACTTTAGGAAGCTGAAGCGGGAGGATCACTTGAGCCCAGGAGTTTACAAGTAGCTAG

GCAACATGATGAGACCCCATTTCTACAAAAAGTGAAAAAAAAAAAAA|GCCAGGTGT|GGTGGCTCACACCTGTAATCCCAGCACTTTG
 >chr22:23851184-23851391 + E=6
GGAGGCTGAGGTGGGCGGTTCACGAGGTCAGGAGATCGAGACCATCCTGGCTAACACGGTGAAACCCTGTCTCTACTAAAAATACAAAA
e-113
AGTTAGCTGGGCGTGGTGGCGGGCGCCTACAGTCCCAGCTACTCGGGAGGCTGAGGCAGGAGAATGGTGTGAACCCGGGAGGCGGAG
