# Supplementary material for: Chromothripsis during telomere crisis is independent of NHEJ, and consistent with a replicative origin
Source: Genome Res. 2019 May;29(5):737–49. doi: 10.1101/gr.240705.118 (PMC6499312; doi:10.1101/gr.240705.118)
Supplement: Supplemental Material [file supp_gr.240705.118_Supplemental_file_1.zip › contigs/annotated_contigs/DB110/contig.2.DB110_length_546_mean_cov_6.10073260073.docx]

**DB110_length_546_mean_cov_6.10073260073**

ATTAGTTCTGTCCCTCTAGAGAACCCTGACTAATACAGATTTTGGTATCAGGAGTGGTTCTGGAGGAACAGAATATTAAGTTTGGAGGT
 >chr4:133278067-133278270 - E=2e-110 p=0e+00
TTTTTTGTTGGTTTTGGGGTTTCTGGAGTTGGCTGCTTAATATAATTAGACGTAAAATGCTAAGGATGGAGAACACTGATAGTCCTTGG

CATGAACTGTTTAAAGAGTTATGCA|CTGTTTAAAGAGTTA|TACTTTTTTTTAACTTAGAGACAGGGTCTCCCTCTGTCACCCAGGCT
 >chr4:133279690-133280018 - E=9e-183
GGAATGCAGTGGTGTGATCATATTTCATGGTAGCCTCAATCTTCTGAGCTCAAGCAATCCTCCCACCTTAGCCTCTCGAGCAGCTGGGA

CTACAGGTACATACCACCATGCCCTGCCATGCTATTTATTTTTGTTTTTGTCGAGACAATGTCTTTCTATGTTGCCTAGGCTGCTCCCA

AACTTATGGCCTTATGTGATCCTCCCACCTAGGTGATCCTCCTGCCTTGATCTCTCAAAGTGTTGGGATTACAGGCATGAGACACTACT

CCAAGCCTGTGCTA
