# Supplementary material for: Chromothripsis during telomere crisis is independent of NHEJ, and consistent with a replicative origin
Source: Genome Res. 2019 May;29(5):737–49. doi: 10.1101/gr.240705.118 (PMC6499312; doi:10.1101/gr.240705.118)
Supplement: Supplemental Material [file supp_gr.240705.118_Supplemental_file_1.zip › contigs/annotated_contigs/DB110/contig.2.DB110_length_546_mean_cov_9.89010989011.docx]

**DB110_length_546_mean_cov_9.89010989011**

GATTTGGCTAGGTATGGAATTCTAGTTTGGAAATAATTTGCTCTCAGAATTTTAAACACATTCTCCATTGCCTCATAGTTTTGGCGTAA
 >chr4:145948658-145948920 - E=5e-146 p=1e-02
ATGTTGAGAAATACAATGCCACTTTTAATTTCTGATGCTTTGCATGTGATCTATTTTTCTCTCAAGTAGCTTTTATAATCTCCTTATCC

TTGATATTCTGAAAATTCATGATGCTGTGCCTTATTGCATATGTGTTTCCTTTATTGTGCAGGGTGCTCAATGGTTTCTATCAA|AT|T

GGAAGCCTCACCTCCTTCCAGTCTTTGCACACATCACTTTCTCATTGAGGCCTATTCTGACCACCTTATTTAAAGCTGTACACATCTGC
>chr4:145947419-145947701 - E=4e-158
CCCTAATACTTCAGCTCTGTCTTTTCCTGCTCTGTTTTTTTCCCATGCACTTTTTATACTTATATGCTATGTAATCACTTATCGATTAC

GTTTATTACTAGAACATCACTTACAAGAGAACAGGGATTTTGTTGTTGTTCTTGGTTTCACTGATATATTCCAACCAATGAGAATAGTG

CCCTGCACAAAATA
