# Supplementary material for: Chromothripsis during telomere crisis is independent of NHEJ, and consistent with a replicative origin
Source: Genome Res. 2019 May;29(5):737–49. doi: 10.1101/gr.240705.118 (PMC6499312; doi:10.1101/gr.240705.118)
Supplement: Supplemental Material [file supp_gr.240705.118_Supplemental_file_1.zip › contigs/annotated_contigs/DB110/contig.2.DB110_length_556_mean_cov_10.4676258993.docx]

**DB110_length_556_mean_cov_10.4676258993**

CGGAAACAATTACAGAAGTTGCTTGACATCACAGGCCAGCCTGTCTGCACCAGTCTCTACTGTGCCCATACCATCAGTCTGAACTGCCC
 >chr11:68196395-68196838 - E=4e-252 p=0e+00
ACTTGTCTCTCCTCCTATTTACTTACTTTGAGACAGGGTCACACTCTGTCACCCAGGCTGGAGGGCAGTGGTGCGATCATGGCTCACTG

TAGCCTTGATCTCCGTGGGTCGAACAACCCTCCTGCCTCAGCCTCTTGAGTAGCTGGGACTACAGGCATGCACCACCATGCCTGGCTTT

TTTTTTTGAGACAGAGTCTTGCTCTATCGCCCAGGCTGGAGCGCAGTGGCATGATCTCGGCTCACTGCAAACTCCACCTCCCGGGTTCA

TGCCATTCTCCTGCCTTAGCCTCCCAAGTAGCTGGGACAATAGGCACCTGCCACTGTGCCTGGCTAAT|TTTCTTTTTTTTTTTTTTT|
 >chr18:74485167-744
GAGACGGAGTCTCACTCTGTCACCCAGGCTGGAGTGCAGTGGCATGGAGCGATCTCGGCTCACCGCAAGCTCCGCCTCCCGGGTTCACG
85294 - E=4e-46
CCATTCTCCTGCCTCAGCCTCCCG
