# Supplementary material for: Chromothripsis during telomere crisis is independent of NHEJ, and consistent with a replicative origin
Source: Genome Res. 2019 May;29(5):737–49. doi: 10.1101/gr.240705.118 (PMC6499312; doi:10.1101/gr.240705.118)
Supplement: Supplemental Material [file supp_gr.240705.118_Supplemental_file_1.zip › contigs/annotated_contigs/DB110/contig.2.DB110_length_556_mean_cov_8.63309352518.docx]

**DB110_length_556_mean_cov_8.63309352518**

TAACAAATGTCCTTTTTTTTTACAATTACCCCCCCTTTTTTTTTACAACTGCCCTATATTGAGAGAAAACTTAATATTATTTTTTGTTT
 >chr1:248324210-248324488 + E=8e-145
TTGTTTTTTTAATTGGCATAGATATTTTTTCTTATAGTTCATTCTGCATTGCACTGTTTTCTTTTTTTTAATTATTATTATACTTTAAG

TTTTAGAGTACATATGCACAATGTGCAAGTTAGTTACATATGTATACATGTGCCATGCTGGTGTGCTGCACCCAAAAACTTAATATTAT

ACTCA|TTT|CTCACACCATATACAAAAATCAATTCAAAATGAATTAAACTTGGATATAAGACCCGAAACTGTAAAGCTACTAGAAGAA
 >chr1:248528218-248528502 + E=3e-159
AATATAGGGAAAAGCTCTGTGACATTGGTTTGGGCAATGTTTTTCTGGATATAAACTCAAACTCCCATGGAACAAAAGCACAAATAAAC

AAATCAGATTATGCAAAACTAAAAAGGTTCTGCACAGTAAAGGAAACAATCAATAGAGTGAAGGGACAACCCACAGAATTAGATAAAGT

ATTTGTAAACTATATTATCTGATA
