# Supplementary material for: Chromothripsis during telomere crisis is independent of NHEJ, and consistent with a replicative origin
Source: Genome Res. 2019 May;29(5):737–49. doi: 10.1101/gr.240705.118 (PMC6499312; doi:10.1101/gr.240705.118)
Supplement: Supplemental Material [file supp_gr.240705.118_Supplemental_file_1.zip › contigs/annotated_contigs/DB110/contig.2.DB110_length_576_mean_cov_12.6371527778.docx]

**DB110_length_576_mean_cov_12.6371527778**

CTCATTGATTGATGATCATTTGGGCTGCTTCCATATTTTTGCAATTGTGAAGTGTGGTGCTGTAAACATGTGTAAGTATCTTTTGAATA
 >chr1:248746875-248747208 + E=1e-176
CAATGACTTATTTTCCTCTGGGTACACACCAAGTAGTGGGATTGCTGGATCAAATGGTAGATATACTTTTAGATCTTTAAGGAATCTCC

AGACTTTTCCATAGTGGTTGTACAAGTTTACATTTGCACCAGCAGTGTAAAAGAGTTCCCTTTTCAGCACATCCATACCAATGTCTATT

TTTTAAAGATTTTTTGATTATGGCCATTCTTGCAGGAGTGAGATGGTATTGCATTGTGGTTTT|GAA|ATAGTATTTCCAAATAATTCA
 >chr1:248529963-2485302
CAATAATTTTTCAACTTATTCCAAGAAGACAACTTCTCTAAAAACATGTAACATAATGGGCCTGGTCAGTTCTTGGTTGATGCTTTGAC
09 + E=2e-136
TCTTTCAGTGTTTCTTATTTTAAAACATGATTTTAAGTTTCAGTTTTGGCTGGGAGAAAATACCCTGTCTAAAGCTGTATTTCAGCCTC

TTGCTTTCAGATTTACCAAATTGTCTTTGTTTGACTTAAAAAAA
