# Supplementary material for: Chromothripsis during telomere crisis is independent of NHEJ, and consistent with a replicative origin
Source: Genome Res. 2019 May;29(5):737–49. doi: 10.1101/gr.240705.118 (PMC6499312; doi:10.1101/gr.240705.118)
Supplement: Supplemental Material [file supp_gr.240705.118_Supplemental_file_1.zip › contigs/annotated_contigs/DB110/contig.2.DB110_length_589_mean_cov_7.89473684211.docx]

**DB110_length_589_mean_cov_7.89473684211**

CTCTGGGATGTAAGAGAAGAATAAGGAAAGGCAACATCTATAAGACCCTATAAGTTACCCAGACATCTGGTTGGATTCTTTATGCTTAC
 >chrX:85508763-85509109 + E=1e-193
TGTATCAGGGCAATGATGCCAGAAAGGAAGTGACACAGATATTTGCAAATTATATTATTTGTAATTTTTGACACATTGGTTGATAAGCT

TCTAATATTTATAGAAAAATCTATTAAATCTTCACAAATATTTTTGTAAGATGTATTGGGTTTCTAAAAGTTTTCTATGGATATTATCA

ATCAGTGAATATTAAGTTAAAAAGAATCAGCTCCAGAAACAATCCTTGGGTATAATGGGAACATCAAACTTCTTGT|TAG|TGCCAATC
 >chrX:8551
TCCATTTTAAACAGGAGGCGAAGAAAATGCTCACTTTTGGCAATTTCTTCTCATCTTAGCATACAGAATAGGTAAATTATGGTAATAGT
0242-85510488 + E=2e-136
TCTATATGTCTCTTGATATAACTTGGTTCTTATTGTTTTTTAAATTGGAACTATTAATAGATTTACACTTTTGGTTTGGAATGAAAGAT

ATATTGAGCTGGGATGAGATTCGGTTAGAAGAAGGTGCAGTGTGTAGTAAAACCTGT
