# Supplementary material for: Chromothripsis during telomere crisis is independent of NHEJ, and consistent with a replicative origin
Source: Genome Res. 2019 May;29(5):737–49. doi: 10.1101/gr.240705.118 (PMC6499312; doi:10.1101/gr.240705.118)
Supplement: Supplemental Material [file supp_gr.240705.118_Supplemental_file_1.zip › contigs/annotated_contigs/DB110/contig.2.DB110_length_600_mean_cov_12.0516666667.docx]

**DB110_length_600_mean_cov_12.0516666667**

AGAAATATCTAAAGTTATGTGACAAATTTGCATATATCTATTAAATTTTTACAGATCAGACTATATATCATGGACCCTGATTTATATAT
 >chr5:8749044-8749528 + E=9e-277
AAAAATTCATCATGACAACTTTATAAAATGCATCTCGACTAAACAGCTAACATTCTGAATTCTGCTTTATGCAGTGTGTTTGGGTCATA

CATAGAGGCCGTGAAAACACTGAGCTTGGCTTAAATACACTGGTTTCTCTTATCTACGTCACCATCAGTTTAAAGTAAAATTTGGAAGA

AAAAATATATTGAAGAATAATATAAGTAGGCACTCAAACAATGTGCCACACACTTCCATCTTAAAACAAAGTTAATATGTAGAATGGCA

CAGGCTTCAGACATTTTCATATTATCTGGTAACACTTTTCTTTAACTACAGCATTTAAAATATATAGGCAGGTTAATATAAGAAATATC

ATTCCAAAGAGTAAATTTTCTCTTTAAAATTTTTTAAT|G|TGATGCCTCCAGCTTTGTTCTTTTGGCTTAGGATTGACTTGGCAATGC
 >chr4:150053107-150053224 - E=1e-58
GGGCTCTTTTTTGGTTCCATATGAACTTTAAAGTAGTTTTTTCCAATTCTGTGAAGAAAGTCATTGGT
