# Supplementary material for: Chromothripsis during telomere crisis is independent of NHEJ, and consistent with a replicative origin
Source: Genome Res. 2019 May;29(5):737–49. doi: 10.1101/gr.240705.118 (PMC6499312; doi:10.1101/gr.240705.118)
Supplement: Supplemental Material [file supp_gr.240705.118_Supplemental_file_1.zip › contigs/annotated_contigs/DB110/contig.2.DB110_length_687_mean_cov_13.5371179039.docx]

**DB110_length_687_mean_cov_13.5371179039**

CAAGACTTGGCAATGCCTCACATAGGGAGGCGGACAGGGGAGGATTTCAGGATGAGCTGAGGTTTTGAGCCCATGCGATGTGGAGGAGG
 >chr20:14580457-14580847 + E=6e-223
GTGGCACTGGCAACTGAGATAGCTATCACTGAGTAGGAACAGGACTGTGAATTGAATTAAGGTGGTGAAAGGACATAGAGGTTGACATT

TTCAGCAGTCACTAGGAATTAAGAGGCTAGAGTATAGGAGAACTCAGCTACTGAGTCCACCACACTGCTGGATAGTGCTGCCATGATAG

GAAATTAATATTTTCAGGAACAAATGATTAAAGTTTTGAAAATGTGGCAAGGCCGTCATCATCAATGCAATGCACACAAAAAAACTGGA

TGTGACTAATCGGATGTCAGTCAAATCACTTAA|T|TTTAATGGAGGAAGGGGCCCACAGTGGCAAAAAGTACCTCAGGGCCGTGAAAG
 >chr20:14582300-14582598 + E=2e-167
TCATAACTTAGCACTGGAAATCACTCCTTACTTAGGTTCTTTAAACCTCTTCTGGTGTCTAGGTCATAATGTTTTCTTCATGCATTTTG

TTGATTCATTAGCCAACTAATTTCTAGCTTATGTCAATTAAAATAAAACTGACCTTTGTTCCCTCATACAATAAGATTTTTCTAACACC

CCCTTCCTAAATAGTTAATTTCCTTTTCTGGGTTACTTCTGTACCATATGATTAAGCATCTCGCAC
