# Supplementary material for: Chromothripsis during telomere crisis is independent of NHEJ, and consistent with a replicative origin
Source: Genome Res. 2019 May;29(5):737–49. doi: 10.1101/gr.240705.118 (PMC6499312; doi:10.1101/gr.240705.118)
Supplement: Supplemental Material [file supp_gr.240705.118_Supplemental_file_1.zip › contigs/annotated_contigs/DB110/contig.2.DB110_length_715_mean_cov_8.67832167832.docx]

**DB110_length_715_mean_cov_8.67832167832**

TGTGAAACTCGTTAATCAGTATCTGTGGAATGGGAATAACCATAGCTATTAGAATGTGTACCTTCTACACCCGGTAGCTCTGTCACCAT
 >chr1:248530927-248531243 - E=2e-178
CATGGAGCTGTTTGTTTACTGCAGTTACATGTCTCCTGTCACACTGGAATGCTCATTGTTCTTTTTCCTTCCTTTACCGAAATATAATT

GCAAAATTAAATAAATATGTTTTGAAACAAGCCTAAATTGAACAAATTATTCTGAAAAACAAAATAAAAGATAAGTTTTATAAAAATTG

AGTAAAATGTGGATGGTAACAACATTTTTTAAAATTATCAAACAGGCCA|AAGCTTTTAC|TTGTGGCGGCAGCCTGGCTGGGAGGGTC
 >chr1:248604910-248605299 +
TATCGATGGTTTCTTGCTCACCCCCGTCACCATGCAGTTCCCCTTCTGTGCCTCTCGGGAGATCAACCACTTCTTCTGCGAGGTGCCTG
 E=3e-222
CCCTTCTGAAGCTCTCCTGCACGGACACATCAGCCTACGAGACAGCCATGTATGTCTGCTGTATTATGATGCTCCTCATCCCTTTCTCT

GTGATCTCGGGCTCTTACACAAGAATTCTCATTACTGTTTATAGGATGAGCGAGGCAGAGGGGAGGCGAAAGGCTGTGGCCACCTGCTC

CTCACACATGGTGGTTGTCAGCCTCTTCTATGGGGCTGCCATGTACACATACGTGCTGCCTCATTCTTACCACACCCCTGAGCAGGACA

AAGCT
