# Supplementary material for: Chromothripsis during telomere crisis is independent of NHEJ, and consistent with a replicative origin
Source: Genome Res. 2019 May;29(5):737–49. doi: 10.1101/gr.240705.118 (PMC6499312; doi:10.1101/gr.240705.118)
Supplement: Supplemental Material [file supp_gr.240705.118_Supplemental_file_1.zip › contigs/annotated_contigs/DB110/contig.2.DB110_length_752_mean_cov_7.96276595745.docx]

**DB110_length_752_mean_cov_7.96276595745**

TGGTGACTTAACAGTTTTAAAAAGGAAGATCAAGGCAGAAAATTAAAAAGTAAACAGTTGAATTAGTATGGAAAAAGTAATCATGCAGT
 >chr1:83330470-83330838 + E=1e-209
CATTTCTTTTAGAAATGGCCCAAACAATAGGCCAGACATATTTCTAGATTTTTACAAATTTGTTTTTAGTAATTCTAGCGCAGTGGTTC

ACAGAGTGTGGTCCACAGTGTTCCTCCAGGTGGTCAGTAACCTGGTCTCTTGTGGGGCTGACATTTCTCGTGTGAATATGTGGTCCTTC

TGTTTCTGGCAACTATTAAAAATGTACTTATTTTATGTAATCAAAACCAGCCATAATTTTAGGAGCATCTCATTCAAAACTTCATTGTT

CCTCTCATCA|AT|ATATATATATATATATATATAGTGATGTTTTAAGTTACTTCTGCATAAGTTTTCATTCATATGTGGACATGTCTC
 >chr1:83331651-83332037 + E=2e-220
CTCTGCCATTAAAACATGCTAGAAGTCACCTCTTCATATGCAGAACGCCATTTATAGCTATTCAAAACCAATCAGTGTCAACCTACCTA

TGCAAGAACATCCTAATGATAAAGTAAATAATAAGGTATCTCAGACTTGGGTAAGAAAAATTGACATAATCACATCATTAGAAATATCA

GGCTTGAATATTGGGATTTGAATAATTCAAATAAGATAGTAAACACAGCAAAGCTTTTTTTTTAACCCAGACAAGAATAAACTTTTATA

TATTTAGAAACATAATCCATAGCTAATGAGCCCAGTAGTTTA
