# Supplementary material for: Chromothripsis during telomere crisis is independent of NHEJ, and consistent with a replicative origin
Source: Genome Res. 2019 May;29(5):737–49. doi: 10.1101/gr.240705.118 (PMC6499312; doi:10.1101/gr.240705.118)
Supplement: Supplemental Material [file supp_gr.240705.118_Supplemental_file_1.zip › contigs/annotated_contigs/DB110/contig.2.DB110_length_755_mean_cov_13.2768211921.docx]

**DB110_length_755_mean_cov_13.2768211921**

CTATTTTGGTTCTTCTTTGTGGAGGAAGTTAGTACTAGATGCCTCTAGTCAGTTATCTGGAAGCTAAACATGCTTCTTTAAATATTATA
 >chr6:103804537-103804974 - E=4e-245 p=2e-02
ATTTTAATTTTAAAACACTTTAGAGTTTACAGAGACATATGTAATTATTTCTTATTATTATTTTAATGGAAGTTCCGTGTGATACATAC

TGAGAAACATTTTCCACTGTGGGCCTTACATAAATGTAACATCAAATTCTTCATCTCCAGTCCTCTTGCTAAGCAAAAATTGCCCAACA

GCACCTGCTAAACTACAAGGAAAATTCCTTCTTCTTCTGAGAAGTGTTTTGGTCCAACTATTCGTAAAACCATCTGGACTTTTGCTGTT

AAAGCTCTATAAAAAGTTATCTTCCTTTCCCCAAGGAAAAACATCCCTCAGAGTTATTCTCATGCTGCATCATTTTAGA|TT|TAAATA
 >chr6:1
AGAAAATTGATTGCACTTCACATACTAGCAATAAATTGTAAGAAGAAATTATATTTAAACAACCTTTTGTACCAGTAAGAAAATGATAA
03802254-103802516 - E=9e-146
CATAACTAAGATGAGTGTAGTAAAAAATAGGTAGGATCTTTGTGAGGACAATTATAAAATGCTACTAAAATATAATGAAAATGAATAAA

TATATAATTTCTTCTTAATGTATTTAAGAATAAATTATTTCATAAATATTCTCCAAATTTAGCTATAAGCTTATGC|GTTTTCTGATAG

AACTCTTGTTTTGTTTTTGATTACTTTTCATGTCTTTATTGGAATT
