# Supplementary material for: Chromothripsis during telomere crisis is independent of NHEJ, and consistent with a replicative origin
Source: Genome Res. 2019 May;29(5):737–49. doi: 10.1101/gr.240705.118 (PMC6499312; doi:10.1101/gr.240705.118)
Supplement: Supplemental Material [file supp_gr.240705.118_Supplemental_file_1.zip › contigs/annotated_contigs/DB110/contig.2.DB110_length_760_mean_cov_10.2289473684.docx]

**DB110_length_760_mean_cov_10.2289473684**

GTCTGAGCAAGGCCTCGTGGCCAGCAGGCTGGTCCACGGACTTCTCTGGCCGGACCTTAACTACCACCTCCCCTCGCCACTCTGTCGTT
 >chr14:104798697-104799077 - E=9e-208
CACCAGAGCATGGGTTGGGTGGCAGGTTCTAGCCCCACGACCATGCCTTCCTGCTCCTGCTGGACAAGCCTTGCCTTCTCAAGCCTGGG

CTCCCCAAACAGCCAAGGGAGGAAAAGGAAATCTTGGTCCCTCTGGGAAGACTGGGACGGTCCAGCTCAGGCTGTGACTTTGCTCCCAG

GAGGCAGGCAGCTCCATCCCAAGCATTCTCAGGGTTCATGCAGACAGCATGTCTGGAGCCCGGGTCTCACGCTCACATCTGATCAAGGA

GAACAAGGGGCCTTTCCTGAAGGG|AAAG|TCAAAATATGACTGCTCATTGACTGTGTACCCAAGAGCTCTGATGGAGATGTACAAAGA
 >chr14:104797365-104797741 - E=2e-211
GATGAACATTGTTCTCATGCCTGGTAATACAACATCCTCTCGGTAGCCCATGCATCAGGAGGCATTTCAACTTTCAAGTCTCATTATTT

AAGAAATACATTTTGTAAGACTGTAGCTGCCATAGATAGTGATTCTTCAAATGGATCTGGGAAAAGTAAAATCTTCTGGAGAAGATTAA

TCCTTCTAGATGCCATGAAGAACATTTGTGATTGAAGATATATCAACATTGACAGGAGCTTAGAAGAAGTTGGCTCCAACCATCATGGA

TGACTTTGAGGGGTTCAGGGCTTCAGCGGAGGAAGGAAATGAAGATGTGG
