# Supplementary material for: Chromothripsis during telomere crisis is independent of NHEJ, and consistent with a replicative origin
Source: Genome Res. 2019 May;29(5):737–49. doi: 10.1101/gr.240705.118 (PMC6499312; doi:10.1101/gr.240705.118)
Supplement: Supplemental Material [file supp_gr.240705.118_Supplemental_file_1.zip › contigs/annotated_contigs/DB110/contig.2.DB110_length_764_mean_cov_12.4947643979.docx]

**DB110_length_764_mean_cov_12.4947643979**

GGGACATACAGAGAAGAACAACAGATACTATGGACTCCAAAATAGGGGAGGATGGGAAGGCAGGATGAGCGTTGACAAATTATCTATAG
 >chr5:8677498-8677842 - E=4e-195 p=6e-03
TGTACAGTGTTCACTATTCGGGTGATGGTACACCAAAAGCCAAGACTTCAACACTATGTAATACATGCTTGCAACAAAACTGCACTGGT

ACCCCCTGAATCTGATAAAATAAAATAAAAATAGAAAAGTCTTATTCTAGCATGATGGTACAAGTCTTTTAACTTTTTTCCATTTCATT

TTAAGCTATTTCACCACTTAATGTATGGAAAAAAAGTTGACTCCGAATTTCATCTTCAATACTATAATTTTGA|TGGA|GGTTGCAATG
 >chr5:8748363
AGTCGAGATCACACTACTGTACTCCAGCCTGGGCAACAGAGCAAGACTCCATCTCAAACAAACAAACAAACTCAAAAATGTATTCTTGT
-8748787 - E=3e-243
AAGTCATTACAATTTATTTTGTGCACATTCTACAAGACTTTCATACTATGAACATTAAGAACATTAGATTGAGGGTAGGGGTTCGTGAC

CAGCCTAGACAACATAGTGAGATCCCATCTCTACAAAAAAAATTTGGAAAAAAATTAGCTTGGGATGATGACACATGTTTATAGTCTCA

GCTACTGAGAAATCAGAGGTCGGAGGATTGTCGAGCCCAGGAGATTGAGACTACAGTGAGTTATGGTTGTGCCACTGAACTTCCATCTG

GGCAACAGAGTGAGCCCTGTCTCCAAAAAGAAAATAAGAATATTAATAACATAT
