# Supplementary material for: Chromothripsis during telomere crisis is independent of NHEJ, and consistent with a replicative origin
Source: Genome Res. 2019 May;29(5):737–49. doi: 10.1101/gr.240705.118 (PMC6499312; doi:10.1101/gr.240705.118)
Supplement: Supplemental Material [file supp_gr.240705.118_Supplemental_file_1.zip › contigs/annotated_contigs/DB110/contig.2.DB110_length_847_mean_cov_8.71900826446.docx]

**DB110_length_847_mean_cov_8.71900826446**

TTCCATTTTTTATTGCATTTATTTATTAAACTTTCCTCCACTCTGGGCCGGAGGTGACTATATATATGCGTGTGTGTGTGTATATATAT
 >chr2:212573882-212574392 + E=7e-280 p=3e-02
ATATACACACACATATATATATATATATACATACATATATATATACATATATATATATGAGAGAGAGAGAGAGAGTAATTTTATAACTA

GTAGAAATCTAGAGTTTTAATAATTATGTTCTATGATGAACCAAGACTAAAATAACTATTCTGAACCTCACTAGATAGAACATTTTTGA

AACCTTTCTACCCATGTTTTATCTGTAAGAATTTTAGCCTTGGCAGGTCACTTTTGGTGGTTTGCAATCTTTCTACTATCACAAAATTC

TTGCAGTGTTTCTCAATGGGGGCAGCACCACCCCACGAACAGGAAGTTGGCACATCTGGAGAGCTGTTTCAGTTGTAACAGAGACTGGG

TGATCCTATAGTTTACAGCAGTTATCACAAGGTTGTTAACTGTCTCTTAAATCAAAGGATT|GA|TACAACAATTATGGGGTGGCAGCT
 >chr2:212575779-212576120
ACATACATACTATTATACTATCTAACATGGGGCTGTATTAAAAGCTACCTATTAAAATAAACAGATATTTAGCAATAATACATATAATA
 + E=3e-187
ATTGATTTGAATGAAAAGAAACACTTTGTTCTATTGTTCTTGCCATTCCTATTTGGACAGATTTATTCATAATATGTCAAATTAGTTTG

CTTTTAGAAATGATCTGATACATGTTCAGAGTAAGATTTTGAATTAAAATATATGAGAGGTATTCTTTATCTGTAGCCTGCAGCACTAA

CATTTAGGGATTAAGTAGTAATATAGACAAATAGATAACATACAATGT
