# Supplementary material for: Chromothripsis during telomere crisis is independent of NHEJ, and consistent with a replicative origin
Source: Genome Res. 2019 May;29(5):737–49. doi: 10.1101/gr.240705.118 (PMC6499312; doi:10.1101/gr.240705.118)
Supplement: Supplemental Material [file supp_gr.240705.118_Supplemental_file_1.zip › contigs/annotated_contigs/DB110/contig.3.DB110_length_1270_mean_cov_13.9094488189.docx]

**DB110_length_1270_mean_cov_13.9094488189**

TTGCTTACTCCCATTTTCAATCCATCAACAGATCTTGTTATTTTCATGCCTTTCTGTCCACTTTCCTGTCTCCACCATAACTGCCACCA
 >chr8:136740637-136741002 + E=2e-201
AGGCAAAGCTCCCACTAAAGCCCCCACAGCCCATCTCCACCTGCACATCCACTGAGCAGACGCCATCTCTGCCTGCACATCCACCTAGC

GGACCCCATCTCCGCCTGCACATCCACTGAGCAGACCTTCCAACTGACACCCACCTGCTCCCATCACTCCCCTGCATAGCCCCCCCCAG

GGCCCCCTCCTGCTCTTAGAGCAGAACCCGACTTCTTGCCCCATCTATGAAATCCTGAACTGTCAGTCAAGGTTCTCAGTTGCAAACCA

CAGA|GCACC|TTTCCCTTCCCCTTCCCCTTCCCCTTCCTCTTGCCCTTCCCGTTCCCCTTCTCCTTCCTTCCTTCCTTCCTTCCTTCC
 >chr8:136815151-136815600 + E=7e-258
TTCCTTCCTTCCTTCCTTCCTTCCTTCCTTCCTTCTTTCATAAATCAGCCACTAATGGACTCTCCACTGTGTGCTAGGTTGTTGCTCAG

AAACAAATAGTGACTGGAGAGAGCAGTGGATGAAACAAGCTGAAAAAGATATGGTGCCTCCCTCAAAAGCCCTAAACTTGAGTAAATAG

GGAACACTTCTGTGGGAATGTCTTGCCAGAGAACCCCAGAGACGATCAAACAACTTGAGTATAATTTCAAAGCAGATTTATATCCATGT

ATTCACATTGTTGTGTGGTTTTGCGAAATCCACAGTAATTGTATATTTTTGTATTCTTTTTCTTTTCTCTCTCTCTCTTTTTTTTGAGA

CAGAGT|TTTG|ATAAGTTAAATGTGCATAGTGTAATTTAATGTTAATTACTAAAAGAGTGTAACCAGTCTATACATCTCTAATCCAAT
 >chr8:144381550-144382015 + E=2e-264
AAAAAGAATAAAACACTGTGATTTAAAATGTAGTCCCAGCTGCTTGGGAGACTGAGGCAGGAGGATCTCGGCTTGGATCCTCAGACCAG

GAGTTTGAGGCAGCAGTGAGCTATGATTGTGCCGCTGCACTCCAGACTGGACAACACAGTGAGAGCCCATAAAATAAATAAATGAACTG

AACACAAACTTCTGTCAATTAAAAAATAAGAACGAAATCAGGCTGGGCCTGAGCGCTGTGGCTCACACCTGTAATCCCAGCACTTTGGA

GGCCAAGGTGGGTGGATCGCTTAAGCCCAGGAGTTCGAAACCAGCCTGGGCAACATAGGGAGAACCTGTCTCTACAAAAAATACAGTAA

GTAAGCCTATAGTTCCAGCCACCCAGGA
