# Supplementary material for: Chromothripsis during telomere crisis is independent of NHEJ, and consistent with a replicative origin
Source: Genome Res. 2019 May;29(5):737–49. doi: 10.1101/gr.240705.118 (PMC6499312; doi:10.1101/gr.240705.118)
Supplement: Supplemental Material [file supp_gr.240705.118_Supplemental_file_1.zip › contigs/annotated_contigs/DB110/contig.4.DB110_length_1603_mean_cov_17.5271366188.docx]

**DB110_length_1603_mean_cov_17.5271366188**

CCAGAAATAAAACCAAATACTACAGCCAACTGATCTTCAACAAAGCAAACAAAAACATAAAAGTGGGGAAAGGACACCCTATTCAACAA
 >chr1:248747460-248747798 - E=5e-191
ATGGTGCTGAGAAAATTGGCAAGCCACATGTAAAGAATGAAACTGGAACCTCATCTCTAACCTTATAGAAAAAACAACTCAGGATAGAT

CAAAGACTTAAACCTAAGACCTGAAATTATTAAAAATCTGGAAAATAACGTCAGAAAATGTTAACTTGTAGACATTGGCTTAGGCAAAG

ACTTCATGACCAAGAACCCAAAAGCAAATGCAATAAAAACAAAAATAAATGGGACTTAATTAAACTAAAAA|C|ATATATATATATATA
 >chr1:24874169
TATATATATATATGTTTGTTTCTCTTGTCTGATTGCTCTAGCTAGAACTTCCAGTAATATGTTGAATAACAATGGTAACAATAGTTGCA
4-248742286 - E=0e+00
CTAAAGTGCAAGACAAAATCTCCTTTAATTTTCTGTCCACTTTCCTCAAGCAGAAGGAGTCTTGCCCCATAGCCACCATAGCTGGTAAT

ATGCTGAGTCTCACCTGAAGCCAGCATGTCTCAGAGTCTCACCCAAGGCCCTCATGACTCTGGCTGGTATCCAACCCTGATGTGGCTGA

GCTGGTATCCAAGATGCAAGACAAAGTCCTCCTCACTCTTCCCTCTCCTCTCCTAAAGCAGAGGAAAGAGGCCTCTTTTGGAGCCACAA

GCCGTGGAGGCTGAGGTTAGGGGATGAGTTTAGCTGCCCTGGCTGGTGTTTTAACAGGTCATGTGTCCCCTGAGTCCACTGGCTCTGGG

CCCAGATTAGCATTAGGATTTGCAGCTCTTGTGGTCTAGACTGCCCTTCAGGTTTGTCTGTGGCTCCAGAGCCACTTTAGCCCTTCATG

GTGAGGCTTGGGTAACTCAAGTTCCAAACACTGGGATTCGCAA|GTCTCAAGTCTCTTTGTTAGCTTTTTGCCTCAATACTGTCAGTGG
 >chr1:248777253-248777554 - E=1e-164
GATGCTGAAATCTCCCATTATTATTGTATGGGAGTCTGAGTATCTTTGTAGGTCTTTAAGAACTTGCTTCATGAACCTGGGTGCTTCTG

TGTTGTATGCATAGATAGCTAGGATAGTTAGATCTTGTTGAATGGAAACCTGTAGCATTATATTATGGCCATCTTTGTCTTTTGTGACC

TTTGCTGGTTTGAAGTCTGTTTTGCCTGAAAACTAGAATCGAAACCCTTGCTTCTTTGTTTCCATTTGCTTGGTAG|ATT|CCCTTCCA
 >chr1:2487
GCAATGGCTGATTTAAATGCACCCTTCATGGTGGGTGTCAGCTGAATTTGGTCTGGGTCTTCTTTCTGCTATAACAAGGGCATTGCTGA
41322-248741695 - E=4e-212
GTTCAGTGTCTTAGAATTGCTGGCTCTCCCTCTCCCCAGTGCACAGAGAAGCTCTCCATACCACACCTCCACTGCTGGGGGATGAAGGA

GTGGTAGTGTCAGTGATGCAATACTGTTTTTCTAACTTCTTCAATGCCTCATTCAGAAATATGAATTTAACTCCATGTACTGTGAGTGC

TCACCCGATTTTTGGTTCTTGTAAAGGTGTTTTTTGTGTAGGTAGTTTTTAAATTGGTGTTCTTGCAGGGGGATGATCAGTGGAAACTT

CTATTC
