# Supplementary material for: Chromothripsis during telomere crisis is independent of NHEJ, and consistent with a replicative origin
Source: Genome Res. 2019 May;29(5):737–49. doi: 10.1101/gr.240705.118 (PMC6499312; doi:10.1101/gr.240705.118)
Supplement: Supplemental Material [file supp_gr.240705.118_Supplemental_file_1.zip › contigs/annotated_contigs/DB111/contig.2.DB111_length_236_mean_cov_4.44915254237.docx]

**DB111_length_236_mean_cov_4.44915254237**

AATGTACTTAATACTTACCTGAGCCATTATGTTTAGGGCATTGTGTTAGTAACTGTGGATGATATGAAGAAAGGCCGTAGAAAGGTTTT
 >chr18:52425514-52425643 + E=1e-66
TCCTTTGAAGAACTGACAGTCTCAATTAAAGAGATTAA|AC|AATGATGTCTCTCTCTCCAGCTTGCAAGCGACCAAAAACAATTAAAA
 >chr18:52425821-52425934 + E=2e-48
AAAAAAAGGAACAACGACAGAGCTAAGAGACCCTACCAGACTTTAATACCAAGTTTTGCC
