# Supplementary material for: Chromothripsis during telomere crisis is independent of NHEJ, and consistent with a replicative origin
Source: Genome Res. 2019 May;29(5):737–49. doi: 10.1101/gr.240705.118 (PMC6499312; doi:10.1101/gr.240705.118)
Supplement: Supplemental Material [file supp_gr.240705.118_Supplemental_file_1.zip › contigs/annotated_contigs/DB111/contig.2.DB111_length_251_mean_cov_8.97211155378.docx]

**DB111_length_251_mean_cov_8.97211155378**

AGCCTCACATGCATTAGCTATTTATCCTGATGC|ATGTATATATAT|ATATATATATATATATATATATATATATATGAGGTACAGGAG
 >chr14:98375965-98375998 + E=1e-08 p=6e-03 >chr14:98376808-98377014 + E=4e-104
GAAAAACAGCATTTATAATGGTGATGTTGAATTGACTGATACTATCTGTGCACTAGGGATCCCCATGGCAATGGGGCAAAAATGCTATC

ATGATAGCTTATCTATCCAGAATTTAGTGAGTTGTCCAATTGTCAGAGAAATTCTAAGAGATCTGTCTTTAAATG
