# Supplementary material for: Chromothripsis during telomere crisis is independent of NHEJ, and consistent with a replicative origin
Source: Genome Res. 2019 May;29(5):737–49. doi: 10.1101/gr.240705.118 (PMC6499312; doi:10.1101/gr.240705.118)
Supplement: Supplemental Material [file supp_gr.240705.118_Supplemental_file_1.zip › contigs/annotated_contigs/DB111/contig.2.DB111_length_253_mean_cov_1.18577075099.docx]

**DB111_length_253_mean_cov_1.18577075099**

TTACCTCTTACAGTTGGCTTGTGACTTCCTCCCACCCCAGGCTGGACGTTGTTTCATGAAAGTGTTTGGCTTCTCCGATG|GG|TTTTG
 >chr3:71473981-71474063 - E=4e-35 >chr3:
AAGGCTGTTTCTGCTTCTGGTTCCCATTCTACTAGATGAGTATTTGCCCTCTGGGTCTCCGTGATTAGAGTATAGAGTGGTCTGGCCAT
38483446-38483613 + E=2e-89
CTCGCTCTATCTGGGGATCCATAGTCAGCAAAACCCAGTGATTCCAAGGAACCCCTGCAATTGTTTTAATG|CTACCT
