# Supplementary material for: Chromothripsis during telomere crisis is independent of NHEJ, and consistent with a replicative origin
Source: Genome Res. 2019 May;29(5):737–49. doi: 10.1101/gr.240705.118 (PMC6499312; doi:10.1101/gr.240705.118)
Supplement: Supplemental Material [file supp_gr.240705.118_Supplemental_file_1.zip › contigs/annotated_contigs/DB111/contig.2.DB111_length_322_mean_cov_1.86335403727.docx]

**DB111_length_322_mean_cov_1.86335403727**

AAACAATCTTTCCAAATGACAAAAATCCCAGAAATTACAAATTCTTTTAAAATTGACAAATACAAAAAAATCACAAAATCTAGAAATAT
 >chr18:52425078-52425183 + E=5e-47 p=0e+00
TTTTATTAGTAAATT|TTATTAGTTGTTAA|TTTTATTAGTAAATTATCTGGCATGCCTCTACAATACTTGCTTTTCTTTTTTTGAGAT
 >chr18:52425168-52425345 + E=2e-90
GGAGTTTCACTCTTGTTGCCTAGGCTGGAGTGCAGTGGTGCGCTCTCGGCTCTGTTGAGTATTAGGTGTAAGATCAATGCTGTGCTTTG

TAACCTAGGTTTTTTTTAGCCTGTATTTT|TTTGGCCATGGGTTGCCCTCCTTTTGAT
