# Supplementary material for: Chromothripsis during telomere crisis is independent of NHEJ, and consistent with a replicative origin
Source: Genome Res. 2019 May;29(5):737–49. doi: 10.1101/gr.240705.118 (PMC6499312; doi:10.1101/gr.240705.118)
Supplement: Supplemental Material [file supp_gr.240705.118_Supplemental_file_1.zip › contigs/annotated_contigs/DB111/contig.2.DB111_length_371_mean_cov_6.42587601078.docx]

**DB111_length_371_mean_cov_6.42587601078**

GTTAATAGGGAGATAAGTGAGGATCATCCTCGAGGCATCTCAAAATCTGTGCTCATCGGCCATGTCCTCCTGAACTTTTGCCTCTGAGG
 >chr5:13310227-13310352 - E=8e-64 p=1e-02
GAACACAAATAGTCTCTCTCTCTCTCTCCCCCTCTT|CATTGTGA|GGTCTGTTCAATCAAAGGCCCAACCCTGCAGTGCTGGGCCTCC
 >chr1:27332292-27332530 + E=6e-132
TGGCACATTCTGCGCCTCTGGTCATGTCCTCCACACACTCTTGGAGGCCCCACCCCACCCCGTGAGGCCCAGTCCCTTCCCTTCTGGCC

AGGGTCTGAGTCAGTTACAAGGCAGCCAGGTGGGATAGGCGTGAGCCAGGAGAGGTTGCACGGGGGTCACGTAGTAATTGACAGTGGCT

GGCACAACCCCGTCAGT
