# Supplementary material for: Chromothripsis during telomere crisis is independent of NHEJ, and consistent with a replicative origin
Source: Genome Res. 2019 May;29(5):737–49. doi: 10.1101/gr.240705.118 (PMC6499312; doi:10.1101/gr.240705.118)
Supplement: Supplemental Material [file supp_gr.240705.118_Supplemental_file_1.zip › contigs/annotated_contigs/DB111/contig.2.DB111_length_431_mean_cov_11.4106728538.docx]

**DB111_length_431_mean_cov_11.4106728538**

TGGCACACACCTGTAGTCCTAGCTACTTGGAAGGCTGAAGCAGGCGGATCCCTTGAGCCTAAGAAGTTGAGGCTATAGTGGGCTATGAT
 >chr7:98435467-98435702 + E=7e-115
TGTGCCACTGTACTCCAGCCTGGTGACAGAGCAAGACCCCATCTCAAAAAAAAAAAAGGAGGAAAGCATTTATTGCAAGAGAGACAACA

CTCTGATCTTAAAGTCTGCAAGCTGCTCAATAGTAAATTTTTTTTTTTTT|GATA|CGGCCTTGACTCAACCTCTTCTACCGCTGCAAG
 >chr7:98444774-98444972 + E=1e-1
GCCTTTTCCTCAGTGAAAGTTTCACAGATGGGTTTCGATAAAATAAGGTGATATTTATAAAGCACTCTGCAATCACTCAGTATTAGTCC
07
TGCCCCCCATCCAGCCCCCTGCCAGCCAGCAGGGACTAGGATGGCCTTATAAAGGGCGCAAGAACTGCAGAGACAA|N
