# Supplementary material for: Chromothripsis during telomere crisis is independent of NHEJ, and consistent with a replicative origin
Source: Genome Res. 2019 May;29(5):737–49. doi: 10.1101/gr.240705.118 (PMC6499312; doi:10.1101/gr.240705.118)
Supplement: Supplemental Material [file supp_gr.240705.118_Supplemental_file_1.zip › contigs/annotated_contigs/DB111/contig.2.DB111_length_448_mean_cov_3.68303571429.docx]

**DB111_length_448_mean_cov_3.68303571429**

CAAGCTCTGGCGATGTTGCAAGCACTGGGTAGGGCGCCTTCCCTATCTCATCTGTAATCCCCACCATAACCTTGCAATGTTACCAATGA
 >chr3:71473480-71473681 + E=2e-106
GGAAACAAGTGCTTTGCCCAGTGCCCACAGCCAGGTAAAGAGGATTCCATGTCTGGTTCTCCTGACTCTAAATTTGTGATATTTCCACC

ACACCACACCATGGAAATTAG|GT|TTTAATGTCTTAGGGCAAGGACAAGCCAGTATAGGCTGTATTCATTCCTTGCTGAGGGCCCTGG
 >chr3:38483604-38483853 + E=2e-138
TTCCTCTGGCTAAGACTAGGCCTAGCTATTTGACTTGTTGTAGGCAGAGTTGGGCCTTCGATTTAAATGCTTTGTACCCTTGATTAGCT

AGAAAGTTTAAGAGACCTAGAGTAGCCTGCTGGCATGAGGCTTCTGAACTGGTAGCCAAAAGTAAATCATCCACATGCTAAAGGACCAG

AGTGC
