# Supplementary material for: Chromothripsis during telomere crisis is independent of NHEJ, and consistent with a replicative origin
Source: Genome Res. 2019 May;29(5):737–49. doi: 10.1101/gr.240705.118 (PMC6499312; doi:10.1101/gr.240705.118)
Supplement: Supplemental Material [file supp_gr.240705.118_Supplemental_file_1.zip › contigs/annotated_contigs/DB111/contig.2.DB111_length_450_mean_cov_5.33333333333.docx]

**DB111_length_450_mean_cov_5.33333333333**

AAAGAAAGTCAGGATCAAAATGCAGAGATAAAATTTGAGCTCTTGAGGGCTCAGGAAAAAAGCTATTGCTCTAACATCAGAGTGGCTCT
 >chr18:52424501-52424841 + E=3e-184
AACCTCTGCTGGATGTCTGTTGGACATCAATTAGAAGACTATTTCCTTCTAAACTTTGCAATTATCATACCTTTTGACTATAAACTATT

TCATCTTAATTTGAGAAACAACAATTGCATAATAGTAAAAAAGATGGTGTGAAATTAAATCAACTCTTTCATGAGAAAGCTGTTGATAT

GTAATTTTATTTACAACCTCTTGTATATATAATGAATTGTTATATGTGTTCACTAAGAACTGAATATGA|ACAA|TTAAGATTGTTGTC
 >chr18:52425416-5
AATTCCCTGGTCATTTGATGCAGACACTGCCTTCACTGTAATTTAAAAAAATATTCAGTAACTTCTCATTCATTCCATTCAATGTACTT
2425530 + E=5e-54
NATACTT
