# Supplementary material for: Chromothripsis during telomere crisis is independent of NHEJ, and consistent with a replicative origin
Source: Genome Res. 2019 May;29(5):737–49. doi: 10.1101/gr.240705.118 (PMC6499312; doi:10.1101/gr.240705.118)
Supplement: Supplemental Material [file supp_gr.240705.118_Supplemental_file_1.zip › contigs/annotated_contigs/DB111/contig.2.DB111_length_460_mean_cov_5.86956521739.docx]

**DB111_length_460_mean_cov_5.86956521739**

AAAAGGAATGACAAACGCAAAGACCTTGAGCTGGTAATGAGCTTGGAATAAGGAGTGGAGAGGCCATCTGCCTTGAAAAGTTGGGAGGC
 >chr8:61782040-61782231 + E=2e-103 p=1e-02
ATGGTCAGATTACATAGGGCCTGGATGGCTCTGGTGGAGGATTTGGGTTTCATGCTGAGTGCTGTAGAAAGTCTTCTGAAGGTTTTATG

CAGAAAAATGGT|C|TCCTAAGCAGGTTGACATTTTCTTCACTTGAACAAAGATGGCAGAATCCCATTTCACATGTTGGCAGGCATGCT
 >chr8:61783581-61783851 + E=5e-151
ATTTAAGTGTGCTGGTGCCTCTCCACAGTAGGATCCTGCTGTGAGCCTTCCCTTCTCATGAGGTCCTTCCTGGGCTCCCAGATAAATGT

CATGATAAATTTGGAGTTGTAGCTAAAGGGCAGCCTAATAGATTTCTAATATATAATAAATAGTAGCACTAGGTCAAAATACTGCTTAG

GAATCACTTTATACTCC
