# Supplementary material for: Chromothripsis during telomere crisis is independent of NHEJ, and consistent with a replicative origin
Source: Genome Res. 2019 May;29(5):737–49. doi: 10.1101/gr.240705.118 (PMC6499312; doi:10.1101/gr.240705.118)
Supplement: Supplemental Material [file supp_gr.240705.118_Supplemental_file_1.zip › contigs/annotated_contigs/DB111/contig.2.DB111_length_479_mean_cov_5.20459290188.docx]

**DB111_length_479_mean_cov_5.20459290188**

CATTATGCTTGTAATTTATGGCAAACAAAATAAATAAGCTCAATGGCTAAAACCTTGCAAAACACCCAGACTTAGACAAAAGGAGCCTT
 >chr8:96346603-96346858 + E=6e-142 p=0e+00
TACATCTGCCTGTTAACTCAGGACTAAATCCCTGGAAGGACTCTCAAAGCTGCTGCCCAGGTTTTCAAGAAGAAACCTCAATCTCGTCA

ATGAACTGAAGCCAAAGGTCAGAGTTCCCTCCAGTGTATACGCAGTCAGTGGGCCCCATTTGTCATTTTGCTCTGGG|AAAATTTGTCA

TTTTGCTCTGATTCC|TGTGGAATCAGGTTTCCTTTTAGGATATTTTAGTTCATTCGTGCTGCTATAGCAAAATACCGCAGCCTGGGTA
 >chr8:96354867-96355065 + E=1e-107
ACTTATAGATAATGGAAATTTACCTTCACGTAGTTCTGGAGGCTGGGAAGTCTAAGATCAAGGTGCTGGCAGGTTTGGTGTCTGGTAAG

GGCTGCTCTCTCTTTCCAAGGAAGGGCTGCTCTCTG
