# Supplementary material for: Chromothripsis during telomere crisis is independent of NHEJ, and consistent with a replicative origin
Source: Genome Res. 2019 May;29(5):737–49. doi: 10.1101/gr.240705.118 (PMC6499312; doi:10.1101/gr.240705.118)
Supplement: Supplemental Material [file supp_gr.240705.118_Supplemental_file_1.zip › contigs/annotated_contigs/DB111/contig.2.DB111_length_497_mean_cov_10.5633802817.docx]

**DB111_length_497_mean_cov_10.5633802817**

ATATATATGCCATATATATATATACTCAATAAATATTTATGGAGTACCTACTATGTGCCCAGCCTTGTCTAGGCTCTGGGAATTAACAA
 >chr3:142914072-142914313 - E=2e-133
GATACATATCTCTTTTGCATATATATGTGTATGCATGTGTGCTTGTATGTGTATTAATATATATGTGTGCATATATATGCAAAAGAACT

GGCATCAGACTTTTCACCAATCATACTGGATGTTAGAAGATGCAGCAATGCTGGTTAA|GTTCT|CAGTAACTTCTCTATAAAGGTTGG
 >chr3:142912867-142913128 -
TTTTTATTATTGTATTTTTTGTATCAACAAGAAAAGAGAGACAATTCAAGATTTTGAAAAACAAAACAAAGCTGTCAAGAAATGTTGTA
E=2e-139
TGTCAAGCCAAAGCAAATTAAAATGCAGTATGATTTTGAGCAAATGAAGACGGAATATGAAGCTGAAAACAGAAAATCCATTTGACCTT

GAAGATTGAAGTTTTTGCCTTCGAGTGCCACAGGTTTGGTGATTTAGGATTATT
