# Supplementary material for: Chromothripsis during telomere crisis is independent of NHEJ, and consistent with a replicative origin
Source: Genome Res. 2019 May;29(5):737–49. doi: 10.1101/gr.240705.118 (PMC6499312; doi:10.1101/gr.240705.118)
Supplement: Supplemental Material [file supp_gr.240705.118_Supplemental_file_1.zip › contigs/annotated_contigs/DB111/contig.2.DB111_length_553_mean_cov_8.95117540687.docx]

**DB111_length_553_mean_cov_8.95117540687**

ATCACCAGTTGCCTAAAGACAAAATAGACAAATGAGATGACATCAAATGAAAAGCCTACTGCATAGCCAAGGAAATAATCAACAGAATT
 >chr12:44638231-44638528 - E=4e-167
AAAAAACAATCTGTGTAATGGAAGAATATATTTGAAATCAATATATTTGATAAGGGGTTAATGTCTAAAGTATATAAAGAACTCAAACT

ATTCAATATTAAGAAAAAAAAATCTGATTAAAAATGGGCAAAGGACCTGAACAGACATTTATCCAAAAAAGACAAACACATTAGTTTGC

CTAATGTTGAATTGTTTTGTGTTGCT|AGAA|GATGTCAGTCAAAAGGTTCAAAGTTTCAGTTAGGAGGAATAAGTTTTAGCGATCAGA
 >chr12:44637291-44637551 - E=8e-145
ATGGCACAGCACAGTGACTATAGTTAATAATAACGTATTTTATATTTCAGAATTTGCTGAAGGAGTTTTAAGTGTATCATCACAAAGAA

ATAAGTGTGTGAAATAATAGATATGTTAATTTGCCTGATTTAATGATTCTATGATATACGTGTGTGTATCATAGCACCACATTGTACCT

CATAAATATATATAATTATTA
