# Supplementary material for: Chromothripsis during telomere crisis is independent of NHEJ, and consistent with a replicative origin
Source: Genome Res. 2019 May;29(5):737–49. doi: 10.1101/gr.240705.118 (PMC6499312; doi:10.1101/gr.240705.118)
Supplement: Supplemental Material [file supp_gr.240705.118_Supplemental_file_1.zip › contigs/annotated_contigs/DB111/contig.2.DB111_length_575_mean_cov_8.5147826087.docx]

**DB111_length_575_mean_cov_8.5147826087**

AGCTCTCTGAGCTGTTGTAACACTAAATAAAACTCTTCTTGTGGCCGGTTGCGAGGGCTCACGCCTGTAATCCCAGGACTTTGGGAAGC
 >chr5:103559058-103559316 - E=1e-143 p=0e+00
TGAGGTGGGTGGATCACCTGAGGTCAGGAGTTCAAGACCAGCCTGACCAACATGATGAAACCCGGTCTCTACTAAAAACACAAAAATTA

TCTGGGCGTGGTGGCACAGGCCACATGTATTATATACATGTGTGTGTGTGTGTTTGTG|TATATATATATATATATATATA|CGTGTAT
 >chr5:103589504-103589843 -
ACATATGTATATACACACATACACACACACACAAACTCTGGGTTCATGGATTTGAAAGGTATCTTATGTTCATGGATTAGAAGAATTAC
E=2e-186
TATTGTTAAAATGTCTATACTACCCAGAGTGAGCTATAGCTTTAGTACAATATTTAATAGAAATATGCCCCTAGATTGTCTCTTCAATA

CATGGTGTTGGGAAAATTGGATATCCACATGCAAAATAATAAAATGAGCCTATGTTTTTTGTAAGCAACATGGTAAACCACAACACAAA

AATCTATAATAGCTTGCCTACAAGATAAAGAAAATTACCTCAA
