# Supplementary material for: Chromothripsis during telomere crisis is independent of NHEJ, and consistent with a replicative origin
Source: Genome Res. 2019 May;29(5):737–49. doi: 10.1101/gr.240705.118 (PMC6499312; doi:10.1101/gr.240705.118)
Supplement: Supplemental Material [file supp_gr.240705.118_Supplemental_file_1.zip › contigs/annotated_contigs/DB111/contig.2.DB111_length_580_mean_cov_9.54482758621.docx]

**DB111_length_580_mean_cov_9.54482758621**

AAATAAGACATGAAGGCAAATTAGAGAAAAACATAATAAAAAGGAATGAACAAAACATCCAAGATCTATGGGATTTTGTAAGAACACCG
 >chr7:85497176-85497450 + E=3e-150 p=2e-02
AACCTATGATTGATTGGGGTACCTGAAAGAGACAGGGAGAACAGAACCAAGTTGGAAAACATACTTCAGGACATCCAAGAGAATTTCTC

CAACCTAGCAAGACAGGCTAACATTCAAATTTAGGGAATCCAGAGAACACCAGTAAAATACTCCATGAGAAGATAACCCCCACAAACAT

AATCATC|TTAGTTCT|TAGAGAACTAAGAGCAAACAAACCTCAAGGGTAACAGAAGACAAGAAAGAACCAAGACGTGAGTGGAACTGA
 >chr7:85498834-85499133 + E=2e-160
AGGAGATGGAGACACTAAAACACTTCAAAAAATCAATGAATCCGTTTTTATGAAAAAATTAATAAAATAGACCATTAGCTAGACTATTA

AAGAAGAAAAGAGAGAAGAATCAAATGGATACAATAAAAAATAATAAAGACCATATCACCACTAACCCCACAGAATTAGAAACAACCAT

CAGAGAATAATATAAACACCTCTATGCAAATAGAGTAGAAAATCTAGA
