# Supplementary material for: Chromothripsis during telomere crisis is independent of NHEJ, and consistent with a replicative origin
Source: Genome Res. 2019 May;29(5):737–49. doi: 10.1101/gr.240705.118 (PMC6499312; doi:10.1101/gr.240705.118)
Supplement: Supplemental Material [file supp_gr.240705.118_Supplemental_file_1.zip › contigs/annotated_contigs/DB111/contig.2.DB111_length_587_mean_cov_15.797274276.docx]

**DB111_length_587_mean_cov_15.797274276**

TTGTCCGCCTCTCAGGAGTGTATTGACAGCTGGAGGTGATGGTGTGTGGCAAATGCCTGGTAAATTCTGCAGTGCTTTGCAAAGGCAGA
 >chr20:24376370-24376650 + E=8e-154
GTGACTTCCCTAACTCTGAGACTGTTCCTACTGCTTCTTCTGGTTAATGTCCACATTCAAAAAGTAAATGCCTCACTGAAATCAGGAAG

AATTTGTTCATCCAATTTGGGCCAAAGCGAGTGGTTCTTTTCTATGAATGCAGTCAGGAGCCTAGTGATGACTTTATTTCTGGTATCTG

AGGAGGTGCTG|CA|ATGGTTGAGCTAGTTTACACTCCCACAAACAGTGTAAAAGCGTTCCTGTTTCTCCACATCCTCTCCAACATCTG
 >chr20:24377709-24378018 + E=3e-174
TTGTTTCCTGACTTTTTAAAGATTGCCATTCTAACTGGTGTGAGATGTTATGGCATTGTGGTTTTGATTTGCATTTCTCTGACGACCAG

TGATGATGAACATTTTTTCATGTGTCTGTTGGTTGCATAAATGTCTTCTTTTGAGAAGTGTCTGTTCATAGCCTTTGCCCACGTTTTGA

TGGGGTTGTTTGATTTTTTCTTATAAATTTAAGTTATTTGTAGATTCTGGATATT
