# Supplementary material for: Chromothripsis during telomere crisis is independent of NHEJ, and consistent with a replicative origin
Source: Genome Res. 2019 May;29(5):737–49. doi: 10.1101/gr.240705.118 (PMC6499312; doi:10.1101/gr.240705.118)
Supplement: Supplemental Material [file supp_gr.240705.118_Supplemental_file_1.zip › contigs/annotated_contigs/DB111/contig.2.DB111_length_597_mean_cov_13.0502512563.docx]

**DB111_length_597_mean_cov_13.0502512563**

TGAAGTTGGTTCCTGAGCTTTCGAACTAAGAAGCCAGATTGCTGGAGTATGAAAATAGTAACATGCTATGCAGTGATGAAATCACCCAG
 >chr3:127238482-127238737 + E=9e-142
TCTTCTAAGGTGAAACAGTACCATTTACATTTAACCTCAAGGAACAACCTGGAATTTTTTTCTTGGAATTTACATTTTCTTGTTTTTGG

TGGGGAGCAGGTGGCGGGGACAGAATTTCACTCTTGTTGCCCAGGCTGGAGTGCAATGGCGTGATCTCAGCTCACTG|A|TACTGTCTG
 >chr3:12
TTTTCTATGTTTAGCCGTGTTTAGGTAAACAAACGCTGCTATGTTACAATTTCCCACAGTACTCAGTACAGTCACATGCTGTACAGGTT
7240479-127240820 + E=2e-190
TGTAGCCTGGGAGCAACAGGCTATCCCATGTAGCCTAGCTGTGTAGTAGTCTATGCCATCTAAGTTGTATAAGGACACTCTTTGCACAA

TGATGAAATTGCCTAAGGATGCATTTCTCAAAACATATCCCCATTGTTAAGTGACACATGACTGCAGTTGGGTACTAGATAAGGTACTA

AAGTTATCTCATATTTTAGTTATTTTAATACAGGTATTGAAATAGCTAAAAAATGAAAAATGTCA
