# Supplementary material for: Chromothripsis during telomere crisis is independent of NHEJ, and consistent with a replicative origin
Source: Genome Res. 2019 May;29(5):737–49. doi: 10.1101/gr.240705.118 (PMC6499312; doi:10.1101/gr.240705.118)
Supplement: Supplemental Material [file supp_gr.240705.118_Supplemental_file_1.zip › contigs/annotated_contigs/DB111/contig.2.DB111_length_623_mean_cov_11.7495987159.docx]

**DB111_length_623_mean_cov_11.7495987159**

GCCCCAGAATTCTAGGTTTGGTTACCAGCACCATGGATGCAGTGGTCACAGGACTTCTATTCATCTGTATTGCCCCAGTAGGAAAACTA
 >chr7:148237076-148237415 - E=3e-189
AGCTTGGCCCTGGCTGCACCCTGCAACCTCAGCACTCAGGAATCTGCTGCTCTGAGCTGAGTCACCAGACTTGGTACTCGAGACGTGAA

TCCTTTTGGGACAGGATTCCTAAATAGGGATCTTCTTTTAGGAGTTCCAGAACAGTTATTTGTAACACATAGAATGGACAAAAGATTAG

AATCCAGAATACGTAAAAAAGATTCTTACCAATCTTTAAGAAAAAATTTTTTTTAAATAAATGAAAAAA|TCA|TAGAAGAATGCATCC
 >chr7:148236074-1
AGTGTGGTTCAATTTACATAAAGGCGAACAACAGACAAAAGTCAATAATGTATTGCTTAGAGATACGGGGTAGAATTGAAAGAAAAGTA
48236361 - E=6e-161
AAAGATTAAATAACATGAAAATCAAGATAGTGGCTACCTATGGTGGAAGATGGAGAGTGAGGGGGTGTAATGGACATGCGTATGAAGAG

AGTGTCCCCGTCATTGATGGATGGTGGTCCATTACCTAAGCTGGGTAGTGGCCACGTAGGTGTTTGCTTTATTATTATTTAAATTATAT

AT
