# Supplementary material for: Chromothripsis during telomere crisis is independent of NHEJ, and consistent with a replicative origin
Source: Genome Res. 2019 May;29(5):737–49. doi: 10.1101/gr.240705.118 (PMC6499312; doi:10.1101/gr.240705.118)
Supplement: Supplemental Material [file supp_gr.240705.118_Supplemental_file_1.zip › contigs/annotated_contigs/DB111/contig.2.DB111_length_631_mean_cov_8.64025356577.docx]

**DB111_length_631_mean_cov_8.64025356577**

GTCATATATTCATGTCATTGAACCGATTTTCACATTGGATGTCTAGAATCACAATAGCCTTCCTTCTGTGTGTCAGCTGGGAACACACC
 >chr13:67790815-67791080 - E=1e-147 p=0e+00
TGGACTGGCTACTTTTCTTTGCTCCATTTCTTGTCATTCACTAGGTGGACTATATTCATGTATGTTGTTAATACATCTGATTTCTTAGT

TTCTTGATGGATTTTAAAGTGATGTAATATTATTTCTGCATAATCTTTTCCGTATATATATCC|ATATATATATATATATATATATAT|
 >chr13:67788707-67789097
ATATATCTGGCCCGCAATAGCCAGACACTCAGAAAATGTTACCTGTTTTTCTACCTCCTGTCCAAGAATACCAGCTTGCCATTGACAGA
 - E=6e-214
TGTTTGCACATTCATTTAGTGAATGCTTTCTTGATTTTGTTTTTGGTTGTTTTCTTGAATCTGATTTCTATTGTATCTTTTTCACGACT

CACCTGTGTTCATCTGCTTTTGTGTTAAAACACTATCTGGTTTCAGATTATTTAGGACTTTTGGAAAGTTGATCTTCATAACAGTATCT

CAATATAGCATATTTGATGTTGTTTTTGATCTTAATCCCTGTTGTTTCACATTTGTATAATTGCTTCGTCTTTCATTTTGAGTATTTTA

CCTATTTCAT
