# Supplementary material for: Chromothripsis during telomere crisis is independent of NHEJ, and consistent with a replicative origin
Source: Genome Res. 2019 May;29(5):737–49. doi: 10.1101/gr.240705.118 (PMC6499312; doi:10.1101/gr.240705.118)
Supplement: Supplemental Material [file supp_gr.240705.118_Supplemental_file_1.zip › contigs/annotated_contigs/DB111/contig.2.DB111_length_632_mean_cov_5.45886075949.docx]

**DB111_length_632_mean_cov_5.45886075949**

TAATATGTATCAATTTTTGAAGAAGAGGATTAAATTACTGTGCATACAGTTATTTTCACTGCAAGAAAGATTTTGCGAAGGAGTTTCGT
 >chr7:146213722-146214034 - E=5e-173
TTTGGGGCACTGTTTGAATTTCATTTATAGCCAATAAAATACTGTCAGAAAAGCAAAGAAGCGTGGCAAGGGAAAGGGGGTTCAGGCAA

CAGAGGGGAGATTCATAAACCTTGTATTTCATCTACATGTTCATAATCTACCCTAATGCATTCAGGAGTGTTTTGGGATTTGGCCAGAT

GACTTTTTCTCTTCAGGGGTTAGTTGCAGAGTCAAGA|GACATATT|GAGATAGGAATCTATAATGATTAATGATATGTCAATAAACAG
 >chr7:146212687-146213015 - E=1e-185
AATTGTCTAAAAGTGATCATAGGATTTCACTCTGGATTTCTTGGACAATGAGCCAGCTACGATTGCCCCCACCTCACACCTCATGATTT

GGAGGGAATCAGCTCATACTTAGTTTTCCATAGGACCAGTGGTTTTCTGACAATAAGAACTACAAATTCCCATTGGGGTCAAGGTATTT

AGTTATTGGGCATCCAATAAATCTATATTGCCTAGCAGTTAACATTGTCTTTGTTCCTCAATATTCTAGCGTGCTCTTAAGATTCCTTA

TTAATAGGAAG
