# Supplementary material for: Chromothripsis during telomere crisis is independent of NHEJ, and consistent with a replicative origin
Source: Genome Res. 2019 May;29(5):737–49. doi: 10.1101/gr.240705.118 (PMC6499312; doi:10.1101/gr.240705.118)
Supplement: Supplemental Material [file supp_gr.240705.118_Supplemental_file_1.zip › contigs/annotated_contigs/DB111/contig.2.DB111_length_636_mean_cov_7.07547169811.docx]

**DB111_length_636_mean_cov_7.07547169811**

CATAAAAGCAATAAAGTTAAATATATACATATTTTGCTGAGAAGTCAAAAGACTTTTTTAAAATTAAACTAACAATATTAAACTAGTCA
 >chr7:63778927-63779278 + E=2e-199
TGTTTACCAAAAGATTTACTCAATTCACATATTCTTAAAAATATTGGGCTTATTTTTTTAATTCATGACCAGTTATCTTTAAGTTAATT

CAGTACCATGTAGATAATACACAAAGACATTTATAGACATATATATACATGTAGACACAACATATAACTTACTCATGTTTGTATCTAAA

AGCCCTAGAGAGGGAGTTCATTGTAAAAGGGAGTAGAGCTTCAGACCTGAAAAGAAACTGTTTACCCACAACTCTGGGGGCTT|C|TGA
 >ch
CCTCGTGATCTGCCTGCCTCGGCCTCCCAAGTGCTGGGATTGCAGGTGTGAGCCACTACGCTCAGACAAAAACATTTCTTTACTTGTCT
r7:63780100-63780386 + E=2e-160
TTTTCTTTCTAAAATTTATCTTTTTCTTTCTAAAATTTACATCAAGAAGGAATTTTGGAGATGGGGCATTTTTGTTTACTGGAGGCCTA

GGGTAATCACTATTCAAAGCTGTTTCTCTTTGAAATTTTTCAGTAAATAGTTTTCTTTTTTCCAAACCACAGTATATGGTTTTATTTAG

TTCCAGAGAGAAGAC
