# Supplementary material for: Chromothripsis during telomere crisis is independent of NHEJ, and consistent with a replicative origin
Source: Genome Res. 2019 May;29(5):737–49. doi: 10.1101/gr.240705.118 (PMC6499312; doi:10.1101/gr.240705.118)
Supplement: Supplemental Material [file supp_gr.240705.118_Supplemental_file_1.zip › contigs/annotated_contigs/DB111/contig.2.DB111_length_650_mean_cov_11.3076923077.docx]

**DB111_length_650_mean_cov_11.3076923077**

ATAAACACCTTTTTCAGATTCTTTCACAATGATTTTATACTTTTCTCTTTGACTTTATAATGTAATGAACTCTATTAGTGCATTTATAA
 >chr13:105045654-105045988 + E=3e-189
TTATGGATTTTTTTAATTTCATGGATAAGACCTAATTGTTAATTACATATTCTAATACAGAGTTAAATTGTATTTATTACTATTATATT

TAGGAGATTTATAACTATGTTCATGAAGAAAATATATATTTTACAATTAAAAAAAATATGTATATATTTTAACATTTTTTGATCTTTCC

AAAATGACTTTATTGCTATCTTTTGAAGGTACAAAAGTCAATTCTTAACCTTCACCTTGTTCACCA|A|GACTTCAATAAAATGTAACC
 >chr13:105047080-105
TTCATGAAGGTAGCGATTTAATAATTCCTGAATAAATTAATAAGTATTCTATTAAAAAGTGTTTCAAATGACCCCCTCTCTAAAGGAAT
047397 + E=5e-173
ATTTATTATAAACAATATTAAATAAATTTGTTTTGAGAAATTTTTATAAGAAGCAAATCTGTTTAATTTTACTTAACCATGTTATTTGC

AAATGTATTGAATGCCAGAAGTGTTAAACTCCAAGAGATATGTTGGACAATTGCATTCTAGTCCAACGACTGTAGCTGCAACCTACAGA

TATTTACTCCTCACGACTGCAGGTAGTGA
