# Supplementary material for: Chromothripsis during telomere crisis is independent of NHEJ, and consistent with a replicative origin
Source: Genome Res. 2019 May;29(5):737–49. doi: 10.1101/gr.240705.118 (PMC6499312; doi:10.1101/gr.240705.118)
Supplement: Supplemental Material [file supp_gr.240705.118_Supplemental_file_1.zip › contigs/annotated_contigs/DB111/contig.2.DB111_length_658_mean_cov_6.88449848024.docx]

**DB111_length_658_mean_cov_6.88449848024**

AGACTACTTTGTCTGATAATGATACTCATACAGATTTCTTATGCTTTCCATTTGCTTAGTTTATTTTTCCATTCTTTTATTTTCAAACA
 >chr9:34470108-34470480 - E=4e-212
TTCTATGTTTTTACATTTAAAATATATTTCTTGTAACAGGCAACATATAGTTGTTTTTTTCTTTTTCCTAGCCTGATAAACTCTGCCTC

TTAATTGGAGGTCTTTACCATGTTGCTCTTTGTTTTCTAGTTTTTCTCATCTGCTTTTGTTTCTCTGTTCATCTTCTGCCTTCTTTTGT

ATTAACTGAAATTTTTTAGTACTCCATTTTGCTTTCTCTATTGGCTTTTCATATGTGCCTTTTTGTATTTTGTGCATGTGTGGTTGCTC

TAAGGGTTACAA|TATG|TTGCCCAGGTTGGTCTCAAACTTCTGGGCTCAAGTGATCCTCCCGTCTCATCCTCCCAAAGTGCTGGGATT
 >chr9:34468352-34468642 - E=1e-162
ATAGGTATGAGCCACCATGCCCAGCCAGGCTCTCTTTTTACAAGAAAAACAAAACAAAACAAAACAAAACAAAACCTTCTTGGCCGGGC

GTGGTGGCTCACGCCTGTAATCCCAGCACTTTGGGAGGCCAAGACTGGTGGATCAGGAGGTTAGGAGATCGAGACCATCCTGGCTAACA

TGGTGAAACCCCGTCTCTACTAAAAATACAAAAAAAA
