# Supplementary material for: Chromothripsis during telomere crisis is independent of NHEJ, and consistent with a replicative origin
Source: Genome Res. 2019 May;29(5):737–49. doi: 10.1101/gr.240705.118 (PMC6499312; doi:10.1101/gr.240705.118)
Supplement: Supplemental Material [file supp_gr.240705.118_Supplemental_file_1.zip › contigs/annotated_contigs/DB111/contig.2.DB111_length_672_mean_cov_8.92857142857.docx]

**DB111_length_672_mean_cov_8.92857142857**

TCAGATGGATACGAGTCTGTGGGTATGGAAGACAAGCCACAGAGGGTTGACAGCTGTCACTGGCTGGTCTTAACGATATGGAAAAGTTG
 >chr8:49291688-49292045 + E=5e-203
ACATGGTGGGTTCCTACTCCTGAATTTTAGGAAAACTTAATTTAAATCAAAACTAACTAATCAAAACATCTAAGTAGAAGACAAATTTC

TTCCAAAGCTCATGCTTCCAATCTTGTGGCTTTCTTAGTTATGGTATTCCACCTGCAGGTGAGAATGTGACAGGCTGAGGGGGACTCCC

ACCCCCACGACCACGGTCCTGGGGAAGACACTGACATCCACAACCCAGAGGCGCTTGGCTGGCCGAGCCCCAGAGGCTGGAGAGGGAG|

GC|TAAATAGAAGTGTTACCATTTCCCTTGAGATGTGAGTTGATGGTGTAGACTGGTGGAGGGCCGAGGAAGCCTGCAGCACTGGGAGA
>chr8:49290574-49290891 - E=6e-179
GAGGGTCCCGCTTGTGGCAGCATGACTGGGAAGAAACACCATGGCAATGGTTTGCAGGTGGGACTGATGGGGAGATGTGCAAGCTGATG

GAAAGTGTAGAGACGTGTTCATTTATGAATCATCTACAGGCCCAGCTAATCAAATTCATGTCAAGCAATGTTGTTCTCCTTCCCAACAG

GCCTGACTCTGGGAACAGGTAAGTAAATGGGGTGTGCACACTTGAGGTGAG
