# Supplementary material for: Chromothripsis during telomere crisis is independent of NHEJ, and consistent with a replicative origin
Source: Genome Res. 2019 May;29(5):737–49. doi: 10.1101/gr.240705.118 (PMC6499312; doi:10.1101/gr.240705.118)
Supplement: Supplemental Material [file supp_gr.240705.118_Supplemental_file_1.zip › contigs/annotated_contigs/DB111/contig.2.DB111_length_705_mean_cov_9.58865248227.docx]

**DB111_length_705_mean_cov_9.58865248227**

TCTTATCCAAAGCATCCCAAAATAATTTCCGTATACTATTTGAAGCATTTTGCCATTAAGCCAAAACCAATAATGCAAATCTGATTCAT
 >chr9:105979345-105979759 - E=2e-234
TCTTAAACTATTTTTAATTAAGTGGTGATTATCTTTCTTTACCTTTAATATTTTGTTTCTAAAGTGTAATACCACTCAAATGTATCAAA

TTCCTGGGGTTGAAATAATGAAATTTATAAACACCGGAAGTAGGAATGGAATTCAGATCATGGGCAGTATATTTTGGAAAGCCATCATA

GTATAACGTTTTCCATGAGGCACGGAACTTGCACATGCTGGAACACTCTTGGAAAGTCTGAGAAAGTGAGATACAGCATGCATGTACAC

ATCTAACGTAAGCTAAGATAATCATATTTTTCCTTGAAGACTTTAATAAAGCAA|AAGA|GTGAGATAGCTGCCAAGATTGAATATAGA
 >chr9:105978368-105978663 - E=1e
TAGCTTAAAAAAAGAAATGACCAGTTCAAACAATTAATCACTGGAAGCATGAAACAAATGCTTCACAGGCTTTCCATTTCTATCCCAGT
-165
TCATCTCTCATGCTCTAATGTTACAATTTGAAGTTACTGAAAACCTAATGTGAAGTTTTATTCTTCAGATTTTTGAATTAAAGTTTTAT

TTATTTTACAACCTCGCTTAGGACTTGTTTGTAAAATTTAGGGTCCTGATTGCATAAGTGTTGGATTACAAGAAATGGATACGT
