# Supplementary material for: Chromothripsis during telomere crisis is independent of NHEJ, and consistent with a replicative origin
Source: Genome Res. 2019 May;29(5):737–49. doi: 10.1101/gr.240705.118 (PMC6499312; doi:10.1101/gr.240705.118)
Supplement: Supplemental Material [file supp_gr.240705.118_Supplemental_file_1.zip › contigs/annotated_contigs/DB111/contig.2.DB111_length_707_mean_cov_9.33521923621.docx]

**DB111_length_707_mean_cov_9.33521923621**

TAATTCTCTGAGAAGATAAGCAGAAATAGAATGGGAATTATTAACCTTGGGACAAGTGAATATTTGGGGGCTTTGGGTAACTTCAGTTA
 >chr2:117319243-117319545 + E=7e-167
CCAGCCAAAGCCAGCTGTGAGCCAACATAGAGAAAGCTATCATGATTAGTTATTTTTGAAAATTCTAGAGGTTTCTCAAAAATTGAATT

TCTTGAGAAACTCGGATCCTCAAAAAAGCTATTCGGTTCCAAATTGGGGTTTAAGCAACTAAGCAAGAACAGTCACTCCCAAATTCTTT

TTGGTCCACAACAGCATTTAAATTGGTCCCTGAAA|CCAATTTTACATTATATAATAATGTA|AAATATAGTTTACAAACCTCCAAATT
 >chr2:117320380-117320768
AGAAGACATTATAGAATTTTATGCACCCCCACCAAAAAATATTGAAAGTCAAACAAACTAACGTATAATACCAAATAGGATAAACTTGG
 + E=3e-204
TATCTACATTATTTATTTAAGAAATACTTGTTAGGCATAACATACAAGGCAGTACTATAAGTACTAGGGATTCCATGTTAAGTAAGACA

GAGATAAGAGCCTTGATTTTATGAAACTTACTGACATTTTAGCTGTCTTTCTCATTTTCTTTTTTAAAAGTGAAGATATTAGATTCTGT

ATTTTACTACCTAAGAGCTCATTTTTAAATGTTAAAAATTAGTACATTGCTTAGTTTCCTAAAAAGTAATATTTGTTTTTAATCAA
