# Supplementary material for: Chromothripsis during telomere crisis is independent of NHEJ, and consistent with a replicative origin
Source: Genome Res. 2019 May;29(5):737–49. doi: 10.1101/gr.240705.118 (PMC6499312; doi:10.1101/gr.240705.118)
Supplement: Supplemental Material [file supp_gr.240705.118_Supplemental_file_1.zip › contigs/annotated_contigs/DB111/contig.2.DB111_length_724_mean_cov_9.94475138122.docx]

**DB111_length_724_mean_cov_9.94475138122**

ACTTTTAAGGTTTCTACTTGAAAGCAACACATATCATTTCTACTTGTTTTTCACTTGCCAAATCAAGTCACATGATGTAGCCTAACATC
 >chr3:67959153-67959453 - E=1e-168 p=2e-02
AAAGGGTCAGGAATGTATGATCTTCCCATGGAGAGAGGGATGGGAGGGGAGGAGAAGAAAACATTGTGAAGAACAATACTGTTTCTACC

ATATCTAGCAATAACTATTATTTATTGAACAATTACTATATGCTAAGCTTTGTGCTACATGCTATTAGAATTATTATAATTCATCTTTA

CAAAATGTCTGTAAGATATATGTTTTTGTTC|CT|GGGCTTGGGGTCTATAAACCAGACATTCTGACACTTGGTTGCTGCTTTTCTCAG
 >chr3:67992356-67992782 + E=1e-244
CATTTGGTTTTCTTTTAAGGTTGTAAATTGATCCCCCACGTAAGACAGTCTTAGGCAATACATGACTTCTTATTAAATAATGACTAATA

TCATTAAGACAATAAAATATTTCCATAAACAAATAATTAAACCCTTATAAGACCTAGGTCCAAATTGAAGTCCCATTCTCTCAGCCCTT

TCAGGTTTTGGGACTGATTTTTTATTTAAATCAGTGACGACTGCAGGCTTAAGCATGTACTATAGGTCCATTAAACGTTGCTTCAAAGA

GAGAGGGAAAGAAATGGCCTCTAATGATCCCCAACTTTCTGGCAATCATACCCTGTCAGTGAAAGGAATAAAAAAGTACTGAAAAGTGA

ATAGAAAATGTGAA
