# Supplementary material for: Chromothripsis during telomere crisis is independent of NHEJ, and consistent with a replicative origin
Source: Genome Res. 2019 May;29(5):737–49. doi: 10.1101/gr.240705.118 (PMC6499312; doi:10.1101/gr.240705.118)
Supplement: Supplemental Material [file supp_gr.240705.118_Supplemental_file_1.zip › contigs/annotated_contigs/DB111/contig.2.DB111_length_740_mean_cov_7.06216216216.docx]

**DB111_length_740_mean_cov_7.06216216216**

AAAAAAAAAGAAAAAAAAAACAAAGAAAAAAAAGAACACATAAGCAAAACGTGGGAATAATGAGATCTAAATTAATCTATCTAAAAATA
 >chr4:151560515-151560937 + E=8e-214 p=9e-03
ATGCACTTTCTACCCCACCCGTGGTACTCAACAGATTCAGAACTGTTCCCTAGAGTGAATAATAGAAATCTGCAAGGAAAGTTTTGGTT

GTCACAAATTACTGAGTGCTACAATGATTTAGTGGGCAGAAGGAGCAAAAAAAAAAAAAAAAATTCTAGACATTCCACAAGAAGAATAG

TTCAATAATAATAATGGTCCTAGATCCTACATTACTTTAGAATGTTCTACTGTATATCAGTGAAAGTGACAAACTAGAAGATAATCTGA

GCCTATGCCCCAAATATATATATATATATATATGTGTGTATGTATATATATATATACATATATATG|TG|ATTAGTAAAATATGTTACC
 >chr4:151563257-1515
TGTAAAATATGAAATCTCACTTTTGCATCAAGACACACCAATGAGCAATTCAAAAGCACAAGAATACATTCATATGTAAATTCTGCATG
63575 + E=2e-179
TATTTCAAAGTTGTGGTTTATTATTTGTTCTTGTTGTTTGGTATTTCTACATAGTATTTTCTTATGCACAAAAAATAGAAAGTTGCGGG

AGAATGGTATAACATACTAGGCCATCAAAAAAAGAAAGATAAAGATAAGCTTTTAAAATTAGTTGTTACTCTTTCTTTTATGAAAGAAA

AGAATGGTATTTTGGTTTTCCATAAATTAA
