# Supplementary material for: Chromothripsis during telomere crisis is independent of NHEJ, and consistent with a replicative origin
Source: Genome Res. 2019 May;29(5):737–49. doi: 10.1101/gr.240705.118 (PMC6499312; doi:10.1101/gr.240705.118)
Supplement: Supplemental Material [file supp_gr.240705.118_Supplemental_file_1.zip › contigs/annotated_contigs/DB111/contig.2.DB111_length_783_mean_cov_6.89655172414.docx]

**DB111_length_783_mean_cov_6.89655172414**

CAGAGGGAGAGGGCATAGCACAAGTCAATTTTATCCTGCAGGGAGAGCACTTAAAGTAAAATTTTATCGAATAATTACACTGGGAAATT
 >chr3:68022753-68023032 + E=5e-153
CTGCTTGAGTTTCCCCATGTACTGTGTGCCTATGTGTGTGTATTAGAGTCTTTCAAGGATCAGATGAGTTAATAAGCATAAAACACACA

GAACAGTGTTTGCCATCGAATAAACAACAAATACTAGCTTTTATTTTATTATAAAATTTGATTGGAGATACCTCACAGATCAATATATT

TCCAAATGA|TCT|TAGCCTGCCTGATGTGCAACCTTAGACAACTAACCCAATCCCTTCCAGTTTCCATTTTCCCACTCGTGTCATGCA
 >chr3:68023432-68023939 - E=3e-293
AGAAGAAGCAGTATTTCTCTTAATCACTTGTTATGGGGACTAAATGAGATACTGTCTTCAAAGCTTGTATATAGTGCTGGCTGATGGCA

ATTGCTTGATAAAATGCCACTATCACTCTTCCATCTCTTAAATAACTATTTTAGAACAAGACCTGTTACTTAACTTCTTTGGGTGGCAT

CTTTTTCATCTGTTAAGTAAGGAAGTTTGACTAGATAATACTGTATTATTTCCCCTTCAGTGGTACAATTTTGCAATACTAAGAACAAT

AGAAATATAAAGTGCATAGTTGTAAAATTGAAGGGATAGTTAGGTGAAAGGATACAGAAAGAGAGAATTGCATTGAAATATGTTTCTAC

AACTCTCTTTAAACATTTTTTTTATTTGTCCACAGTTTATTTAACAGACCCCTATGGGACTACCTACTCTGTC
