# Supplementary material for: Chromothripsis during telomere crisis is independent of NHEJ, and consistent with a replicative origin
Source: Genome Res. 2019 May;29(5):737–49. doi: 10.1101/gr.240705.118 (PMC6499312; doi:10.1101/gr.240705.118)
Supplement: Supplemental Material [file supp_gr.240705.118_Supplemental_file_1.zip › contigs/annotated_contigs/DB111/contig.3.DB111_length_1063_mean_cov_6.1872060207.docx]

**DB111_length_1063_mean_cov_6.1872060207**

CCCTTCCCCCCCCCCTCCCCCGGCCCCGGCCTGCCCACCCGCTGCGCCTGACTCCCCCCCCTCCACCCCTGCCCGCCCCC|CCTCTTCA
 >chr9:1
CCTGTTTCCCCCCCTCCCCCCACCCCAGCCAGCCCTTCCTCTTTACCTGTTTCCCCTCCCCTCCCCCCACCCCAGACAGCCCTTCCTAT
38337813-138338340 - E=6e-293 p=0e+00
TCACCTGTTTCCCCGCCTCCCCCGTCTGGAATGGCTAAATACACTCTTTGGTGAAGTGCTGGGTCAGGCCTCTTGTCTATTTTTCCTAT

TGTGTTAATGATCATTTTTCTTGCTGCTTCATAAAATTTCTTTAAATAATAAAATGGAAATGACTGAAAATGGCTATTTTTGGTTACAT

ATATTACAAATATTTTATTCCACTTTCTAACTTGCCTTTTCCTTTTCTTCCTGGAATCTTTTGATGAATAGAATCTCTTATCTTCTTCC

CATTTTGAAAGCTGTGGTAAAATAGGCTGGGCATGGTGGCTCGTGCCTGTAATCCCAGCACTTTGAGAGGCCGAGGTGGGCTAATCATT

TGAGGTCAGGAGTTCGAGACCAGCCTGGCCAACATGGTGAAACCCTATCTCTACTAAAAACACAAAAATTAGCC|ATGTGTGCTGGCGG

TCA|CCTGTAATCCCAGCTACTTGGGAGGCTGAGGCAGGGGAGTCACTTAAACGTGGGAGGTGGAGGTTACAGTGAGCCAAGATCATGA
 >chr22:41102622-41102928 - E=2e-153 p=0e+00
CACCGCACTCCAGCCTGCGTGATACAGTGAGATTCCATCTCAAAAAAAAAAAAAGAGGAATACAATATTTTAAGAAGATTTCTCCAGTC

TTGGTGTTTGAGATGGACTGGATTGGTGAAAGAGACTAGAAAACCAGAGACCCTTTGGGGATTTACTGTAGTAATCCAGGTATGTAATG

AGGAGGGCTTAGACTAGGATGCTACCAAGAAAG|TAGCATCCTAC|TTAGCTGGGTGTAGTGGTACGTGCCTGTGGTCCCAGCTACTTG
 >chr22:41100247-41100379 - E=2e-67
GGAGGCTGAAGCAGGACAATTGCTTGAACCAGGGAGGTGGAGGTTGCGGTGAGCCAAGATTGTGGCACTGCACTCCAGCCTGGGCAACA
