# Supplementary material for: Chromothripsis during telomere crisis is independent of NHEJ, and consistent with a replicative origin
Source: Genome Res. 2019 May;29(5):737–49. doi: 10.1101/gr.240705.118 (PMC6499312; doi:10.1101/gr.240705.118)
Supplement: Supplemental Material [file supp_gr.240705.118_Supplemental_file_1.zip › contigs/annotated_contigs/DB111/contig.3.DB111_length_491_mean_cov_5.69246435845.docx]

**DB111_length_491_mean_cov_5.69246435845**

TTTCAACTATGAATTGATTAACATCTTTTTCCCTTGATAAGAAATGAAAGACTACAGTTAGTTGTCTGATATGTACAAATTAACTTGAA
 >chr5:155612561-155612858 + E=3e-164 p=0e+00
GTTAAGGATAATCTGGCATCTGACTTTCCCCAAGGAAACCTTGCCAGAAACAGCGTGATTTATTTTTAACACTTTTCACAAAAACCCTC

CCCTGTGGGACCTCAACAAATGTCACTTCTGTTGTGAGACTTTTAAAAACAGTGGGCTGTGATTTTTACAAACTTATAAATATGGCCAT

ATATATATGTGTGTGTATATATATATATAT|GTGTGTG|TATATATATATATGTATATATATATATATACACACACATATATGTATATA
 >chr17:49926565-49926623 - E=3e-20
TATATATA|T|AGAGAGAGAGAGAGAGAGAGAGATGTCCATGTCTCCCATATGCTTTCTCTACTGTGTTCATAATTGCTCTGTTTCAGA
 >chr5:155612872-155612992 + E=1e-36
ACTCTTCATGACTCTGGGTACTTTTGATCGGCACCATAGATC|AGTGTTCG
