# Supplementary material for: Chromothripsis during telomere crisis is independent of NHEJ, and consistent with a replicative origin
Source: Genome Res. 2019 May;29(5):737–49. doi: 10.1101/gr.240705.118 (PMC6499312; doi:10.1101/gr.240705.118)
Supplement: Supplemental Material [file supp_gr.240705.118_Supplemental_file_1.zip › contigs/annotated_contigs/DB111/contig.3.DB111_length_525_mean_cov_6.82666666667.docx]

**DB111_length_525_mean_cov_6.82666666667**

CATGTACACACACAGCACACATACACATAGCACAAGTGTATACACACAGCACAAGCATACACAGACAGCACAGGTGCACACACACCACA
 >chr8:49291304-49291496 - E=6e-104
TGCGTACACACACACTTGTATACATACACTGTGCATTTCTCTCAGTGAACATTCACCACATGCCTTGGGTGCATGCAGTATTATGCTGC

ATGAGATAGGG|TCC|ATTAGAAAGAGGGTGGAGGCTGGTCTACACACCAGGGCTTTCAGTCTGTAGTTGA|G|TTTTCCGTAGGTCCC
 >chr8:49290298-49290357 + E=7e-24 >chr8:49291030-
ACCACTCATCAGAGTCACAGAGCAAAGCTGCATACCTGGGTGCCAGACGCCAGCCACTCTCCACCGGCACTGCGTTGGGGGTGAGCTTC
49291308 + E=1e-155
TTAACAGGTGGTGCAAACATGAATGCTCTCCACCTGCGGGCCCCAGCTACACCTCAGAGCAATCTCGGAGCCTTTATAAATAAAAATGT

CTCAAGTCCTCACTCGATTTGGTAGATTAGGGAGTCTCTTCAAAATTCCTTCAGTGCTTTGAAGGTGAAGCCAGGTTTGGGGAC
