# Supplementary material for: Chromothripsis during telomere crisis is independent of NHEJ, and consistent with a replicative origin
Source: Genome Res. 2019 May;29(5):737–49. doi: 10.1101/gr.240705.118 (PMC6499312; doi:10.1101/gr.240705.118)
Supplement: Supplemental Material [file supp_gr.240705.118_Supplemental_file_1.zip › contigs/annotated_contigs/DB111/contig.3.DB111_length_527_mean_cov_10.5161290323.docx]

**DB111_length_527_mean_cov_10.5161290323**

CTGTATTGTAGTACAATTATTGAGCACTTAAAGGCTCTGTTCAGATTCCAGCTTGCCCATTACAGTAGGAGGATGTTTCAGGAACTTGA
 >chr18:56518010-56518210 + E=1e-108 p=0e+00
GAAATAACATTATGTCGGACAATGGAATCAGACCAGGCACGGTGGCTCAAGTCTGTAATCCCAGCACTTTGGGAGGCCAAGGGGAGTGG

ATCGCTTGAGGCCAGGAGTTAG|T|TGGCCAGGAGTTAGAGACCAGCCTG|GC|CAACTATTTTAAAGACGAGCCTTTGAGGACAATAT
 >chr7:55576166-55576193 - E=1e-04 p=9e-03 >chr18:56518840-565
CTGATGGATATAATTTGGTTTTCTAGCTTATGGGAACTGAGCTCAATGGACAGTAACAGTGAATGAAGTTGGAGGAAGGAAATGCAGTG
19141 + E=1e-169
ATTATGGGTGGGGGATACTTGGCAAGGCAAGGAATGAATAGGGGTATGTTTAAAAAATTATTTTTACTTCCAAATTTATGGCTTAATTT

TGATTTGTGTTAGGATTTGTTAGCTGTTATTTGCCCGGGGTTTGGCAGTTTGGTATGTACTGGTGGTCTTAATCTGGTATCTGGAC
