# Supplementary material for: Chromothripsis during telomere crisis is independent of NHEJ, and consistent with a replicative origin
Source: Genome Res. 2019 May;29(5):737–49. doi: 10.1101/gr.240705.118 (PMC6499312; doi:10.1101/gr.240705.118)
Supplement: Supplemental Material [file supp_gr.240705.118_Supplemental_file_1.zip › contigs/annotated_contigs/DB111/contig.3.DB111_length_650_mean_cov_8.53846153846.docx]

**DB111_length_650_mean_cov_8.53846153846**

CCTGATATATATTTCAGGAAAATATAACAAAGAATCAATTTTGTAGAATTCAGTTAGCTAGGGGTTAAATTCTACCCTAGCAACAGAAA
 >chr5:160998385-160998669 + E=4e-159
CTTTACAGCTGCTGTCAGAAACAGTAATTACTCTGGTCAATATTCTCTAAGAAACTCCAGCTGTCTTATCAAGCTTGCTTTGTCAGAAA

GACTCCTTTCCTGTTAGGGGCATCAGTTGTAGAATAACCATGATGGTGCCAATGAAAGAGTTGAAAAAAATGTTTTAGAAGCCATAATG

TGGCATATATATATA|TA|AATTTAAAGTTCTGATAATATCT|ACT|AGAGCTAATTATTACCATCCTCATTTTACCTAAAATAAAACT
 >chr5:160998700-160998728 + E=4e-05 >chr5:160999261-160999604 + E=1e
TCAACTTTTTAACCTGAGTTAATTTCCATGATCGATGCTTAAATACTTTTTTAGCATCGTCCTATGCCATGCTTAGACACTGAGACCTT
-191
TTTTAATTCTCAGACTTACCAAGCCTGTTCTTAACTTAGGCACTTTGAGCTAGATGTTTTTGGGGTTTTCTTTCTCACATTATTGTTCC

CTCTGTCCATGTTGCCATCTCCTGATCTTTGTATAGCTGGCTCCTGCTTACAATTACAATTTCATCTTAAATGCCTCATCTCCCAAGGG

GTCTCACTTGATCATCATACATAAGGTTGTC
