# Supplementary material for: Chromothripsis during telomere crisis is independent of NHEJ, and consistent with a replicative origin
Source: Genome Res. 2019 May;29(5):737–49. doi: 10.1101/gr.240705.118 (PMC6499312; doi:10.1101/gr.240705.118)
Supplement: Supplemental Material [file supp_gr.240705.118_Supplemental_file_1.zip › contigs/annotated_contigs/DB111/contig.3.DB111_length_842_mean_cov_11.2232779097.docx]

**DB111_length_842_mean_cov_11.2232779097**

TCCCCACCAACCCACCCACCCACCAACCTACCTACCCTGTTAAATCTGTCTCCCTTGGTACCCTGACTAGTACACACAAGCTGGAAGGT
 >chr5:13311035-13311211 - E=5e-94
TTTAGTTGAGATCCTGCCTTGGTAATCTTAAAGGCATGTTGTCTTCTAGGTTTGTTTATGACATCAACTAAAATGACTACGAT|AGCC|
 >chr
TGGGGCTCTCAGCAGGAGGACCCTTTGGTTATTGCATCACACTTTTGGACCAGCATGCCGGTGGGGTGCAGGCGCCATTTGTCTTGGAA
1:27331946-27332253 - E=9e-173 p=1e-02
GTCAATTTCCTTCAGGAGACAAAGCAAGTTTGGGGTGGTAGCCTTAATGCCCAGCACCTTGGATTGATTCCCATGGTCTAAGAGGGTCT

TTTTGACTAAGGCCAGCCTGTGGTGATCCCAGCAGCTTTGGAGGGCCTGCAGCCCACACACTGACTGATACATGGTATGTTACAGGGAC

TTGTCACTTTGGATCAGGCTGGGCCACTACAGGG|CC|AAATGTCTTTAGTCAAAGCAAGTTCACCTGTCTACTCTGGAGTTGACATGA
 >chr5:13308786-13309151 - E=1e-207
ATAGATATCTATCCCCAAAATGGTAGATAATATCTCTCTCTATATATGTAGAGAGAGAGATAGATATGTGTGTGTGTGTTTATGTATAC

ACACACATATACACACACGTGTATATATATAGACACATATATATGCACACATGTATATATGTATATATAAAAATACATATATGTGGGTA

TTTATAATATACATATATGCATATATAATACATATATAAAATAACATAATATGCATATATATCTCTCCACCTTGTTGCTATTGTTCCCT

ATGGGCTCATATTTTTCACTCTCATTAAAGTAGCCATTTTGCCAG
