# Supplementary material for: Chromothripsis during telomere crisis is independent of NHEJ, and consistent with a replicative origin
Source: Genome Res. 2019 May;29(5):737–49. doi: 10.1101/gr.240705.118 (PMC6499312; doi:10.1101/gr.240705.118)
Supplement: Supplemental Material [file supp_gr.240705.118_Supplemental_file_1.zip › contigs/annotated_contigs/DB111/contig.4.DB111_length_805_mean_cov_10.150310559.docx]

**DB111_length_805_mean_cov_10.150310559**

AAAATATTGTTAATAAGACTGGACTGACAAGATAAATGAAATATGAACACACACATACACTAAAGTTGATGAAATTCATTTATGGGAGA
 >chr8:85254282-85254456 + E=8e-93
ACCAAACCTTTACTTAGGGAACTGTAATTCAGAAAATAAAAAGGACAAAAAGAGCAGAATCTATTAATGTCAGTCAAGTTCAC|AG|GA
 >ch
TATTTACTGTCAATAGGTTTATACTATTAATGAGTTATATTCATAGTGGTATGAAATATTAGTTATGTCAGCTTCTTACAATTCATGGG
r8:85255414-85255703 + E=5e-162 p=0e+00
ACTTTTGCAGACCTTCAGAAATGCAGATAAGCATTGCTCTGCTCACTTTGAATATCGGATACATCCTCCTTGCACTTTGAAAAGCAAAT

AGAATACATACATTTATCAATGCTGACATTGATACCTTTCTACATAGCTCAGCAAAGAATCATCCAAATAATACAAAATTGAAAAACTG

GAAGGCTAAACAAAATAA|ACTAAACAAAAT|AAGGCTAAACAAAATAAGCCGTCTTATATTTTACAGTTGAAATACTTAGATGTAAGT
 >chr8:85255686-85255868 + E=1e-97
GAATTAAGTAATTCTACCCCTATATAAAGTGATATTTTTTCCTCCTCTTAATTCTTTCTTTCAGGAAGAAGCACAGATAAACAGAAGGC

TAACACAATATGATTTATTTATTTATTTATTTATTT|GTTTGCCTTTGACGCCCACTCTTGCTCTCCCACCCAGCCTCCAGTCCCCTCG

CCCCCTCCCCCCCCCCCCCCCCCCCCCCCCCCCGCGCCCCCCCCCCCCCCCCCCCCCCCCCCCCCCCCCCCCCCCCCCCCCCCCCCCCC
 >chr2:33141312-33141410 - E=4e-35
CCCCCCCCC
