# Supplementary material for: Chromothripsis during telomere crisis is independent of NHEJ, and consistent with a replicative origin
Source: Genome Res. 2019 May;29(5):737–49. doi: 10.1101/gr.240705.118 (PMC6499312; doi:10.1101/gr.240705.118)
Supplement: Supplemental Material [file supp_gr.240705.118_Supplemental_file_1.zip › contigs/annotated_contigs/DB111/contig.4.DB111_length_933_mean_cov_9.57341907824.docx]

**DB111_length_933_mean_cov_9.57341907824**

ACATGTACCAAATGGGTTTCTCGCTTTTTCAGCCCTTAACTAAACAAGCACATCCAGGTGAACTCCACCCCCCAGCCCAAAAAGGTAAG
 >chr1:27330345-27330643 + E=3e-167 p=3e-02
TCTGTTGTGTTTACACTGTGAGCCTCTCATGCCAAGGCTGAAATAGCCAACTCAGGAGGTGAGACAAACTTAGTCCTTCCCATGACCAT

AGTTGGGTGGTCACAGCTGGGATGGGGGTGGGAGCTGGTTTTAGTCACCAAGCAAAGAAAGCCTTTCTGTTCACTTTCCAGCCATTTTG

AGCCCAGCAGCACAAGGGACTCAGAGC|AGGT|CCTTCTTTCCCCAGAGGAAGCATGGCACTAAGGTTCAGTGTAGACCGTCTTTATTG
 >chr1:27331473-27331947 + E=4e-270
GCAGGTGTTAAGAGTGCAAAATATCAACAAACCCAGGGGAATACGCAAGGGGGTGGGAGTATGGCTCCCCTACCCCATGTGAGAGCCCT

GTAACCAAGCCAGTGGGGTGGGAACGTTGACTCGACTGTGGCAAATTCAGGCTCAGCACCTTCCAAAGAACAAGCTCCCAGGCAGGAGG

GCTCCTTGCAACACAAGGGGGAAAGGAGTGGCACCCTGGAAGGGGCCTGGGCTGCGACCCACCCTGGGCTGCTTGGCTCCTGTATACTG

CCCACCTCAACCCCTCAAGAGGAAGGCTTCACAGCTGGGGGTATGTAGTTCAGAGAACCGGGCTAAACCCAGCCCTCCCCAAACCCAGG

TATCTGCCTCGGGCCTCAGTTTCCCTCCTCCCAGTGATTACCCAAGTTGGCCCATC|AG|AACTTGAAACTTGGCATTCAAGCTTCAAT
 >chr5:13309467-13309552 - E=3e
TAACCAGTTGGTACTGCCAACTTTCTACCTTTCAGACAAGTTTTCGCCTCTTCA|CATTGCCAACTGGTTAATTGAAGCTTGAATGCCA
-39 p=1e-02 >chr5:13310344-13310426 - E=2e-3
AGTTTCAAGTTCTTGAAGGTTAAGAAAGCCTTTCTTATTGGTTAATAG7
