# Supplementary material for: Chromothripsis during telomere crisis is independent of NHEJ, and consistent with a replicative origin
Source: Genome Res. 2019 May;29(5):737–49. doi: 10.1101/gr.240705.118 (PMC6499312; doi:10.1101/gr.240705.118)
Supplement: Supplemental Material [file supp_gr.240705.118_Supplemental_file_1.zip › contigs/annotated_contigs/DB112/contig.2.DB112_length_286_mean_cov_5.76923076923.docx]

**DB112_length_286_mean_cov_5.76923076923**

GGTTTCCTCCTGAGGTGTGTCCTGTGAGCTCACCTTGCTATGGTAATGGGGAAAACACTGGCATAGGACTAGGAAGAGCTGGGATCTAG
 >chr6:20193032-20193157 - E=6e-64 p=4e-02
GCCTGGAGCCAGTATTTATTAAGCGTGTGACCT|TGG|TAGTCAAAGATGTTTTAGAGGAATAAACATAAAAGGTTATTTTTATCCCAA
 >chr6:20187118-20187282 - E=2e-81
TAAGTTTAACTTTGCAATAAACATTTCCACCTAACTTGGTTAATTTGTTTCATTAAACGTGTTTATAGGCTGTAGGTTTTGCTTAAAGC

TGAAAGGCTCAGATGCCCACA
