# Supplementary material for: Chromothripsis during telomere crisis is independent of NHEJ, and consistent with a replicative origin
Source: Genome Res. 2019 May;29(5):737–49. doi: 10.1101/gr.240705.118 (PMC6499312; doi:10.1101/gr.240705.118)
Supplement: Supplemental Material [file supp_gr.240705.118_Supplemental_file_1.zip › contigs/annotated_contigs/DB112/contig.2.DB112_length_318_mean_cov_7.54716981132.docx]

**DB112_length_318_mean_cov_7.54716981132**

CCCAAAGTGCTGAGATTACAGGCGTGAGCCACC|GTGCCTGGC|CAATGCCCTCATTTCAATGGGCCACAAGGAAATTAATTCTGTCAA
 >chr2:101939793-101939835 + E=7e-14 p=2e-02 >chr12:46174881-46175166 - E=8e-161
CAATCACTGAGTGAGTTTGAAAGCAGACTCCTCCCTAGTCAAACTTTGAGATGACTGAAACCCAGGCCAACCATTGACTGCAGTCTTGA

TTGCAACAGATTGACAGTGACAGACTCTTAGCTAGAGTCTAAGCTAGAACTAAATTGTGCCCAGATTCAGAACTCACAGAAACTTTAAG

ATAATAAATGTTTGTTGTTTTAAGTGCTGGGTTTGAGGGTAATTTGTAATGCA
