# Supplementary material for: Chromothripsis during telomere crisis is independent of NHEJ, and consistent with a replicative origin
Source: Genome Res. 2019 May;29(5):737–49. doi: 10.1101/gr.240705.118 (PMC6499312; doi:10.1101/gr.240705.118)
Supplement: Supplemental Material [file supp_gr.240705.118_Supplemental_file_1.zip › contigs/annotated_contigs/DB112/contig.2.DB112_length_326_mean_cov_5.8527607362.docx]

**DB112_length_326_mean_cov_5.8527607362**

AACTATAGAAGAATATTGAACGTATTTCATTGTTGGTGGTATGAACAAGTGCCAATTTTAAAATGTTACAAGATTTGTTCACATTTTAC
 >chr4:136639606-136639874 + E=3e-147
AGTTAATTGAACGATATATTTGCAAATAATCATAATAGTAATAACTATAACAATAAAATTTAATATTAATTTTGAATTTATGTGTGCCA

AGTACCATGCTAATTTACATATGGTACTAATTTTAAACTTATAAAAAGTTCATTCATATTATTGTAATTCATATTTTATAATAAAGCA|

GC|AAACCTAAATTCTATTTTTTGCTTCTGTGAATTTCACTTCTTCAGATACCTCACATAT>chr4:136645122-136645182 + E=1e-24
