# Supplementary material for: Chromothripsis during telomere crisis is independent of NHEJ, and consistent with a replicative origin
Source: Genome Res. 2019 May;29(5):737–49. doi: 10.1101/gr.240705.118 (PMC6499312; doi:10.1101/gr.240705.118)
Supplement: Supplemental Material [file supp_gr.240705.118_Supplemental_file_1.zip › contigs/annotated_contigs/DB112/contig.2.DB112_length_328_mean_cov_10.0609756098.docx]

**DB112_length_328_mean_cov_10.0609756098**

TGTGCACTCATTTTTGGTTCCATGTGAATTTTAGGATTATTTTTTCTAGTTCTGTAAAGAATGATGTTGGTATTTTGATGGGAATTGCA
 >chr2:214501155-214501313 + E=9e-84
TTGAATTTGTAGATTGCTTTTGGCAGTATGGTCATATTTACAATATTGATTCTACCTGTCCAT|GAGCAT|TTAGACCATTTACATTCA
 >chr2:214503940-2145041
AAGTTAGTATTGAGATATGAGGTACTATTCTATTCACCATGCCATTTGTTGCCTGAATACCTTGGGATTTTTGTATTGTGTTGTTGTTT
16 + E=1e-94
TATAGGTCCTATGAGATTTATGCTTTAAAGAGATTCTATTTTGGTATATTTTGAGGATTTGTT
