# Supplementary material for: Chromothripsis during telomere crisis is independent of NHEJ, and consistent with a replicative origin
Source: Genome Res. 2019 May;29(5):737–49. doi: 10.1101/gr.240705.118 (PMC6499312; doi:10.1101/gr.240705.118)
Supplement: Supplemental Material [file supp_gr.240705.118_Supplemental_file_1.zip › contigs/annotated_contigs/DB112/contig.2.DB112_length_358_mean_cov_2.51396648045.docx]

**DB112_length_358_mean_cov_2.51396648045**

TGTGATCCACCCGCCTTGGCCTCCCAAAGTGCTGGGATTACAGGCGTGAGCCACCGCGCC|TGGCCAG|GCACATCAAATTTTATCAAT
 >chr22:17862066-17862133 + E=7e-26 >chr12:46175155-46175453 +
TGAGTACTCAGTACTTGCCTAGTATAACTAAACCCTTAAAATATAACATGTTGATTATGTTTTGGAATAAAGGTCAGATTATCATTGGT
 E=2e-168
CATTACTTGCTAGCCTTCCGGTAAGTACCAAGTCTTACAAGAGTAATGGATATATTTTCGCCAAGTTGAGGAACCTCCACTTTTCTGTT

ATAATTTTTCTTGGAAGTACTTTACTTGGATTGGTTTGAAGGGCTTCACCATAACTCTGCTTTTCAGGCTTTTAAAAATGGAAGTGATT

GTTG
