# Supplementary material for: Chromothripsis during telomere crisis is independent of NHEJ, and consistent with a replicative origin
Source: Genome Res. 2019 May;29(5):737–49. doi: 10.1101/gr.240705.118 (PMC6499312; doi:10.1101/gr.240705.118)
Supplement: Supplemental Material [file supp_gr.240705.118_Supplemental_file_1.zip › contigs/annotated_contigs/DB112/contig.2.DB112_length_456_mean_cov_8.22368421053.docx]

**DB112_length_456_mean_cov_8.22368421053**

ATAAATACTGGCATTACATAAAGTCTTTTACCAATATAGATTTTTCAGTGAATCACATTTAAAGTTACCTCACATGTAATTTTGAATGA
 >chr7:51917898-51918219 - E=9e-179
TGGAGCAAAACTGCACTGCCAGCCCACTTTACAATGCAGGGGATTAGGCTGGCATTACACTTAAGCACTTCCTAATTCACAGGATGGCA

ATCTCCACTGTGGCTCCCTGGAACACGGTGAGAGGCTTTTGCTTCCCCAGGTTGCTGAAGCTGTAATTAAAGACATAAGAGTACCAATC

TCCCTGTGCTAATTACTTGCTGCTTAAAACCTGCTTGGGATATAATTTGGC|CCT|TAAGTGTTTATTACATGGTTTATTAACAGGTTT
 >chr7:51907085-51907223 - E=2e-71
AGGAGGGAGGAGCCAAGATGGCCGAATAGGAACAGCTCCGGTCTACAGCTCCCAGCATGAGCGACGCAGAAGACGGGTGATTTCTGCAT

TTCCATCTGAGGT
