# Supplementary material for: Chromothripsis during telomere crisis is independent of NHEJ, and consistent with a replicative origin
Source: Genome Res. 2019 May;29(5):737–49. doi: 10.1101/gr.240705.118 (PMC6499312; doi:10.1101/gr.240705.118)
Supplement: Supplemental Material [file supp_gr.240705.118_Supplemental_file_1.zip › contigs/annotated_contigs/DB112/contig.2.DB112_length_469_mean_cov_12.2878464819.docx]

**DB112_length_469_mean_cov_12.2878464819**

AGTGGCGTGATCTCGGCTTGCTGCAAGCTCCGCCTCCTAGGTTCACGCCATTCTCCTGCCTCAGCCTCCCAAGTAGCTGGGACTACAGG
 >chr8:7227439-7227755 - E=3e-164
CACCCGGCACCATGCCCAGCTAATTTTTTGTATTTTTTTTCCAGTGGAGACAGGGTTTCACGGTGTTAGCCAGATCTTTGCCCATTTTA

AAAAAAAAATTCAACTTTTATTTTAGATTCAGGAGATGTATGTGCAGGTTTTTACACTGGCACATTGTGTGATGCTGAGGAGTATGAAT

GATCTTGTAACCCAGATAGAAAACATAATACCCAATAGATAG|TTTTT|ATATCTATTCGCCTTCCATTTGTTTTCAAGTCCAGTTTAC
 >chr8:7225838-7225998 - E=1e-84
CTCCAAAGAATTTTAGAATATAGAGTCTCCAGAAGACTCTAGTTTGTCTGGTTTCTTGTTGTATCCCTTGACTGCGAATGCTTTCTAGT

ACATATAATACACTCAAAAGTACTTA
