# Supplementary material for: Chromothripsis during telomere crisis is independent of NHEJ, and consistent with a replicative origin
Source: Genome Res. 2019 May;29(5):737–49. doi: 10.1101/gr.240705.118 (PMC6499312; doi:10.1101/gr.240705.118)
Supplement: Supplemental Material [file supp_gr.240705.118_Supplemental_file_1.zip › contigs/annotated_contigs/DB112/contig.2.DB112_length_478_mean_cov_8.89958158996.docx]

**DB112_length_478_mean_cov_8.89958158996**

AAGATTTCTTGCCCTAATTCCCAGGAGTATAAACATGATGAGATGTCATGTCCTGATTAAGTTACGTGGCAATAGGGATTCTGCAGATA
 >chr6:7814287-7814578 - E=1e-163
TAATTAAGGTTACTAATCAGTTGACTTTGAGTACAATCAGAAGGGAGATTGCCCATGTGGGCCTAATTGAATCTCAGCAGCTCTTTAAA

AGCACATAGTTTTCTCTAGCTGGTAACAGAAGAGAGAGGCAGAGGGATCCCAGGCATAATGAGCTGTTATTGGCTATGAAGATGGAAAG

GGCCACTTGGTGAGAAATACAGGC|CCAAATGGGCTCAAATGA|TTTTTTTTTGTAGAGACAGGGCTTTGCCATGTTGCTCAGGCTGAT
 >chr6:7813161-7813328 - E=4e-84
CTTGAATACCTGCCTCAGCCTCCAGTGCTGGAATTACAGGCAGAAGCCATCACACCTGACCCAGCATGTTTTTTTTTTTTCCTCACCTT

TGTAGAAATCACTGAAAACACAGATACGTGGCCAG
