# Supplementary material for: Chromothripsis during telomere crisis is independent of NHEJ, and consistent with a replicative origin
Source: Genome Res. 2019 May;29(5):737–49. doi: 10.1101/gr.240705.118 (PMC6499312; doi:10.1101/gr.240705.118)
Supplement: Supplemental Material [file supp_gr.240705.118_Supplemental_file_1.zip › contigs/annotated_contigs/DB112/contig.2.DB112_length_482_mean_cov_4.35684647303.docx]

**DB112_length_482_mean_cov_4.35684647303**

ATCCTTTTCTAAACAATTTGGAGATGCTCATTTTGCTATTTGCCCCTGAAATTCCACTGTAATATTTTTCAGCTGCCACAGGTAGCAGA
 >chr12:24576910-24577100 - E=9e-103 p=3e-02
TCAGTGGTTGGTGATTTTATATTCTTTGAGTTTTATGTGGAACCCAAAGGAATAATTAAATTGTGTGATTATTGCCCACTGTCATTTTT

ATTTAAAAAAAT|AAAATAAAATTCACTCCATTTCATTCC|AGCCACTGTGCCTGGTTGAAAATATTCTAAGTTGTTACCTTTTTTATA
 >chr12:24575414-24575679 - E=6e-145
ATCCAACTAATTTGGTTGGCCTCAAGCAATCAATATATGTAGGTAACTTTCATTAGATCTCTGTGGAAAGGATTTTTGGAAACATGCAG

AATTCCCACACATCATTTGGGTTAGAATAAGTTATGAAACTGTGCAGACAACCCTAAAATTTCATCTACAAGGTTTGTTGTGGGTTCAG

GTGACTCTGCAGATCAGTTATCTTACATATGATGGCTCA
