# Supplementary material for: Chromothripsis during telomere crisis is independent of NHEJ, and consistent with a replicative origin
Source: Genome Res. 2019 May;29(5):737–49. doi: 10.1101/gr.240705.118 (PMC6499312; doi:10.1101/gr.240705.118)
Supplement: Supplemental Material [file supp_gr.240705.118_Supplemental_file_1.zip › contigs/annotated_contigs/DB112/contig.2.DB112_length_501_mean_cov_12.874251497.docx]

**DB112_length_501_mean_cov_12.874251497**

GTAAAAACCCTAAAAACCCATTAAAAGATAAAAGGTAGATATTAAAAATTAGCTGGGCATGGTGATGTGCCCATAGTCCCAGCTACTTG
 >chr4:143554983-143555261 + E=9e-156
GGAGACTGAGGCAGGAGGATCAGCTGAGCCCAGGAGGTTGAGGCTCTGGTGAGCAGTGATCACACCGCTGCACTCCAGCCTGACCAACA

GAGTAAGACCATGTCTCAGATAAAAAGGAGGGAGGTGGGGGGAAAAGGCAGGGGGCTGGAGGGAGATATTAGAAACACTTTAATGCAGA

TAAATTCAGCA|TTAAAGTGA|TTGAGATCACTGCATCATGGGGGCAGTTTCCCCCATGCAGTTCTAGTGGTAGTGAGTGATTTCGCAC
 >chr4:143557176-143557390 + E=3e-114
AAGATCTGATGGTTTTATAAGGGGCTTCTCCTTTCACTCGGCACCCATTCTGCCTCCTGCTGCCCTGTGAAGAGGTGACTTCTGCCATG

ATTGAAAGTTTCCTGAGGTCTCCCCAGCAATGCAGAACTGTGAGTCAATTAAACCTCT
