# Supplementary material for: Chromothripsis during telomere crisis is independent of NHEJ, and consistent with a replicative origin
Source: Genome Res. 2019 May;29(5):737–49. doi: 10.1101/gr.240705.118 (PMC6499312; doi:10.1101/gr.240705.118)
Supplement: Supplemental Material [file supp_gr.240705.118_Supplemental_file_1.zip › contigs/annotated_contigs/DB112/contig.2.DB112_length_520_mean_cov_5.19230769231.docx]

**DB112_length_520_mean_cov_5.19230769231**

CTCTGCCTCATAAAAAAGAAAAAAAAGATGCTTTAAGTGCCTTAAAAACAGAAGATATTAATATTGAAATTCTCTTCACCCTAAATAGA
 >chr17:54581825-54582043 - E=1e-119
GAATAACATATTAAGATCATTAAAATATAAACCAGTAATCAGATATACTTCAAGGTGAGAATGCTGTTGATAATAAAGGTGAATAATAA

TTTCTTGTATGGTATCTTCTGCTGGTGATGATCTAGTCC|A|TATATATATATATATGAAATTTGTGCTTCAGTGAATTTATTTCCACT
 >chr17:54580560-54580863 - E=9e-165
GTGTTTGAGTAGACTTTAAATAATTTATAAATGAAGACTTTTACTGAATCTTTCATCAGGCCTTCAGTAGAACCTCCGGGGTTTGTTCA

CCTTCTGATATCCCAAGAGAAATAATATCTCCAGATTCTTGTTTATTTTTTTGAGCTGTGTATTTTATGCCATAGTACTCTTAACATCA

TCTGGAAAAAGGTTACCAAGATTTACATTTGGAAGATCAGGATAACCTTCAAATGCATCTGAAGGAAACAACACCCC
