# Supplementary material for: Chromothripsis during telomere crisis is independent of NHEJ, and consistent with a replicative origin
Source: Genome Res. 2019 May;29(5):737–49. doi: 10.1101/gr.240705.118 (PMC6499312; doi:10.1101/gr.240705.118)
Supplement: Supplemental Material [file supp_gr.240705.118_Supplemental_file_1.zip › contigs/annotated_contigs/DB112/contig.2.DB112_length_535_mean_cov_8.63925233645.docx]

**DB112_length_535_mean_cov_8.63925233645**

TCTTCCTCATGGTGTGAATCTGGAGGCATTCTCCATTCCCCAAACATTTACTCAGCACCGACTCTGTGTCAAACATAGTAGGAGGTAAG
 >chr9:126398688-126398957 - E=3e-150
AAGAGGAATATTTGGGAGGCCGAGGAGGGCGGATCACCTGAGCTCGGGAGTTCGAGACCAGCCTGGCCAATTTGGCGAAACCCTGTCTT

TGCTAAAAATACAAACATTAATTGGGCGTGGTAGCAGGTGCCTGTAATCCCAGCTACTCAGGAGGCTGAGGCAGGAGAATCACTTGAAC

GT|TAAGTATCTCTTGCAGTCAGTACGTTTTCAGTCTAACTAGTCATGATGGAAATATTCTCACGTGAGATGGAATAGCCCTCTGTTAC
 >chr9:126363276-126363544 - E=1e-149
AGATGAGAAAAACTCAGTAATGATGAGGAGAATCCCAGTAAAAAAATGTGAAACCATCATTGATGCTTTTGTTTTCGTATTTTCCTATC

ATCATTTATGTCATTATATATAGGTTGCATTTAGCAATGTTAAAACTGGCTTCAGAGAAAACTTAAAAGTTTATAAATTGTTACCAAGA

GA
