# Supplementary material for: Chromothripsis during telomere crisis is independent of NHEJ, and consistent with a replicative origin
Source: Genome Res. 2019 May;29(5):737–49. doi: 10.1101/gr.240705.118 (PMC6499312; doi:10.1101/gr.240705.118)
Supplement: Supplemental Material [file supp_gr.240705.118_Supplemental_file_1.zip › contigs/annotated_contigs/DB112/contig.2.DB112_length_546_mean_cov_6.86813186813.docx]

**DB112_length_546_mean_cov_6.86813186813**

ACTTGGAGATGTACTTCTGAAGTTCATGTTTTTCCTTTCCTTTATCTATAAATGTGAAAAAAAAACAGTGAGAATGATTTTGCTTTGAT
 >chr7:42385853-42386094 + E=1e-128 p=5e-03
TATGGAACAGTGTTCATGTGAATGAATTTCATAATATTTTAATTAAATATTTCATAACTTGATTCCTAAATACATGTACACACTGACCA

AATGTGGCCCCAAAATATATTTAGTTTAAAGATTTCCAGCCAATACTTCAAGTTTTCC|ATGG|CAGAGACACGGAGCTGAATACTCAC
 >chr7:42387490-42387800 + E=
CCAGTTCCCACTAAAATCTAATTTTATTTGAATGTAAAAAGCGTGACAGTGACTATGTTGCTGATTCCAAACTCTGCTTCCTCAACCCC
6e-175
CACAAACCAATTGAGAGACTGTGAATTAAACATTGCATAAGAAACAGACATCATTTTTATTCCTCACTTGGAATCATCTCAGCATCACC

TTCACTTAATCACAATTGCAAAACACCCCAGAGTCTACAAGAGAAGTTGTCTAGGGGTCTCATCAGAAAGAGAGGGAGACAGGATATAA

AATTACTTCACATC
