# Supplementary material for: Chromothripsis during telomere crisis is independent of NHEJ, and consistent with a replicative origin
Source: Genome Res. 2019 May;29(5):737–49. doi: 10.1101/gr.240705.118 (PMC6499312; doi:10.1101/gr.240705.118)
Supplement: Supplemental Material [file supp_gr.240705.118_Supplemental_file_1.zip › contigs/annotated_contigs/DB112/contig.2.DB112_length_559_mean_cov_2.95169946333.docx]

**DB112_length_559_mean_cov_2.95169946333**

GTAACTGCTTGGCTAGGAGAGATTGACTTACCCCCAGTGGGGTCCCTCATTAATGGCACACATTGGACTAAGGTACCAGCTAACACTAT
 >chr3:163935216-163935454 - E=1e-131
ATATCACTCTACCATCCTCCCACTGTGTGTAAGTTATAAAAGTTCTAACCCTTATGTGTACCTGCCCAAACACAGTTGTGGCTACATCA

TTGAAAAGGAAATCCCTTAAAATTCTTGTTTGCAGGTAGCTTCAAACTGGTCAACACA|AC|ATATCCGTCTTCTAAAATCCCTCAAAC
 >chr3:163952964-163953287 -
AGCCTCTCTTTACAATACTCCCTGGAATTTTGATTATTCACTCCCATTCCTGGAACAGTCACATCTTGATTATAGTATTTTCCTCTCCA
E=9e-183
AATCTTTCACTGCTTTGAGTTCAGTCATCTGTAAAATGCTAAGCTAATAGTGGCAGCTCTCTGATTAAAACCCTTGGATAGCTATCCAT

TATCTTTGTCTAAATCATTTTAAAATATCTTAACTGTCATGTTTTTTTATTTTAATTTCCTCTGTCAAAACGTAAGATTTCCTTATTGT

TTCATGATTAATATGAACTTAGCTTTC
