# Supplementary material for: Chromothripsis during telomere crisis is independent of NHEJ, and consistent with a replicative origin
Source: Genome Res. 2019 May;29(5):737–49. doi: 10.1101/gr.240705.118 (PMC6499312; doi:10.1101/gr.240705.118)
Supplement: Supplemental Material [file supp_gr.240705.118_Supplemental_file_1.zip › contigs/annotated_contigs/DB112/contig.2.DB112_length_564_mean_cov_18.7358156028.docx]

**DB112_length_564_mean_cov_18.7358156028**

GCAGAGCACCAAGCAGACTCTTGGGGTCCCTAATTCCAGGACCTGACTCTTGGATGATGTTTCTGGACCTGCCCTGGGCCAGAAGGGAG
 >chr2:214509874-214510067 - E=2e-104
CCCACTGCCCTAAAAGCTGAGTTCCAGTCCAGTTCCTCATTCACCACAGCTGCCTTAAGAGCCCTTGGGCCTTAAGGAAACATTGGCTT

GTAGTCTGGCAGTA|C|ACTGGGTGGCTAGACCCAGAAGACAAATAACAATCACGGCAGTCCAGCTCTCAGGAAGCTGCATCCCTAGGA
 >chr2:214504890-214505262 - E=3e-212
AAAGGGGGAGAACACCACATCAAGGGAGCACCCAATGAGAAAAAAATAATCTGAACAGCAGCTCCTGTGCTCCAGATCTTTCCACTGAC

ATAGTCTACCCAAATGAGAAGGAACCAGAAAAACAATTCTGGTAATATTAAAAAACAAGGTTCTATAACACCTCCAGAAGATCACACTA

GTTCACTAGCAATGGATCCAAACCAAGAAGAAATATCTGAATTGTCAGAAAAAGAATTCAGAAGGTCAATTATTAAGCTACTCAAGGAG

GCACCAAAGAAAGGAGAAAACAAACTTAAATT
