# Supplementary material for: Chromothripsis during telomere crisis is independent of NHEJ, and consistent with a replicative origin
Source: Genome Res. 2019 May;29(5):737–49. doi: 10.1101/gr.240705.118 (PMC6499312; doi:10.1101/gr.240705.118)
Supplement: Supplemental Material [file supp_gr.240705.118_Supplemental_file_1.zip › contigs/annotated_contigs/DB112/contig.2.DB112_length_602_mean_cov_16.196013289.docx]

**DB112_length_602_mean_cov_16.196013289**

CCGTTTACCTGAGAGGCCAGGTTCACATTTGGGAAGAAGAGATTTTCTCCCTCCGCAGCAGCAGCGCAGCCTGCACGAGGTTGCTGGGG
 >chr10:1440970-1441303 + E=1e-188
GAAACAGACCGCTCTGTCTTTGGGGTCTTGATGGGTAGAGTGGTTTGCCAGAGAACCACCGAAGAGCCTTCCAGGGACTTCTAAGAGTG

GGCACTTACTTCAGCCCTGGGAAATCTCTCCTCAGAGCCTGGCACAGGTTTTGGTATCAGCGGGGCCACGACAAATGTTCACTGAATGA

GTAAATGAATGACATGGAATGTAGGAAACACGAAATAAAATGAACTGAGAAGATTTGCAGGGAC|TG|ACAATTGCGTAGGTCGAAACC
 >chr10:1488842-1489113
CTCTGAGGCCATCAACCCTCCCGGGGCCTAAGTCAGGCACTTTGGCAGGAGTATCAATAAGTGAGAAATCGTTGAATATGTTCACAAGA
 - E=2e-151
AAGCAAAACTTATCAAAGTCCACTCTGAACCAAGCACTATAATAGGCGAACTTGCATACAAGAACGTTTTAACTGTCACGAGAACACTG

TAAGAGAGATGAAAAATTATTCCTTTAAAAAGTATGAAAACTGGCACACAGAGTTCAAGTGACTGCCCTG
