# Supplementary material for: Chromothripsis during telomere crisis is independent of NHEJ, and consistent with a replicative origin
Source: Genome Res. 2019 May;29(5):737–49. doi: 10.1101/gr.240705.118 (PMC6499312; doi:10.1101/gr.240705.118)
Supplement: Supplemental Material [file supp_gr.240705.118_Supplemental_file_1.zip › contigs/annotated_contigs/DB112/contig.2.DB112_length_604_mean_cov_10.3377483444.docx]

**DB112_length_604_mean_cov_10.3377483444**

TTTTCTGTCTCTTCTGAGCCCTCCAAACTGTTCCAACCTCAGCCTGTTACTCAGTTCCAAAGTTGCGTCCACATTTTTGGGTATCTTTT
 >chr21:48011280-48011655 - E=5e-211
AAGTTGCACCCCACTCTACTGAATACCAATCTACTGTATTAGTCAATTTTTACATTGCTGATAAAGACATACCCAAGACTGGGCAAAGG

AAAGACTGGGCAAAGGAAAGGCTTTACAAAGGGAAGAGGTTTAATGGAGAACTCAGTTTTACATGGCTGGGGAAGCTTCACAATCATGG

TGGAAGGCAAGGAAGAGCAAGTCACATCTTATGTGGATGGCAGCAGGCAAACAGAGAATGAAAGCTTGTGCAGGAAAACTTCCCCCATA

TAATAACCATCAAATCTCA|A|CCTGATAGGAGATGCAGTGGCTGCTGCAGGTTACCATGGAGACAGGGAGGCCAGGGCCCAGGGGAGC
 >chr17:42387849-42388077 - E=2e-125
CCATGGCCTGCTAAAGGGAGCTGTTCTGGGAGCTGGAGGCAAACCATGTGACAGTGATGAGGCAGTAGGGGCCTGGGGGTTACTGAGCC

TTTGTTATGTCCCTGACCCCCCAAAACTCTATCCTTCCCTCCCAGCTCTAAACCACCTTCAGCCCTTCAACT
