# Supplementary material for: Chromothripsis during telomere crisis is independent of NHEJ, and consistent with a replicative origin
Source: Genome Res. 2019 May;29(5):737–49. doi: 10.1101/gr.240705.118 (PMC6499312; doi:10.1101/gr.240705.118)
Supplement: Supplemental Material [file supp_gr.240705.118_Supplemental_file_1.zip › contigs/annotated_contigs/DB112/contig.2.DB112_length_654_mean_cov_11.629969419.docx]

**DB112_length_654_mean_cov_11.629969419**

TCTTTGTGTTTTGCCCTGTTTTACGGTGAGGTAGGAGGGAACCCATCTGGGGACCGGTAGGTGCAGGTGCAGTAGGACGTGGGACTTTT
 >chr10:1176647-1176928 + E=2e-157
GGACCCGTCCTTTGGTGCAGCTCGCCAGGGATGAGAGGCACCTCCCTACTTGGGTCTTCAGGAGCTGGTCCAAGGAGCTTCGAATCTAA

GTCATCTAGAATGACCCTGAAATGACTGACAGCCCCGGGCCCAAGAAAAACCCATAACCACCTCAGATGGATCTGACGTGGCTAAGGGA

CAAACAGCAAATAT|CAAAAAAAT|ATCAAAATTAAAATTGTTTTTATAACTTTAGATTTATTAAAGTTCTTTAAAAATGCATTAAGCA
 >chr10:1180649-1181013 + E=3e-207
TACTAGAAAAGATGGATGAATTCAGCTACATTAAAATTAAAAGATTCTGTTCATTATAAGACACCGTTAGGGTGAAATGATAAACCACA

AGTGGTAGTTGCAACATATATAATTCACAAAGGACTGCTATCCAGGATAGTGAACAAAATAAGATTTTATATATCTATAGGAGAATTGG

AAATTGAACAGCAGACCTGAATAGGTATGTAACAAAAGAGAAAATATGAGTAGCCAATAACAAAATACAGAAATTGAGAACTTGAAAAT

TAAGGCCAAAATGAAATGCCATTTCACAGTCAT
