# Supplementary material for: Chromothripsis during telomere crisis is independent of NHEJ, and consistent with a replicative origin
Source: Genome Res. 2019 May;29(5):737–49. doi: 10.1101/gr.240705.118 (PMC6499312; doi:10.1101/gr.240705.118)
Supplement: Supplemental Material [file supp_gr.240705.118_Supplemental_file_1.zip › contigs/annotated_contigs/DB112/contig.2.DB112_length_669_mean_cov_14.9237668161.docx]

**DB112_length_669_mean_cov_14.9237668161**

AGGACACAAACAAATGGAAGAATATTCCATGCTCATGGAGAGGAAGAATCAATATCGTGAAAGTGGCCATACTGCCTAAAATAATTTAT
 >chr6:44731828-44732188 - E=7e-202 p=1e-03
AGATTCAATGCCATCCCCATCAAGCTACCAATGATTTTCTTCACAGAATTGGAAAAAAAACTACTTCAAAGTTCTTATGGAACCAAAAA

AAGAGCCCACATAGCCAAGACAATCCTAAGTAAAAGAACAAAGCTGGAGGCATCATGCTACCTGACTTCAAACTATATTACAAGGTTCC

AGTAACCAAAACAGCATGGTACTGGTACCAAAACAGAGATATAGACAAATGGAACAGAACAGAGGCCTCAGAAATAACACCACACATCT

AC|AA|AAAACAGAACAGAAAAACTGGAAACTCTAAAAAGCAGAGCGCCTCTCCTCCTCCAAAGGAACGCAGTTCCTCACCAGCAACGG
 >chr6:44727023-44727334 - E=2e-175
AACAAAGCTGGATGGAGAATGACTTTGACGAGCTGAGAGAAGAAGGCTTCAGACGATCAAATTACTCTCAGCTACAGGAGGAAATTCAA

ACCAAAGGCAAAGAAGTTGAAAACTTTGAAAAAAATTTAGAAGAATGTATAACTAGAATAACCAATACAGAAAAGTGCTTAAAGGAGCT

GATGGAGCTGAAAACCAAGGCTCGAGAACTACGTGAAGAATGCAGAAG
