# Supplementary material for: Chromothripsis during telomere crisis is independent of NHEJ, and consistent with a replicative origin
Source: Genome Res. 2019 May;29(5):737–49. doi: 10.1101/gr.240705.118 (PMC6499312; doi:10.1101/gr.240705.118)
Supplement: Supplemental Material [file supp_gr.240705.118_Supplemental_file_1.zip › contigs/annotated_contigs/DB112/contig.2.DB112_length_673_mean_cov_7.31500742942.docx]

**DB112_length_673_mean_cov_7.31500742942**

AAGAGGTGGATTTCTGCTGCTTCCAATCCCTGATCTCAACATTTCACGGATCATGACCACAAAGTAATTTCTGGCAACTAAATGATTTT
 >chr6:151568427-151568855 - E=8e-246 p=0e+00
AGATTGTTCCAAGAGAAGAAATTTTAAAAGTGATAGCTAAAGAAGTAGGTCATCCTCCAAGCAAATAAATTTAAAGATGGTTCATTAAT

TTGGAAAGGAAAAGGAGCAAAGGAGAGTAAGACTGCTCTACCTCAGCTGCGTTTTTATTCTTGAACTTGCTTTTTTCCAATTTGATTAA

TTTCTGGGGAAAAAGTTTTTTTGTTTTTTTGTTTTGTTTTGTTTTTTTAAATAGAGATAGGGTCTTGCTCTGCCACCCAGGCTGTAGTG

CAGTAGCATGATCATAGCTCACTGCAGCCTCATACTCCTGGGTGGAAGCAATCCTCTCACCTCAGCCT|TCCA|CGGTGGAACTGCAAA
 >chr6:151567394-15
GTGGTAGAACCTCTTTGGAAAGTCAATTTAGGAAGGGTTAAATTTTTTGTTTTTGTTTTTGTTTTTGTTTTTGAGACGGAGTCTTGCAC
1567643 - E=5e-138
TGTCACCCAGGCTGGAATGCAGTGGTGAGATCTCAGCTCACTGCAACCTCCCCCTCCCAGGTTCAAGAGATTCTCCTGCCTCAGCTTCC

CAAGTAGCTGGGATTACAGGCACCTGCCACCACACCCAGCTAATTTTTTGTG
