# Supplementary material for: Chromothripsis during telomere crisis is independent of NHEJ, and consistent with a replicative origin
Source: Genome Res. 2019 May;29(5):737–49. doi: 10.1101/gr.240705.118 (PMC6499312; doi:10.1101/gr.240705.118)
Supplement: Supplemental Material [file supp_gr.240705.118_Supplemental_file_1.zip › contigs/annotated_contigs/DB112/contig.2.DB112_length_677_mean_cov_10.4135893648.docx]

**DB112_length_677_mean_cov_10.4135893648**

CACCATGCCTGGCTAATTTTTGTATTTTTGGTAGAGATGGGCGGGGGGGGTCTCACCATGTTGGCCAGTCTAGTCTTGAACTCCTAACC
 >chr10:987404-987765 + E=2e-196
TCAGGTGATCCGCCCGCCTTGGCCTCCCAAGTGCTGGGATTACAGGCGCGAGCCACTGCACCCGGCCTGATCTTGCTTTTTAAGCGATT

GCAATTCCACCTGGTGCTATGGTGATCTGGTGTTCCATGGCCTGTTTACCAGAAGAGCAATGCTTTTGTTGTGCTATATTGTTATTTCA

GTTATTTTTTTATTTTTTATTTTTTGTCTTTTTTTGTTTGTTTGCTTTGTTTTGTTTTGTTTGAGACAGGGCCTCAGCCTGTCACCTAG

ACTGG|TG|AACCTCTGCTTCTCACCTGCATACAGTAATTTCTATGTGAAACATCTTTTCAAAGTCTCGCAGTGGCCTCCTGGACACAG
 >chr10:986495-986809 - E=4e-177
TCTGGCATTGACCGGCCACCCCAGGATGAGACTAGCTCACACCTCTCCTGCGCTCCCGCACTTCTCCCGTCATGCCTTCTGGTCCAAAT

GGTTCCATTTTGTCTCTTTCTTATGTACCCCACAAATGCCCGTGCTATTTTTCTGTTTCAAAAACTTTCTTTCCACTGTGGGAAACATG

TCAGTCCTTGTTCCTCTAATGCTGCTGGGCTCTTCATGCCCACTTCAGGCACTGGG
