# Supplementary material for: Chromothripsis during telomere crisis is independent of NHEJ, and consistent with a replicative origin
Source: Genome Res. 2019 May;29(5):737–49. doi: 10.1101/gr.240705.118 (PMC6499312; doi:10.1101/gr.240705.118)
Supplement: Supplemental Material [file supp_gr.240705.118_Supplemental_file_1.zip › contigs/annotated_contigs/DB112/contig.2.DB112_length_692_mean_cov_7.15895953757.docx]

**DB112_length_692_mean_cov_7.15895953757**

ATTTTCCAATTATACTACATTGCTAAATAGTTACATTGAGACTTGGCTTTGTATTTTTTAATACACAAAGGATATCTTAAACTGCAATT
 >chr11:38400559-38400918 - E=3e-204
TTATGCTCATATTCATCATAAAGTTGTGTTATATGTTTAAATATAAAAAAAAACTTATGCCTTCCTGACTCACCACCTTCAATAAGAGA

AGAAATCATTGGCAGTGTCATCAATATTACCTTAACAAACCAAAATAATTCCATATTTCATATTTATTTTTTCTGTCTTCTGCCTACTT

CTAAACAATCTATGTCCTAATAGCCTTGTGGTCACAGAGTATAGGCCCCAACATTTTATAGTATTTCTTTAGCAATAATGTAACAAATG

TTA|CAT|TATATATATATGCTTAATAACATCTGAGTAAGAGTATTCACATGTTTACTTTTTAACTATTATAATAATATTCAAATTATT
 >chr11:38390568-38390899 - E=1e-182
TTGATCACCAAAGAAGAAAGTGTTGAGTTATTGAAAACTATAAGATCTATACAAAATGAAGGCTGGTAATCATGTTTAATTAATATCAT

GGATTTGACCATTGGTCTCTAGACAGTTCGCAACACACAAGAGCTTGACCCAGAGCATATTCCACTTATTATGCTCTGCCAGTTACACG

TTCCATACTCCTAACCTACAACACTAAAAAAAAGACAAGAAAATCATGTCGTCTTCTCAGGCTGTTGCTTT
