# Supplementary material for: Chromothripsis during telomere crisis is independent of NHEJ, and consistent with a replicative origin
Source: Genome Res. 2019 May;29(5):737–49. doi: 10.1101/gr.240705.118 (PMC6499312; doi:10.1101/gr.240705.118)
Supplement: Supplemental Material [file supp_gr.240705.118_Supplemental_file_1.zip › contigs/annotated_contigs/DB112/contig.2.DB112_length_697_mean_cov_6.22668579627.docx]

**DB112_length_697_mean_cov_6.22668579627**

TGATCCCATATGGCTGTAATATGAAGTACTTAAATCTCCTCCAGTTGAAGTAGAAAATATGTCAGAACTCATTAAGAAGTTATCCATAT
 >chr5:103021255-103021648 + E=1e-224
ACTGTATTGATTTTTATAAATTGCTTCTTAAATTATAGTTCAGCATGACACACATGAACATCACTGAGATCAGGTATTATTTGGACTAA

ATTAACACATTTTGCAAGTAGTTACTGTCATAGCTATTCTCTGATGCTATGCCATGATAATCCTTGTCCTTTGGTAAATTATTTAAAAC

CCAGACTTTTATTTTTATTCTCATCAAAGGTATATGTTGAATAAATTGTATTTATTGTTATGTTATAGACGTCAACCCTGGTGCCTAAC

AAATTATGACCCTTCTCAGGTCAATCTTCCCTA|TCCA|TTTGTGTTGCAATAAAGGAATGCTTGAGACCAGAAATTTTATAAAGAAAA
 >chr5:103023250-103023558 + E=2e-170
GAGGTTTATGTGGCTTACAGTTCTGAAGGCCGTACAAGAAGGATGGTACCAACATCTGCATCTGGTGTGGGCCTCAGGAAGCATACACT

CATGGCAGAATATATACCCTCATGGCAATGGGGAGCAGGCACCATGTGGGGAGAGAGGGATGGAAGGAGAAGACAGGGAGGTGCCAGAC

TCTTTCCAACAACCAACGCTCTAGATAACAAACAGAGCAGAACTCACTCATTACCATGGCACCAAGTCTTTCATGA
