# Supplementary material for: Chromothripsis during telomere crisis is independent of NHEJ, and consistent with a replicative origin
Source: Genome Res. 2019 May;29(5):737–49. doi: 10.1101/gr.240705.118 (PMC6499312; doi:10.1101/gr.240705.118)
Supplement: Supplemental Material [file supp_gr.240705.118_Supplemental_file_1.zip › contigs/annotated_contigs/DB112/contig.2.DB112_length_705_mean_cov_10.4255319149.docx]

**DB112_length_705_mean_cov_10.4255319149**

GCTTTGGCCTCCCAAAGTACTTGGATTACAGGCATGAGCCACCATGCCCGCCCCAAGCATTCCATTTTAAAACACAACCCCTACTCTCT
 >chr21:36037192-36037601 - E=2e-234
GGCATCCTTCTCTGCCTTATTTTCCCCCATAGCAGTTATCTTCATCTGACACATAATATATTCTAGTTGTTATCTGTCTGCCCTACCTC

ACCCCACTAGGATGGTGATTCTGTGAAAGCAGGGGTTTTTGCTTGTCATTGCCTAGGATAGACCAGGCACATAGTAGAGCTCCATAAAT

ATTTCCTGAATGAGTGAATACACACGTGCCTTCAAATACATATGCACCAACCTGAACTAGTTTTGGAAAATACTTTTCTCAGTTAGTTT

TATACTTTGGTGGGCAAGCTAATGTCACAAGACACACTATTCACAGAAATT|CT|GCGTAACACAGAGGAGCTGGGAGGCTGGTCTCAC
 >chr21:36217508-36217806 - E=2e-167
CAGGCCAGGCAGCCATAGGCAGAGACTCAAGGTAGACAGGCCAGGGGGAAGTTGTGTACCTTTCGAATCGGGAAAGGATTGAGGAGGGG

GAATGTTGCCGGACAGAAGGCTGTCCTGGGCGTCCATCCTTGCCCTCCATCCCTGTCTCCATCCCTGGCAAGGATGGAACCTTCCCTTC

CTTCATTTCCTAACTGGCTCAAATGTGGTCACCAAGGTGAATAGGAGGCACTAGTGTCTTGGAATAAACAGAACTTTCCCTCCC
