# Supplementary material for: Chromothripsis during telomere crisis is independent of NHEJ, and consistent with a replicative origin
Source: Genome Res. 2019 May;29(5):737–49. doi: 10.1101/gr.240705.118 (PMC6499312; doi:10.1101/gr.240705.118)
Supplement: Supplemental Material [file supp_gr.240705.118_Supplemental_file_1.zip › contigs/annotated_contigs/DB112/contig.2.DB112_length_712_mean_cov_10.5997191011.docx]

**DB112_length_712_mean_cov_10.5997191011**

CTGGCCCCACCCACACATGCTCTTCACCTTTGAACGACAGCAGCCTAGGCCATCCGGCTTTGCACAACCGGAACAGCTTCCACTTGGAA
 >chr10:1416542-1416788 - E=3e-127
AGCAAAAGTTTCACCACAGTCATGACAATATAAAGCGTTGGCCTTTCTCCCCCAGATGCTGTCCTTTAATGCGTTAAAACTTTAGACTT

AGCACCACATTCCGGACACCGTATCAAACAAGGTCACGTGCGTATGAGGCTCTGGAGGGTGCCCGTGG|TGT|GAGAAGAAGGAGGAAT
 >chr10:1391745-
GGAGGGTGGAAAGTTGCCCATAGTGAAGGAGGCAAGTTTAAAGGGAAGGGTAGAGGCATGGAGAATTGGTTGTGGGGAGCAGCCAAGGC
1392208 - E=8e-255
AGGCGTCCCCACAATTGACCTGCCACCAAGGGAACGTGGGTGAATGACCAAGGCAGGCTTCCCCACGGAGATCAGACATCAATGGAACA

TGGATGAATAATCAGAGAGGCGTCCCCATAAATGATTAAACACCAAGGGAAGGCTGCCTTCCCAAGTCCGTGACCAGCGCCGGAGTTTT

GGGTCAATGGATAAAATGTGTCTCCTTTGTCTCTACAAGAAATGAAAGGAATTGAAATTAAGAGAAGGGAGAGATTGAAGGGTGGCGCC

AAGATTGAAAGGAGAAAGAGGTTGAGGGATAGGGAGGGAGGTTGGCGAAGAGAGTAAAGAAGAGGCCACTTACTGGATTTAAAATTGGT

GA
