# Supplementary material for: Chromothripsis during telomere crisis is independent of NHEJ, and consistent with a replicative origin
Source: Genome Res. 2019 May;29(5):737–49. doi: 10.1101/gr.240705.118 (PMC6499312; doi:10.1101/gr.240705.118)
Supplement: Supplemental Material [file supp_gr.240705.118_Supplemental_file_1.zip › contigs/annotated_contigs/DB112/contig.2.DB112_length_725_mean_cov_12.5972413793.docx]

**DB112_length_725_mean_cov_12.5972413793**

TTATTTGTATTGTTGTCATTTTTAAAACTCAGAACAAAACTGATATACATTGACTTTTTTTTTTTTTTTAAGTAACTGACAAATGACAT
 >chr5:103038746-103039113 + E=1e-194
GAGACCACCAACTTCTTTCTCATGGGTATCACGATATTTAAATATGTCCCCTTTTAGCAAATGAAGCTTTAAACTAATCAGCTATTAAA

CACATGATTGCCAAGCACCTTATTTTCAAAGAATTTATCCATATTCCCTTAGGATCAGGCCAAATGGCTTAACTGATTTCACTATTCCC

CCTTAAGGTCACTTGGTTAATGACAATAAAACACTTAATTATAGTAGAGTCGCAGATCAAGCTTCCCAGCTCACTCCAGTTTTACTGAG

AA|AAAATCAGCGGTCACTTTGCAATTTTCTTTTTCCTAAAAGTATAAATTAGGAATTAATATAATACCCAGTACACACTAAACAAGCT
 >chr5:103039981-103040350 + E=3e-210
CTGTCATGGTAGAGATTTTGCACAGAGCTTCAAAGCTCGTGTTAAAGGAAATAAGGAAGCTGTAACAGGGAACGTGTCTGTTCTTATTA

CTTGGGTTAGAGGAATTTCTGGAAAAAGTTCTGTTCCTAGAGGAAATCTAATAAACCACTTCTGAGCTGCATTTTATTTTTACTTTTAC

TTTTTACTTTTCGACAAACTGATTAACATTTCAAGGTTTTTCTCCCCCTCATGTATGATTTTGTAGACTTCATTGACTCATGATTCAGA

CATAAGGTTGTATG
