# Supplementary material for: Chromothripsis during telomere crisis is independent of NHEJ, and consistent with a replicative origin
Source: Genome Res. 2019 May;29(5):737–49. doi: 10.1101/gr.240705.118 (PMC6499312; doi:10.1101/gr.240705.118)
Supplement: Supplemental Material [file supp_gr.240705.118_Supplemental_file_1.zip › contigs/annotated_contigs/DB112/contig.2.DB112_length_728_mean_cov_7.09340659341.docx]

**DB112_length_728_mean_cov_7.09340659341**

AGTGGAAGTTGAAACCAGAAGAGTATAGGCATCATCTAGGGAGAAACTGTAGCGTGAGCTAAGGGATGCATATGGAACATGAGATATCA
 >chr12:81500905-81501310 + E=7e-229
CAGCCAATAAAAGATGAAGCAGAGAAAACAAGATGCAACAAACATATTGAAAAAGAGCTGGCAAAGAAGTAGGAGAAGAATAATGCAAA

TAAATGACAAAGTAAATTCTGTGGAATTGAGGGAAGCCCCTTTTTTGGGTAAAATATCATGAACATAATTGAATATTGATCACAAAGAA

TGGTAGAATAGAAGTCATAGATAAAGGAAAAGAGAATAATGATATTATCAAGAAATATTCTAGCAAGTAAATAGTTTCCCTGCCAGAAA

TGTTTTTTTTTTCAGGTACAAATATATACATACATATATATATATATAT|GT|ATTAGATTCTCATAAGGAGAATGCAACCTAGATTCC
 >chr12:81499791-81500112 - E=2e-181
TCACATGTGCAGTTCACAATATGGCTTGTGTTCCTATGAGAATCTAATGCCACCCCTGATCTGACAGGAGTTGGAGCTCAGGCAGTAAT

GTGAGCAGTGGGGAGCAGCTATAAATACAGATGAAGCTTTGCTCAGTCACCTGCTGCTCACCTCCTGCTGTGTGGCCCAGTTCCTAACA

GGCCACAGACTGGTACCAGTCCATGGCCCTAGGGTTGGGGACCCCTGCTTTAGATGACATTCAAAACCCATTGATCTACTCTGTTTCAT

TTCCACTCATCCAACTTT
