# Supplementary material for: Chromothripsis during telomere crisis is independent of NHEJ, and consistent with a replicative origin
Source: Genome Res. 2019 May;29(5):737–49. doi: 10.1101/gr.240705.118 (PMC6499312; doi:10.1101/gr.240705.118)
Supplement: Supplemental Material [file supp_gr.240705.118_Supplemental_file_1.zip › contigs/annotated_contigs/DB112/contig.2.DB112_length_765_mean_cov_5.79607843137.docx]

**DB112_length_765_mean_cov_5.79607843137**

CAAACAGCCCAGGTTCAAATTTAGTCTCTACCACTGTGAAATTGGGCAAATTGTCTAACCTCTGTATGCTTCTGTTTCTTCATTTGCAA
 >chr5:105891474-105891850 - E=2e-214
AGTGGGGTTTATATTACTGACATAATCGTTGTGAAAATTAAAATATTTTAAGCATAAACATTTAAAAATAATGGTGGCAGACAGCAAGT

CCTAAGTGTAAACATTATTATTATTTGTTACAACGCAAATTTCAACTTCTCTAGTTCCAAACATGTCATTTCTTGTGTATTCCACATAT

AGTTTACCATAAGCTTTTCAAGTTTCATCTGTGAAACCAGTGGGTATTATAAGTAAAACTACATTAATTGTATAGATTAATTTGGAGGA

AAAGATATATATATATATAT|CATATA|TAACTCAGCTGGATTGCATTGCTAGCGGTAGTTGTGGATAGAGCCAGACAAGGTGAAGACA
 >chr10:1235353-1235712 + E=4e-201
CATGTGCTCAGGCCTAGAAATGAAAGCCTGGCTTGTGTTTAATTTTAACTCCTGTGGCTGGAAATGTGAAAATGCATTAACTCCTGGCT

CATTCCTCCTGAAGTGGGCATCGGCTGATTGAAGACGGAGCTCGAGTCCCAAGGACAGAGGGTGTCTGCAGCTGCAGGGGCACCTACTC

AGGCCTGTCCCACCCTGTTCCTCAAGATAACACAGCAGCACATACCTGGTGACCCAGTCCACCCAGAACACATGGCCCTACCTCCTTCC

CCAGAACACGTGGCGCTACCTCCTTCCCCAG|TTCACAGCACAAAATGTCCCCTGG
