# Supplementary material for: Chromothripsis during telomere crisis is independent of NHEJ, and consistent with a replicative origin
Source: Genome Res. 2019 May;29(5):737–49. doi: 10.1101/gr.240705.118 (PMC6499312; doi:10.1101/gr.240705.118)
Supplement: Supplemental Material [file supp_gr.240705.118_Supplemental_file_1.zip › contigs/annotated_contigs/DB112/contig.2.DB112_length_767_mean_cov_9.24641460235.docx]

**DB112_length_767_mean_cov_9.24641460235**

ACGGTGGCTCACACCTATAATCCCAACATTTTGGGAGGCCGAGGCGGCTGGATCACCTGAGGTCAGGAGTTCAAGACCAGCTTGACCAA
 >chr10:1135957-1136390 - E=1e-239
CATGGTGAAACCCCACATCTACTAAAAATACAAAACTTAGCTGGGCGTGGTGGCATGTGCCTGTACTCCCAGCTACTCGGGAGGCCGAA

GCAGGAGAATCCTTTGAACCAGGGATGCGGAAGTTGCAGTGAGCCGAGATCCTACCACTGCACTCCAGCCTGGTGACACAGCAAAACTC

CATCTCAAAAAATAAATTAATTAAAAATAAATTTTAAAAAACTACAAAATTTAGCCAGGCGTGGTGGCGGGCACCTATAATCCCAGCTA

CTCGGGAGGCTGAAGCAGGAGAATCGTTTCAACGTGGGAGGCGGAGGTTGCAGCAAATGAAGATTGCACCACTGCAC|CAGTGCCCTCT
 >chr10:11
TCACTTGCTCACTCGCCCTCCCTCACACCTCCACGCTGCCAGGCTGAGAGGTGGCATCGCCTGTGTGCACAGAGGGACAGGTGTGGGTG
32874-1133208 + E=4e-189
GGGCCCGTGGCTCTGAGCGCCTGTCTGCCCGTCCACAGGGCCCTGATGAGCTGTGGAGCCTTCCCCTCCCCTTTCCCAGGACCTCCTCT

CCTTTGGCCATTCCTGCTGTCACAGTGTCTTCTCTTTCTTGCCCTGGACATTTCCCCTTAGTGTGTAGACAGGCTGGCGCCTTTCCCGT

GTTAGACCCCCTCCTCCGTGCTGCCTTCTTACCTGTCTTCCTCCCCTGGCATCCCA
