# Supplementary material for: Chromothripsis during telomere crisis is independent of NHEJ, and consistent with a replicative origin
Source: Genome Res. 2019 May;29(5):737–49. doi: 10.1101/gr.240705.118 (PMC6499312; doi:10.1101/gr.240705.118)
Supplement: Supplemental Material [file supp_gr.240705.118_Supplemental_file_1.zip › contigs/annotated_contigs/DB112/contig.3.DB112_length_1123_mean_cov_14.5120213713.docx]

**DB112_length_1123_mean_cov_14.5120213713**

CTGGTTTCAACTTCCACTTTGATCTCCAGCCCTAACCTTCTTTTCTGAGCTCCATACCTTATAGCCAAAAGTCTGCAGGACATCTCTAC
 >chr12:81500712-81500923 - E=7e-115 p=3e-02
TTAAAAAAAATAACCTGTCTAAAATTATACTCATTTCACTCCACCTCCACCCCCACCCCCACAGCTCCATCACAACTGCAAACTTGCTC

CTCCTCCTGTTTTCTAAATTTCAAGGAACTAT|G|AGTCTCCACTCATGGAGGAAGGGCCGAGGAAAGAGACAAAATACTTTTGCTAAG
 >chr12:81501789-81502344 + E=6e-222 p=0e+00
AGTAGGATGCTGGGAAGAGGTAATATTTTTGAACAAACATGAGATCAGTCACAGGGGAATTCAGAATCATTATATAAATTAATTCTGTG

CTATGCTGTAGGTAATCAAATTTGCTATTATTGAAATACATAGGGTCATTTGACTTTGATTAAATGATTCTTCCTCCTGTTGCTTTCTG

TGGCTATTATTATTATTATTATTAATTATTATTATTATTATTATTATTGTTATTTTGAGACAGAGTCTCGCTCTGTCGCCCAGGCTGGA

GTGCAGTGGCGTGATCTTGGCTCACTGCAACCTCCACCTCCTGGGTTCAAGCAATTCTCCTGCCTCAGCCTCCCGAGTAGCTGGGACTA

CAGGTGCCCGCCACCACGCCTGGCTAACTTTTGTATTTTTAGTAGAGACAGGGTTTTCACCATGTTGGCCAGGATGGTCTTGATCTCTT

GACCTCATGATCTGCCCCCCTCGGCCTCCCAAAGTGCTGAGATTACAGGCGTGAGCCAC|AGCACCTGGACAAT|TTTTTTTTTTTTTG
 >chr4:2724997
AAGCAGAGTCTAGCTCTGTCGCCCAGGCTGGAGTGCAGTGGCACAATCTCAGCTCGCTGCAACCTCCGCCTCCTGGGTTCAAGCAAATC
-2725313 - E=6e-154
TCTGCCTCAGCCTCCCGAGCAGCTGGGATTACAGGCACCCACCACCACATCTTGCTAATTTTTGTATTTTCAGTAGAGAGGGGGTTTCA

ACATCTTTGGCCAGAATGGTCTTGAACTCCTGACCTCATGATCCACCCGCCTTGGCCTCCCAAAGTGGGATTACAGGCATGAGCCACCG

TGCCTGGCCTAAAGGATACTGTTTTTTGGTTTGAG|GCGGAGTGTCGCGCTGTCGCCCCG
