# Supplementary material for: Chromothripsis during telomere crisis is independent of NHEJ, and consistent with a replicative origin
Source: Genome Res. 2019 May;29(5):737–49. doi: 10.1101/gr.240705.118 (PMC6499312; doi:10.1101/gr.240705.118)
Supplement: Supplemental Material [file supp_gr.240705.118_Supplemental_file_1.zip › contigs/annotated_contigs/DB112/contig.3.DB112_length_514_mean_cov_5.93774319066.docx]

**DB112_length_514_mean_cov_5.93774319066**

GCAAAGTCACGCACGCAGTGATTAATTCATTTTATGCAGTTAATGGGAAGCGGGCTCAAGGCT|TCC|CATTTATATATAAATATATAT
 >chr10:1222638-1222701 + E=3e-26 >chr5:163756276-1637
ATATTTATATATAA|ATATAT|ATATTTATATATAAATATATATATTCCCATTTATATATAAATATAAAATACTGGGTGTTCACATACA
56317 + E=5e-07 p=0e+00 >chr5:105891084-105891490 - E=2e-206
TGACATAGAAAACCTCTCTTCTGCTTCTTTCCTCTCCTCTTTCTTCTTCTTCTTCTTGTTTTTCTGTAATGCCTATCCATAAAATTTTA

TATTATTCCTCAAAAATATCTTGCATATGTACTTCAGATATTGATAAAAAATTAATATTTCCACTTCTTTTCTAAATGCTATTTTTCAT

CTATTATATTGCCTCTATTTTTGCTTCTGGTTTATTGGCAAATAGTTTATTTGCATAACGATGATACAATTTGTTACATGGTGACCTAA

TGAATTCCAATAATTTGTCTATAGAAGTATAATTATTTTCTGAAAATATCTGTAAATAATTAGAGGTTTTTTC
