# Supplementary material for: Chromothripsis during telomere crisis is independent of NHEJ, and consistent with a replicative origin
Source: Genome Res. 2019 May;29(5):737–49. doi: 10.1101/gr.240705.118 (PMC6499312; doi:10.1101/gr.240705.118)
Supplement: Supplemental Material [file supp_gr.240705.118_Supplemental_file_1.zip › contigs/annotated_contigs/DB112/contig.3.DB112_length_661_mean_cov_10.8925869894.docx]

**DB112_length_661_mean_cov_10.8925869894**

AGACACCGGTTAAAGGTTGGATTTTGGACGGCCAAAGGTGATCCTGGCACAGGGTAAGGGGGGGACTAATTGC|GGACCTAGCCCACAG
 >chr7:56727730
GCATAGCAATGAGTCTCATATCTAAACCAGTCAATAGAAGAGATGTTGAATCTCATCCCTAGACTTGGAGAAATGAGTAAGATCATGGG
-56727883 + E=3e-56 p=2e-03
TTCATATAGGCACAAATGTTTCAGAGTGCATTGCAAGTCTTACACATAT|ATACAGCCACACTTGACGCAAAGAGTAG|TATAACAAGG
 >chr7:567
TCCAGTACACAGCTGAGATTTTGACTCTCATATGCATACACTGCCAACAGTAAAGAATGTCACACTCCCACATAGACACAGCCCACTGT
27917-56728080 + E=3e-62 p=0e+00
TGAGGTTCTGAATCTCACACCCAAAGGCAGTCAGAAGTTGGTATTTTTACTCTTATATGT|GGAT|ACTAATATACCCCTGGCTCAGTA
 >chr7:63746800-63747048 -
TTCAGATGATGTGACTCTCCTGCCTGGTTCTTCCCACAGGTGGGATTCTGGCATATACCTAGGTACGGATCACAGGAATAATAATGACT
E=2e-134
GTCATATGAGAGCCCAGCCAATAGAAGAGATTCTGACTCTCATGGCTGAGCTTAGAACAATCAGTAAGGTTCTGGGTCTCCTAAATGTA

TCACAATTACAGAGGATTATGACACTCATAAATATTCTATAAA
