# Supplementary material for: Chromothripsis during telomere crisis is independent of NHEJ, and consistent with a replicative origin
Source: Genome Res. 2019 May;29(5):737–49. doi: 10.1101/gr.240705.118 (PMC6499312; doi:10.1101/gr.240705.118)
Supplement: Supplemental Material [file supp_gr.240705.118_Supplemental_file_1.zip › contigs/annotated_contigs/DB112/contig.3.DB112_length_792_mean_cov_7.52398989899.docx]

**DB112_length_792_mean_cov_7.52398989899**

TCACCCAGGCTGGAGTGCAATGGTACCTGGGATTACGGGCACACCACCACACCCAGCTAATTTCTGTATTTTTAGTACAGATGGGGTTT
 >chr7:75311383-75311491 + E=4e-53
CACCGTCTTGGCCAGG|TTG|TCTCCTGGGAAATGGGAGTATTAATTCCCTCTGGGCTGCTGTTTTTTGTTTTGTTTTGTTTTGTTTTG
 >chr7:75311512-75311974 - E=3e-240 p=0e+00
TTTTGTTTTGTTTTCTGTCTGTAAAATGAGGATGTTGGCCAGGCACCGTGGCTCAATCCCGTCATCTCAGCACTTTGGGAGGCTGAAGC

AGAAGGATGGCTTAAGCCCAGGAGTTCAAAACCAGCCTGGGCAACATAGTGAGATCCCACCTTTAGAAAAAAAAAAACTAGCTGGCTAT

AGTAGCATGTAGCCTCCTGTAGTCACAGCTACTGAGGAGGCTGAGGCGGGAGGATCACTTGAGCCCAGGAGTTCGAGGCTGCAGTGAGC

TATGATCGCACCACTGCACTTCAACCTGGGTGACACAGCAAGACACTGTCTCTTAAAAAAAAAAGATGATAGGCTGGTGGGGGTGACTC

ATGCCTGA|AATCCCAGCACTTTGGGAGGCCAAGGTGGGTGGATC|GCCGGAGCTTAGGAGTTTGAGAACAGCCTGGCCAACATGGTGA
 >chr7:75313508-75313760 + E=1e-136
AACCGTGTCTCTACTAAAAACACAAAAAATTAGCCAGGTGTGGTGGCGCGCAACTATAATCCCAGCTACAGGGGAGGCTGAGGCAGGAG

ACTAGTTTGAACCCGGGAGGCAGAGGTTGCAGTAAGCCGAGATTGCATCACTGCACTCCAGCCTGAGCAACAGAACAAGACTCC
