# Supplementary material for: Chromothripsis during telomere crisis is independent of NHEJ, and consistent with a replicative origin
Source: Genome Res. 2019 May;29(5):737–49. doi: 10.1101/gr.240705.118 (PMC6499312; doi:10.1101/gr.240705.118)
Supplement: Supplemental Material [file supp_gr.240705.118_Supplemental_file_1.zip › contigs/annotated_contigs/DB113/contig.2.DB113_length_263_mean_cov_6.48669201521.docx]

**DB113_length_263_mean_cov_6.48669201521**

TTTCCACACACCAATAACATTCAAGCTGAAAGTTATAT|CAAGAAT|AACTCATGGGCCAGGTGCAGCGGCTCATGCCTGTAATCCCAG
 >chr3:57424345-57424390 + E=9e-16 >chr10:12314432-12314632 - E=3e-97
CACTTTGGGAGGCCAAGGCAGGTGGATCACCTGAGGTCAGGAGTTCAAGACCAGCCTGGCCAACATAGTGAAACCCCATCTCTACAAAA

AATATAAAAAGTAGCTGAGCACGGTGGCACATGCCTGTGGTCCCACCTACTCAGAAGGCTGA|GGCAGGAGAATGGCGTGAACTCGGG
