# Supplementary material for: Chromothripsis during telomere crisis is independent of NHEJ, and consistent with a replicative origin
Source: Genome Res. 2019 May;29(5):737–49. doi: 10.1101/gr.240705.118 (PMC6499312; doi:10.1101/gr.240705.118)
Supplement: Supplemental Material [file supp_gr.240705.118_Supplemental_file_1.zip › contigs/annotated_contigs/DB113/contig.2.DB113_length_270_mean_cov_4.98148148148.docx]

**DB113_length_270_mean_cov_4.98148148148**

GTGGCGTTCTCTGTATTTCCTGAATCTGAATGTTGGCTTGCTTTGCTGGATTGGGGA|AG|CTCAGAGTAATTTGATCGTCTGAAGCCT
 >chrUn_gl000240:26713-26772 - E=3e-21 >chr1:143130550-143130760 + E
TCTTCTCTCAGCTCGTCAAAGTCATTCTCCATCCAGCTTTGTTCCGTTGCTGTTGAGGAACTGCATTCCTTTGGAGGAGGAGAGGCGCT
=2e-112
CTGCTTTTTAGAGTTTCCAGTTTTTCTGTTCTGTTTTTTCCCCATCTTTGTGGTTTTATCTACGTTTGGTCTTTGATCATGGTGATGTA

CA|GAT
