# Supplementary material for: Chromothripsis during telomere crisis is independent of NHEJ, and consistent with a replicative origin
Source: Genome Res. 2019 May;29(5):737–49. doi: 10.1101/gr.240705.118 (PMC6499312; doi:10.1101/gr.240705.118)
Supplement: Supplemental Material [file supp_gr.240705.118_Supplemental_file_1.zip › contigs/annotated_contigs/DB113/contig.2.DB113_length_349_mean_cov_5.57593123209.docx]

**DB113_length_349_mean_cov_5.57593123209**

TGCAGTCACGCAATCTCAGCTCACTGCAACCCCCACCTCCTAGGTTCAAGCGATTCTCCTGCCTCTCAGCCTCCCGAGTAGCCAGGATT
 >chr10:12408380-12408623 - E=2e-122 p=0e+00
ACAGGCATGCGCTACCAGGCCTGGCTAATTTTTTTTTTATTTTTAGTAGACATGGGGTTTCACTATGTTGGCCAGGCTCGTCTCAAACT

CCTGTCCTCAAGTGCTTCACCCACCTTGGCCTG|CCAAAGTGCTAGGATTACAGGTGTGAGCCACTG|TGCTGGGACTGACTTTTTGAT
 >chr4:140703578-140703716 + E=1e-71
CAAGCTGGCATGGTGAAACTAATATTAACAGTTTACATGGACACAAAGATAAAAGTGTTAGCTGATATATATATGTAGAAAAGT
