# Supplementary material for: Chromothripsis during telomere crisis is independent of NHEJ, and consistent with a replicative origin
Source: Genome Res. 2019 May;29(5):737–49. doi: 10.1101/gr.240705.118 (PMC6499312; doi:10.1101/gr.240705.118)
Supplement: Supplemental Material [file supp_gr.240705.118_Supplemental_file_1.zip › contigs/annotated_contigs/DB113/contig.2.DB113_length_370_mean_cov_10.6378378378.docx]

**DB113_length_370_mean_cov_10.6378378378**

TAGTTCATCCTAACCCAAGATGTATAACTTTTACAGATAGAAAAGACAATAGCAAAAAAAAGAGTTTATATGAGTCATAAGCAAGTATT
 >chr5:164899336-164899500 - E=2e-79 p=5e-02
TGAAAATGAATAAACACAAGAGTTGGTATATGACATTTAATAAAATAAATCTATATTTTACTCACCAAAGAAGA|GTAAAA|TTTTTTT
 >chr5:
GCAGATTTGAGTTCATTGTAGATTCTAGATATTAGTCCTTTGTCAGATGTATAGATTGTGAAGATATTCTCCCACTCTGTGGGTTGTCT
164897446-164897647 - E=1e-109
GTTTACTCTGATGACTGTTCCTTTTGCTGTGCAAAAGTTCTTTAGTTAAGTTCCAACCATTCATCTTTATTTTTATTGCATTTGCTTTT

GGGTTCTTGGTCATGA
