# Supplementary material for: Chromothripsis during telomere crisis is independent of NHEJ, and consistent with a replicative origin
Source: Genome Res. 2019 May;29(5):737–49. doi: 10.1101/gr.240705.118 (PMC6499312; doi:10.1101/gr.240705.118)
Supplement: Supplemental Material [file supp_gr.240705.118_Supplemental_file_1.zip › contigs/annotated_contigs/DB113/contig.2.DB113_length_397_mean_cov_7.04282115869.docx]

**DB113_length_397_mean_cov_7.04282115869**

AATCTACTTTCAGTCTCTGAATTTGCCTATTCCAAATATTTTGTATAAGTGGAATCATACAATATTTGTCCTTTTGCTTCTGGCGTATT
 >chrX:73465143-73465372 - E=2e-126
TCACTTAGCACAATGCCTTCAAGGTTCATCCATGTTATAGCATATGTCAGAACTTTATTCTTTTTTATAGCTGAATAATATTTCATTGT

GTGTATATGCCACATTTTGTTTATCATTCATTTTGTTTATGTACCATGTTT|G|CAGCTCACTGCAACCTCCGCCTCCCGGTTTCAAGC
 >chrX:73553319-73553486 - E=5e-89
GATTCTCCTGCCTCAGCCTCCCAAGTAGCTGAGACTACAGGCATGCGCCACCATGCCCAGCTAATTTTTGTATTTTTAGTAGAGACGGG

GTTTCACCATGTTGGCCAGGATGGTCTTGATCTCTTGACCTTG
