# Supplementary material for: Chromothripsis during telomere crisis is independent of NHEJ, and consistent with a replicative origin
Source: Genome Res. 2019 May;29(5):737–49. doi: 10.1101/gr.240705.118 (PMC6499312; doi:10.1101/gr.240705.118)
Supplement: Supplemental Material [file supp_gr.240705.118_Supplemental_file_1.zip › contigs/annotated_contigs/DB113/contig.2.DB113_length_427_mean_cov_11.4051522248.docx]

**DB113_length_427_mean_cov_11.4051522248**

GCACATGAGCCGTTGGCTCGATCTGCAAG|GAGCTTCCGACACAGTGGCTTCTGAGGTCTTTGAAACTAGATTCACTTTCAGATTTGAT
 >chr9:68421513-68421834 + E=3e-149
TATTTTATTTATGATTTGATATTTTCATTGAACCCCATGATATGATAAAGTTTTCCAGAACCTGCTAGAACAATTGACTTTGCTTTCAG

CATTATGAAGCCACTGGGGCATATAAGTAGTTCATTTATGTTTCTATTTAACCAGACAGGAACAAGTTGAGCTACTTTTCTGGCCACCT

TCGTTTAGACCTGTTTTTTTTTAGTTATTTTTGTTTGTTTGTTTTTGAAATGGGTCTCACTCTGTCACCTAGGCTGGAATGCAGTG|AT

TATACATATTTACTATTATTATACA|TAATAGTAAATATGTATAATCAGAGTTTGAGGTTTTTTCAATGAAGGC >chr9:68429195-68429244 - E=4e-13
