# Supplementary material for: Chromothripsis during telomere crisis is independent of NHEJ, and consistent with a replicative origin
Source: Genome Res. 2019 May;29(5):737–49. doi: 10.1101/gr.240705.118 (PMC6499312; doi:10.1101/gr.240705.118)
Supplement: Supplemental Material [file supp_gr.240705.118_Supplemental_file_1.zip › contigs/annotated_contigs/DB113/contig.2.DB113_length_430_mean_cov_5.17674418605.docx]

**DB113_length_430_mean_cov_5.17674418605**

AATTTAAAATAATAATAAATAATAAATTAATTTAAAAATTAAAAAAATAAATTGTGTCTCCCCAAAAATTCATATGCAGAAGCTCTAAC
 >chr7:67195966-67196124 - E=1e-83
TTCCCAGTGTGATGGTACTTGGAGATGGGGTCTTTGGGGGTAATTAGGTTTAGATGAGGTCATGAGG|GT|TCGCTACCTGCCTCTGAA
 >chr7:67194515-6719
GAGCCGTCCCTATCCTTGGATTAATTAGCGTGGTTTTTGAACTTTGGATACACAAATTCGTGCCGTACATGTCTCCTGCATCCACCTTC
4789 - E=2e-147
ATCTGCTCAGAGCTTCCTTTCCTTTAGTTTCCTGAGAATGGCTCTTTTCATGGCAGAAACATTCCACTCGCTGAACTCAGGTTCCTGGA

GCCAAAAGTGTCACAGGAAAGCCAGCTTCCAGGAACACAGACTCCGGTGGCTGTCGGGGAACCACTCAAGCCTCCG
