# Supplementary material for: Chromothripsis during telomere crisis is independent of NHEJ, and consistent with a replicative origin
Source: Genome Res. 2019 May;29(5):737–49. doi: 10.1101/gr.240705.118 (PMC6499312; doi:10.1101/gr.240705.118)
Supplement: Supplemental Material [file supp_gr.240705.118_Supplemental_file_1.zip › contigs/annotated_contigs/DB113/contig.2.DB113_length_434_mean_cov_8.20506912442.docx]

**DB113_length_434_mean_cov_8.20506912442**

AGAGTGAGACTCCATCTCAAAAAAAACTACACCGAGACACCATCTTTCCCTAGTCAGAACGGTCATTACTAAAAAGTCAAAAAACATTA
 >chr4:91306493-91306646 - E=4e-57
GACTTTGACACAGATATGGTTAAAAAAAAAAGGAACACTTACATACTATTGGT|ATGTA|AGTAAAGTGTCTTACACACAGAAAGACAC
 >chr4:91068053-91068340 - E=
AGTAAAGTGTCTTACACATGGAAAGCATTAAATGACTGTTAAGTTATTAAACAAAGTATTAAAAATAAATAAATACAATAGTAAATAAA
2e-158
TAAATCACTGAACAAATGAATTAAACAACTAAGTAAATAAATAAATAAAATAATCAAAATACAAGTCGTAGCCTGAAGACAATTAGAAG

AAACATTGTGGTTTGACTTCATAAATTCTTGAGATCATAAAAATGAATATTTAATCTTACGTGTTAATTACTTTTAAAAG
