# Supplementary material for: Chromothripsis during telomere crisis is independent of NHEJ, and consistent with a replicative origin
Source: Genome Res. 2019 May;29(5):737–49. doi: 10.1101/gr.240705.118 (PMC6499312; doi:10.1101/gr.240705.118)
Supplement: Supplemental Material [file supp_gr.240705.118_Supplemental_file_1.zip › contigs/annotated_contigs/DB113/contig.2.DB113_length_450_mean_cov_8.33333333333.docx]

**DB113_length_450_mean_cov_8.33333333333**

ATTTTTGTTGCTTTGTTTTTATTTACATGTAATTAAAAAAATCTTTTAATTGATATTAAACCAGGCATTTTGGAAGCAGAACACTCTCG
 >chr18:40417734-40417988 + E=2e-141
GTTTCTGTCTACACAGTACCAATGAAATTAGGTAAATATAGGACTATAGCAGTAAAACATGAAGTAGAAAAATAAACGAAGCATATCAA

TTAACATTTTCCAGGAAGGAAAATACCTGAGTGACTTTTTGGGGAGAAAAAGATGTCATGGCAATATAAAAACCTG|TACTAA|TCAAG
 >chr
CCAGATGGGTCCTGGCAATAGGCATCACAACACCCAGCCCATAATGACAAGAGAAATGCTAACATGACAGAAAGTTAGTAAACATATTA
18:40418942-40419132 + E=8e-103
AAAATCTTCCCTCATCCTAAACCAATCCTTTGCACACTGGAAAATGAGAAGAGCAAAATCCCCATAAAACAAATTAACAAAAATCAAGA

GAGGGAG
