# Supplementary material for: Chromothripsis during telomere crisis is independent of NHEJ, and consistent with a replicative origin
Source: Genome Res. 2019 May;29(5):737–49. doi: 10.1101/gr.240705.118 (PMC6499312; doi:10.1101/gr.240705.118)
Supplement: Supplemental Material [file supp_gr.240705.118_Supplemental_file_1.zip › contigs/annotated_contigs/DB113/contig.2.DB113_length_493_mean_cov_6.69371196755.docx]

**DB113_length_493_mean_cov_6.69371196755**

TTGGTGAGTGCTAATAAAAACAGCAGCTGACGTTTTATTCCTTGAGTGTATTGAAAAAACTCTGGCTTTGGAATCTGGCAGTTTTTACC
 >chr9:17626860-17627205 - E=3e-196
GGCAGTCCCAATTCTTCTATTTAGTAAGTTAAACATGTTATTCAGTCTTTGGCACCTCCAATACAGGGTAGTTGTGACAATTACATGAG

GAGAGGTACATCCAGCACACGACCAGGGCCTGACAGAAAGTGGAGGTTAAAGAAGATTATCCTGGCTCAGACCTGCTCAGGGAACCAGT

AATGGGGGAGGGGAAGCTGAGCCAGCCCCCAAATTTGGTAGTTAGAAAGGAGACTATTTTCAAGGCCTCCTCTTCCTC|TTCCTCGA|C

TCCCACAGGCTCCATCTGCTGTGTCCTGCGGCCAGAGCCTCCCTCTGGGAACCAAAACCAAAGAGCCAAGTGGACACCTCCCCAGCCTC
>chr9:17626009-17626149 - E=1e-72
ACAAGACAGTCCATCAGCTCAGGTAGGGAAGATGAGATGGGGAAGACTGA
