# Supplementary material for: Chromothripsis during telomere crisis is independent of NHEJ, and consistent with a replicative origin
Source: Genome Res. 2019 May;29(5):737–49. doi: 10.1101/gr.240705.118 (PMC6499312; doi:10.1101/gr.240705.118)
Supplement: Supplemental Material [file supp_gr.240705.118_Supplemental_file_1.zip › contigs/annotated_contigs/DB113/contig.2.DB113_length_511_mean_cov_6.16438356164.docx]

**DB113_length_511_mean_cov_6.16438356164**

ATTTTTTTTGTATTTTTAGTAGACGGGGTTTCACTGTGTTAGCAAGGATGGTCTCAATCTCCTGACCTCATGATCCACCCGCCTCGGCC
 >chr16:67921651-67921960 + E=2e-168 p=1e-02
TCCCCGTGTTGGGATTACAGGCGTGAGCCACCGCGCCCGGCCTATTCTTTACTTTCTAAATTGAAAAACTCAGCCAGGCATAGTGGCTC

AATCTTGCAGTATCACCTCCTCTAGGAACTATTCCCTAACCCTGACTACAAGCTGAATCAGAAGGCTCCTCTAGGTCCACACAGCTCCT

GGATTCCCTTCTGCCTCAGTACTGATAGCACTAGCTTGTTAT|AT|ATATTCCTTTTAACCCTCACAACAACTCTGTAAGGTCAGGATT
 >chr16:67922803-67923003 + E=9e-109
ATGACCCCCCCTTATATTTTATTTTATTTTTTGAGACAAGGTCTCATTCTGTCACTCAGGCTGGAGTGCAGTAACTCAGTCCTGACTCA

CTGCAGCCTTGACCTCCTGGGCTCAAGCGATTCTCTAGCCTCAGCCTCTTGTGTAGCTAGGACTATAG
