# Supplementary material for: Chromothripsis during telomere crisis is independent of NHEJ, and consistent with a replicative origin
Source: Genome Res. 2019 May;29(5):737–49. doi: 10.1101/gr.240705.118 (PMC6499312; doi:10.1101/gr.240705.118)
Supplement: Supplemental Material [file supp_gr.240705.118_Supplemental_file_1.zip › contigs/annotated_contigs/DB113/contig.2.DB113_length_515_mean_cov_6.9067961165.docx]

**DB113_length_515_mean_cov_6.9067961165**

TCATTCTGACATTTACAGATTTAACATCCTACTTCACAAGATGCTCATACCCTTAATCCATTACAG|CCGATG|CCTCAGCTTCAGGTC
 >chr9:68422112-68422178 + E=5e-16 >chr1:14313082
TGTTGGAGTTTTCTAGAGGCCCATTCCCGACCCTGTTTGCCTGGTATCAGCAGTGGTGTCTGCAAAACCATGGATTTTCGTGATCCGCG
0-143131179 + E=9e-200
AATGCTGCTGTCTGATCGTTCCTCTGGAAATTTTGTCTCAGAGGAGTACCTGGTCGTGTGAGGTGTCAGTCTGCCCCTGCTAGGGGGCG

CCTCCCAGTTAGGCTGCTCGGGGGTCAGGGGTCAGGGACCCACTTCAGGAGGCAGTCTGCCCATTCTCAGATCTCCAGCTGCATGCTGG

GAGAACCACTGCTCTCCTCAAAGCTGTCAGAGAGGGACATTTAAGTCTGCAGAGGTTACTGCTGTATTTTTGTTTG|AGAGAAGACTAG

ATCTGGTTAGTGTCAAAGTACCCCATATGGGTTTGGGCACTGGTTCTCGAGAAGTGAGTTTATCTTTATGTTC
