# Supplementary material for: Chromothripsis during telomere crisis is independent of NHEJ, and consistent with a replicative origin
Source: Genome Res. 2019 May;29(5):737–49. doi: 10.1101/gr.240705.118 (PMC6499312; doi:10.1101/gr.240705.118)
Supplement: Supplemental Material [file supp_gr.240705.118_Supplemental_file_1.zip › contigs/annotated_contigs/DB113/contig.2.DB113_length_552_mean_cov_6.94202898551.docx]

**DB113_length_552_mean_cov_6.94202898551**

TATATGAGATCCATTTTTATGGCAACATTTCTGGCCTGTAATTGATATTCTCTCCAAAAAATTTAGTCAAAGAATTAGACAAACTCCAT
 >chr10:19464392-19464854 - E=1e-251
CTGTGATCTGAAAATATTAAATTTTGAATAGAAACACAGAATTTTAAGGTCCAGTTTTAAATTTTGGTTGGCCTTACATCATTTTCCCA

AACACAACCTTTTTGCTTTATTTGAACTCCTGGGTGATCCTGAAATATGAATATTATTACATGCTCATTTGAAGTTCTGAATAAAATTA

ATGGAGAAGTGAATATTTATAAAAGTGTATTTAAAATATGCAAAATAATTATAATTTTTTCATAATATTTATAATTAAAATATTTGGAT

GCAATAATTTCAGCTGCAAGCATAGAATATTTTCCATATTACTGGAAGAGAGGCCCAAGGACTAGACCAGATGTTTACCTTTTAATATA

TATATATATAAATATA|T|AAAAATAATATCTATCGCCATGTGTCATTATGGAAAGAAATTCAACATGTGTTTTGGAGTAAAAAAATAA
 >chr10:19462156-19462248 - E=7e-39
GTTACAGAAAAATATTATAG
