# Supplementary material for: Chromothripsis during telomere crisis is independent of NHEJ, and consistent with a replicative origin
Source: Genome Res. 2019 May;29(5):737–49. doi: 10.1101/gr.240705.118 (PMC6499312; doi:10.1101/gr.240705.118)
Supplement: Supplemental Material [file supp_gr.240705.118_Supplemental_file_1.zip › contigs/annotated_contigs/DB113/contig.2.DB113_length_569_mean_cov_10.5061511424.docx]

**DB113_length_569_mean_cov_10.5061511424**

GAGTAAGTTCTAGGGATTTACTGCACAATATAGTACCTCTAGTTAACAGTATTGCACACTTAAAATTTTAAGAAGGTAGATCTCATATT
 >chr8:100948092-100948401 + E=3e-174 p=3e-03
ATATATATATAAAAGAAACCAGGATACATTTAGAGGTGGTGGCTATAATTTTATTGTGATGATAGTTTTATGGGTGTGCGCATACATCC

TAACTCATGAAATTGCGTACATTAAATATGTACAGTTTTTTTGTTTATGTTATACCCCAATAAAGCTCGTTTTTAAGAAGAGTCCTCAT

TTGTATAATGACGAGCAATAGATAAAGATGTTTTTAAAGCAA|AAGACCAATATATATATA|TATATATATATATACCCATATATATAG
 >chr8:100949685-100949928
AAAAAGCTTACAGAATAAGGATATAAAGAAGATATTTTTGTATAGCTTATGATATGTTTGGATTTTAAGCTAAGTGTTATTACAGAAGA
+ E=1e-129
GTCAAAAAGTTTTTAAAAAATTAAAAGTTTATAACGTAAAAAACTTACACTAAGGCTGAGTGCAGTGGCCCATGCCTTCAATCACAGCA

ACTCGAGAGGCTGAGGTGGGGGAATTGCTTGAGGACA
