# Supplementary material for: Chromothripsis during telomere crisis is independent of NHEJ, and consistent with a replicative origin
Source: Genome Res. 2019 May;29(5):737–49. doi: 10.1101/gr.240705.118 (PMC6499312; doi:10.1101/gr.240705.118)
Supplement: Supplemental Material [file supp_gr.240705.118_Supplemental_file_1.zip › contigs/annotated_contigs/DB113/contig.2.DB113_length_572_mean_cov_10.8094405594.docx]

**DB113_length_572_mean_cov_10.8094405594**

TTTATGGCTGCATAGTATTCCAGGGTGTATATGTGCCACATTTTCTTAATCCAGTCTATCATTGATGGACATTTGGGTTGGTTCCAAGT
 >chr10:13120445-13120684 - E=4e-132
CTTTGTTATTGTGAATAGTGCCGCAATAAACATATGTGTGCATGTGTCTTTATAGTAGTATAATTTACAATCCTTTGGGTATGTACCCA

GTAATGGGATTGCTTGGTTAAATGGTATTTCTAGTTCTAGATCCTTGAGGAATAGCCACA|C|ACAGTTTCTATGGTTCAGGAATCTAG
 >chr10:12932458-12932749 -
GCATGGCATATCTGGGTTCTCTGCATCATGGTCTGTCACAAGCCTGCAGTCAAGATATCGGGCAGGGCTAGGGTCTTATCTGAAGGCTC
 E=2e-163
GACTGGGAAGGATCCATTTCTAAGCTCACATGATGGTTGGCAGGATTTGGTTTCTTGCAGGTTGTTGAACTGAAGCCCTCAATTCCTCC

CTGGCTGCTGGCTGCCTTCAGTTCCTTGCCAAGTGTGCATCCCCAGCATGGCTGCTTGCTTCATCAAAGTGTGCAGACTAAGAGGG|AG

ATCGGAAGAGCGTCGTGTAGGGAAAGAGTGTTAAGATTAGT
