# Supplementary material for: Chromothripsis during telomere crisis is independent of NHEJ, and consistent with a replicative origin
Source: Genome Res. 2019 May;29(5):737–49. doi: 10.1101/gr.240705.118 (PMC6499312; doi:10.1101/gr.240705.118)
Supplement: Supplemental Material [file supp_gr.240705.118_Supplemental_file_1.zip › contigs/annotated_contigs/DB113/contig.2.DB113_length_576_mean_cov_10.4166666667.docx]

**DB113_length_576_mean_cov_10.4166666667**

ATAGGGTTG|AGGTGATCACCTAATCAATAGAGACAACACCCAACCAGATAGTGGTGTAACCATTTACTACCAGTCCTTGTCAGAGGGC
 >chr10:20773189-20773434 + E=1e-132
TCAAGGGCCATAAAATGAGCAGGACTTCACCAGCTACACATGGCCGTCTTGACTGACCCTGTCTTGCTGCCACTGGTGATCAGTACCCA

GCATCTGCCATCCAAGGTTCTGACCAAATCAAAGACGTTTCCTTGCAAGATATTAACATCCATCTGGACCAAGCCAG|A|GCCTGGAGC
 >chr5:27
CCCTTTGGTCAACCACTGTCTGGGGCAAAGGAGGACTCAGGGCTTCTGGAGCTGGTCTCTGGAATGGCTGGGATTCCATCCCCTGGTCC
80498-2780819 - E=2e-181
CCTGGTGGAGAGGCTGGAGTGGAAACAGGTTGAACCAGTTATGCTTAGAGGCCTCTCACCTGCATTTGAGGTGTGTGGGACCCGAGGGC

ACATTCCAACAGTGTTGGAGGCAGAGGGCTGGGGGCGGGGGAGCAGGGAGAGGCCAAGTGAGTGGGCAGCTGGGGCCATTGCACAAGCG

GAGAAGGACCTAAAGTACAAGAGTGAGCTTGCCGAGGTCCTGGCG
