# Supplementary material for: Chromothripsis during telomere crisis is independent of NHEJ, and consistent with a replicative origin
Source: Genome Res. 2019 May;29(5):737–49. doi: 10.1101/gr.240705.118 (PMC6499312; doi:10.1101/gr.240705.118)
Supplement: Supplemental Material [file supp_gr.240705.118_Supplemental_file_1.zip › contigs/annotated_contigs/DB113/contig.2.DB113_length_583_mean_cov_14.0600343053.docx]

**DB113_length_583_mean_cov_14.0600343053**

TTCTGCGATCAGTAAGAGTATTCTTAGGATGAAAATTGAAATAAGGGTTGAAAAGGTTAGAAACGAGTAAGTGACTTTTTTTTTTAATA
 >chr3:21705412-21705691 - E=5e-149 p=1e-03
TTATCTTTTCAGCCTTCAGATAACAACAGTGGTTGTTAAAAGGCAAGTCTGAGCTTGTCAATGACAGGACATGTCAATGCAACCAAAGC

AGAAAGGATTAACATGAGAGGTCACTCAATGTCCAGTGATCTAAATAACACAGCTTTCTTGTTGCAATATCTTGTCCCCATGATGGATT

TAAATAAAT|TCAAAGAATTTTAAGGGACTCAAATAAAAAATAGGTATTTTAAGTGACTATATATAGAGATAAGAAACTTTATTTTTTA
 >chr3:21695387-21695694 - E=4e-173
ATTTCTATATTCAAGTTTTTTTTTTAAATGGAAGGTAAAAGAAATGACAGTCCAGTTGTACAATAGATTGGGGCATTTTAAAGCAGTTT

CTTTAAAGAAATCACATAGAAAAATAATCAGTTTTATCAAAACATCTTTATGTTCTTTAGAACATCTGTACATATGTACCATGTTAATG

CTTCTGTGACTTTTACCACATAGTATGTTGCAAAGCAACAGTTTTTGCCT
