# Supplementary material for: Chromothripsis during telomere crisis is independent of NHEJ, and consistent with a replicative origin
Source: Genome Res. 2019 May;29(5):737–49. doi: 10.1101/gr.240705.118 (PMC6499312; doi:10.1101/gr.240705.118)
Supplement: Supplemental Material [file supp_gr.240705.118_Supplemental_file_1.zip › contigs/annotated_contigs/DB113/contig.2.DB113_length_583_mean_cov_8.5180102916.docx]

**DB113_length_583_mean_cov_8.5180102916**

GAACG|TGGAAGTCATCCAACGAGTGCCACAGAGTTGTGTTAGGGTGATAAGATCATAGATACATTTTTTTGTATTGTAATTGTCTATA
 >chr10:12316539-12317027 - E=5e-246 p=0e+00
AACTTGTCATTTCATACATAAAAACAAATTCAAGGCCGGGCACAGTGGCTCACGGCTATAATCCCAGCACTTTAGGAGGCCGAGGCGGG

TGGATCACTTGAACCCAGGAGTTTGAGACCAGCCTGGGCAACAGAGTGAGACTCCGTCTCTACTAACAATACAAAAATTAGCCAGGCGT

GGTGGCGGGCACCTGTAATCCCAGCTACTTCGGAGGCTGAGGCAGGAGAATTGCTTGAACTCGGGAGGTGGAGGGTGCAGTGAGCCAAG

ATCATGCCACTGCACTTCAGCCTGGGTGATAGAATGAGACACTTCGTCAAAAAATAACAT|AGGCCGGGCACGGTGGCTCACACCTGTA
 >chr15:43923559-43923727 -
ATCCCAGCACTTTGGGAGGCCGAGGCGGGCAGATCACAAGGTCAGGAGAT|TAGGATCATCCTGGCCAACATGGTGAGACCCTGTCTCT
E=2e-89
ACTAAAATACAAAAAATCAGCTGGACGTTGTGGCACATGCCTGTAGTCCCAG
