# Supplementary material for: Chromothripsis during telomere crisis is independent of NHEJ, and consistent with a replicative origin
Source: Genome Res. 2019 May;29(5):737–49. doi: 10.1101/gr.240705.118 (PMC6499312; doi:10.1101/gr.240705.118)
Supplement: Supplemental Material [file supp_gr.240705.118_Supplemental_Material.docx]

**Chromothripsis during telomere crisis is independent of NHEJ and consistent with a replicative origin**

Kez Cleal^1^, Rhiannon E. Jones^1^, Julia W. Grimstead^1^, Eric A. Hendrickson^2¶^, Duncan M. Baird^1¶*^

^1^ Division of Cancer and Genetics, School of Medicine, Cardiff University, Heath Park, Cardiff, CF14 4XN, UK.

^2^Department of Biochemistry, Molecular Biology, and Biophysics, University of Minnesota Medical School, Minneapolis, MN 55455, USA

^¶^joint senior authors

*Correspondence email: bairddm@cardiff.ac.uk

# Supplemental Methods

Table of Contents

[Supplemental Methods 1](#_Toc1134909)

[Supplemental Methods 2](#_Toc1134910)

[Cloning and DNA sequencing 2](#_Toc1134911)

[Paired-end read mapping 3](#_Toc1134912)

[Time spent in crisis 3](#_Toc1134913)

[Assessing DN-*TERT* copy number 4](#_Toc1134914)

[Copy number quantification 4](#_Toc1134915)

[Filtering of single nucleotide variants 6](#_Toc1134916)

[Estimation of clonality 6](#_Toc1134917)

[Chained structural variant identification 7](#_Toc1134918)

[Identification of fold-back inversions 8](#_Toc1134919)

[Identification of kataegis 9](#_Toc1134920)

[Mutational signatures near SV breakpoints 9](#_Toc1134921)

[Nearest neighbor analysis 10](#_Toc1134922)

[Assembly of structural variants into contigs 11](#_Toc1134923)

[Mapping of contigs to find structural variants, microhomology and insertions 11](#_Toc1134924)

[Identification of significant sequence similarity between adjacent alignments 14](#_Toc1134925)

[Verification of post-crisis contig identity by single molecule assay 15](#_Toc1134926)

[Clustering of SV signatures 16](#_Toc1134927)

[Supplemental code 17](#_Toc1134928)

[References 19](#_Toc1134929)

# Supplemental Methods

## Cloning and DNA sequencing

The HCT116 human colon cancer cell line was originally obtained from Dr Bert Vogelstein (Johns Hopkins University), they were authenticated by whole genome sequencing and mycoplasma tested using the Venor^®^GeM kit (minerva biolabs). Knockout cells and derivative complemented versions were generated using recombinant adeno-associated virus-mediated gene targeting as previously described (Oh et al. ; Jones et al. ; Oh et al. ; Liddiard et al.). To initiate telomere erosion, cells were transduced with amphotropic retroviral vectors containing a DN-*TERT* cDNA (Hahn et al.) single-cell clones were isolated under selection and cultured through the point of crisis as described previously (Preto et al. ; Jones et al. ; Liddiard et al.). Following the escape from crisis DNA was extracted using standard proteinase K, RNase A, phenol/chloroform protocols (Chong) and quantified by spectrophotometry (NanoDrop, Thermo Fisher Scientific). Whole Genome Re-sequencing was undertaken by the Beijing Genome Institute using the Illumina HiSeq X-Ten platform, 150 bp paired-end, at a mean target depth of 15x, giving an insert size of approximately 412 bp ± 93 bp s.d.

## Paired-end read mapping

Read-pairs were mapped by bwa mem (v0.7.1) using hg19 as the reference and applying the -M flag to mark secondary mappings (Li and Durbin 2009). Duplicate reads were marked using the Picard Tools library (v1.108) whilst sorting and indexing were performed using samtools (v1.2) (Li et al. 2009). We do not expect the conclusions reached within our study to be altered by mapping to GRCh38 reference, as typically only small differences are seen in the number of variants identified between references (Guo et al. 2017).

## Time spent in crisis

At each passage cell number was counted, and the frequency of cell division was calculated from the number of cells seeded during the previous passage and the growth time. The time spent in crisis was determined by assessing the depression in cell division frequency experienced during crisis. This was achieved by defining a cell division frequency threshold, calculated as the mean overall cell division frequency minus the standard deviation of the cell division frequency. The cumulative time a culture spent below this threshold was taken as the time spent in crisis.

## Assessing DN-*TERT* copy number

Read-pairs were isolated from the *TERT* locus for which both primary alignment start positions were found within exons, or within 5 bp of an exon start site. Alignments were kept if they were not marked as duplicates, secondary or supplementary. If reads were marked as a proper pair, they were only kept if each read was mapped to a separate exon and one of those reads displayed a soft-clip. Any read falling within the first two exons of the *TERT* gene was also discarded as these exons were too long to reliably separate reads derived from cDNA from genomic DNA. The copy number of the cDNA reads was then determined in relation to the median genome coverage.

## Copy number quantification

Copy number profiles were determined by assessing the depth of sequencing coverage. Mean read coverage was calculated for 10 kb non-overlapping windows over the genome to generate a raw coverage array C_R_. The GC content rounded to the nearest percentage point of each bin was determined using the hg19 reference. Mapability of each window was then calculated from the UCSC track ‘wgEncodeCrgMapabilityAlign50mer.bigWig’ using bwtool to extract mapability values before calculating a mean mapability value for the 10 kb bin (Pohl and Beato 2014; Rosenbloom et al. 2015). Mapability values were normalized to the range 0 to 100 and rounded to 1 decimal place. Bins with a GC content < 30 or > 60 %, or a mapability value < 70, or a coverage > 500 were discarded from further analysis. Bins with identical GC content and mapability values were then grouped together, and a median coverage value was calculated for that bin C_b_.

Bins were represented as a two-dimensional array A_ij_ with elements a_ij_ = C_b_ where i is the GC content (30, 60] = $\left\{ i \begin{aligned} \mathbb{\in R |}30<i \leq60, 10 i\mathbb{\in Z} \end{aligned} \right.\}$, and j is the mapability (70, 100] = $\left\{ j \begin{aligned} \mathbb{\in R |}70<j \leq100, 10 j\mathbb{\in Z} \end{aligned} \right.\}$.

The median coverage of the whole genome C_g_ was then subtracted from A_ij_ to give a distance matrix D_ij_ = A_ij_ – C_g_, representing the deviation of a bin from the genomic median. The surface of D_ij_ was then interpolated using a radial basis function (Rbf) kernel from the Scipy interpolate module (smooth value = 5) (Jones E 2001). The binned raw coverage values C_R_ could then be normalized to GC and mapability values according to v = C_R_ – d_ij_ where d_ij_ is the element from D_ij_ with GC content and mapability value equivalent to the raw bin C_R_.

As a final noise reduction step, a Haar wavelet transform was applied from the PyWavelets module (level=2, mode=’per’). Here, wavelet coefficients were thresholded using the universal threshold:

$threshold=\sigma\sqrt[2]{2 log(l)}$

Where $\sigma$ is the median absolute deviation of the noisy coefficients and $l$ is the length of the input array. This gave a normalized array of coverage values *N*.

To investigate relative CN changes with respect to parental lines, the normalized CN bins *N* of the parental line were used as a background and subtracted from values calculated for daughter samples from the same background *N*_sample_ *- N*_parental_. To estimate the absolute CN of each bin, normalized CN bins *N* were divided by the median normalized genome CN and multiplied by two to represent a diploid genome${N_{d}=2(N}/{\mathrm{Median}(N))}$.

To identify diploid regions of the genome for downstream single nucleotide variant analysis, $N_{d}$ data was used and diploid segments were identified by a median CN > 1.6 and CN < 2.4, with segments smaller than 1 Mb being discarded.

To identify CN boundaries, $N_{d}$ profiles were segmented using the copyNumber package from R with parameters gamma=600, kmin=10 (Nilsen et al. 2012).

To assess the phylogeny of CN variants, a distance matrix was created C_ij_ with elements c_ij_ = 1 / n_ij_, where n_ij_ is the number of common breakpoints between sample i and sample j. Tree reconstruction was then performed by the UPGMA (Unweighted Pair Group Method with Arithmetic Mean) algorithm implemented using the PyCogent library for Python (Knight et al. 2007).

## Filtering of single nucleotide variants

Variant quality score recalibration was run (truth sensitivity=98) (McKenna et al. 2010). Variants were filtered discarding all; indels; loci with mean cohort-coverage <5; bi- or multi-allelic sites; variants present in >1 sample; GQ<30; depth>100; overlapping UCSC simple repeat track (Benson 1999; Rosenbloom et al. 2015). Sites with one genotype of 0/1 were kept. To ensure uniqueness, SNVs were crosschecked against other samples from the same background, searching alignment files (Pysam) for reads that matched the call. If >1 read with base-quality>20 was identified in another sample, the SNV was discarded (Li et al. 2009).

## Estimation of clonality

Samples that underwent cloning post-crisis under protocols B and C were classified as monoclonal. For samples that underwent cloning via protocol A, clonality was estimated by a combination of two methods. The first method assessed the variant allele frequency (VAF method) distribution at diploid regions of the genome. Here only unique variants were selected with the expectation that a monoclonal sample will have a VAF peak centered on 0.5, and polyclonal samples will have a VAF profile skewed towards lower values.

The second method (CN method) analyzed changes in the VAF distribution in the context of a CN change, assessing both unique and non-unique variants. As HCT116 parental lines possessed preexisting CN variants, only genomic intervals that were CN2 among all parental and WT-puro samples were selected for further analysis. For each genetic background, heterozygous loci within these intervals with a genotype of 0/1 and quality > 30, and which were present in both parental and daughter samples were selected for further analysis. VAF and CN profiles were then visualized as heat maps to identify signatures of a polyclonal or monoclonal culture. Evidence of a monoclonal culture consisted of the presence of a peak at CN -1, and VAF=1.00; or two peaks at CN +1 with VAF=0.33 and 0.66. Evidence of a polyclonal culture consisted of peaks at CN -1 with a VAF < 1, or a significant deviation from integer CN values on the x-axis.

If evidence was conflicting between the two methods, the CN method took precedence due to the much larger number of variants used in the analysis. Clonality of samples was thus manually inferred based on these sets of observations, classifying samples as mono- or polyclonal, or of unknown clonality if insufficient evidence could be gathered to categorize the sample.

## Chained structural variant identification

We regarded identifying chains of SVs to be a special case of clustering. Breakpoint clustering occurs when several breaks are found near to one another. A chain of SVs includes the possibility that multiple clusters of breaks can be ‘linked’ together. We started with the assumption that chromothripsis-like rearrangements form patterns that resemble a chain of events on the reference genome. Thus, for a given chain, the spacing of neighboring SVs on the reference genome would be expected to be closer than random to give the appearance of a chain. If the spacing of neighboring breaks was below a statistical threshold, then they were considered sufficiently close to be part of a potential chain. However, as unrelated breakpoints can occur close to one another by chance, we also defined a minimum number of breakpoints to classify a chain. The definition of a chain of SVs was thus defined by the significance threshold for assessing separation distances, and the minimum number of breaks in the linked subgraph to qualify the set of SVs as a chain. To identify suitable parameters, simulations were run by shuffling break point positions for each sample along the same reference chromosomes on which they were identified. The significance threshold chosen was thus α = 0.04, and a threshold of 8 nodes was chosen for the minimum number of nodes required in the subgraph to qualify a SV chain. To define clusters of breakpoints within a chain, removal of the black edges leaves only isolated clusters of nodes that are linked with intra-cluster grey edges. A cluster of breakpoints is thus the genomic interval over a collection of nodes linked by intra-cluster grey edges. Additionally, only intervals with > 1 node qualified as a cluster.

## Identification of fold-back inversions

Inversion less than 50 kb in size were extracted from the unique call set with paired-end support of ≥ 3 reads and a MAPQ ≥ 2. Only inversions mapping to the ‘normal’ human reference chromosomes (chr1 … chrY) were kept. Read depth was then quantified in 150 bp bins across the inverted segment; *inv_D_*. Additionally, the depth of 20 kb of sequence before *b_D_* and after *a_D_* the inversion start and end coordinates was also quantified. Inversions with a mean depth ≥ 75 over these three windows were discarded. Only inversions with a characteristic step pattern were kept for further analysis such that *b_D_* > *inv_D_* > *a_D_*, or *b_D_* < *inv_D_* < *a_D_*. T-tests were performed to assess the significance of each step in the CN profile, t-test (*b_D_*, *inv_D_*), t-test (*inv_D_*, *a_D_*), and inversions with a maximum p-value ≥ 0.02 were discarded.

## Identification of kataegis

Kataegis clusters were identified according to criteria outline in (Roberts et al. 2013). Using the unique SNV dataset, APOBEC (apolipoprotein B mRNA-editing enzyme, catalytic polypeptide-like) cytidine deaminases mutation signatures were identified as clusters of SNVs on the same strand that occur in TCW motifs where W corresponds to either the bases T or A (Smith et al. 2012). Clusters were identified by grouping together mutations which fell ≤ 10 kb of one another. Next, tightly spaced mutations with < 10 bp separation were categorized as complex and considered to arise in a single mutation event (Roberts et al. 2013). Complex events were only counted once, and all groups with ≥ 2 events were kept for further analysis. The cluster p-value was determined using a negative binomial distribution:

$$cluster p-value=\sum_{j=0}^{x-k} \left( \frac{\left( k-1 \right)+j-1}{j} \right)\left( 1-\pi\right)^{j} \pi^{k-1}$$

Where x is the cluster interval size in bases, k is the number of mutation events in the cluster, and π is the genomic mutation probability defined as $\frac{n}{G}$, where n is the number of genomic mutations and G is the size of the genome. Subsequently, clusters with a p-value ≥ 1 x10^-3^ were discarded.

## Mutational signatures near SV breakpoints

To analyze mutational signatures of SNVs, first all SNVs that were not classified as SV-associated were grouped into a final class labelled as ‘Other’. Mutational signatures were then delineated using the deconstructSigs package with parameters signatures.cutoff=0.06, signatures.ref=signatures.cosmic (Rosenthal et al. 2016).

The value of the deconstructSigs parameter ‘signatures.limit’ which controls the limit on the number of signatures reported, was determined using a procedure based on leave-p-out-cross-validation. Data was partitioned into 10 overlapping sets, such that each set consisted of approximately 90% of the original data. For each set, this was achieved by removing every 10^th^ + *i* data point, with *i* ranging from (0, 10] for each partition. We observed that setting the signatures.limit parameter to an arbitrarily high value, typically resulted in the software reporting a set of signatures with varying stability, with major signature components tending to be common across all partitions, whilst minor signature components tending to fluctuate in their identity and weights. We therefore minimized the signatures.limit value so that all reported signatures showed a high level of stability across all partitions. This was achieved by first ranking the signature-weights array (corresponding to the weight of each of the 30 predefined signatures in each partition), resulting in a ranked array A_i,j_ of size i=10 and j=30. The relative frequency of the mode across columns A_j_ was then calculated according to $f_{M}={n_{j}}/{10}$, where $n_{j}$ is the number of rows with value equal to the mode. For each signature, the value of $f_{M}$ corresponded to how frequently the most common rank is observed across partitions. The signatures.limit parameter was minimized so that $\min\left( f_{M} \right)\geq0.7$, meaning that for a given signature in the output, at least 70% of partitions displayed the same signature at the same rank.

## Nearest neighbor analysis

For each sample, nearest neighbor distances were determined for intra-cluster breakpoints. For two points x and y which are both nearest neighbors to each other, the distance d_xy_ was only included once to avoid violating the assumed independence of nearest neighbor distances.

## Assembly of structural variants into contigs

Secondary alignments were discarded and high coverage regions were randomly down-sampled to 1000 reads. Collected reads were further down-sampled using kmer (v2.0; parameters -k21 -C20 -x5e8)(Crusoe et al. 2015), prior to assembly using SPAdes (v3.10.1; parameters -k21,33,55,77,111 –careful -s) (Bankevich et al. 2012). Contigs >150 bp in length were kept. Input reads were then mapped to contigs using bwa mem. The coverage of each contig was determined from reads with >125 bp of aligned sequence. Contigs with a mean coverage >45 were discarded. Contigs were marked for simple repeats using tantan (v13) and discarded if ≥80 % of bases were deemed repetitive (Frith 2011).

## Mapping of contigs to find structural variants, microhomology and insertions

Contigs were mapped to hg19 using the LAST mapper (v852; parameters -r1 -q4 -a6 -b1 -D1000 -P6 -K3 -C3) and alignments with an identity below 0.86 were discarded, to generate a set of candidate alignments. An optimal set of alignments was chosen by selecting the longest path in a directed acyclic graph, where nodes represented alignments and edges represented the joining of two alignments. The longest paths is then informed by a set of edge weights. Edge weight is calculated as the alignment score of the downstream vertex (vertex v) minus the edge cost associated with the joining of those alignments. Edge costs were chosen based on the initial scoring scheme for the LAST mapper, of which the match score of +1 per aligned base was the most pertinent. A list of edge costs and their chosen values are given below.

| Cost type | Value | Description |
| --- | --- | --- |
| Intra-chromosomal | 14 | Given to an edge which represents and intra-chromosomal SV |
| Inter-chromosomal | 15 | Given to an edge which represents an inter-chromosomal SV. This cost is 1 point higher than the intra-chromosomal cost to resolve potential ties with intra-chromosomal alignments. |
| Insertion | 1 | A gap between successive query alignments represents an insertion. A cost is associated with an insertion to penalize large gaps between query alignments. |
| Microhomology | 3 | Microhomology is defined as the overlap of two query alignments to the reference genome. Overlapping alignments are therefore counted twice when totaling the longest path distance. A microhomology cost is thus introduced to counter this property and prevent extreme overlaps of query alignments. |

Edges are added between vertices u and v if the query start coordinates v_Qstart_ > u_Qstart_. Additionally, an edge is only added between u and v if the query end coordinates of v are > 20 bp beyond the query end coordinates of u such that v_Qend_ – u_Qend_ > 20 bp.

As unmapped sequence may occur at the start or end of the contig sequence, two special vertices are added to the graph which represent the beginning and end of the sequence; u_start_, v_end_. These vertices have an alignment score of zero and serve to penalize unmapped sequence at the start or end of the contig. The optimal path is then the longest path from u_start_ to v_end_, and can be defined by a recursive function;

distance(v_end_) = max _(u, v)∈E_ {distance(u) + weight _(u, v)_}

Where E is the set of edges, max _(u, v)_ is the longest incident path on v, and weight _(u, v)_ is defined by the edge type;

weight _(u, v)_ = A_v_ $-$ $\left\{ \begin{aligned} 14+3M +I if (u, v) is intra-chromosomal \\ 15+3M +I \mathrm{if}\left( u, v \right) is inter-chromosomal \\ I \mathrm{otherwise} \end{aligned} \right.$

Where A_v_ is the alignment score of vertex v, *M* is the length of microhomology and *I* is the length of insertion in base pairs. This algorithm is available online: https://github.com/kcleal/fnfi, and can be invoked using the ‘fnfi align’ command.

Following identification of the optimal path, contigs were further filtered, keeping those with a mean identity > 0.95 and a path distance > 0. For contigs with ≤ 2 alignments, one alignment was required against the ‘normal’ chromosomes found in hg19 {chr1 ... chrY}. For contigs with > 2 alignments, at least 2 alignments were required against the ‘normal’ human reference chromosomes, else the contig was discarded. Contigs which shared any alignments with those derived from other samples were also discarded. Finally, any contigs with alignments to the *TERT* locus chr5:1251850-1295754 or the Ligase 3 locus chr17:33306147-33332286 were discarded as they were found to map vector cDNA.

## Identification of significant sequence similarity between adjacent alignments

To identify patches of sequence similarity between successive query alignments, pairwise alignment was performed using the Smith-Waterman algorithm from Scikit-bio (v0.4.2, parameters gap_open_penalty=10, gap_extend_penalty=10) (Zhao et al. 2013). For neighboring alignments with microhomology, the microhomology sequence was included in both sequences when performing pairwise alignment. For sites with an insertion between successive alignments, the origin of the insertion is ambiguous and may presumably arise from the action of a polymerase at one side of the break. For such sites, two pairwise alignments were generated, with the insertion included at the left-hand sequence and then aligned to the right-hand sequence, or the insertion included at the right-hand sequence and aligned to the left-hand sequence. These two pairwise comparisons were then scored using the output alignment according to:

$$score=1-\left( 1- \prod_{i=1}^{r} m_{i} \right)^{n-r+1} m_{i}\left\{ \begin{aligned} 0.25 if match \\ 0.75 otherwise \end{aligned} \right.$$

Where r is the length of the pairwise alignment, n is the length of the longest input sequence, and $m_{i}$ is the base at index i of the pairwise alignment and can correspond to a match or mis-matched base. The arrangement with the lowest score was then selected for further analysis.

For each stretch of sequence similarity identified by pairwise alignment a probability value was estimated for the observed alignment scores by Monte Carlo simulation. For input alignments with lengths *l*1 and *l*2, 1000 pairs of random sequences were drawn from {A, T, C, G} with length *l*1 and *l*2 and aligned using Smith-Waterman to generate a distribution of alignment scores. The probability of obtaining an equivalent or higher alignment score was then calculated using the cumulative density function: $P(X \geq a)=1-CDF (s)$, where s is the proportion of simulated alignment scores with values less than the observed score. Patches of sequence similarity with a probability < 0.05 were regarded as significant.

## Verification of post-crisis contig identity by single molecule assay

Oligonucleotide PCR primers were designed from the DB53_1501 contig sequence (Supplemental Fig. S24A) DB53_1501F: AACATTGTTGCCTACACACGAG; DB53_1501R1: GTAAGCAGGATTCTTCAGCAAC. DNA from *LIG3*^-/-:NC3^: sample 53 was used to generate a 704 bp amplicon spanning 8 breakpoints, the amplicon was not detected in control DNA obtained from the cancer cell line MCF7 or in a PCR with no DNA (data not shown). PCR conditions were as follows: 10 μl volumes containing between 50 ng of DNA, 0.5 μM of the telomere-adjacent primers, 1.2 mM NTPs, 75 mM Tris-HCl pH 8.8, 20 mM (NH_4_)_2_SO_4_, 0.01 % Tween-20, 1.5 mM MgCl2, and 1U of Taq polymerase. Cycled under the following conditions: 33 cycles of 94 °C for 15 seconds, 54°C for 30 seconds and 72°C for 1 min. The resulting 704 bp PCR product was gel purified to be used as a hybridization probe. To detect the amplicon at the single molecule level, the parental cells (*LIG3*^-/-:NC3^) were analyzed in isolation (50ng/reaction and 10ng/reactions) or spiked with dilutions of *LIG3*^-/-:NC3^: sample 53 from post-crisis cells. The DNA fragments were resolved by 1% TAE agarose gel electrophoresis, and detected by Southern hybridization with a random-primed α-^32^P labeled (GE Healthcare Life Sciences) probe generated from the purified PCR product and a probe to detect the 1kb molecular weight marker (Stratagene, La Jolla, USA). The hybridized fragments were detected by phosphor imaging with Typhoon FLA 9500 phosphoimager (GE Healthcare Life Sciences).

## Clustering of SV signatures

As we did not find statistical differences between genetic backgrounds in terms of microhomology or insertions usage, we performed a pooled analysis of all backgrounds. SVs were classified into 8 categories, subdividing SVs according to their status as chained vs non-chained, followed by complex (referring to more than one SV identified on the parent contig) vs simple, and finally into intra-chromosomal vs inter-chromosomal events. Agglomerative clustering (linkage=’average’) was then performed using the Scikit-learn python package, using the mean insertion, microhomology, mis-match rate and indel rates of each category (Pedregosa et al. 2011).

# **Supplemental code**

**Algorithm to select optimal set of alignments**

#cython: language_level=2, boundscheck=False, wraparound=False

**import** **array**

**from** **cpython** **cimport** **array**

**import** **numpy** **as** **np**

cimport numpy **as** np

DTYPE = np.float

ctypedef np.float_t DTYPE_t

**def** **optimal_path**(np.ndarray[DTYPE_t, ndim=**2**] segments, float contig_length,

float max_insertion=**1500**, float min_aln=**20**,

float max_homology=**1500**, float ins_cost=**1**, float hom_cost=**3**,

float inter_cost=**15**, float intra_cost=**14**):

"""

Choose a set of candidate alignments from a list. Input is a single set of alignments in

Numpy array float format. Input array must be pre-sorted by Query_start.

:param segments: Numpy 2d float array. Rows correspond to alignments, with values;

[[Chromosome_key, Query_start, Query_end, Alignment_score], ...]

:param contig_length: Length of the input contig in base pairs

:param max_insertion: Maximum contig insertion allowed (bp)

:param min_aln: Minimum alignment length to consider (bp)

:param max_homology: Maximum homology to consider (bp)

:param ins_cost: Insertion cost

:param hom_cost: Microhomology cost

:param inter_cost: Inter-chromosomal cost

:param intra_cost: Intra-chromosomal cost

:return: tuple; Indexes of the input array that were chosen, score of chosen set

"""

cdef array.array int_array_template = array.array('i', [])

cdef array.array float_array_template = array.array('f', [])

cdef int i, j, p, end_i, next_i

cdef float current_chrom, current_start, current_end, next_chrom, next_start, \

next_end, jump_cost, next_score, micro_h, ins, best_score, current_score, sc, max_s

pred = array.clone(int_array_template, len(segments), zero=True)

node_scores = array.clone(float_array_template, len(segments), zero=True)

# Deal with first score

node_scores[**0**] = segments[**0**, **3**] - (segments[**0**, **1**] * ins_cost)

pred[**0**] = -**1**

# Choose the best score out of the edges.

# Start from segment two because the first has been scored

**for** i **in** range(**1**, len(segments)):

next_chrom = segments[i, **0**]

next_start = segments[i, **1**]

next_end = segments[i, **2**]

next_score = segments[i, **3**]

p = -**1** # Predecessor

# Walking backwards mean the search may be terminated at some point

**for** j **in** range(i-**1**, -**1**, -**1**):

current_chrom = segments[j, **0**]

current_start = segments[j, **1**]

current_end = segments[j, **2**]

current_score = segments[j, **3**]

# Allow alignments with minimum sequence and max overlap

**if** next_start > current_end - max_homology **and** next_end > current_end + min_aln **and** \

next_start - current_start > min_aln:

**if** next_start > current_end **and** next_start - current_end > max_insertion:

**continue**

micro_h = current_end - next_start

ins = **0**

**if** micro_h < **0**:

ins = abs(micro_h)

micro_h = **0**

**if** next_chrom == current_chrom:

jump_cost = intra_cost

**else**:

jump_cost = inter_cost

current_score = node_scores[j] - (micro_h * hom_cost) - (ins * ins_cost) - jump_cost + next_score

**if** current_score > best_score:

best_score = current_score

p = j

node_scores[i] = best_score

pred[i] = p

# Update the score for jumping to the end of the sequence

max_s = -**1e6**

end_i = **0**

**for** i **in** range(len(segments)):

sc = node_scores[i] - (ins_cost * (contig_length - segments[i, **2**]))

**if** sc > max_s:

max_s = sc

end_i = i

# Get path from max

indexes = [end_i]

**while** True:

next_i = pred[indexes[len(indexes) - **1**]]

**if** next_i == -**1**:

**break**

indexes.append(next_i)

**return** indexes[::-**1**], max_s

Note the above function is written in ‘.pyx’ format and must be compiled using Cython (https://cython.org/) before being used from Python.

best_score = next_score - (next_start * ins_cost) # Worst case

# Walking backwards; visit incoming edges on the graph

**for** j **in** range(i-**1**, -**1**, -**1**):

current_chrom = segments[j, **0**]

current_start = segments[j, **1**]

current_end = segments[j, **2**]

current_score = segments[j, **3**]

# Allow alignments with >minimum sequence extension and <max overlap

**if** next_start > current_end - max_homology **and** next_end > current_end + \

min_aln **and** next_start - current_start > min_aln:

**if** next_start > current_end **and** next_start - current_end > max_insertion:

**continue**

micro_h = current_end - next_start

ins = **0**

**if** micro_h < **0**:

ins = abs(micro_h)

micro_h = **0**

**if** next_chrom == current_chrom:

jump_cost = intra_cost

**else**:

jump_cost = inter_cost

current_score = node_scores[j] - (micro_h * hom_cost) - \

(ins * ins_cost) - jump_cost + next_score

**if** current_score > best_score:

best_score = current_score

p = j

node_scores[i] = best_score

pred[i] = p

# Update the score for jumping to the end of the sequence

max_s = -**1e6**

end_i = **0**

**for** i **in** range(len(segments)):

sc = node_scores[i] - (ins_cost * (contig_length - segments[i, **2**]))

**if** sc > max_s:

max_s = sc

end_i = i

# Get path from max

indexes = [end_i]

**while** True:

next_i = pred[indexes[len(indexes) - **1**]]

**if** next_i == -**1**:

**break**

indexes.append(next_i)

**return** indexes[::-**1**], max_s

# **References**

Picard Tools. p. v1.108.

Bankevich A, Nurk S, Antipov D, Gurevich AA, Dvorkin M, Kulikov AS, Lesin VM, Nikolenko SI, Pham S, Prjibelski AD et al. 2012. SPAdes: a new genome assembly algorithm and its applications to single-cell sequencing. *J Comput Biol* **19**: 455-477.

Chong L. 2001. Molecular cloning - A laboratory manual, 3rd edition. *Science* **292**: 446-446.

Crusoe MR, Alameldin HF, Awad S, Boucher E, Caldwell A, Cartwright R, Charbonneau A, Constantinides B, Edvenson G, Fay S et al. 2015. The khmer software package: enabling efficient nucleotide sequence analysis. *F1000Res* **4**: 900.

Frith MC. 2011. A new repeat-masking method enables specific detection of homologous sequences. *Nucleic Acids Research* **39**.

Hahn WC, Stewart SA, Brooks MW, York SG, Eaton E, Kurachi A, Beijersbergen RL, Knoll JH, Meyerson M, Weinberg RA. 1999. Inhibition of telomerase limits the growth of human cancer cells. *Nat Med* **5**: 1164-1170.

Hunter JD. 2007. Matplotlib: A 2D graphics environment. *Comput Sci Eng* **9**: 90-95.

Jones E OE, Peterson P. 2001. SciPy: Open Source Scientific Tools for Python.

Jones RE, Oh S, Grimstead JW, Zimbric J, Roger L, Heppel NH, Ashelford KE, Liddiard K, Hendrickson EA, Baird DM. 2014. Escape from telomere-driven crisis is DNA ligase III dependent. *Cell Rep* **8**: 1063-1076.

Knight R, Maxwell P, Birmingham A, Carnes J, Caporaso JG, Easton BC, Eaton M, Hamady M, Lindsay H, Liu ZZ et al. 2007. PyCogent: a toolkit for making sense from sequence. *Genome Biol* **8**.

Krzywinski M, Schein J, Birol I, Connors J, Gascoyne R, Horsman D, Jones SJ, Marra MA. 2009. Circos: An information aesthetic for comparative genomics. *Genome Research* **19**: 1639-1645.

Li H, Durbin R. 2009. Fast and accurate short read alignment with Burrows-Wheeler transform. *Bioinformatics* **25**: 1754-1760.

Li H, Handsaker B, Wysoker A, Fennell T, Ruan J, Homer N, Marth G, Abecasis G, Durbin R, Proc GPD. 2009. The Sequence Alignment/Map format and SAMtools. *Bioinformatics* **25**: 2078-2079.

Liddiard K, Ruis B, Takasugi T, Harvey A, Ashelford KE, Hendrickson EA, Baird DM. 2016. Sister chromatid telomere fusions, but not NHEJ-mediated inter-chromosomal telomere fusions, occur independently of DNA ligases 3 and 4. *Genome Res* **26**: 588-600.

Nilsen G, Liestol K, Van Loo P, Vollan HKM, Eide MB, Rueda OM, Chin SF, Russell R, Baumbusch LO, Caldas C et al. 2012. Copynumber: Efficient algorithms for single- and multi-track copy number segmentation. *Bmc Genomics* **13**.

Oh S, Harvey A, Zimbric J, Wang YB, Nguyen T, Jackson PJ, Hendrickson EA. 2014. DNA ligase III and DNA ligase IV carry out genetically distinct forms of end joining in human somatic cells. *DNA Repair* **21**: 97-110.

Oh S, Wang YB, Zimbric J, Hendrickson EA. 2013. Human LIGIV is synthetically lethal with the loss of Rad54B-dependent recombination and is required for certain chromosome fusion events induced by telomere dysfunction. *Nucleic Acids Research* **41**: 1734-1749.

Pedregosa F, Varoquaux G, Gramfort A, Michel V, Thirion B, Grisel O, Blondel M, Prettenhofer P, Weiss R, Dubourg V et al. 2011. Scikit-learn: Machine Learning in Python. *J Mach Learn Res* **12**: 2825-2830.

Pohl A, Beato M. 2014. bwtool: a tool for bigWig files. *Bioinformatics* **30**: 1618-1619.

Preto A, Singhrao SK, Haughton MF, Kipling D, Wynford-Thomas D, Jones CJ. 2004. Telomere erosion triggers growth arrest but not cell death in human cancer cells retaining wild-type p53: implications for antitelomerase therapy. *Oncogene* **23**: 4136-4145.

Roberts SA, Lawrence MS, Klimczak LJ, Grimm SA, Fargo D, Stojanov P, Kiezun A, Kryukov GV, Carter SL, Saksena G et al. 2013. An APOBEC cytidine deaminase mutagenesis pattern is widespread in human cancers. *Nature Genetics* **45**: 970-+.

Rosenbloom KR, Armstrong J, Barber GP, Casper J, Clawson H, Diekhans M, Dreszer TR, Fujita PA, Guruvadoo L, Haeussler M et al. 2015. The UCSC Genome Browser database: 2015 update. *Nucleic Acids Research* **43**: D670-D681.

Rosenthal R, McGranahan N, Herrero J, Taylor BS, Swanton C. 2016. deconstructSigs: delineating mutational processes in single tumors distinguishes DNA repair deficiencies and patterns of carcinoma evolution. *Genome Biol* **17**.

Smith HC, Bennett RP, Kizilyer A, McDougall WM, Prohaska KM. 2012. Functions and regulation of the APOBEC family of proteins. *Semin Cell Dev Biol* **23**: 258-268.

Zhao MY, Lee WP, Garrison EP, Marth GT. 2013. SSW Library: An SIMD Smith-Waterman C/C plus plus Library for Use in Genomic Applications. *Plos One* **8**.
